# Gauging the Strength of the Molecular Halogen Bond via Experimental Electron Density and Spectroscopy†

**DOI:** 10.1021/acsomega.3c00619

**Published:** 2023-06-05

**Authors:** Felix Otte, Johannes Kleinheider, Bastian Grabe, Wolf Hiller, Franziska Busse, Ruimin Wang, Nora M. Kreienborg, Christian Merten, Ulli Englert, Carsten Strohmann

**Affiliations:** ‡ Inorganic Chemistry, TU Dortmund University, Otto-Hahn-Str. 6, 44227 Dortmund, Germany; § Faculty of Chemistry and Chemical Biology, TU Dortmund University, Otto-Hahn-Str. 4a, 44227 Dortmund, Germany; ∥ Inorganic Chemistry, RWTH Aachen University, Landoltweg 1, 52056 Aachen, Germany; ⊥ Institute of Molecular Science, Shanxi University, Wucheng Road 92, 030006 Taiyuan, P. R. China; # Organic Chemistry II, Ruhr University Bochum, Universitätstraße 150, 44801 Bochum, Germany

## Abstract

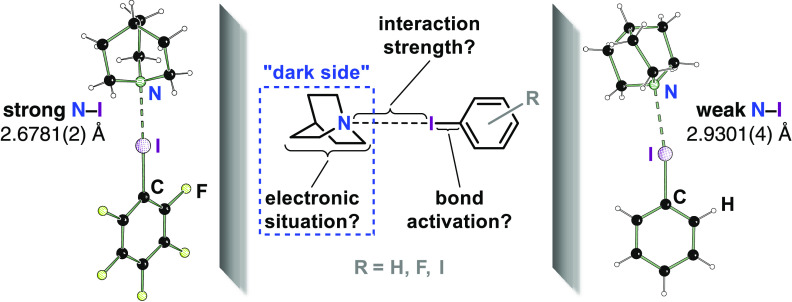

Strong and weak halogen bonds (XBs) in discrete aggregates involving the same acceptor are addressed by experiments in solution and in the solid state. Unsubstituted and perfluorinated iodobenzenes act as halogen donors of tunable strength; in all cases, quinuclidine represents the acceptor. NMR titrations reliably identify the strong intermolecular interactions in solution, with experimental binding energies of approx. 7 kJ/mol. Interaction of the σ hole at the halogen donor iodine leads to a redshift in the symmetric C–I stretching vibration; this shift reflects the interaction energy in the halogen-bonded adducts and may be assessed by Raman spectroscopy in condensed phase even for weak XBs. An experimental picture of the electronic density for the XBs is achieved by high-resolution X-ray diffraction on suitable crystals. Quantum theory of atoms in molecules (QTAIM) analysis affords the electron densities and energy densities in the bond critical points of the halogen bonds and confirms stronger interaction for the shorter contacts. For the first time, the experimental electron density shows a significant effect on the atomic volumes and Bader charges of the quinuclidine N atoms, the halogen-bond acceptor: strong and weak XBs are reflected in the nature of their acceptor atom. Our experimental findings at the acceptor atom match the discussed effects of halogen bonding and thus the proposed concepts in XB activated organocatalysis.

## Introduction

A halogen bond (XB) denotes a close contact between a usually heavy halogen atom (the XB donor) and a nucleophile such as N, O, or another halogen (the XB acceptor).^1−3^ A picture at sub-atomic resolution is commonly invoked to understand this attractive interaction between two electronegative atoms: the XB donor shows a more positively charged region opposite to its σ bond, the so-called σ hole. This electrophilic region of the heavy halogen interacts with the nucleophile, resulting in a linear arrangement about the XB donor:^4−6^ the sub-atomic picture from theory is reflected in a characteristic XB geometry!^7^

Similar to hydrogen bonds, halogen bonds cover a wide range of interaction energies and have found applications in a variety of fields.^8−10^ Many experimental studies on halogen bonds focus on geometry as outlined above: a short intermolecular contact between an often C-bonded XB donor and an electronegative acceptor atom with a lone pair, with a C–donor···acceptor angle of ca. 180° can be reliably identified by diffraction experiments at standard resolution. These experiments often address extended structures, and the formation of XBs can be triggered by suitably chosen constituents. In this sense, crystal engineering represents an increasingly popular domain of halogen bonding,^11−16^ and its results may shed light on the subtle balance between different secondary interactions.^17^ In favorable cases, the very orientation of interacting molecules may suggest an experimental answer concerning the dominant force, e.g., π–π stacking vs XB.^18^ In principle, diffraction can provide information beyond molecular geometry: X-rays interact with the electrons in a (crystalline) solid. At sufficiently high resolution, precise experiments on flawless crystals can measure the experimental electron density and its derived properties such as the electrostatic potential (ESP), and such experiments may disprove or confirm the sub-atomic models from theory. In practice, however, only a small subset of diffraction experiments meets the requirements for an experimental charge density study and geometry arguments prevail.

The electronic structure of the XB donor varies with chemical substitution. Electron-withdrawing substituents close to the XB donor will intuitively increase the σ hole and lead to stronger halogen bonds; for the same XB donor and acceptor atoms, shorter contacts may be anticipated. For the very short I···N halogen bond in the cocrystal of 4-(dimethylamino)pyridine with 1,4-diiodotetrafluorobenzene (DITFB),^19,20^ the elongation of the C–I σ bond and the concomitant redshift in the Raman spectrum could be shown.^21^

**Figure 1:**
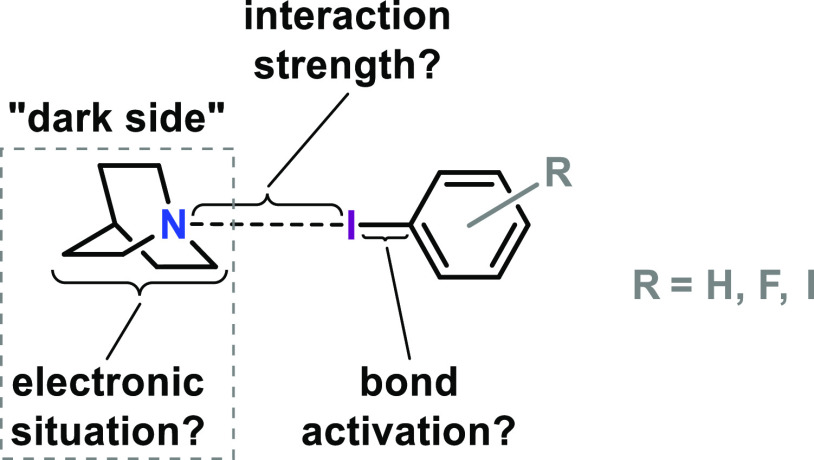
Chemical substitution transmitted via the halogen bond.

In this contribution, we take the next step toward the “dark side” highlighted in Figure 1 and investigate whether and to what extent chemical substitution of the XB donor can affect the electronic situation in the acceptor, at or in close proximity of which most XB activated organocatalytic reactions occur.^22−24^ Previously, theoretical studies of simulated spectroscopic properties^25,26^ had suggested an effect of donor variation upon the electronic situation at the acceptor side. We here provide an experimental answer!

**Figure 2:**
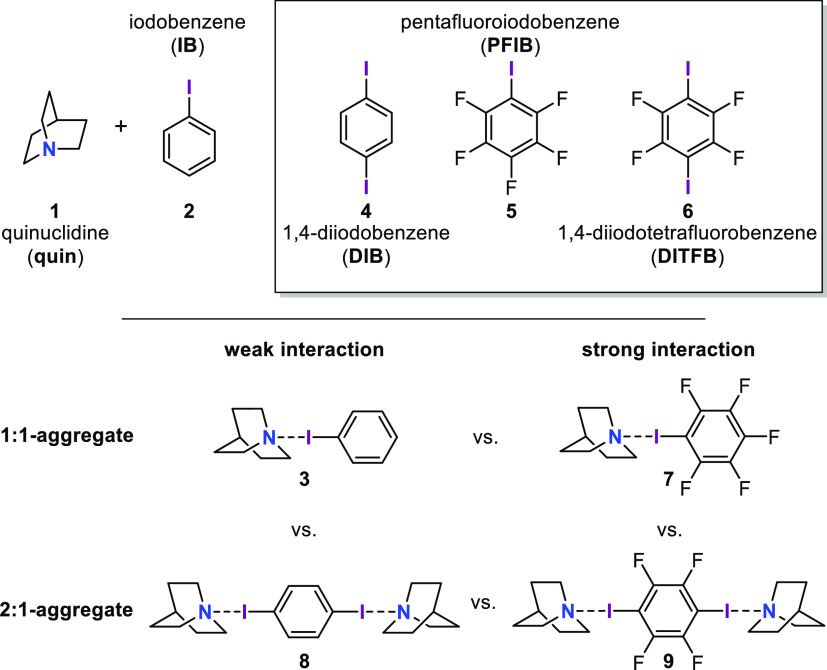
XB donor and acceptor components and their aggregates; experimental electron densities have been determined for **3**–**9**.

For this purpose, we deliberately focus on molecular aggregates rather than on extended structures: For such discrete objects, studies in solution can complement diffraction. In addition to classical one-dimensional (1D) techniques, two-dimensional (2D) methods and bond titrations offer insights into interactions in solution.^27−29^ We characterize the XBs using a combination of NMR and vibrational spectroscopy with molecular geometry. Experimental electron density studies based on high-resolution X-ray diffraction play a key role in this context: on the one hand, they clearly document the electronic situation of the XB donor as a function of chemical substitution. On the other hand, they may prove sufficiently sensitive to measure the subtle effect on the XB acceptor transmitted via the halogen bond. In case of success, this approach may not only find analytical applications in evaluating XBs but also allow us to understand bond activation as a consequence of short directional intermolecular interactions. To the best of our knowledge, no attempt has yet been made to measure the effect of different XB donors on the same acceptor via experimental electron density. Our attempt to address this question makes use of the molecular components summarized in Figure 2.

We use quinuclidine (**1**) as XB acceptor in all cases; in order to achieve the most meaningful comparisons with respect to XB geometry and electron density, our XB donor atom is arene-bonded iodine.

## Results and Discussion

### Halogen Bond Geometry

**Figure 3:**
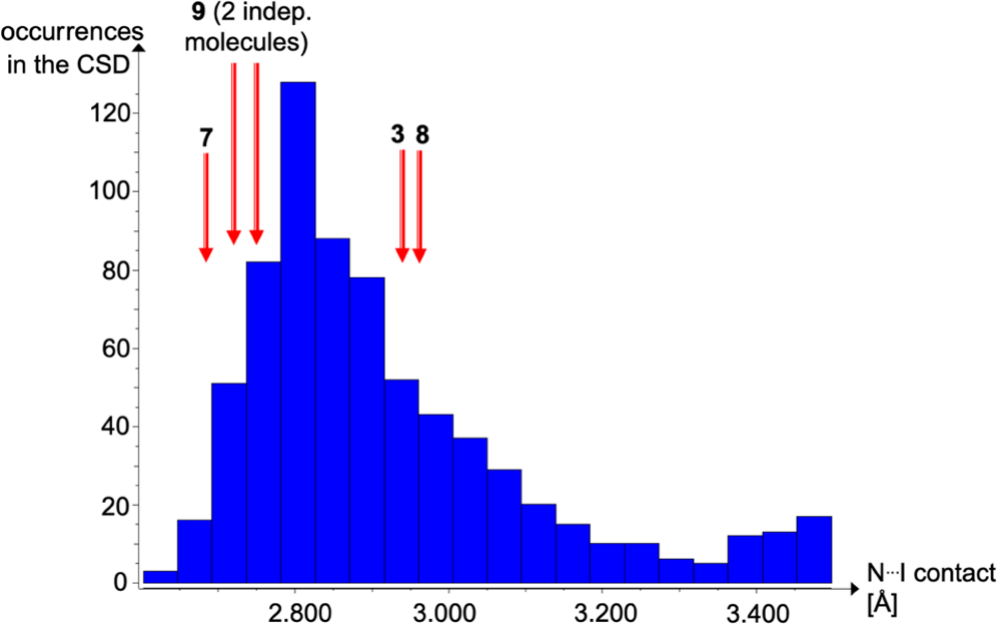
Histogram of N···I contact distances in the CSD;^30^ halogen bonds reported in this work are marked by red arrows.

The halogen donors **2** and **4**–**6** form classical molecular crystals. Their structures are discussed in the Supporting Information (SI), and we will come back to selected short contacts in the section about experimental charge density. In their quinuclidine adducts **3** and **7**–**9**, N···I halogen bonds represent the only contacts significantly shorter than the sum of the van der Waals radii of the partaking atoms. These N···I halogen bonds range between 2.6778(3) and 2.9560(3) Å, and Figure 3 puts them into the context of comparable structures deposited with the CSD.^30^ The histogram includes 715 N···I contacts shorter than 3.5 Å in error-free crystal structures without disorder in which the halogen donor I is bonded to a single carbon atom and in which the halogen acceptor N is bonded to at least two carbon atoms. Van der Waals radii should, of course, not be taken as hard limits. 3.48 Å corresponds to the shortest radii sum suggested in a recent compilation,^31^ and our histogram at roughly this distance region features an increase in contact occurrences.

**Figure 4:**
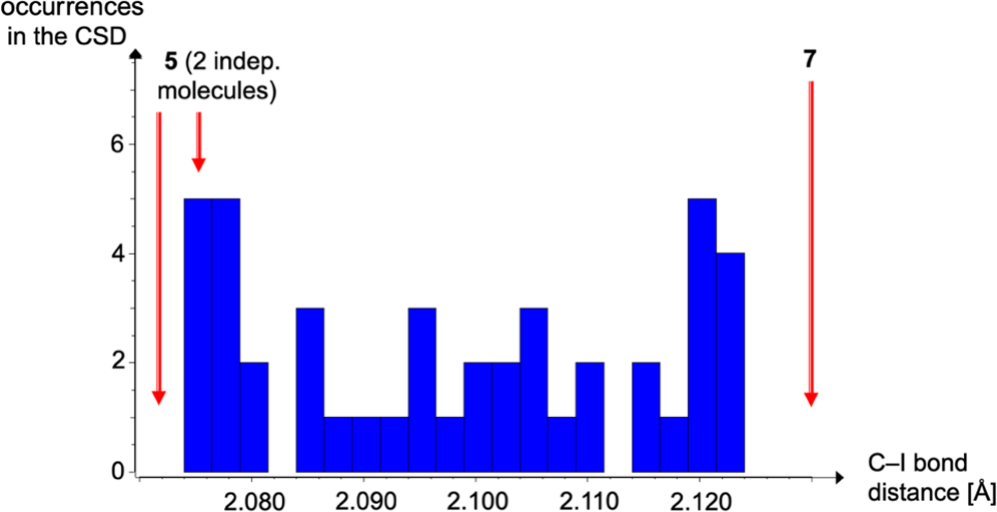
Histogram of C–I distances in PFIB structures from the CSD^30^ with C–I in **5** and **7** marked by red arrows.

The histogram in Figure 3 allows us to address the N···I contacts in **7** and **9** as short and those in **3** and **8** as unexceptional but it does not distinguish between discrete motifs and extended structures. The latter seem particularly popular in crystal engineering, an important driving force behind the structural studies of halogen bonds.^32^ Short and presumably strong halogen bonds have been associated with increasing three-center-four-electron bond character,^20,33^ and the effect of this type of bonding should be reflected in a longer C–I bond distance of the halogen-bond donor. The fluorinated XB donors **6** and, to a lesser extent, **5** have been used as building blocks in co-crystals, and we can therefore compare the C–I in these constituents not only to their halogen-bonded counterparts **9** and **7** but also to their deposited structures in the CSD. The graphical summary for PFIB structures is shown in Figure 4 and confirms the predicted trend: the halogen bond in **7**, among the shortest contacts in Figure 3, leads to an exceptionally long C–I bond.

We emphasize that an experimental verification of the above-mentioned anticorrelation between short I···N contacts and long I–C bonds must refer to a histogram or be restricted to low-temperature diffraction data of elevated resolution; individual results of standard diffraction experiments will be hardly significant. We have explained in a previous report^21^ that I···N contacts with their strong electrostatic contribution cover a much wider distance range than covalent I–C bonds. Both parameters are, however, associated with comparable standard uncertainties. In the case of standard X-ray diffraction studies, the vast majority behind the histograms, I–C distances show a spread of 0.05 Å and experimental uncertainties of up to 0.01 Å, and the resulting correlation is necessarily noisy.

**Figure 5:**
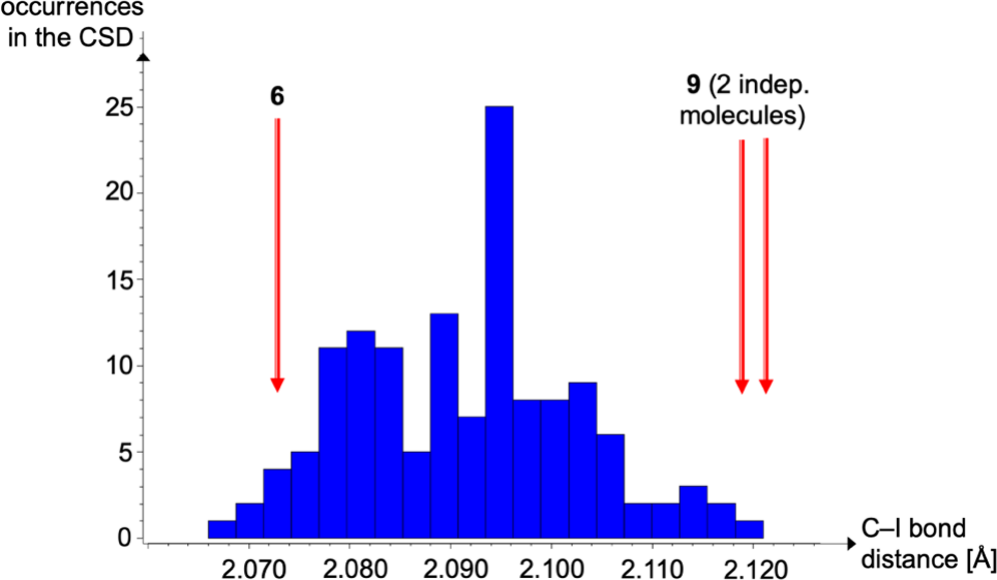
Histogram of C–I distances in DITFB structures from the CSD^30^ with C–I in **6** and **9** marked by red arrows.

As mentioned above, DITFB (**6**) represents a potential building block for extended structures and therefore has been used more often in crystal engineering. The corresponding histogram in Figure 5 therefore contains more examples of high-quality structures from the CSD. The effect of halogen bonding is also clearly visible: both symmetrically independent short N···I contacts (Figure 3) in **9** result in very long C–I bonds in the associated XB donors (Figure 5).

No meaningful histograms can be provided for the XB donors **2** and **4** because less than 10 structural studies involving these molecules have been deposited in the CSD; the associated refcodes and distances are compiled in the SI. As all diffraction experiments were performed under very similar conditions, even minor trends may be perceived: we can compare the C–I bond distances in these XB donor molecules to their weakly halogen-bonded quinuclidine adducts **3** and **8**. In either case, even the longer N···I contacts of ca. 2.95 Å lead to elongated C–I bonds, albeit the effect is much less pronounced as in the above-mentioned cases of stronger XBs. The C–I bond distance in iodobenzene increases from 2.100(5) Å in **2** to 2.1111(4) Å in the adduct **3**, and the C–I distance in 1,4-diiodobenzene from 2.0944(6) Å in **4** to 2.1098(3) Å in **8**.

### Experimental Electron Density and Diffraction

Diffraction affords the electron density distribution. Advanced experiments may provide a picture at sub-atomic resolution and thus directly reflect the effects of intra- and intermolecular bonding. Experimental electron densities obtained from high-resolution diffraction experiments are often analyzed according to Bader’s Quantum theory of atoms in molecules (QTAIM).^34^Table 1 summarizes key quality indicators for the conventional spherical structure models (the so-called independent atom model, IAM) and the multipole model (MM) on which the QTAIM is based. This compilation shows that our diffraction experiments on **4**–**9** qualify for such an advanced analysis of the electron density. Partitioning of the electron density distribution into atomic basins and integration over the associated volumes affords QTAIM charges for individual atoms.

**Table 1:** Key Quality Indicators for the Diffraction Experiments

compound	**4**	**5**	**6**	**7**	**8**	**9**
resolution sin θ/λ [Å^–1^]	1.20	1.20	1.20	1.11	1.10	1.10
R1 (IAM)	0.0168	0.0197	0.0141	0.0111	0.0086	0.0141
wR2 (IAM)	0.0337	0.0499	0.0309	0.0319	0.0241	0.0329
R1 (MM)	0.0112	0.0178	0.0112	0.0078	0.0061	0.0109
wR2 (MM)	0.0263	0.0288	0.0241	0.0186	0.0178	0.0215
Δρ max/min [eÅ^–3^]	0.651/–0.843	0.747/–0.697	0.656/–0.659	0.317/–0.279	0.347/–0.370	0.522/–0.457

### Experimental Charge Density of the Halogen-Bond Donor Molecules

**Figure 6:**
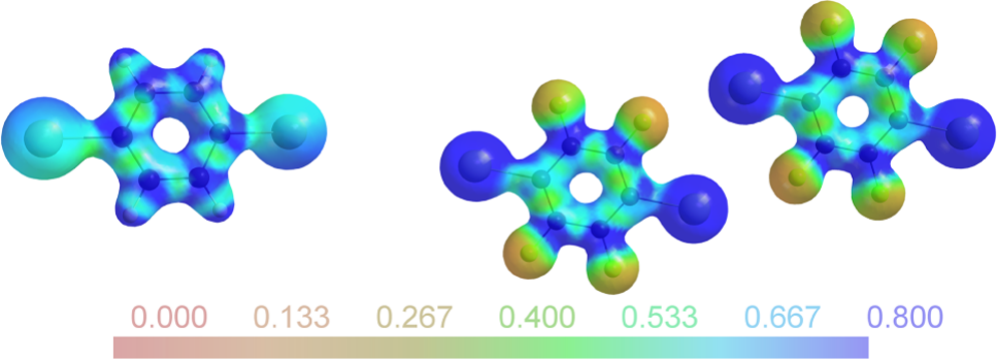
Experimentally derived electrostatic potential for **4** (left) and the two independent molecules in **6** (right) mapped on an electron density isosurface of 0.5 eÅ^–3^.^35^

We have been able to obtain high-resolution diffraction data for the XB donors **4**, **5**, and **6**; in the case of iodobenzene (**2**), a phase transition precludes the determination of the electron density. The topologically equivalent constituents diiodobenzene **4** and its perfluorinated counterpart **6** are well suited for a comparison of their electronic situation based on a QTAIM analysis. A very strong intramolecular effect of fluorine substitution on the iodine charges may be expected and is indeed experimentally observed (Table S7, Supporting Information). In the case of **4**, the heavy halogen represents the most electronegative atom and is intuitively associated with a negative charge. In contrast, the F substituents in **6** lead to a positive charge on iodine. This difference is visibly reflected in Figure 6, which compares the electrostatic potentials (ESPs) of **4** and **6**.

The same color scale for the ESP has been used for both parts of Figure 6. The iodine atoms in the perfluorinated compound **6** are associated with a much more positive ESP than in **4**; for the former, a stronger interaction and a shorter contact distance to halogen-bond acceptors can be expected.^36^ This idea is also corroborated by the large number of halogen-bonded co-crystals in which DITFB acts as a halogen donor.

### Experimental Charge Density of the Halogen-Bonded Aggregates

**Figure 7:**
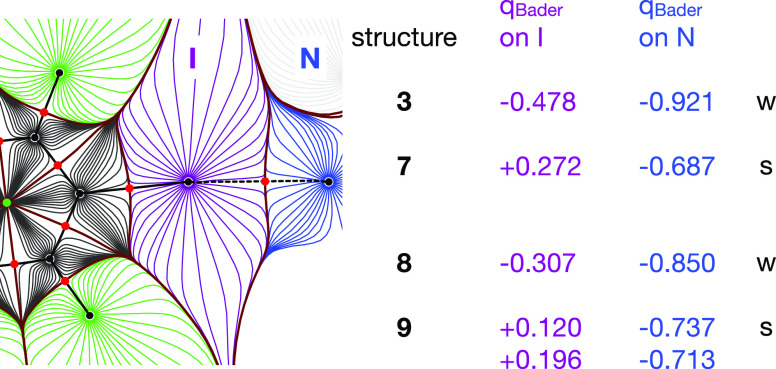
QTAIM charges (*q*_Bader_) obtained via integration of the atomic basins around I and around N for weak (w) and strong (s) halogen bonds. The gradient trajectory plot for **7** has been used as an example for the atomic basins.

We have shown above that fluorine substitution in **6** leads to significantly more positive iodine atoms. In turn, the more positive region at the halogen-bond donor results in stronger I···N contacts. Halogen bonds are associated with a strong electrostatic contribution, but charge transfer components should also not be neglected.^33^ We may expect a redistribution of the electron density from the region about the halogen-bond acceptor nitrogen to that of the halogen-bond donor iodine. Figure 7 summarizes our results in this context.

A strong effect of intramolecular fluorine substitution on the QTAIM charges at iodine is perceived and not surprising: the heavy halogen carries negative charges in the absence of fluorine neighbors at the aromatic ring. We note a second more subtle but consistent trend: The atomic charges at the halogen-bond acceptor N are less negative in the case of shorter contacts, thus indicating a higher degree of redistribution of electron density between the constituents. This effect on the halogen-bond acceptor via electronic modification of the XB donor has to the best of our knowledge not been observed previously and may prove relevant for concepts such as activation of a molecule by manipulating its neighbors in the crystalline state or, in the case of a sufficiently strong XB, in solution. Together with the trend in the Bader charges we observe a concomitant shrinkage of the Bader volumes; this information is compiled in Table S7, Supporting Information

**Figure 8:**
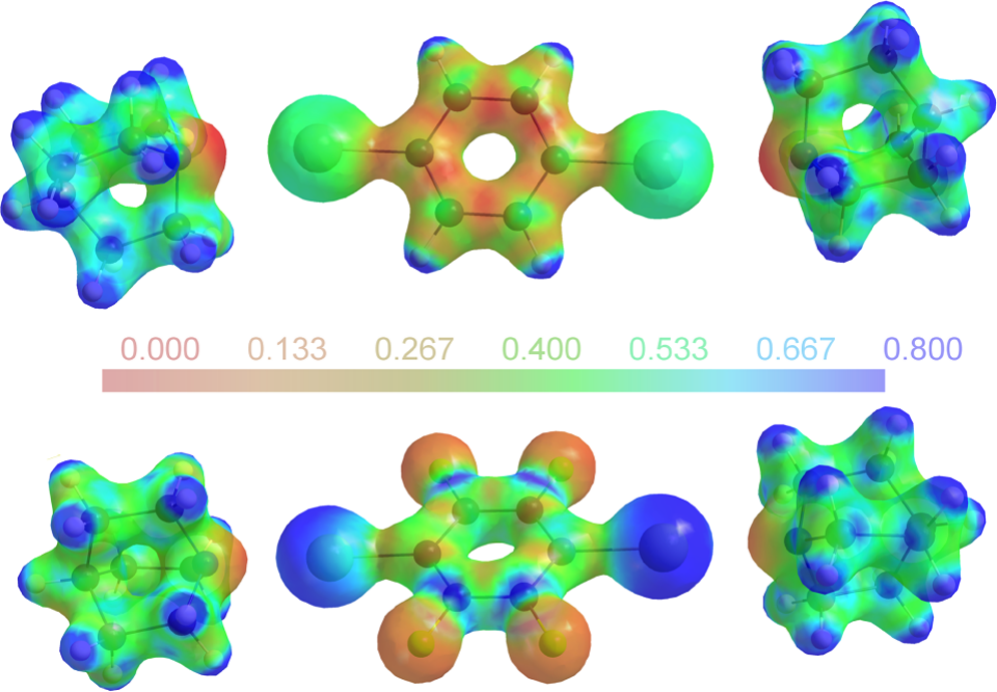
Experimentally derived ESP for **8** (top) and **9** (bottom) mapped on an electron density isosurface of 0.5 eÅ^–3^;^35^ for **9**, only one of two symmetrically independent trimolecular aggregates is shown.

In analogy to Figure 6, the DIB (**4**) and DITFB (**6**) XB donors can be compared in their halogen-bonded co-crystals **8** and **9**. Figure 8 shows the ESP for the topologically equivalent trimolecular aggregates.

**Figure 9:**
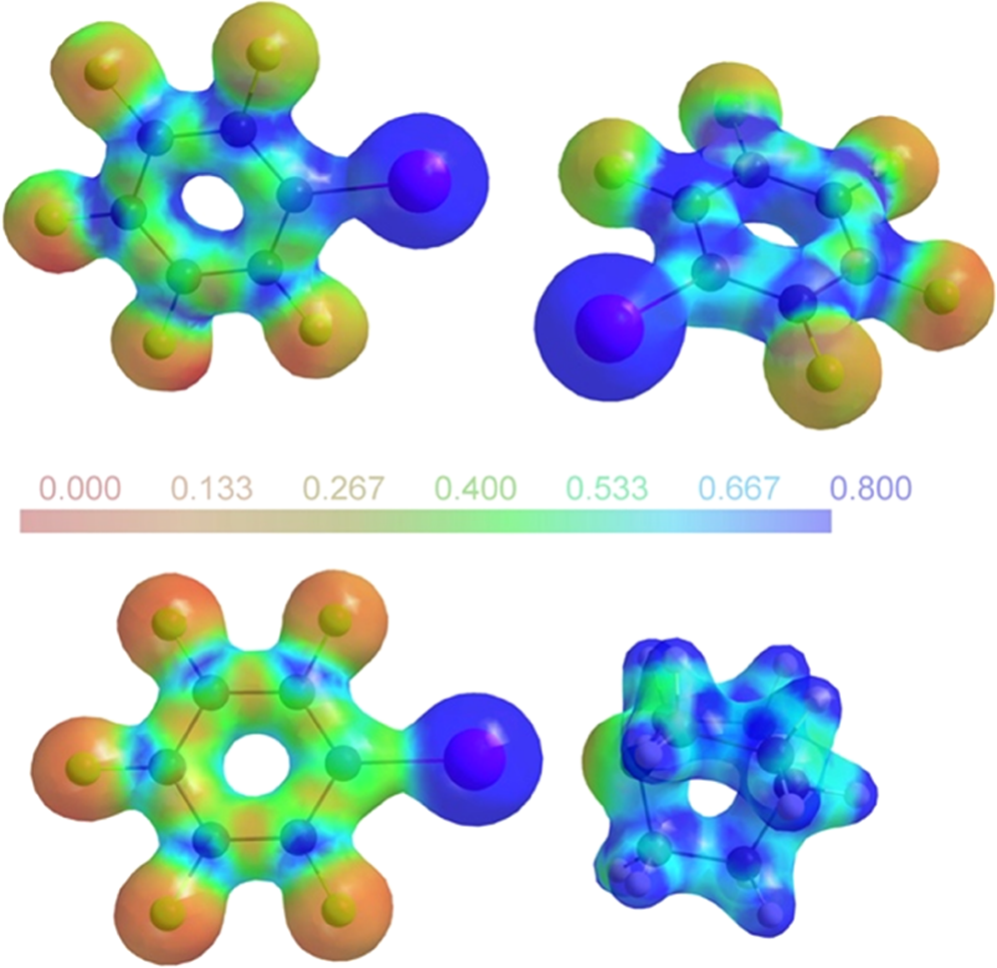
Experimentally derived ESP for **5** (top) and **7** (bottom) mapped on an electron density isosurface of 0.5 eÅ^–3^.^35^

Polarization of the nitrogen nucleophiles may be perceived in both **8** and **9**. The iodine XB donors are associated with a visibly more positive ESP in **9**. Figure 9 provides a visual comparison between the halogen-bond donor PFIB (**5**) and its quinuclidine aggregate **7**.

PFIB crystallizes with two independent molecules in the asymmetric unit, related by a single rather short F···I contact (3.0732(6) Å). The ESP for a halogen-bonded adduct in **7** (Figure 9, bottom) reflects the lateral symmetry expected for an isolated aggregate. Not only the halogen-bond distance but also the charge distribution in **7** is very similar to that observed in **9**. In contrast, the ESPs associated with the two independent molecules on PFIB (Figure 9, top) show clear discrepancies from lateral symmetry.

**Figure 10:**
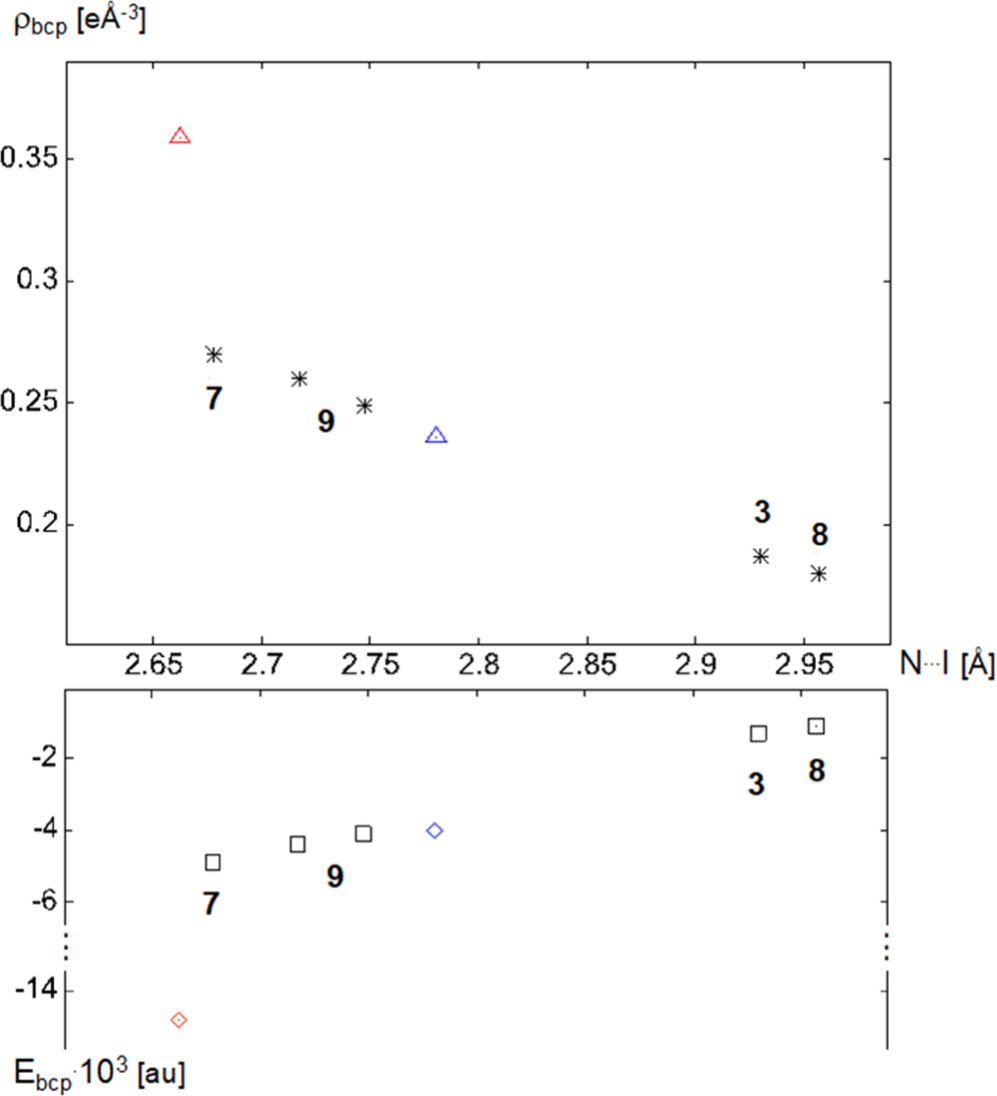
Top: Electron densities ρ_bcp_ in the N···I contacts; red and blue triangles refer to literature data.^20,37^ Bottom: total energy densities in the bcps for N···I contacts; red and blue diamonds refer to literature data.^20,37^

We now proceed to an analysis of the properties in the bond critical points (bcps) of the short and presumably strong (**7**, **9**) and the longer (**3**, **8**) halogen bonds between quinuclidine N and arene iodine. Figure 10 summarizes two QTAIM-derived quantities useful for gauging the halogen-bond strength, namely, the electron density ρ_bcp_ (top) and the total energy density *E* (bottom) in the bcps.

Figure 10 (top) confirms that the shorter N···I contacts in **7** and **9** are associated with higher electron density than those in **3** and **8**. In the absence of further charge density studies on halogen bonds between arene-I and quinuclidine N, two literature results have been included to put our results into a scientific context. The value marked with a blue triangle refers to the aggregate of (*E*)–1,2-bis(4-pyridyl)ethylene with DITFB (**6**);^37^ it fits the distance range of the data reported in this work. The red triangle denotes the very short halogen bond between DITFB and 4-(dimethylamino)pyridine,^20^ a powerful nucleophile. These two data points do not strictly qualify for a comparison: they do not involve quinuclidine as an XB acceptor and must therefore be viewed with caution. Energy densities in the ρ_bcp_ have proven useful to categorize short contacts.^38,39^Figure 10 (bottom) shows that the halogen bonds in **3** and **7**–**9** are associated with a negative total energy density *E*, obtained as the sum of the (positive) kinetic energy density *G* and the (negative) potential energy density *V*; details concerning the calculation of the energy densities are given in the Supporting Information. Data for the two non-quinuclidine aggregates mentioned above have been included in this graphical comparison as blue and red diamonds. The criteria of Figure 10 consistently place our halogen bonds into two groups: the longer contacts encountered in **3** and **8** are associated with smaller ρ_bcp_ and hardly negative total energy density *E* in the bcps. In contrast, the shorter contacts in **7** and **9** show higher ρ_bcp_ and significantly negative total energy.

In principle, the electron density offers an almost ideal meeting ground for experiment and theory. Strictly spoken, a complementary theoretical investigation of the weak and strong halogen bonds in **3**, **7**, **8**, and **9** must be based on calculations under periodic boundary conditions of the extended crystals but such calculations exceed our competence. As a modest first approach to address the different energy contributions to the halogen bonds, we recurred to the calculation of pairwise intermolecular interaction energies with CrystalExplorer;^40,41^ the results are shown in Figure S15 and Table S31. The total energy clearly distinguishes the strong halogen bonds in **7** and **9** from those in **3** and **8**. By coincidence, the sequence of the electrostatic contributions *E*_ele_ for halogen-bonded pairs turns out as *E*_ele_(**7**) < *E*_ele_(**9**) < *E*_ele_(**3**) ≈ *E*_ele_(**8**) and thus matches the energies in the bond critical point of the XBs shown in Figure 10b.

We wish to point out the excellent match between our experimental results and the recent calculations by Miller et al.^42^ These authors have studied the transition from intermolecular halogen contacts to covalent bonds with the help of QTAIM analysis and concluded that the van der Waals normalized distance^43^ between an XB donor D and an acceptor A, *R*_D···A_ = *d*_D···A_/(*r*_D_ + *r*_A_), can be used as a basic parameter to classify the interaction type within a continuous distance regime. They have identified a reduced distance of about 0.78 as an interesting value, stating that shorter XBs are associated with an overall negative energy density. The synopsis in Table 2 fully confirms these statements for our compounds: **7** and **9**, with reduced distances shorter than this limit, show significantly negative, **3** and **8** with their longer and weaker XBs only insignificant total energy densities.

**Table 2:** Synopsis of Halogen Bonds, Reduced Contact Distances, and Total Energy Densities in **3** and **7**–**9**^a^

compound	**3**	**7**	**8**	**9**	
distance *d*_N···I_ [Å]	2.9301(4)	2.6781(2)	2.9568(3)	2.7173(5)	2.7476(5)
*R*_N···I_	0.83	0.76	0.84	0.77	0.78
*E* [au]	–0.0013	–0.0049	–0.0011	–0.0044	–0.0041

a *R*_N···I_ is the reduced distance *d*_N···I_/(*r*_I_ + *r*_N_); *r*_I_ and *r*_N_ are the van der Waals radii^43^ for I and N, respectively; *E* is the total energy density [au]

### Vibrational Spectroscopy

In addition to the characterization of the halogen-bonded adducts **3**, **7**, **8**, and **9** in their crystal structures, Raman and NMR studies were performed. For this purpose, Raman spectra were recorded from all compounds **1** to **9**, and the respective spectra were also quantum chemically calculated to assign the bands to the respective vibrations. Good agreement was found between experiment and simple density functional theory (DFT) level theory, although the intensity of the bands of the XB donor differed slightly, probably due to the pseudopotential used for iodine. The usage of common solvents was deliberately avoided to improve the signal-to-noise ratio and to circumvent problems with the solubility of DIB. Thus, the solids **1**, **4**, and **6**, as well as the XB adducts **8** and **9** were measured as pellets in solid phase, whereas IB and PFIB were used as solvents for quinuclidine for the measurement of **2**, **3**, **5**, and **7**. Based on the redshift of the stretching vibration of the C–I bond (the symmetric one in the case of **4**, **6**, **8**, and **9**) in the XB donors (Table 3, shift C) and the blueshift of one of the bands of the wagging vibration of the CH_2_ adjacent to the nitrogen of quinuclidine (Table 3, shift A), the adduct formation could be identified. Moreover, the magnitude of the signal shift appears to be reasonably correlated with the strength of the interaction, as the redshift indicates a weakening of C–I bond, which should be more pronounced in the stronger XBs. Besides the expected observation of the strong interaction between DITFB and quinuclidine (in **9**), the weak interaction between DIB and quinuclidine (in **8**) was also observed in the solid state. This offers the possibility to identify weak halogen bonds even in the case of nondeterminable crystal structures. In solution, only the strong interaction between PFIB and quinuclidine (in **7**) could be detected. However, due to the strong excess of the XB donor in the measurements of the solutions for **3** and **7**, not all shifts were traceable.

**Table 3:** Experimental Vibrational Shifts Relative to the Respective Pure Compounds^a^

XB	shift A^b^ [cm^–1^]	shift B^b^ [cm^–1^]	shift C^c^ [cm^–1^]
**3**	X^d^	1	0
**7**	18	X^d^	–19
**8**	9	5	–5
**9**	17	–2	–15

a Shifts A and B are belonging to the XB acceptor quinuclidine, and shift C to the respective XB donor.

b Wagging vibrations of the CH_2_ adjacent to the nitrogen of quinuclidine.

c Stretching vibration of the C–I bond of the XB donors (the symmetric one in the case of **4**, **6**, **8**, and **9**).

d Nondetectable shift due to a strong solvent-related peak of the XB donor.

### NMR Spectroscopy in Solution

**Figure 11:**
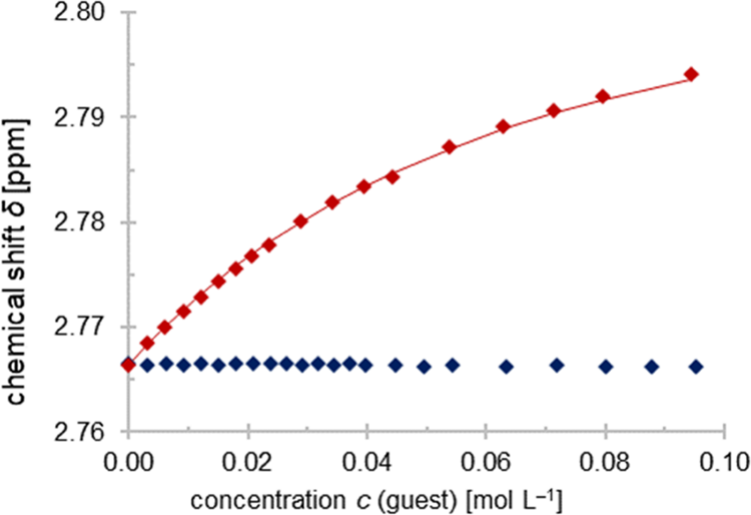
Chemical shift δ at room temperature of NCH_2_ of quinuclidine as host plotted against the guest concentration of the XB donors IB (blue) and PFIB (red). The red line represents the logarithmic fit of the PFIB data set.

In the NMR study, we started with a proof of principle and determined the strength of the halogen bond in **7**, which is already known in the literature via one-dimensional ^1^H NMR titration.^28^ For the ^1^H NMR titration with quinuclidine as host, the values for *K*_a_ = 20 ± 1 M^–1^ and Δ*G* = −7.4 ± 0.1 kJ/mol and with PFIB as host *K*_a_ = 20 ± 1 M^–1^ and Δ*G* = −7.5 ± 0.2 kJ/mol were obtained, respectively (Graphs S1 and S2, Supporting Information). Compared to the literature (*K*_a_ = 20 ± 4 M^–1^ und Δ*G* = −7.5 ± 0.4 kJ/mol),^44^ this confirms our results and evaluations. The weak halogen-bonded adduct **3** showed no evidence of a halogen bond at room temperature in solution (Figure 11).^29^

Similar to **3**, the halogen bond of **8** could not be detected due to the insufficient solubility of DIB in cyclohexane-*d*_12_, as already shown in the Raman studies.

When considering XB adduct **9**, the concentrations of DITFB used were halved in each case, with reference to the equivalent concentration of iodine. Here, *K*_a_ = 17.9 ± 0.9 M^–1^ and Δ*G* = −7.1 ± 0.1 kJ/mol were obtained for quinuclidine as host and *K*_a_ = 20 ± 1 M^–1^ and Δ*G* = −7.5 ± 0.1 kJ/mol were obtained for DITFB as host (Graphs S3 and S4, Supporting Information). The values show a similar interaction strength of **7** and **9**, but they do not match after the host is changed (Table 4). One reason for the deviation could be that the two halogen bonds were assumed to be two identical and independent interactions in the evaluation, which is not the case. In addition, reading the exact shift from the ^19^F NMR spectra proved to be extremely complicated because the initially shown singlet signal of the DITFB shifts differently due to the formation of one of the halogen bonds (ortho and meta fluorine substituents react differently to the formed halogen bond). Moreover, due to the formation of one of the interactions, two pairs of equivalent fluorine atoms (ortho and meta) are formed from four originally chemically equivalent fluorine atoms, thus leading to further splitting of the NMR signal and forming of an indefinable multiplet (e.g., Figure S86, Supporting Information). From this multiplet, integral values were used to estimate the center and thus determine the shift of the overall signal. Another possible source of error lies in the formation of the second halogen bond. In this step, the pairs of chemically equivalent fluorine atoms are combined to form four chemically equivalent fluorine atoms again. Since at this point each fluorine atom is both an ortho and a meta substituent with respect to the formed halogen bonds, the respective shifts could partially cancel each other out and thus be a source of further inaccuracy.

**Table 4:** Experimental Binding Energies of PFIB and DITFB with Quinuclidine

halogen bond (host^a^)	*K*_a_^b^ (M^–1^)	Δ*G*^c^ [kJ/mol]
7 [literature]^44^	20 ± 4	–7.5 ± 0.4
7 (quinuclidine)	20 ± 1	–7.4 ± 0.1
7 (PFIB)	20 ± 1	–7.5 ± 0.2
9 (quinuclidine)	17.9 ± 0.9	–7.1 ± 0.1
9 (DITFB)	20 ± 1	–7.5 ± 0.1

a Host regarding the NMR titration equals compound with constant concentration during the experiments.

b Association constant *K*_a_ in cyclohexane at 298 K, determined by curve-fitting of ^1^H and ^19^F NMR titration data, respectively.

c Free binding energy calculated from the association constant.

## Conclusions and Further Work

Scheiner and Hunter^25^ have recently been able to calculate the effect of different XB donors on the acceptor molecule ammonia; these authors find a good correlation between the NMR shielding of the ammonia nitrogen atom and the XB strength. In this study, we have been able to experimentally verify such an effect for the first time: our experimental comparison of XBs subtended by the acceptor molecule quinuclidine and electronically different iodoarene donors has shed light on the “dark side” of the electronic situation at the XB acceptor. We have presented the first experimental proof that F substitution in the XB donor has direct effects not only on the short contact itself but also on the electronic properties of the XB acceptor. Density-derived quantities such as Bader charges and atomic volumes at the acceptor N site confirm this intermolecular correlation. Consequently, tuning of electronic properties and possibly also of reactivity via remote substitution in the XB donor partner becomes feasible. In addition to this hitherto unexplored aspect, the expected straightforward effects of fluorine substitution at the XB donor on the short contact are observed. Even conventional structure models show that the increasing σ hole at the I donor leads to shorter XBs. Multiple advanced experiments confirm that these shorter contacts correspond to stronger interactions. In solution, NMR titrations provide an estimate of 7 kJ/mol for the interaction energy of the presumably stronger XB systems and confirm their formation. In condensed phase, a redshift in the symmetric C–I stretching vibration reflects the interaction energy in the halogen-bonded adducts and was assessed by Raman spectroscopy. In the solid, high-resolution X-ray diffraction allows us to assess the electron density and its derived properties. Among these, the total energy density in the bond critical point ranks the shorter contacts as stronger, and the electrostatic potential in the XB system matches predictions from theory. The results above were obtained for XBs involving the same acceptor molecule, quinuclidine. In future work, we will vary the acceptor molecule and probe its role for the halogen bond, in particular with respect to hybridization at the XB acceptor site.

## Supporting Information Available

Material and methods; synthetic chemistry and compound characterization; IAM and MM refinement; NMR spectra; RAMAN spectra; and quantum chemical calculations (PDF)

## Dedication

^†^ Dedicated to Professor Klaus Jurkschat on the occasion of his 70th birthday.
