# Supplementary material for: Gauging the Strength of the Molecular Halogen Bond via Experimental Electron Density and Spectroscopy†
Source: ACS Omega. 2023 Jun 5;8(24):21531–9. doi: 10.1021/acsomega.3c00619 (PMC10286298; doi:10.1021/acsomega.3c00619)
Supplement: Supplementary file 1 — ao3c00619_si_001.pdf [file ao3c00619_si_001.pdf]

Supporting Information
ACS Omega

Gauging the Strength of the Molecular Halogen Bond *via* Experimental Electron Density and Spectroscopy

Felix Otte[a], Johannes Kleinheider[a], Bastian Grabe[b], Wolf Hiller[b], Franziska Busse[c], Ruimin Wang[c,d], Nora M. Kreienborg[e], Christian Merten[e]*, Ulli Englert[c,d]* and Carsten Strohmann[a]*

[a] TU Dortmund University
Inorganic Chemistry
Otto-Hahn-Str. 6, 44227 Dortmund, Germany
[b] TU Dortmund University
Faculty of Chemistry and Chemical Biology
Otto-Hahn-Str. 4a, 44227 Dortmund, Germany
[c] RWTH Aachen University
Inorganic Chemistry
Landoltweg 1, 52056 Aachen, Germany
[d] Shanxi University
Institute of Molecular Science
Wucheng Road 92, 030006 Taiyuan, P. R. China
[e] Ruhr University Bochum
Organic Chemistry II
Universitätstraße 150, 44801 Bochum, Germany

Abstract: Strong and weak halogen bonds (XBs) in discrete aggregates involving the same acceptor are addressed by experiments in solution and in the solid state. Unsubstituted and perfluorinated iodobenzenes act as halogen donors of tunable strength; in all cases, quinuclidine represents the acceptor. NMR titrations reliably identify the strong intermolecular interactions in solution, with experimental binding energies of approx. 7 kJ mol^{-1}. Interaction of the σ hole at the halogen donor iodine leads to a red shift in the symmetric C–I stretching vibration; this shift reflects the interaction energy in the halogen-bonded adducts and may be assessed by Raman spectroscopy in condensed phase even for weak XBs. An experimental picture of the electronic density for the XBs is achieved by high resolution X-ray diffraction on suitable crystals. QTAIM analysis affords the electron densities and energy densities in the bond-critical points of the halogen bonds and confirms stronger interaction for the shorter contacts. For the first time, the experimental electron density shows a significant effect on the atomic volumes and Bader charges of the quinuclidine N atoms, the halogen bond acceptor: strong and weak XBs are reflected in the nature of their acceptor atom. Our experimental findings at the acceptor atom match the discussed effects of halogen bonding and thus the proposed concepts in XB activated organocatalysis.

DOI: X

Table of Contents

1	**General Remarks**	**S2**
2	**Experimental Procedures**	**S3**
3	**Single-Crystal X-ray Diffraction Analysis**	**S5**
4	**Simplified analysis of pairwise interactions in the crystal**	**S28**
5	**Quantum Chemical Calculations**	**S29**
6	**NMR Investigations in Solution**	**S35**
7	**Raman spectroscopic data**	**S84**
8	**References**	**S92**

1 General Remarks

All reactions with oxygen- and moisture-sensitive compounds were performed under an atmosphere of argon in dried solvents, which were distilled prior to use. All other solvents and commercially available reagents, including the NMR solvents, were purified by a kugelrohr distillation (Glass Oven B-585 from *Büchi*) and stored under an atmosphere of argon. The NMR solvent benzene-d6 was also stored over sodium wires.

The NMR spectra were measured on a 500 MHz *Bruker Avance NEO*, 600 MHz *Bruker Avance III HD* and 500 MHz *Agilent Technologies DD2* spectrometer at 25 °C. Chemical shifts (δ in ppm) are referred to tetramethylsilane (TMS), with the deuterium signal of the solvent serving as internal lock. ^{1}H, ^{13}C, ^{15}N HMBC and 1D NOESY spectra were recorded.

Data collections for the compounds **2** and **3** were conducted on a *Bruker D8 Venture* four-circle diffractometer by *Bruker AXS GmbH* using a *PHOTON II* CPAD detector by *Bruker AXS GmbH*. X-ray radiation was generated by a microfocus source *IµS* Mo by *Incoatec GmbH* with *HELIOS* mirror optics and a single-hole collimator by *Bruker AXS GmbH*.

For the data collection, the programs *APEX 3 Suite* (v.2019.1-0) with the integrated programs *SAINT* (integration) and *SADABS* (absorption correction) by *Bruker AXS GmbH* were used. Using *Olex*^{2} [1], the structures were solved with the *ShelXT* [2] structure solution program by Intrinsic Phasing and refined with the *XL* [3] refinement package using Least Squares minimization.

For the selection of air and moisture sensitive crystals the *X-TEMP 2* [4] system was used in combination with a *SMZ1270* stereomicroscope from *Nikon Metrology GmbH*. *MicroGrippers* from *MiTeGen* were used for mounting.

The Raman spectra were recorded on a Bruker IFS 66 IR spectrometer with a FRA 106 Raman module. Samples were measured in a pressed pallet prepared from pure compounds **1**, **4**, **6** and the corresponding crystallized halogen bond complexes **8**, **9**. The liquid compounds **2**, **5** were measured in pure phase and the corresponding halogen bond complexes **3**, **7** were measured as solutions of **1** in **2**, **5**, respectively. All Raman spectra were recorded with a resolution of 2 cm^{-1} and accumulated for ~1000 scans (measurement time 1h).

2 Experimental Procedures

2.1 Synthesis of the pentafluoroiodobenzene·quinuclidine adduct (7)

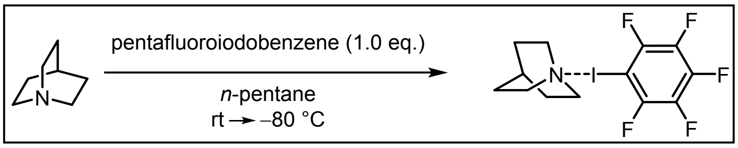

First a SCHLENK flask was purged of air and moisture by alternating application of vacuum in combination with heating and inert gas. Then a mixture of quinuclidine (111 mg, 1.0 mmol, 1.0 eq.) and pentafluoroiodobenzene (0.13 mL, 1.0 mmol, 1.0 eq.) was combined in 0.5 mL *n*-pentane under argon atmosphere. The mixture was stored for two days at –30 °C and then at –80 °C. After a few days suitable crystals for X-ray structural analysis were obtained.

2.2 Crystallization of pentafluoroiodobenzene (5)

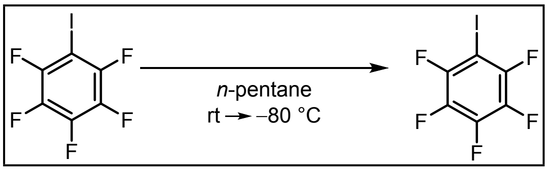

First a SCHLENK flask was purged of air and moisture by alternating application of vacuum in combination with heating and inert gas. Then pentafluoroiodobenzene (0.26 mL, 2.0 mmol, 1.0 eq.) was added in 0.5 mL *n*-pentane under argon atmosphere. The mixture was stored for one day at –30 °C and then at –80 °C. After one day suitable crystals for X-ray structural analysis were obtained.

2.3 Synthesis of the diiodobenzene·quinuclidine adduct (8)

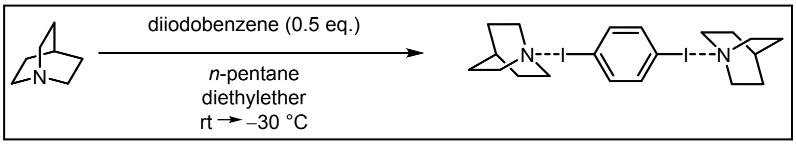

First a SCHLENK flask was purged of air and moisture by alternating application of vacuum in combination with heating and inert gas. Then a mixture of quinuclidine (111 mg, 1.0 mmol, 1.0 eq.) and diiodobenzene (160 mg, 0.5 mmol, 0.5 eq.) was combined in a mixture of 2.0 mL *n*-pentane and 1.0 mL diethylether under argon atmosphere. The mixture was stored for two days at –30 °C. After a few days suitable crystals for X-ray structural analysis were obtained.

2.4 Crystallization of diiodobenzene (4)

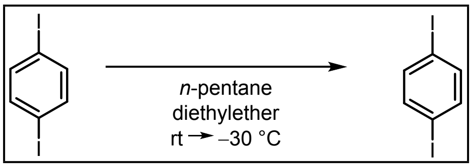

First a SCHLENK flask was purged of air and moisture by alternating application of vacuum in combination with heating and inert gas. Then diiodobenzene (160 mg, 0.5 mmol, 1.0 eq.) was added a mixture of 2.0 mL *n*-pentane and 1.0 mL diethylether under argon atmosphere. The mixture was stored for one day at –30 °C. After one day suitable crystals for X-ray structural analysis were obtained.

2.5 Synthesis of the 1,4-diiodotetrafluorobenzene·quinuclidine adduct (9)

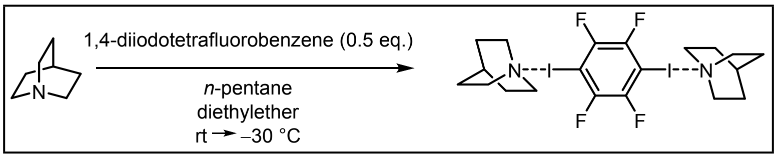

First a SCHLENK flask was purged of air and moisture by alternating application of vacuum in combination with heating and inert gas. Then a mixture of quinuclidine (111 mg, 1.0 mmol, 1.0 eq.) and 1,4-diiodotetrafluorobenzene (200 mg, 0.5 mmol, 0.5 eq.) was combined in a mixture of 2.0 mL *n*-pentane and 1.0 mL diethylether under argon atmosphere. The mixture was stored for two days at –30 °C. After a few days suitable crystals for X-ray structural analysis were obtained.

2.6 Crystallization of 1,4-diiodotetrafluorobenzene (6)

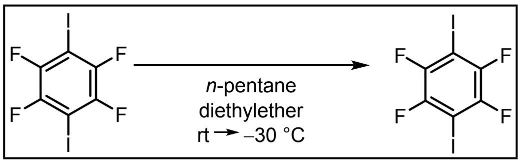

First a SCHLENK flask was purged of air and moisture by alternating application of vacuum in combination with heating and inert gas. Then 1,4-diiodotetrafluorobenzene (200 mg, 0.5 mmol, 1.0 eq.) was added a mixture of 2.0 mL *n*-pentane and 1.0 mL diethylether under argon atmosphere. The mixture was stored for one day at –30 °C. After one day suitable crystals for X-ray structural analysis were obtained.

2.7 Overview of structures containing iodobenzene and 1,4-diiodobenzene deposited in the CSD

Searches were performed with version 5.42, including updates until May 2021, and limited to error-free structures without disorder based on diffraction data collected at T ≤ 150 K. Database entries in the CSD [5] are given as refcodes, followed by the C–I distances.

Iodobenzene (**2**)
Individual entries: COYSER 2.110 Å, FOSNOR 2.115 Å, FOSNUX 2.103 Å, UFAQUO 2.090 Å, INAJEP 2.109 Å, INAJEP01 2.111 Å, REKYAI01 2.102 and 2.104 Å, REKYAI02 2.099 Å.
Database average: 2.105 Å

1,4-Diiodobenzene (**4**)
Individual entries: TALPAB 2 x 2.097 Å, PILKIG 2.089 and 2.094 Å, PILLIH 2 x 2.095 Å, PILLON 2 x 2.098 Å, PILMAA 2 x 2.085 Å, YESZEB 2 x 2.113, ZANQIR 2 x 2.112 Å.
Database average: 2.099 Å

3 Single-Crystal X-ray Diffraction Analysis

3.1 Individual Structures

The diffraction pattern of the XB adduct **7** could be measured at 100 K. The crystallographic data and results of the structure refinements are listed below.

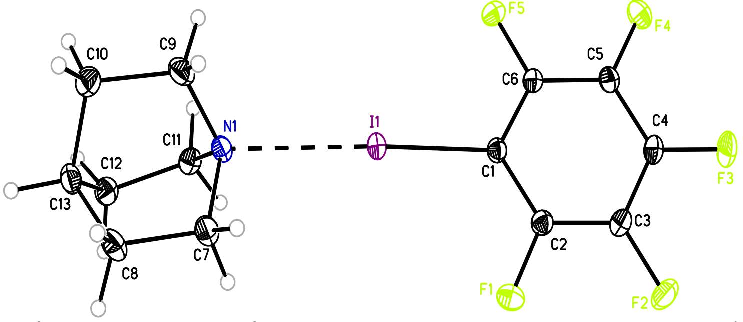

Figure S1: Displacement ellipsoid plot of the molecular structure of the XB adduct **7** in the crystal, with ellipsoids drawn at 50% probability. Numbering of hydrogen atoms omitted for clarity. Selected interatomic distances [Å] and angles [°] at IAM level: C1–I1 2.1297(3), I1–N1 2.6777(4), C1–I1···N1 177.33(2). CCDC numbers 2212579 (IAM) and 2212580 (MM).

Table S1: Crystallographic data and structural refinements of the XB adduct **7** (IAM and MM).

	**XB adduct 7**
Empirical formula	$C_{13}H_{13}F_5IN$
Formula weight [g·mol^{-1}]	405.14
Temperature [K]	100(2)
Crystal system	triclinic
Space group	$P\bar{1}$
Lattice parameters [Å]	6.3312(3)
	10.1433(5)
	11.7305(6)
α [°]	75.2959(9)
β [°]	87.2438(9)
γ [°]	71.7974(9)
Cell volume [Å^3]	691.79(6)
Z	2
Calculated density ρ [g·cm^3]	1.945
Absorption coefficient μ [mm^{-1}]	2.360
F(000)	392.0
Crystal size [mm^3]	0.237 × 0.171 × 0.099
Radiation	MoKα (λ = 0.71073)
Data collection 2Θ [°]	4.368 to 103.998
Index ranges	–14 ≤ h ≤ 14<br>–22 ≤ k ≤ 22<br>–26 ≤ l ≤ 26
Reflections collected	604345
Independent reflections	15783 [R_{int} = 0.0405, R_{sigma} = 0.0074]
**Refinement results for the IAM**	
Data / Restraints / Parameters	15783/0/182
Goodness-of-fit of F^2	1.171
Final R indexes [I≥2σ (I)]	R1 = 0.0111, wR2 = 0.0315
Final R indexes (all data)	R1 = 0.0123, wR2 = 0.0319
Largest diff. peak/hole [e·Å^{-3}]	0.62/–0.69
**Refinement results for the MM**	
Data / Parameters	15219/543
Goodness-of-fit of F^2	1.075
Final R index [I≥2σ (I)]	R_1 = 0.0078
Final R indexes (all data)	R_1 = 0.0086, wR_2 = 0.0186
Largest diff. peak/hole [e·Å^{-3}]	0.32/–0.28

The diffraction pattern of the XB donor **5** could be measured at 100 K. The crystallographic data and results of the structure refinements are listed below.

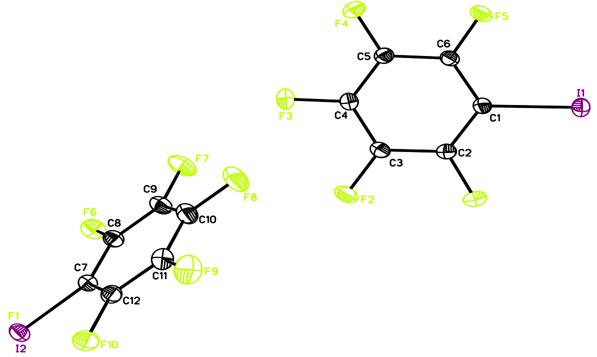

Figure S2: Displacement ellipsoid plot of the molecular structure of the XB donor **5** in the crystal, with ellipsoids drawn at 50% probability. Numbering of hydrogen atoms omitted for clarity. Selected interatomic distances [Å] at IAM level: C1–I1 2.0718(7), C7–I2 2.0755(6). CCDC numbers: 2212575 (IAM) and 2212576 (MM).

Table S2: Crystallographic data and structural refinements of the XB donor **5** (IAM and MM)

	**XB donor 5**
Empirical formula	C_6F_5I
Formula weight [g·mol^{-1}]	293.96
Temperature [K]	100(2)
Crystal system	monoclinic
Space group	$P2_1/c$
Lattice parameters [Å]	15.5311(7)
	10.7005(5)
	8.8320(4)
β [°]	94.7657(11)
Cell volume [Å^3]	1462.72(12)
Z	8
Calculated density ρ [g·cm^3]	2.670
Absorption coefficient μ [mm^{-1}]	4.406
F(000)	1072.0
Crystal size [mm^3]	0.230 × 0.194 × 0.074
Radiation	MoKα (λ = 0.71073)
Data collection 2Θ [°]	4.628 to 116.998
Index ranges	$-37 \leq h \leq 37$<br>$-25 \leq k \leq 24$<br>$-21 \leq l \leq 20$
Reflections collected	972328
Independent reflections	21163 [R_{int} = 0.0399, R_{sigma} = 0.0073]
**Refinement results for the IAM**	
Data / Restraints / Parameters	21163/0/218
Goodness-of-fit of F^2	1.043
Final R indexes [I≥2σ (I)]	R1 = 0.0197, wR2 = 0.0473
Final R indexes (all data)	R1 = 0.0263, wR2 = 0.0505
Largest diff. peak/hole [e·Å^{-3}]	1.34/–1.22
**Refinement results for the MM**	
Data / Parameters	20245 / 721
Goodness-of-fit of F^2	1.020
Final R index [I≥2σ (I)]	R_1 = 0.0178
Final R indexes (all data)	R_1 = 0.0197, wR_2 = 0.0288
Largest diff. peak/hole [e·Å^{-3}]	0.75/–0.70

The diffraction pattern of the XB adduct **8** could be measured at 100 K. The crystallographic data and results of the structure refinement are listed below.

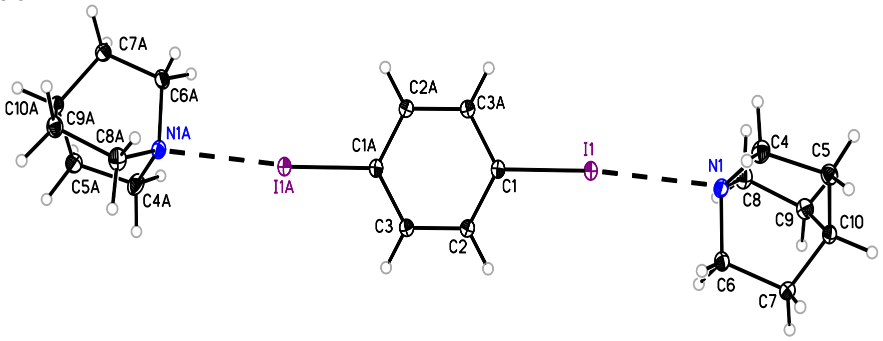

Figure S3: Displacement ellipsoid plot of the molecular structure of the XB adduct **8** in the crystal, with ellipsoids drawn at 50% probability. Numbering of hydrogen atoms omitted for clarity. Selected interatomic distances [Å] and angles [°]:C1–I1 2.1098(3), I1–N1 2.9562(3), C1–I1···N1 173.700(10). CCDC numbers: 2212581 (IAM) and 2212582 (MM).

Table S3: Crystallographic data and structural refinements of the XB adduct **8** (IAM and MM).

	**XB adduct 8**
Empirical formula	$C_{20}H_{30}I_2N_2$
Formula weight [g·mol^{-1}]	552.26
Temperature [K]	100.0
Crystal system	monoclinic
Space group	$P\bar{1}$
Lattice parameters [Å]	a = 5.9760(2)
	b = 6.3011(3)
	c = 13.5664(6)
α [°]	85.8230(10)
β [°]	87.9940(10)
γ [°]	84.7670(10)
Cell volume [Å^3]	507.16(4)
Z	1
Calculated density ρ [g·cm^3]	1.808
Absorption coefficient μ [mm^{-1}]	3.105
F(000)	270.0
Crystal size [mm^3]	0.230 × 0.147 × 0.117
Radiation	MoKα (λ = 0.71073)
Data collection 2Θ [°]	6.02 to 103.00
Index ranges	–13 ≤ h ≤ 13, –13 ≤ k ≤ 13, –29 ≤ l ≤ 29
Reflections collected	617953
Independent reflections	11337 [R_{int} = 0.0338, R_{sigma} = 0.0055]
**Refinement results for the IAM**	
Data / Restraints /Parameters	11337/0/110
Goodness-of-fit of F^2	1.211
Final R indexes [I≥2σ (I)]	R_1 = 0.0086, wR_2 = 0.0240
Final R indexes (all data)	R_1 = 0.0090, wR_2 = 0.0241
Largest diff. peak/hole [e·Å^{-3}]	0.64/–0.62
**Refinement results for the MM**	
Data / Parameters	11245/355
Goodness-of-fit of F^2	1.031
Final R index [I≥2σ (I)]	R_1 = 0.0061
Final R indexes (all data)	R_1 = 0.0120, wR_2 = 0.0178
Largest diff. peak/hole [e·Å^{-3}]	0.35/–0.37

The diffraction pattern of the XB donor **4** could be measured at 100 K. The crystallographic data and results of the structure refinement are listed below.

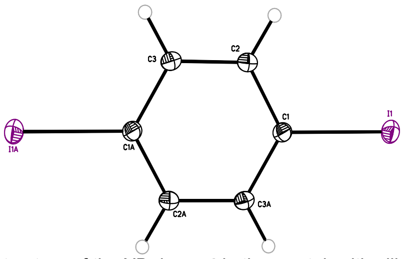

Figure S4: Displacement ellipsoid plot of the molecular structure of the XB donor **4** in the crystal, with ellipsoids drawn at 50% probability. Numbering of hydrogen atoms omitted for clarity. Selected interatomic distance [Å] at IAM level: C1–I1 2.0959(7). CCDC numbers: 2212573 (IAM) and 2212574 (MM).

Table S4: Crystallographic data and structural refinements of the XB donor **4** (IAM and MM).

	**XB donor 4**
Empirical formula	$C_6H_4I_2$
Formula weight [g·mol^{-1}]	329.89
Temperature [K]	100(2)
Crystal system	orthorhombic
Space group	*Pbca*
Lattice parameters [Å]	7.1675(5)
	6.0945(4)
	16.8665(11)
Cell volume [Å^3]	736.77(9)
Z	4
Calculated density ρ [g·cm^3]	2.974
Absorption coefficient μ [mm^{-1}]	8.432
F(000)	584.0
Crystal size [mm^3]	0.216 × 0.199 × 0.158
Radiation	MoKα (λ = 0.71073)
Data collection 2Θ [°]	4.83 to 116.994
Index ranges	–17 ≤ h ≤ 17<br>–14 ≤ k ≤ 14<br>–40 ≤ l ≤ 40
Reflections collected	386085
Independent reflections	5327 [R_{int} = 0.0439, R_{sigma} = 0.0062]
**Refinement results for the IAM**	
Data / Restraints /Parameters	5327/0/38
Goodness-of-fit of F^2	1.249
Final R indexes [I≥2σ (I)]	R_1 = 0.0168, wR_2 = 0.0330
Final R indexes (all data)	R_1 = 0.0184, wR_2 = 0.0336
Largest diff. peak/hole [e·Å^{-3}]	1.49/–1.19
**Refinement results for the MM**	
Data / Parameters	5184/145
Goodness-of-fit of F^2	1.009
Final R index [I≥2σ (I)]	R_1 = 0.0112
Final R indexes (all data)	R_1 = 0.0132, wR_2 = 0.0263
Largest diff. peak/hole [e·Å^{-3}]	0.65/–0.84

The diffraction pattern of the XB adduct **9** could be measured at 100 K. The crystallographic data and results of the structure refinement are listed below.

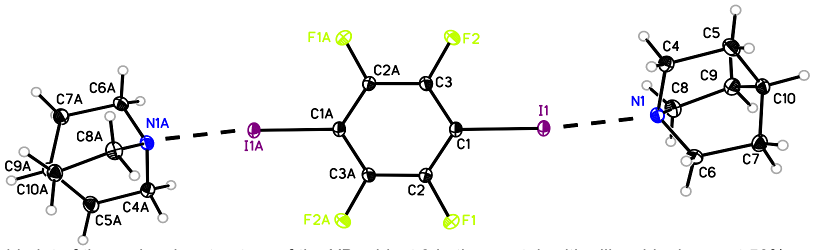

Figure S5: Displacement ellipsoid plot of the molecular structure of the XB adduct **9** in the crystal, with ellipsoids drawn at 50% probability. The second independent molecule of the asymmetric unit and the numbering of hydrogen atoms are omitted for clarity. Selected interatomic distances [Å] and angles [°] at IAM level: C1–I1 2.1153(6), C4–I2 2.1190(3), I1–N1 2.7155(5), I2–N2 2.7466(5), C1–I1···N1 173.83(2) C4–I2···N2 177.91(1). CCDC numbers: 2212584 (IAM) and 2212583 (MM).

Table S5: Crystallographic data and structural refinements of the XB adduct **9** (IAM and MM).

	**XB adduct 9**
Empirical formula	$C_{40}H_{52}F_8I_4N_4$
Formula weight [g·mol^{-1}]	1248.45
Temperature [K]	100.0
Crystal system	triclinic
Space group	$P\bar{1}$
Lattice parameters [Å]	a = 6.2088(4)
	b = 9.7255(7)
	c = 18.1940(16)
α [°]	82.183(3)
β [°]	89.300(3)
γ [°]	79.614(4)
Cell volume [Å^3]	1070.49(14)
Z	1
Calculated density ρ [g·cm^3]	1.937
Absorption coefficient μ [mm^{-1}]	2.980
F(000)	604.0
Crystal size [mm^3]	0.142 × 0.120 × 0.119
Radiation	MoKα (λ = 0.71073)
Data collection 2Θ [°]	4.298 to 102.998
Index ranges	–13 ≤ h ≤ 13, –21 ≤ k ≤ 21, –40 ≤ l ≤ 40
Reflections collected	1039029
Independent reflections	23973 [R_{int} = 0.0547, R_{sigma} = 0.0096]
**Refinement results for the IAM**	
Data / Restraints / Parameters	23973/0/253
Goodness-of-fit of F^2	1.106
Final R indexes [I≥2σ (I)]	R_1 = 0.0141, wR_2 = 0.0320
Final R indexes (all data)	R_1 = 0.0173, wR_2 = 0.0330
Largest diff. peak/hole [e·Å^{-3}]	0.59/–1.46
**Refinement results for the MM**	
Data / Parameters	23200/591
Goodness-of-fit of F^2	1.011
Final R index [I≥2σ (I)]	R_1 = 0.0109
Final R indexes (all data)	R_1 = 0.0122, wR_2 = 0.0215
Largest diff. peak/hole [e·Å^{-3}]	0.52/–0.46

The diffraction pattern of the XB donor **6** could be measured at 100 K. The crystallographic data and results of the structure refinement are listed below.

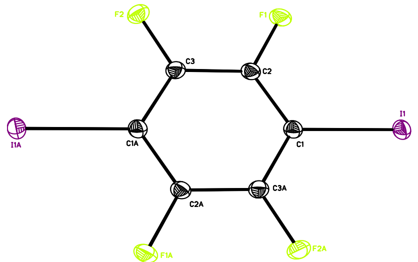

Figure S6: Displacement ellipsoid plot of the molecular structure of the XB donor **6** in the crystal, with ellipsoids drawn at 50% probability. Numbering of hydrogen atoms omitted for clarity. Selected interatomic distance [Å] at IAM level: C1–I1 2.0737(6). CCDC numbers: 2212577 (IAM) and 2212578 (MM).

Table S6: Crystallographic data and structural refinements of the XB donor **6** (IAM and MM).

	**XB donor 6**
Empirical formula	$C_6F_4I_2$
Formula weight [g·mol⁻¹]	401.86
Temperature [K]	100.0
Crystal system	monoclinic
Space group	$P2_1/c$
Lattice parameters [Å]	a = 6.1920(2)
	b = 11.5826(4)
	c = 5.7257(2)
β [°]	94.0660(6)
Cell volume [Å³]	409.61(2)
Z	2
Calculated density ρ [g·cm³]	3.258
Absorption coefficient μ [mm⁻¹]	7.683
F(000)	356.0
Crystal size [mm³]	0.126 × 0.080 × 0.050
Radiation	MoKα (λ = 0.71073)
Data collection 2Θ [°]	6.596 to 116.998
Index ranges	–14 ≤ h ≤ 14, –27 ≤ k ≤ 27, –13 ≤ l ≤ 13
Reflections collected	267331
Independent reflections	5919 [R_{int} = 0.0370, R_{sigma} = 0.0070]
**Refinement results for the IAM**	
Data / Restraints /Parameters	5919/0/55
Goodness-of-fit of F²	1.107
Final R indexes [I≥2σ (I)]	R_1 = 0.0142, wR_2 = 0.0390
Final R indexes (all data)	R_1 = 0.0165, wR_2 = 0.0401
Largest diff. peak/hole [e·Å⁻³]	1.48/–1.09
**Refinement results for the MM**	
Data / Parameters	5824/211
Goodness-of-fit of F²	0.963
Final R index [I≥2σ (I)]	R_1 = 0.0112
Final R indexes (all data)	R_1 = 0.0123, wR_2 = 0.0241
Largest diff. peak/hole [e·Å⁻³]	0.66/–0.66

Table S7: AIM (Bader) Charge (e) and Volume (Å³). [6]

Atom	Structure	**3**	**7**	**5**	**8**	**4**	**9**	**6**
I	Q [e]	–0.478	+0.272	+0.332; +0.380	–0.307	–0.280	+0.120; +0.196	+0.206
	V [Å³]	48.58	43.63	44.06; 42.64	49.79	48.15	55.46; 45.12	52.79
N	Q [e]	–0.921	–0.687		–0.850		–0.737; –0.713	
	V [Å³]	10.11	9.35		10.05		9.35; 8.96	

3.2 Statistics of the electron density studies

Results for the electron density study of XB adduct **7** are given below.

Table S8: R-value statistics as a function of resolution (in resolution shells) of XB adduct **7**.

θ	sin(θ)/λ	#	R1	wR2	S	Rs	av(I/SigW)	av(I)	av(SigW)
12.38	0.302	315	0.013	0.056	3.145	0.005	55.26	3423.57	57.29
15.68	0.380	321	0.012	0.047	2.608	0.004	53.80	2026.92	33.64
18.02	0.435	319	0.011	0.040	2.168	0.004	52.72	1457.74	24.63
19.90	0.479	311	0.011	0.039	2.064	0.004	50.60	1150.00	19.74
21.51	0.516	322	0.011	0.035	1.827	0.005	49.49	987.66	17.20
22.94	0.548	304	0.011	0.033	1.680	0.005	48.68	810.95	14.38
24.22	0.577	348	0.010	0.027	1.333	0.006	45.85	626.16	11.44
25.40	0.603	316	0.009	0.023	1.110	0.006	45.14	600.87	11.12
26.49	0.628	292	0.008	0.022	1.008	0.007	43.62	509.13	9.69
27.52	0.65	331	0.009	0.022	0.995	0.007	42.27	456.10	8.83
28.49	0.671	333	0.009	0.022	0.985	0.008	40.59	397.16	7.91
29.41	0.691	300	0.009	0.022	0.961	0.008	39.83	356.64	7.27
30.28	0.709	328	0.009	0.022	0.931	0.009	39.34	326.67	6.88
31.12	0.727	308	0.009	0.022	0.889	0.010	37.19	301.92	6.50
31.93	0.744	333	0.010	0.022	0.859	0.012	35.74	253.53	5.79
32.71	0.76	330	0.009	0.020	0.814	0.011	37.36	284.44	6.39
33.46	0.776	294	0.010	0.023	0.848	0.013	33.48	210.16	5.12
34.20	0.791	311	0.011	0.023	0.830	0.014	33.04	204.80	5.11
34.91	0.805	325	0.013	0.025	0.885	0.015	31.42	173.90	4.53
52.00	1.109	9742	0.012	0.030	0.870	0.016	25.56	81.30	2.39

Table S9: Resolution and completeness statistics (cumulative and Friedel pairs averaged) of XB adduct **7**.

θ	sin(θ)/λ	Completeness [%]	Expected	Measured	Missing
20.82	0.500	99.9	1452	1451	1
23.01	0.550	99.9	1912	1911	1
25.24	0.600	100	2501	2500	1
27.51	0.650	100	3180	3179	1
29.84	0.700	100	3975	3974	1
32.21	0.750	100	4904	4903	1
34.65	0.800	100	5939	5938	1
37.17	0.850	100	7113	7112	1
39.77	0.900	100	8474	8473	1
42.47	0.950	100	9932	9931	1
45.29	1.000	100	11599	11598	1
48.27	1.050	100	13437	13436	1
51.43	1.100	100	15414	15413	1
52.00	1.109	100	15784	15783	1

$R_{sigma} = \sum\sigma_{Int} / \sum Int =$ 0.0074

Table S10: Missing reflections of XB adduct **7**.

Nr.	H	K	L	sin(θ)/λ	θ	I(calc.)	I(calc.)/I(max)
1	0	0	1	0.044	1.80*	2776.75	0.08016

Results for the electron density study of XB donor **5** are given below.

Table S11: R-value statistics as a function of resolution (in resolution shells) of XB donor **5**.

θ	sin(θ)/λ	#	R1	wR2	S	Rs	av(I/SigW)	av(I)	av(SigW)
12.38	0.302	335	0.014	0.056	2.483	0.003	42.95	14211.95	295.15
15.68	0.38	339	0.014	0.056	2.328	0.003	40.03	7476.51	158.77
18.02	0.435	329	0.012	0.046	1.821	0.003	37.67	5243.01	113.78
19.90	0.479	347	0.016	0.051	1.863	0.003	34.17	3676.24	82.10
21.51	0.516	328	0.014	0.041	1.480	0.004	33.23	3102.32	70.67
22.94	0.548	337	0.014	0.040	1.357	0.005	31.50	2523.29	59.01
24.22	0.577	338	0.012	0.034	1.111	0.006	29.50	1870.20	45.75
25.40	0.603	339	0.012	0.031	1.023	0.006	29.90	1872.85	46.18
26.49	0.628	335	0.015	0.034	1.024	0.008	26.74	1316.90	34.93
27.52	0.65	337	0.012	0.031	0.916	0.009	26.19	1190.59	32.65
28.49	0.671	337	0.015	0.035	0.985	0.010	25.14	1014.53	28.83
29.41	0.691	324	0.016	0.034	0.974	0.010	25.30	1096.69	30.81
30.28	0.709	357	0.021	0.040	1.047	0.013	22.99	754.02	23.61
31.12	0.727	334	0.018	0.035	0.959	0.012	23.88	871.96	26.02
31.93	0.744	332	0.017	0.033	0.822	0.014	21.43	638.97	20.94
32.71	0.76	343	0.013	0.030	0.732	0.013	21.43	517.24	17.46
33.46	0.776	330	0.013	0.032	0.801	0.011	21.19	561.96	18.05
34.20	0.791	319	0.014	0.035	0.807	0.013	19.71	431.92	15.04
34.91	0.805	356	0.014	0.035	0.825	0.011	20.29	433.42	14.77
58.50	1.200	14767	0.031	0.067	0.884	0.031	9.52	112.87	6.64

Table S12: Resolution and completeness statistics (cumulative and Friedel pairs averaged) of XB donor **5**.

θ	sin(θ)/λ	Completeness [%]	Expected	Measured	Missing
20.82	0.500	99.9	1538	1537	1
23.01	0.550	100	2043	2042	1
25.24	0.600	100	2650	2649	1
27.51	0.650	100	3364	3363	1
29.84	0.700	100	4207	4206	1
32.21	0.750	100	5157	5156	1
34.65	0.800	100	6268	6267	1
37.17	0.850	100	7521	7520	1
39.77	0.900	100	8912	8911	1
42.47	0.950	100	10524	10523	1
45.29	1.000	100	12251	12250	1
48.27	1.050	100	14200	14199	1
51.43	1.100	100	16327	16326	1
54.82	1.150	100	18654	18653	1
58.50	1.200	100	21164	21163	1

$R_{sigma} = \sum\sigma_{Int} / \sum Int = 0.0073$

Table S13: Missing reflections of XB donor **5**.

Nr.	H	K	L	sin(θ)/λ	θ	I(calc.)	I(calc.)/I(max)
1	1	0	0	0.032	1.32*	31360.94	0.10612

Results for the electron density study of XB adduct **8** are given below.

Table S14: Resolution and completeness statistics (cumulative and Friedel pairs averaged) of XB adduct **8**.

θ	sin(θ)/λ	#	R1	wR2	S	Rs	av(I/SigW)	av(I)	av(SigW)
12.38	0.302	233	0.011	0.041	3.188	0.004	76.08	3170.71	38.09
15.68	0.38	234	0.009	0.035	2.682	0.003	74.08	1988.35	23.97
18.02	0.435	233	0.008	0.028	2.068	0.003	70.50	1474.39	18.19
19.90	0.479	222	0.010	0.031	2.167	0.004	67.80	1127.95	14.29
21.51	0.516	249	0.010	0.029	1.993	0.004	66.00	1044.80	13.38
22.94	0.548	224	0.009	0.024	1.631	0.004	65.08	829.10	10.97
24.22	0.577	245	0.008	0.022	1.487	0.004	63.17	741.01	9.98
25.40	0.603	234	0.008	0.020	1.328	0.005	60.92	661.50	9.09
26.49	0.628	232	0.008	0.022	1.311	0.005	55.21	514.14	7.35
27.52	0.65	228	0.007	0.020	1.229	0.005	57.97	509.86	7.33
28.49	0.671	228	0.007	0.017	1.057	0.005	59.19	497.72	7.20
29.41	0.691	241	0.007	0.019	1.108	0.006	55.12	421.69	6.29
30.28	0.709	228	0.008	0.020	1.109	0.006	52.16	361.75	5.60
31.12	0.727	235	0.008	0.020	1.126	0.006	52.18	347.06	5.40
31.93	0.744	237	0.008	0.019	1.088	0.006	52.72	334.03	5.24
32.71	0.76	235	0.007	0.019	1.067	0.006	51.82	314.88	5.00
33.46	0.776	233	0.009	0.024	1.272	0.006	49.11	274.23	4.48
34.20	0.791	225	0.008	0.021	1.070	0.007	47.83	246.05	4.15
34.91	0.805	246	0.007	0.019	1.004	0.007	47.84	249.60	4.21
51.50	1.101	6895	0.009	0.021	0.806	0.011	34.89	112.47	2.47

Table S15: R-value statistics as a function of resolution (in resolution shells) of XB adduct **8**.

θ	sin(θ)/λ	Completeness [%]	Expected	Measured	Missing
20.82	0.500	99.9	1059	1058	1
23.01	0.550	99.9	1408	1407	1
25.24	0.600	99.9	1836	1835	1
27.51	0.650	100	2334	2333	1
29.84	0.700	100	2913	2912	1
32.21	0.750	100	3583	3582	1
34.65	0.800	100	4356	4355	1
37.17	0.850	100	5212	5211	1
39.77	0.900	100	6197	6196	1
42.47	0.950	100	7285	7284	1
45.29	1.000	100	8494	8493	1
48.27	1.050	100	9855	9854	1
51.43	1.100	100	11306	11305	1
51.50	1.101	100	11338	11337	1

$R_{sigma} = \sum\sigma_{Int} / \sum Int =$ 0.0055

Table S16: Missing reflections of XB adduct **8**.

Nr.	H	K	L	sin(θ)/λ	θ	I(calc.)	I(calc.)/I(max)
1	0	0	1	0.037	1.51*	2067.06	0.10158

Results for the electron density study of XB donor **4** are given below.

Table S171: R-value statistics as a function of resolution (in resolution shells) of XB donor **4**.

θ	sin(θ)/λ	#	R1	wR2	S	Rs	av(I/SigW)	av(I)	av(SigW)
12.38	0.302	84	0.010	0.025	2.217	0.006	84.86	19261.07	196.63
15.68	0.38	88	0.010	0.026	2.008	0.004	69.80	7959.24	78.45
18.02	0.435	82	0.009	0.026	1.983	0.004	66.97	7160.67	69.91
19.90	0.479	79	0.012	0.030	1.957	0.004	55.67	3682.96	40.57
21.51	0.516	91	0.012	0.028	1.901	0.004	57.46	4276.08	45.82
22.94	0.548	86	0.011	0.026	1.663	0.004	53.36	3587.47	39.93
24.22	0.577	82	0.015	0.034	1.852	0.005	44.58	2070.78	26.86
25.40	0.603	89	0.013	0.028	1.694	0.005	50.67	2597.55	32.21
26.49	0.628	78	0.011	0.026	1.551	0.005	51.74	2347.35	30.21
27.52	0.65	86	0.017	0.033	1.668	0.006	40.82	1501.29	21.72
28.49	0.671	84	0.014	0.029	1.493	0.005	41.18	1735.80	23.84
29.41	0.691	87	0.018	0.033	1.575	0.006	38.08	1326.66	20.04
30.28	0.709	89	0.013	0.026	1.273	0.006	40.48	1234.68	19.93
31.12	0.727	85	0.018	0.033	1.467	0.007	36.41	1003.05	17.63
31.93	0.744	82	0.013	0.025	1.180	0.006	37.37	1157.90	18.60
32.71	0.76	88	0.013	0.028	1.258	0.006	36.03	1077.89	17.71
33.46	0.776	81	0.026	0.045	1.689	0.008	28.52	683.97	13.21
34.20	0.791	85	0.019	0.033	1.353	0.007	31.85	836.09	15.21
34.91	0.805	76	0.020	0.035	1.327	0.008	30.26	691.58	13.73
58.50	1.200	3725	0.023	0.045	1.014	0.013	16.33	217.73	6.82

Table S18: Resolution and completeness statistics (cumulative and Friedel pairs averaged) of XB donor **4**.

θ	sin(θ)/λ	Completeness [%]	Expected	Measured	Missing
20.82	0.500	100	382	382	0
23.01	0.550	100	515	515	0
25.24	0.600	100	668	668	0
27.51	0.650	100	844	844	0
29.84	0.700	100	1053	1053	0
32.21	0.750	100	1298	1298	0
34.65	0.800	100	1579	1579	0
37.17	0.850	100	1895	1895	0
39.77	0.900	100	2244	2244	0
42.47	0.950	100	2647	2647	0
45.29	1.000	100	3085	3085	0
48.27	1.050	100	3572	3572	0
51.43	1.100	100	4110	4110	0
54.82	1.150	100	4694	4694	0
58.50	1.200	100	5327	5327	0

$R_{sigma} = \sum\sigma_{Int} / \sum Int = 0.0062$

Results for the electron density study of XB adduct **9** are given below.

Table S19: R-value statistics as a function of resolution (in resolution shells) of XB adduct **9**.

θ	sin(θ)/λ	#	R1	wR2	S	Rs	av(I/SigW)	av(I)	av(SigW)
12.38	0.302	494	0.018	0.048	2.930	0.005	57.45	6178.48	88.78
15.68	0.38	487	0.013	0.041	2.272	0.005	51.34	3939.98	57.50
18.02	0.435	507	0.011	0.032	1.743	0.005	48.91	2974.69	45.16
19.90	0.479	471	0.012	0.033	1.686	0.006	46.03	2221.72	35.50
21.51	0.516	497	0.011	0.028	1.423	0.007	44.93	2029.14	33.25
22.94	0.548	503	0.011	0.028	1.285	0.008	40.75	1513.51	26.26
24.22	0.577	495	0.011	0.025	1.185	0.007	40.87	1380.65	23.95
25.40	0.603	490	0.010	0.024	1.084	0.007	38.84	1248.66	22.17
26.49	0.628	482	0.011	0.024	1.015	0.008	36.89	1022.21	19.25
27.52	0.65	492	0.011	0.024	1.024	0.009	35.51	951.70	18.35
28.49	0.671	495	0.011	0.025	1.013	0.010	34.76	866.72	17.32
29.41	0.691	519	0.011	0.023	0.888	0.011	31.94	717.93	15.43
30.28	0.709	470	0.011	0.022	0.853	0.011	32.70	708.59	15.50
31.12	0.727	465	0.011	0.023	0.849	0.013	30.24	616.35	14.15
31.93	0.744	531	0.011	0.024	0.891	0.012	31.69	640.97	14.43
32.71	0.76	486	0.011	0.024	0.826	0.013	29.03	519.26	12.58
33.46	0.776	491	0.011	0.024	0.817	0.014	28.46	478.43	11.98
34.20	0.791	495	0.012	0.024	0.812	0.015	27.55	455.81	11.68
34.91	0.805	460	0.011	0.026	0.874	0.014	27.74	447.95	11.26
51.50	1.101	14643	0.018	0.038	0.882	0.023	18.31	118.49	6.65

Table S20: Resolution and completeness statistics (cumulative and Friedel pairs averaged) of XB adduct **9**.

θ	sin(θ)/λ	Completeness [%]	Expected	Measured	Missing
20.82	0.500	99.9	2243	2241	2
23.01	0.550	99.9	2986	2984	2
25.24	0.600	99.9	3884	3882	2
27.51	0.650	100	4918	4916	2
29.84	0.700	100	6160	6158	2
32.21	0.750	100	7575	7573	2
34.65	0.800	100	9161	9159	2
37.17	0.850	100	11024	11022	2
39.77	0.900	100	13069	13067	2
42.47	0.950	100	15366	15364	2
45.29	1.000	100	17946	17944	2
48.27	1.050	100	20734	20732	2
51.43	1.100	100	23896	23894	2
51.50	1.101	100	23975	23973	2

$R_{sigma} = \sum\sigma_{Int} / \sum Int =$ 0.0096

Table S21: Missing reflections of XB adduct **9**.

Nr.	H	K	L	sin(θ)/λ	θ	I(calc.)	I(calc.)/I(max)
1	0	0	1	0.028	1.13*	70.30	0.00087
2	0	1	1	0.056	2.29	2687.02	0.03328

Results for the electron density study of XB donor **6** are given below.

Table S22: R-value statistics as a function of resolution (in resolution shells) of XB donor **6**.

θ	sin(θ)/λ	#	R1	wR2	S	Rs	av(I/SigW)	av(I)	av(SigW)
12.38	0.302	94	0.011	0.053	2.462	0.006	44.66	6813.51	135.74
15.68	0.38	96	0.012	0.057	2.491	0.004	41.53	3710.84	72.97
18.02	0.435	92	0.010	0.042	1.847	0.004	42.52	3008.65	59.62
19.90	0.479	93	0.010	0.042	1.771	0.004	40.18	2104.70	42.47
21.51	0.516	94	0.010	0.032	1.338	0.004	40.09	1922.03	39.23
22.94	0.548	97	0.008	0.030	1.238	0.005	38.50	1653.99	34.08
24.22	0.577	93	0.010	0.028	1.116	0.005	36.77	1355.44	28.50
25.40	0.603	95	0.012	0.035	1.332	0.005	34.35	1114.80	23.91
26.49	0.628	92	0.011	0.033	1.202	0.006	33.30	901.69	19.93
27.52	0.65	95	0.012	0.033	1.195	0.006	32.11	907.62	20.12
28.49	0.671	94	0.014	0.029	1.024	0.007	31.35	747.86	17.30
29.41	0.691	94	0.015	0.029	1.052	0.008	32.47	758.69	17.77
30.28	0.709	94	0.016	0.034	1.137	0.008	29.68	623.06	15.04
31.12	0.727	93	0.011	0.029	0.986	0.009	29.94	595.75	14.48
31.93	0.744	96	0.018	0.041	1.284	0.010	26.67	477.60	12.30
32.71	0.76	103	0.010	0.027	0.906	0.009	29.65	492.01	12.38
33.46	0.776	90	0.013	0.031	1.015	0.008	29.57	439.40	11.07
34.20	0.791	89	0.014	0.042	1.294	0.008	27.31	369.14	9.44
34.91	0.805	95	0.011	0.031	1.033	0.008	30.07	393.79	9.95
58.50	1.200	4130	0.018	0.043	0.927	0.015	17.43	122.32	4.25

Table S23: Resolution and completeness statistics (cumulative and Friedel pairs averaged) of XB donor **6**.

θ	sin(θ)/λ	Completeness [%]	Expected	Measured	Missing
20.82	0.500	100	424	424	0
23.01	0.550	100	567	567	0
25.24	0.600	100	743	743	0
27.51	0.650	100	941	941	0
29.84	0.700	100	1176	1176	0
32.21	0.750	100	1452	1452	0
34.65	0.800	100	1752	1752	0
37.17	0.850	100	2103	2103	0
39.77	0.900	100	2507	2507	0
42.47	0.950	100	2945	2945	0
45.29	1.000	100	3441	3441	0
48.27	1.050	100	3951	3951	0
51.43	1.100	100	4565	4565	0
54.82	1.150	100	5220	5220	0
58.50	1.200	100	5919	5919	0

$R_{sigma} = \sum\sigma_{Int} / \sum Int =$ 0.0070

3.3 Refinement details for 4-9

Refinements were conducted with all intensity data $I > 0$. The final refinements on F^2 comprised multipoles up to hexadecapoles for non-H atoms and up to bond directed dipoles for the H atoms. Contraction parameters for non-H atoms and ϰ' for I were refined freely; ϰ' was fixed to 1.0 for F, N and C and to 1.2 for H atoms. With the exception of adduct **4**, positional parameters of H atoms were freely refined. As examples for quality control, scatter plots for the MM refinements of **5**, **7** and **8** are provided.

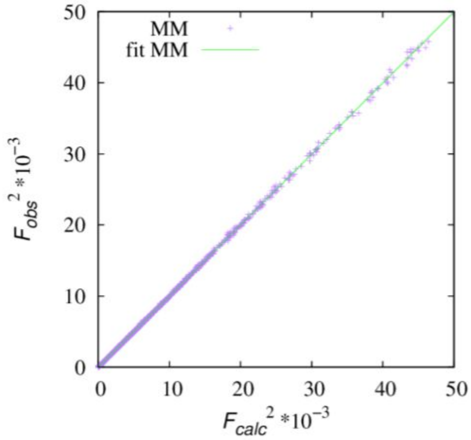

5

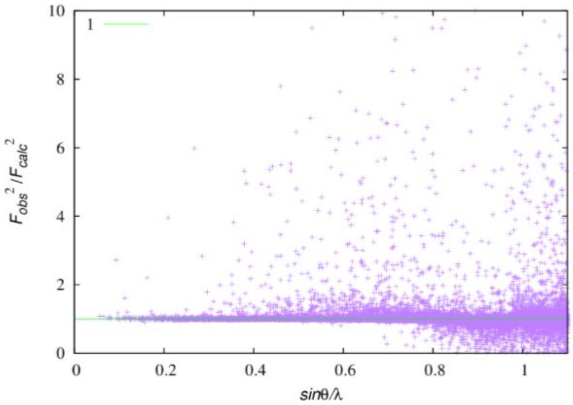

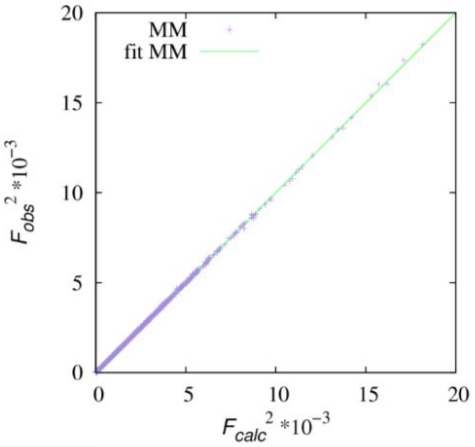

7

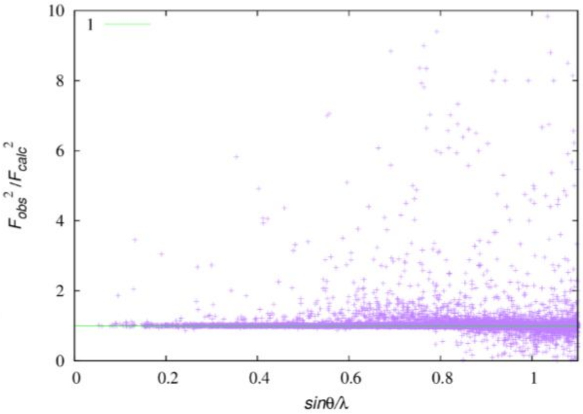

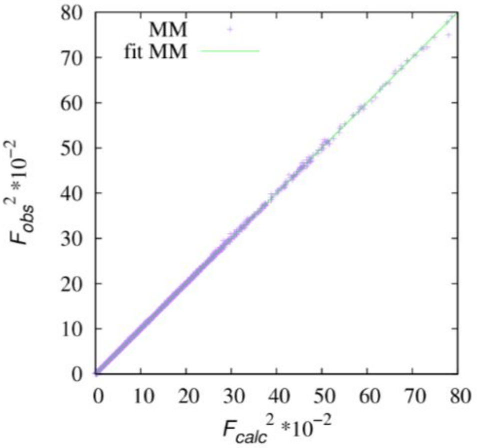

8

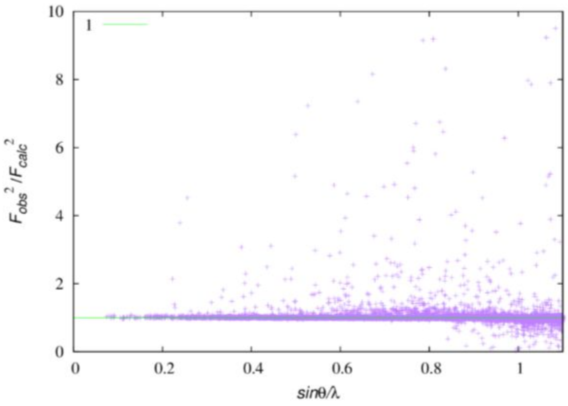

Figure S7: Scatterplots for X-ray refinement results (MM) for the XB donor **5** and XB adducts **7**,**8**.

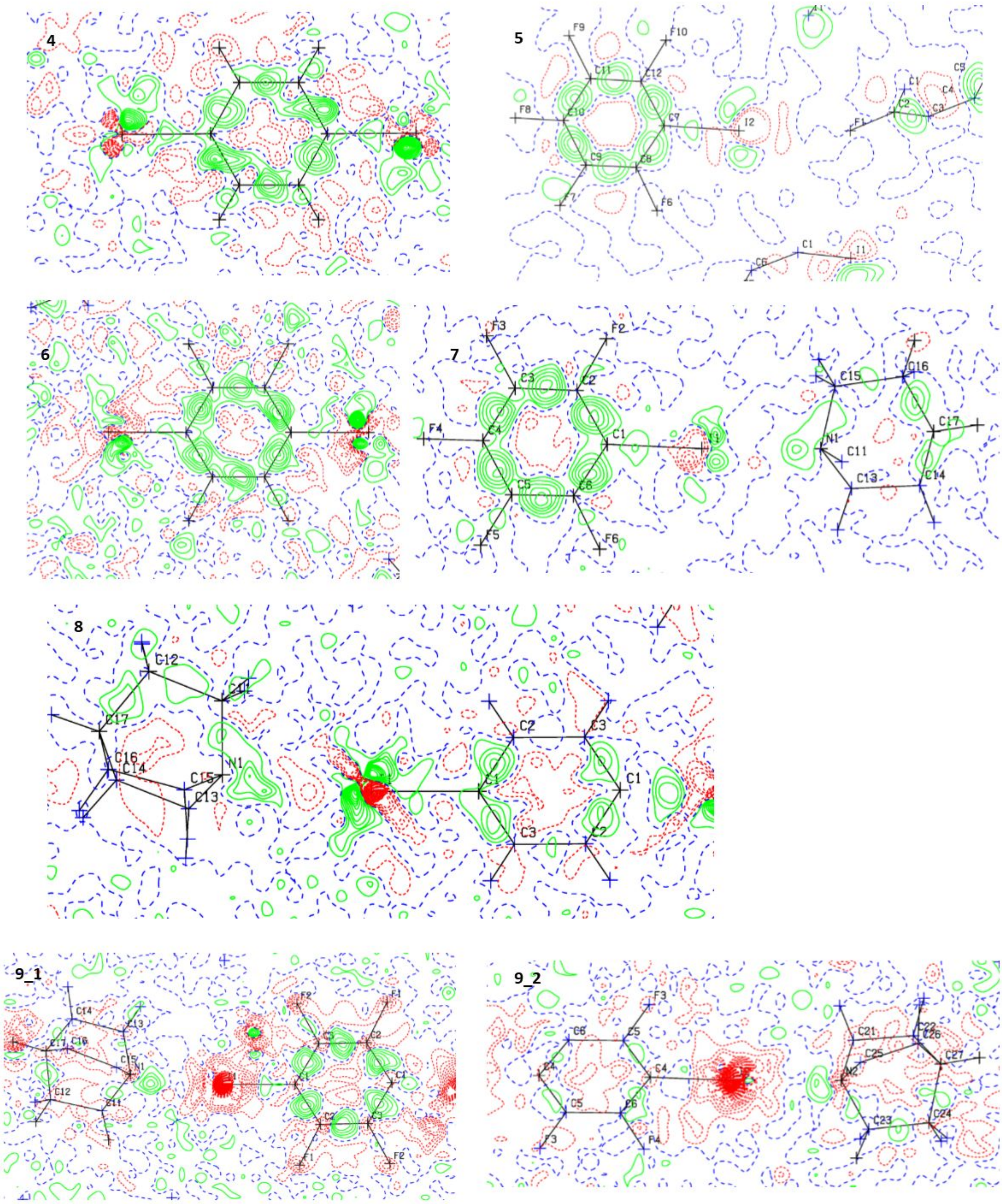

Figure S8: Residual electron density maps for the XB donors **4**-**6** and the XB adducts **7**-**9** after the IAM refinement. The contour interval is 0.10 e·Å^{-3}, green lines indicate positive, red lines negative and blue lines zero contours.

3.4 Deformation electron density

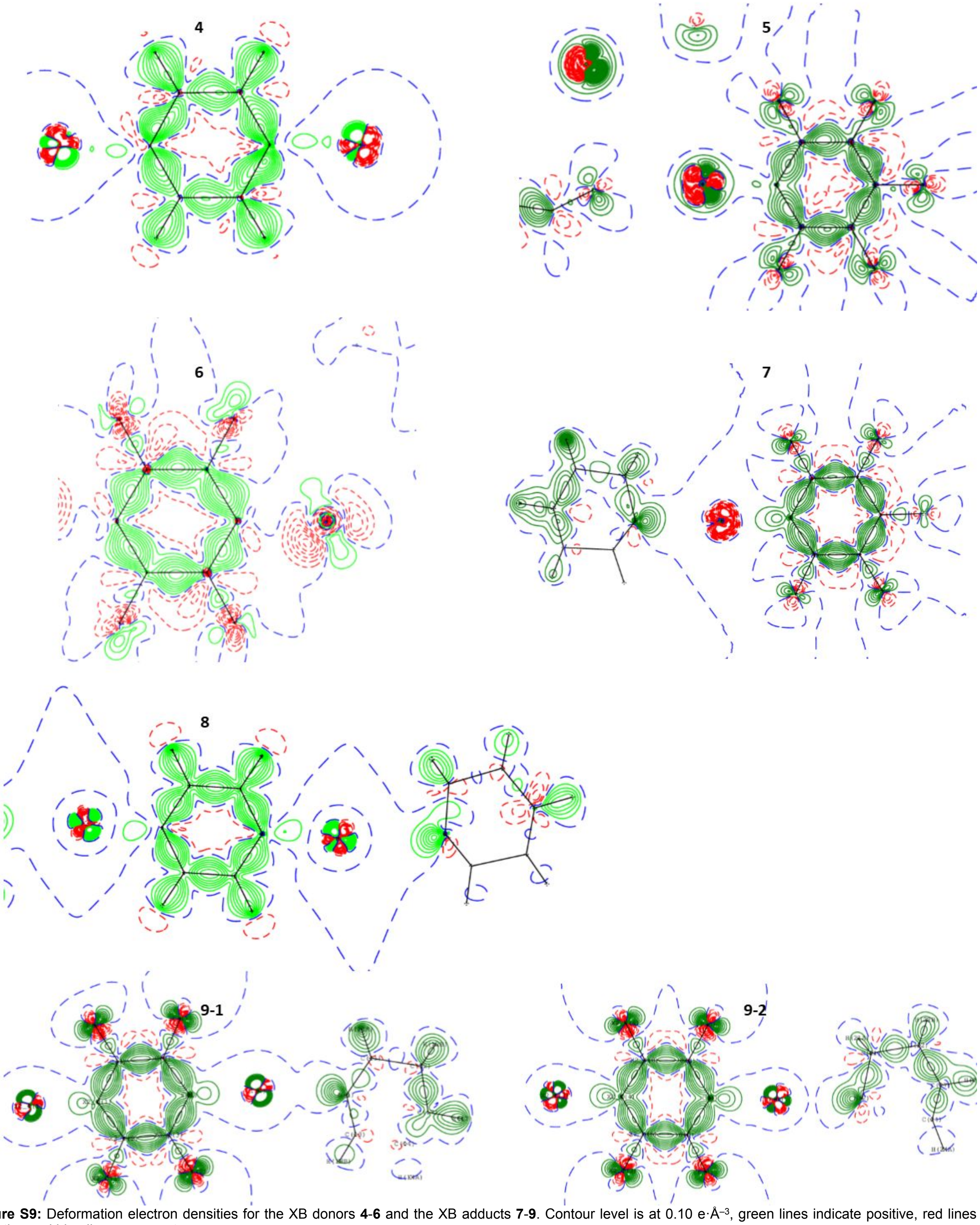

Figure S9: Deformation electron densities for the XB donors **4**-**6** and the XB adducts **7**-**9**. Contour level is at 0.10 e·Å^{-3}, green lines indicate positive, red lines negative and blue lines zero contours.

3.5 Electrostatic potential

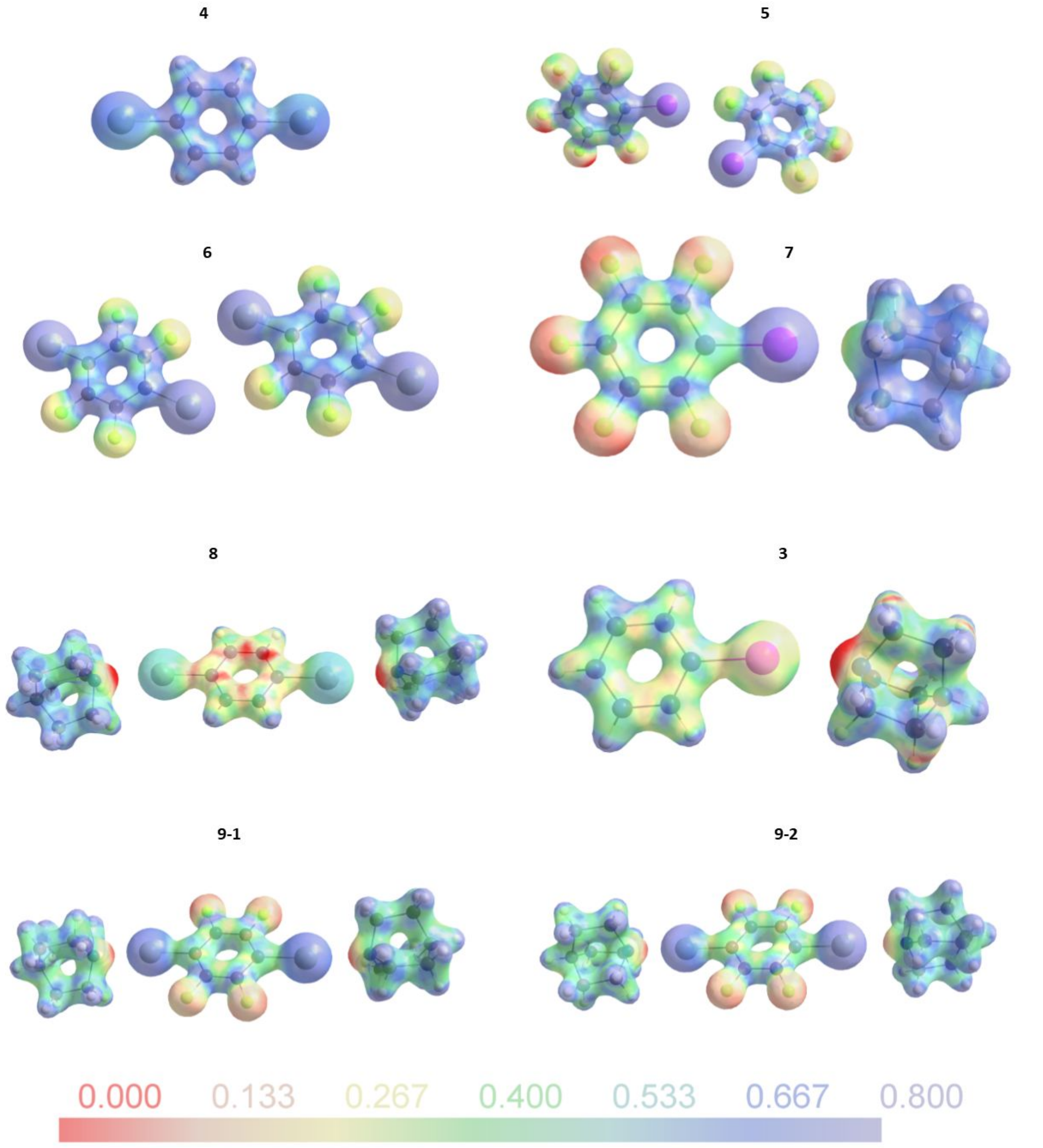

Figure S10: Electrostatic potential mapped on an isosurface pf electron density (ρ = 0.5 e·Å^{-3}) of the XB donors **4**-**6** and the XB adducts **3**, **7**-**9** (program *MoleCoolQt* [7]).

3.6 Topological analysis

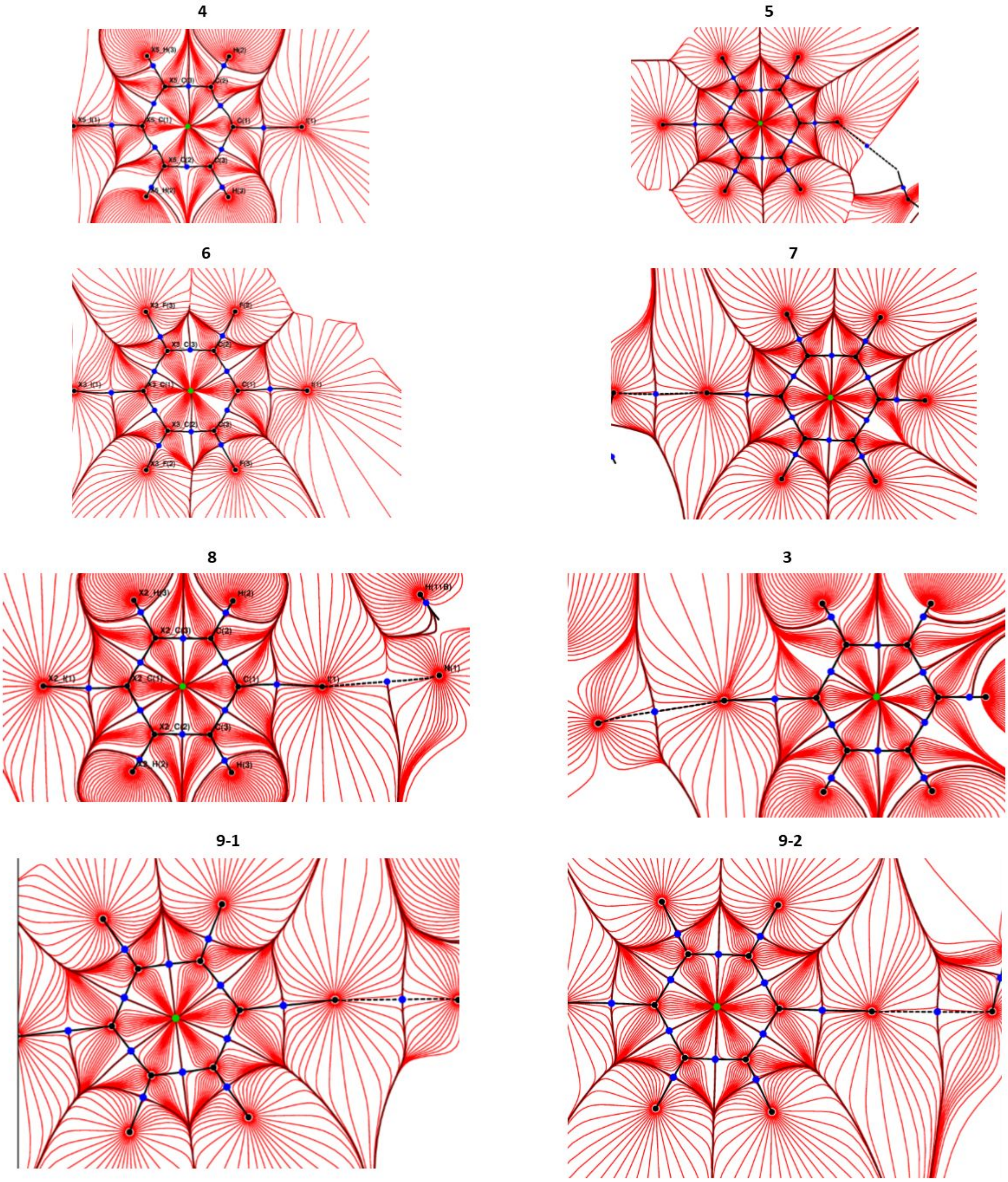

Figure S11: Atom basins of the XB donors **4**-**6** and the XB adducts **3**, **7**-**9**. Bond paths are shown as black solid (covalent bond) or dotted (interaction) lines. Bond critical points are displayed as blue dots, green dots mark ring critical points and black ones the atom positions.

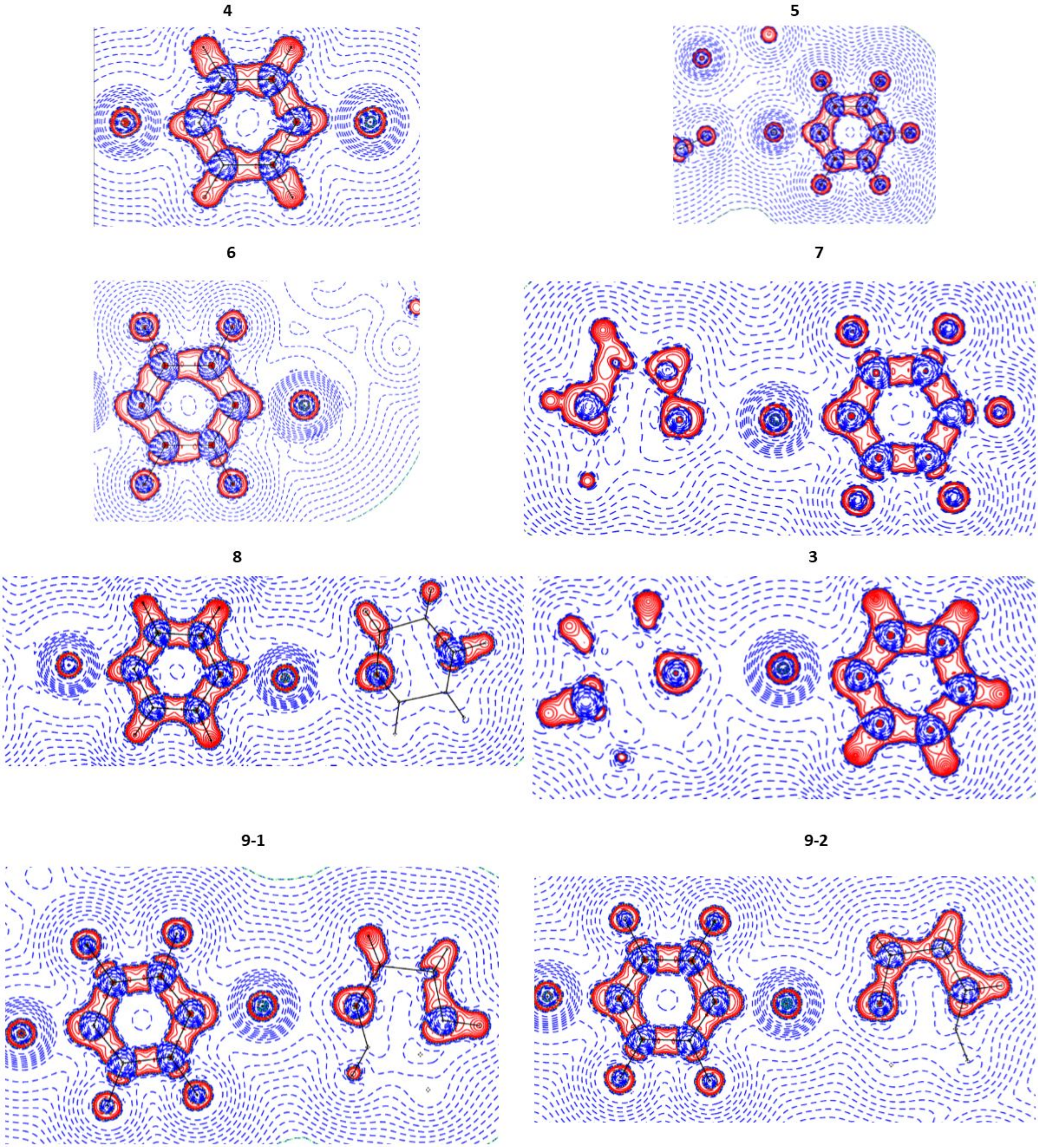

Figure S12: Laplacian of the electron density of the XB donors **4**-**6** and the XB adducts **3**, **7**-**9**, with positive values in blue, negative values in red and contours at $\pm 2^n \cdot 10^{-3}$ e·Å^{-5}.

Energy densities in the bcps may be used to categorize secondary interactions. The kinetic energy density *G* and the ratio between kinetic energy density and electron density, *G*/ρ in the bcp, were derived as suggested by Abramov [8], and the potential energy density *V* was obtained according to the local virial theorem [9, 10].

Table S24: Properties of the electron density in the bcps of the halogen bonds XB adducts **3**, **7**-**9**. R_{12} is the bond path, d_1 its component with respect to the first atom, ρ the electron density, ∇^2 the Laplacian in the bcp, *G* the kinetic, *V* the potential and *E* the total energy density.

Bond	Dist. [Å]	R_{ij} [Å]	d_1 [Å]	d_2 [Å]	ρ [e·Å^{-3}]	∇^2 [e·Å^{-5}]	*G* [a.u.]	*G/ρ* [a.u.]	*V* [a.u.]	*E* [a.u.]
I1···N1										
**3**	2.9301(4)	2.9309	1.6115	1.3194	0.187(4)	1.716(2)	0.0191	0.69	–0.0205	–0.0013
**7**	2.6781(2)	2.6783	1.4628	1.2155	0.270(4)	2.477(3)	0.0306	0.76	–0.0354	–0.0049
**8**	2.9568(3)	2.9574	1.6363	1.3211	0.180(3)	1.658(2)	0.0183	0.69	–0.0194	–0.0011
**9**	2.7173(5)	2.7174	1.4838	1.2337	0.260(4)	2.359(3)	0.0289	0.75	–0.0334	–0.0044
	2.7476(5)	2.7476	1.4938	1.2538	0.249(4)	2.192(2)	0.0269	0.73	–0.0310	–0.0041
I1–C1										
**3**	2.1091(4)	2.1106	1.1631	0.9475	0.80(2)	2.76(5)				
**7**	2.1295(3)	2.1296	1.1558	0.9739	0.76(2)	3.39(3)				
**8**	2.1096(3)	2.1127	1.1443	0.9685	0.84(2)	1.36(3)				
**9**	2.1142(4)	2.1144	1.1492	0.9652	0.79(2)	2.75(3)				
	2.1189(5)	2.1190	1.1356	0.9834	0.82(2)	2.48(3)				

Table S25: Properties of the electron density in the bcps of the XB adduct **7**. R_{12} is the bond path length, d_1 its component with respect to the first atom, ρ the electron density, ∇^2 the Laplacian in the bcp.

Bond	Dist. [Å]	R_{ij} [Å]	d_1 [Å]	d_2 [Å]	ρ [e·Å^{-3}]	∇^2 [e·Å^{-5}]
I1···N1	2.6781(2)	2.6783	1.4628	1.2155	0.270(4)	2.477(3)
I1–C1	2.1295(3)	2.1296	1.1558	0.9739	0.76(2)	3.39(3)
F2–C2	1.3390(3)	1.3391	0.8145	0.5246	2.03(3)	–21.7(2)
F3–C3	1.3361(3)	1.3363	0.8192	0.5237	2.02(3)	–20.4(2)
F4–C4	1.3306(3)	1.3307	0.7994	0.5312	2.09(4)	–19.4(2)
F5–C5	1.3328(3)	1.3330	0.8144	0.5186	2.03(2)	–20.9(2)
F6–C6	1.3364(3)	1.3365	0.8146	0.5218	2.04(2)	–21.9(2)
N1–C11	1.4761(4)	1.4761	0.8009	0.6751	1.85(2)	–13.05(7)
N1–C13	1.4740(4)	1.4740	0.8012	0.6728	1.85(2)	–14.88(6)
N1–C15	1.4763(4)	1.4766	0.7997	0.6769	1.83(2)	–13.58(6)
C1–C2	1.3905(3)	1.3906	0.6647	0.7260	2.16(3)	–19.57(9)
C1–C6	1.3899(3)	1.3900	0.6489	0.7411	2.11(4)	–19.8(2)
C2–C3	1.3903(4)	1.3910	0.6859	0.7051	2.23(3)	–20.35(8)
C3–C4	1.3898(4)	1.3903	0.7253	0.6650	2.21(3)	–22.2(2)
C4–C5	1.3875(4)	1.3878	0.6674	0.7204	2.16(4)	–19.5(2)
C5–C6	1.3879(3)	1.3886	0.7053	0.6833	2.26(2)	–21.5(2)
C11–C12	1.5461(5)	1.5462	0.7848	0.7614	1.69(2)	–14.91(5)
C12–C17	1.5345(4)	1.5355	0.7957	0.7397	1.72(3)	–15.95(8)
C13–C14	1.5505(5)	1.5507	0.7908	0.7599	1.68(2)	–13.7(2)
C14–C17	1.5349(5)	1.5349	0.7895	0.7454	1.70(3)	–14.50(8)
C15–C16	1.5436(5)	1.5438	0.7872	0.7566	1.69(2)	–14.1(2)
C16–C17	1.5353(5)	1.5355	0.7848	0.7507	1.72(3)	–15.02(8)

Table S26: Properties of the electron density in the bcps of the XB donor **5**. R_{12} is the bond path length, d_1 its component with respect to the first atom, ρ the electron density, ∇^2 the Laplacian in the bcp, G the kinetic, V the potential and E the total energy density.

Bond	Dist. [Å]	R_{ij} [Å]	d_1 [Å]	d_2 [Å]	ρ [e·Å^{-3}]	∇^2 [e·Å^{-5}]	G [a.u.]	G/ρ [a.u.]	V [a.u.]	E [a.u.]
I2···F1	3.0718(5)	3.0724	1.7478	1.3245	0.081(2)	1.063(2)	0.0092	0.76	–0.0073	0.0019
F6···C5^k	3.1369(9)	3.1418	1.4909	1.6509	0.036(2)	0.662(2)	0.0050	0.95	–0.0032	0.0018
F3···F7^i	2.7759(8)	2.7761	1.3726	1.4035	0.059(2)	0.958(2)	0.0077	0.88	–0.0054	0.0022
F4···F6^k	2.8627(8)	2.8636	1.4434	1.4203	0.046(2)	0.846(2)	0.0066	0.96	–0.0043	0.0022
F10···C3^j	3.0253(8)	3.0652	1.4086	1.6566	0.054(3)	0.696(2)	0.0057	0.72	–0.0042	0.0015
F10···C4^j	3.0495(9)	3.0938	1.5303	1.5635	0.037(4)	0.734(3)	0.0056	1.02	–0.0035	0.0020
C1–I1	2.0760(6)	2.0766	0.9244	1.1523	0.79(2)	3.9(2)				
C7–I2	2.0716(6)	2.0785	0.9297	1.1488	0.82(2)	2.3(2)				
F1–C2	1.3377(7)	1.3377	0.8053	0.5324	1.97(2)	–15.52(9)				
F2–C3	1.3289(7)	1.3296	0.8126	0.5170	1.97(3)	–16.3(2)				
F3–C4	1.3302(8)	1.3311	0.7990	0.5320	1.99(3)	–13.3(2)				
F4–C5	1.3288(7)	1.3293	0.8130	0.5163	1.96(2)	–16.2(2)				
F5–C6	1.3347(7)	1.3347	0.8067	0.5281	1.97(2)	–15.9(2)				
F6–C8	1.3358(8)	1.3365	0.8078	0.5286	1.97(3)	–15.93(9)				
F7–C9	1.3319(8)	1.3323	0.8051	0.5273	1.97(3)	–15.0(2)				
F8–C10	1.3312(9)	1.3335	0.8001	0.5335	1.87(4)	–11.1(2)				
F9–C11	1.3320(9)	1.3324	0.8048	0.5276	1.97(2)	–15.0(2)				
F10–C12	1.3362(8)	1.3367	0.8054	0.5313	1.98(2)	–15.6(2)				
C1–C2	1.3904(8)	1.3909	0.6855	0.7054	2.14(3)	–18.9(2)				
C1–C6	1.3918(8)	1.3919	0.6785	0.7134	2.09(4)	–18.0(2)				
C2–C3	1.3848(8)	1.3852	0.7249	0.6603	2.21(3)	–23.0(2)				
C3–C4	1.3888(8)	1.3906	0.6949	0.6957	2.12(3)	–20.1(2)				
C4–C5	1.3873(9)	1.3876	0.7249	0.6626	2.18(3)	–21.1(2)				
C5–C6	1.3913(9)	1.3919	0.6733	0.7186	2.22(2)	–21.6(2)				
C7–C8	1.3901(8)	1.3903	0.6989	0.6914	2.09(3)	–18.3(2)				
C7–C12	1.3881(9)	1.3894	0.6590	0.7305	2.13(4)	–18.9(2)				
C8–C9	1.3853(9)	1.3875	0.7330	0.6545	2.14(3)	–19.5(2)				
C9–C10	1.3880(11)	1.3897	0.6994	0.6903	2.23(3)	–22.7(2)				
C10–C11	1.3892(10)	1.3897	0.7357	0.6540	2.18(4)	–21.0(2)				
C11–C12	1.3896(9)	1.3913	0.7089	0.6825	2.18(3)	–20.9(2)				

i = $+x, +y, +z$; j = $-x, ½+y, ½-z$; k = $-x, -y, -z$

Table S27: Properties of the electron density in the bcps of the XB adduct **8**. R_{12} is the bond path length, d_1 its component with respect to the first atom, ρ the electron density, ∇^2 the Laplacian in the bcp, G the kinetic, V the potential and E the total energy density.

Bond	Dist. [Å]	R_{ij} [Å]	d_1 [Å]	d_2 [Å]	ρ [e·Å^{-3}]	∇^2 [e·Å^{-5}]	G [a.u.]	G/ρ [a.u.]	V [a.u.]	E [a.u.]
I1···N1	2.9567(2)	2.9574	1.6363	1.3211	0.180(3)	1.658(2)	0.0183	0.69	–0.0194	–0.0011
I1–C1	2.1094(1)	2.1127	1.1443	0.9685	0.84(2)	1.36(3)				
N1–C11	1.4723(1)	1.4726	0.8140	0.6586	1.77(3)	–12.83(8)				
N1–C13	1.4715(1)	1.4724	0.8260	0.6464	1.80(2)	–14.14(5)				
N1–C15	1.4722(1)	1.4724	0.8054	0.6670	1.80(2)	–12.51(6)				
C1–C2	1.3960(3)	1.3961	0.6961	0.7000	2.16(3)	–21.5(2)				
C1–C3	1.3965(3)	1.3968	0.6801	0.7167	2.07(4)	–18.6(2)				
C11–C12	1.5477(1)	1.5478	0.7789	0.7689	1.63(2)	–12.60(6)				
C12–C17	1.5343(1)	1.5347	0.7828	0.7519	1.72(3)	–14.26(8)				
C13–C14	1.5512(1)	1.5517	0.7632	0.7885	1.68(2)	–12.89(2)				
C14–C17	1.5338(3)	1.5345	0.7736	0.7609	1.60(3)	–11.78(8)				
C15–C16	1.5530(1)	1.5537	0.7555	0.7983	1.65(2)	–13.10(2)				
C16–C17	1.5334(3)	1.5336	0.7561	0.7775	1.71(3)	–14.41(7)				

Table S28: Properties of the electron density in the bcps of the XB donor **4** and calculated values. R_{12} is the bond path length, d_1 its component with respect to the first atom, ρ the electron density, ∇^2 the Laplacian in the bcp.

Bond	Dist. [Å]	R_{ij} [Å]	d_1 [Å]	d_2 [Å]	ρ [e·Å^{-3}]	∇^2 [e·Å^{-5}]
I1–C1	2.0959(6)	2.0966	1.1610	0.9356	0.836(2)	1.073(2)
C2–C1	1.3945(8)	1.3960	0.6414	0.7546	2.145(2)	–19.876(2)
C1–C3	1.3935(8)	1.3963	0.7741	0.6222	2.100(2)	–19.920(2)
C2–C3^i	1.4012(8)	1.4044	0.6860	0.7184	2.036(2)	–18.332(2)

i = –*x*, 1–*y*, –*z*

Table S29: Properties of the electron density in the bcps of the XB adduct **9** and calculated values. R_{12} is the bond path length, d_1 its component with respect to the first atom, ρ the electron density, ∇^2 the Laplacian in the bcp, G the kinetic, V the potential and E the total energy density.

Bond	Dist. [Å]	R_{ij} [Å]	d_1 [Å]	d_2 [Å]	ρ [e·Å^{-3}]	∇^2 [e·Å^{-5}]	G [a.u.]	G/ρ [a.u.]	V [a.u.]	E [a.u.]
I1···N1	2.7172(3)	2.7174	1.4838	1.2337	0.260(4)	2.36(3)	0.0289	0.75	–0.0334	–0.0044
I2···N2	2.7471(3)	2.7476	1.4938	1.2538	0.249(4)	2.19(3)	0.0269	0.73	–0.0310	–0.0041
F1···F1^i	2.8674(3)	2.8720	1.4532	1.4187	0.046(2)	0.794(2)	0.0062	0.91	–0.0042	0.0020
F2···H26B	2.481(3)	2.5096	1.4195	1.0901	0.052(2)	0.756(2)	0.0061	0.79	–0.0043	0.0018
I1–C1	2.1143(2)	2.1144	1.1492	0.9652	0.79(2)	2.75(3)				
I2–C4	2.1193(2)	2.1190	1.1356	0.9834	0.82(2)	2.48(3)				
F1–C2	1.3400(4)	1.3402	0.8218	0.5184	1.98(2)	–20.6(2)				
F2–C3	1.3410(4)	1.3412	0.8271	0.5142	1.95(2)	–21.44(2)				
F3–C5	1.3389(5)	1.3391	0.8222	0.5169	1.98(2)	–20.80(2)				
F4–C6	1.3409(4)	1.3410	0.8214	0.5196	1.98(2)	–20.54(2)				
N1–C11	1.4745(2)	1.4754	0.8076	0.6677	1.81(3)	–11.87(7)				
N1–C13	1.4761(2)	1.4765	0.8119	0.6646	1.82(2)	–12.00(6)				
N1–C15	1.4748(2)	1.4753	0.7987	0.6766	1.77(3)	–10.58(7)				
N2–C21	1.4750(2)	1.4755	0.8011	0.6744	1.80(3)	–11.41(7)				
N2–C23	1.4738(2)	1.4743	0.8032	0.6711	1.81(3)	–11.14(7)				
N2–C25	1.4760(2)	1.4769	0.8095	0.6674	1.82(3)	–11.80(7)				
C1–C2	1.3894(5)	1.3897	0.6889	0.7008	2.19(3)	–21.2(2)				
C2–C3	1.3865(4)	1.3869	0.6974	0.6895	2.20(2)	–20.18(5)				
C4–C5	1.3898(5)	1.3899	0.6785	0.7113	2.14(3)	–19.2(2)				
C4–C6	1.3896(5)	1.3901	0.6848	0.7053	2.19(3)	–20.4(2)				
C11–C12	1.5482(2)	1.5485	0.7733	0.7752	1.65(2)	–12.54(2)				
C12–C17	1.5318(5)	1.5324	0.7625	0.7699	1.69(2)	–13.17(2)				
C13–C14	1.5456(2)	1.5456	0.7743	0.7713	1.65(2)	–12.62(2)				
C14–C17	1.5312(5)	1.5312	0.7570	0.7743	1.70(2)	–12.18(2)				
C15–C16	1.5445(2)	1.5445	0.7710	0.7735	1.66(2)	–12.80(2)				
C16–C17	1.5300(5)	1.5301	0.7642	0.7659	1.68(2)	–13.21(2)				
C21–C22	1.5456(2)	1.5458	0.7716	0.7742	1.64(2)	–12.44(2)				
C22–C27	1.5309(6)	1.5309	0.7570	0.7739	1.70(2)	–12.22(2)				
C23–C24	1.5493(3)	1.5492	0.7707	0.7786	1.64(2)	–12.41(2)				
C24–C27	1.5322(2)	1.5320	0.7650	0.7671	1.68(2)	–13.07(2)				
C25–C26	1.5454(2)	1.5455	0.7688	0.7767	1.65(2)	–12.44(2)				
C26–C27	1.5305(6)	1.5306	0.7614	0.7692	1.69(2)	–13.28(2)				

i = 1–*x*, 1–*y*, 1–*z*

Table S30: Properties of the electron density in the bcps of the XB donor **6** and calculated values. R_{12} is the bond path length, d_1 its component with respect to the first atom, ρ the electron density, ∇^2 the Laplacian in the bcp, G the kinetic, V the potential and E the total energy density.

Bond	Dist. [Å]	R_{ij} [Å]	d_1 [Å]	d_2 [Å]	ρ [e·Å^{-3}]	∇^2 [e·Å^{-5}]	G [a.u.]	G/ρ [a.u.]	V [a.u.]	E [a.u.]
I1···F2^i	3.4588(4)	3.4625	2.0441	1.4184	0.044(2)	0.528(2)	0.0043	0.66	–0.0031	0.0012
F2–C2	1.3341(4)	1.3356	0.8228	0.5128	1.88(5)	–16.1(3)				
F3–C3	1.3312(4)	1.3333	0.8628	0.4705	1.93(6)	–19.1(3)				
C1–C2	1.3911(2)	1.3939	0.6675	0.7263	2.32(5)	–23.3(2)				
C1–C3	1.3930(2)	1.3944	0.6699	0.7245	2.09(5)	–21.2(2)				

i = 2–*x*, –*y*, –*z*

3.7 Residual electron density distribution in 4-9

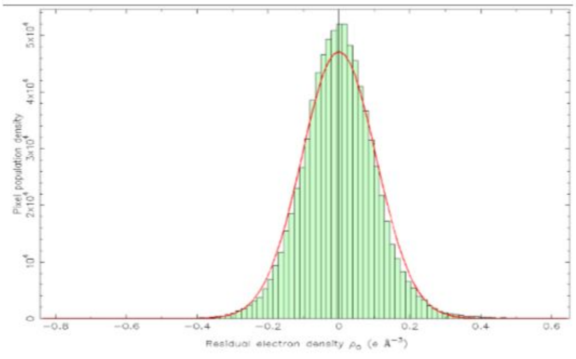

4

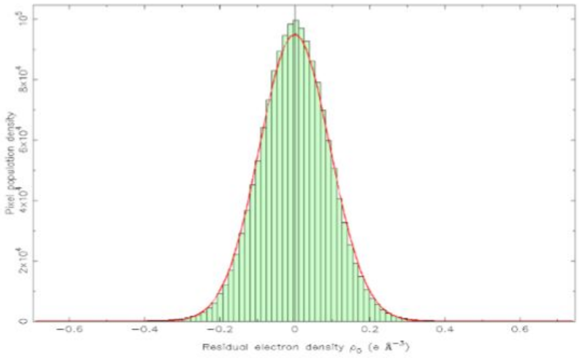

5

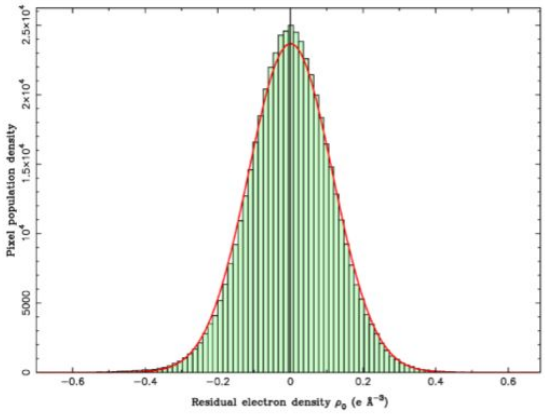

6

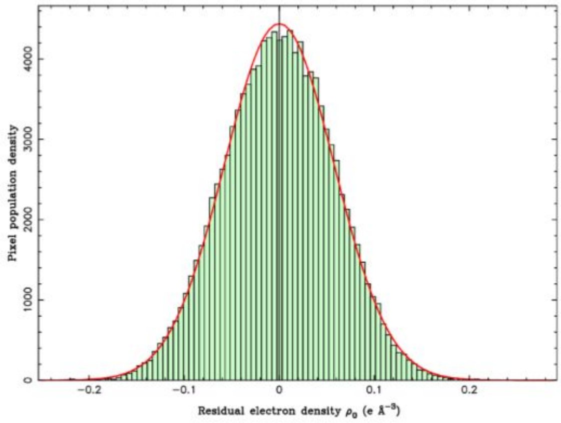

7

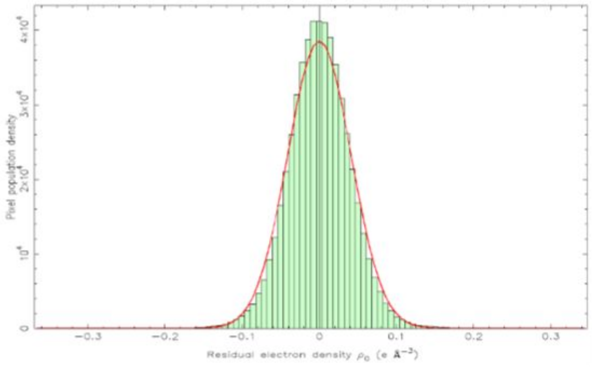

8

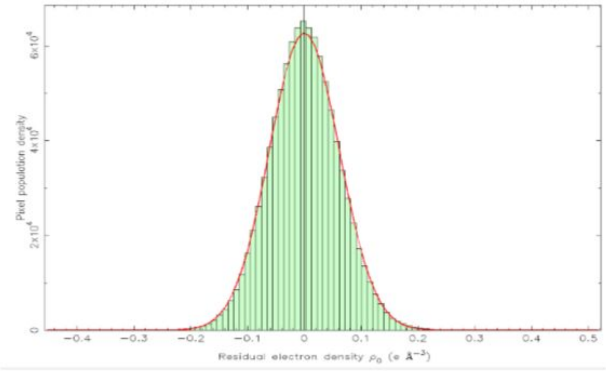

9

Figure S13: Probabillity distribution histograms of the residual electron density of the XB donors **4**-**6** and the XB adducts **7**-**9**.

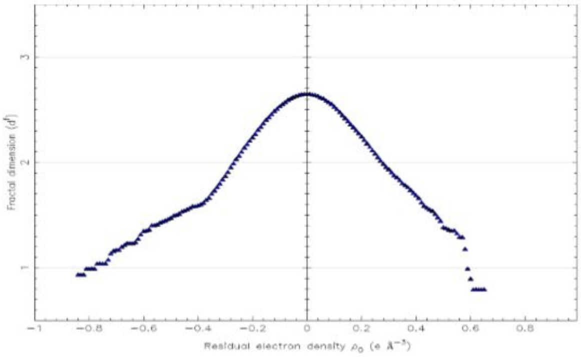

4

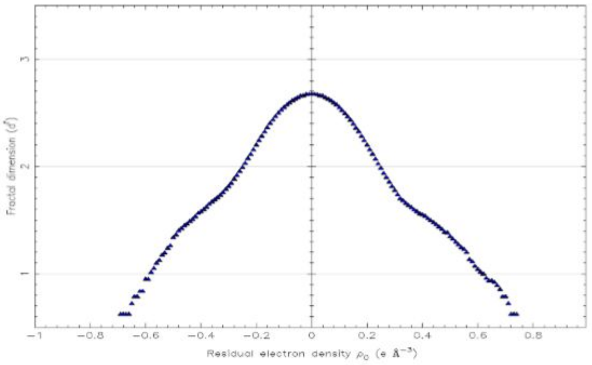

5

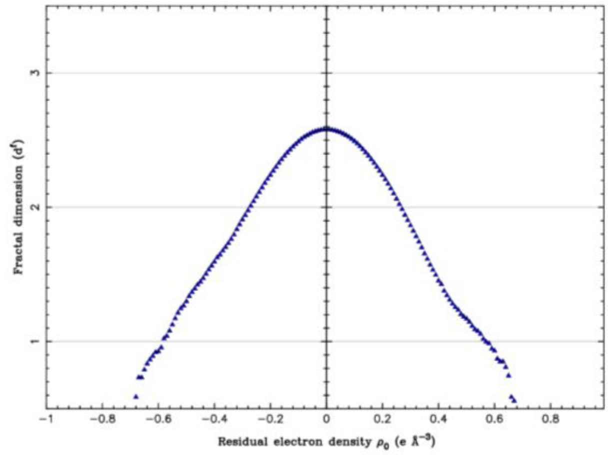

6

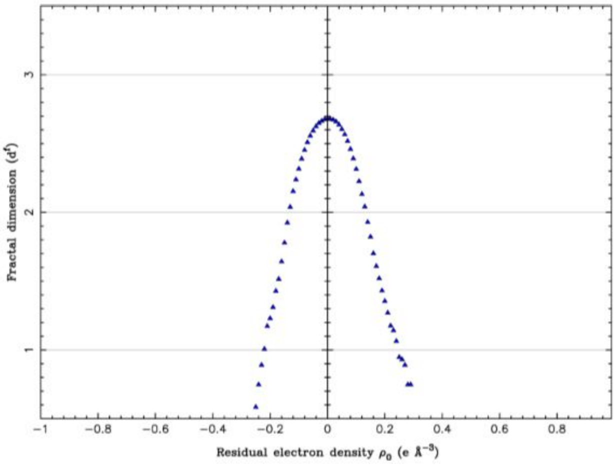

7

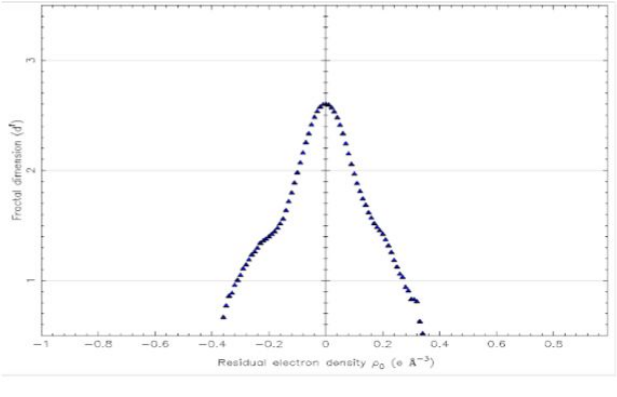

8

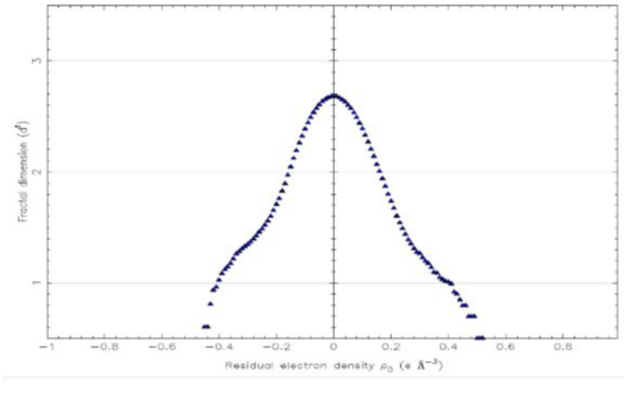

9

Figure S14: Fractal dimension plots of residual electron density of the XB donors **4**-**6** and the XB adducts **7**-**9**.

4 Simplified analysis of pairwise interactions in the crystal

Interaction energies for the halogen-bonded aggregates were calculated with the help of CrystalExplorer [11, 12]. The "fast" energy model [13] was used.

Electrostatic contribution to the halogen bond in **3**

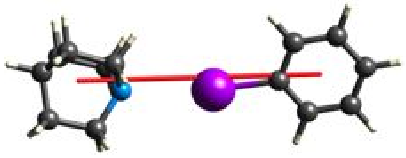

Electrostatic contribution to the halogen bond in **7**

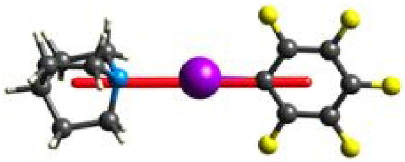

Electrostatic contribution to the halogen bond in **8**

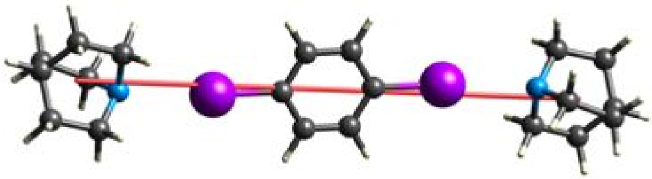

Electrostatic contribution to the symmetrically independent halogen bonds in **9**

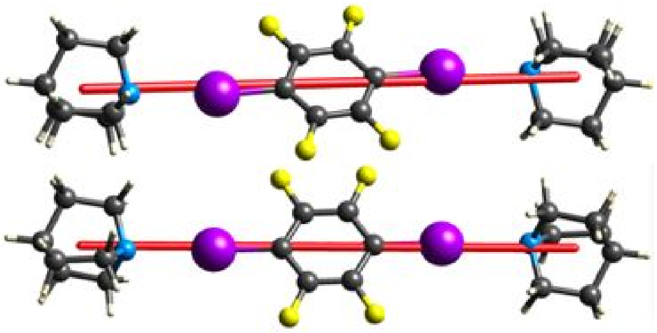

Figure S15: Electrostatic contributions to the halogen bonds in the adducts **3**, **7**, **8** and **9**; the radius of the red cylinder is proportional to the electrostatic interaction energy.

Table S31: Pairwise interaction energies [kJ·mol^{-1}] for the XB adducts **3**, **7**, **8** and **9**.

Comp		$E_{electrostatic}$	$E_{polarization}$	$E_{dispersion}$	$E_{repulsion}$	E_{total}
**3**		–38.3	–6.5	–17.3	54.9	–14.3
**7**		–88.3	–15.8	–19.8	108.9	–29.8
**8**		–37.1	–6.3	–16.8	48.5	–17.7
**9**	1st aggregate	–78.6	–13.9	–19.5	95.2	–29.5
**9**	2nd aggregate	–72.5	–13.1	–19.0	84.5	–31.1

5 Quantum Chemical Calculations

All quantum-chemical calculations were performed with *Gaussian16 Rev. B01* [14] or *Orca 4.2.0-shared.* [15, 16] First, calculations with the functional B3LYP [17, 18] and M062X were performed (*Gaussian16*). Starting from the atomic coordinates of the crystal structure, geometry optimizations and frequency calculations for the reactants and the XB-adduct were performed. Different basis sets [taken from the basic set exchange (BSE)] [19] were chosen and the B3LYP calculations were used both with and without GRIMME's dispersion correction (gd3) [20].

Table S32: Calculated bond lengths and the SCF, ZPE and GIBBS energy Δ*G* for pentafluoroiodobenzene **5**. Calculations were made with *Gaussian16*.

method	basis set	SCF [hartree]	ZPE [hartree]	ΔG [hartree]	C–I [Å]
B3LYP gd3	def2-QZVP	–1,025.95260298	–1,025.90337100	–1,025.94108700	2.08963
B3LYP gd3	def2-QZVPD	–1,025.95314440	–1,025.90391300	–1,025.94162900	2.08965
B3LYP gd3	def2-QZVPP	–1,025.95260298	–1,025.90337100	–1,025.94108700	2.08963
B3LYP gd3	def2-SVP	–1,025.03734198	–1,024.98798900	–1,025.02582200	2.10568
B3LYP gd3	def2-SVPD	–1,025.09184931	–1,025.04220000	–1,025.07989300	2.10708
B3LYP gd3	def2-TZVP	–1,025.91136880	–1,025.86216100	–1,025.89989600	2.0918
B3LYP gd3	def2-TZVPD	–1,025.91563391	–1,025.86635600	–1,025.90407500	2.0918
B3LYP gd3	def2-TZVPP	–1,025.91136880	–1,025.86216100	–1,025.89989600	2.0918
B3LYP gd3	LanL2DZ	–739.16590638	–739.11754300	–739.15548300	2.10705
B3LYP gd3	LanL2DZDP	–739.35397848	–739.30483900	–739.34264500	2.08785
B3LYP	def2-QZVP	–1,025.94248829	–1,025.89321700	–1,025.93094200	2.08713
B3LYP	def2-QZVPD	–1,025.94302955	–1,025.89375900	–1,025.93148300	2.08715
B3LYP	def2-QZVPP	–1,025.94248829	–1,025.89321700	–1,025.93094200	2.08713
B3LYP	def2-SVP	–1,025.02715809	–1,024.97776800	–1,025.01561100	2.10321
B3LYP	def2-SVPD	–1,025.08165776	–1,025.03196900	–1,025.06967300	2.10461
B3LYP	def2-TZVP	–1,025.90124666	–1,025.85200000	–1,025.88974400	2.08932
B3LYP	def2-TZVPD	–1,025.90551105	–1,025.85619400	–1,025.89392100	2.08931
B3LYP	def2-TZVPP	–1,025.90124666	–1,025.85200000	–1,025.88974400	2.08932
B3LYP	LanL2DZ	–739.15557409	–739.10716200	–739.14511000	2.10463
B3LYP	LanL2DZDP	–739.34377852	–739.29459800	–739.33241400	2.08547
M062X	def2-QZVP	–1,025.53665773	–1,025.48597500	–1,025.52353200	2.06663
M062X	def2-QZVPD	–1,025.53717721	–1,025.48649300	–1,025.52404800	2.06664
M062X	def2-QZVPP	–1,025.53665773	–1,025.48597500	–1,025.52353200	2.06663
M062X	def2-SVP	–1,024.61397324	–1,024.56327000	–1,024.60094700	2.08132
M062X	def2-SVPD	–1,024.66305101	–1,024.61211100	–1,024.64971800	2.0819
M062X	def2-TZVP	–1,025.48475651	–1,025.43411300	–1,025.47170000	2.06813
M062X	def2-TZVPD	–1,025.48847070	–1,025.43778800	–1,025.47536700	2.06818
M062X	def2-TZVPP	–1,025.48475651	–1,025.43411300	–1,025.47170000	2.06813

Based on the theoretical calculation of XB adducts 3 and 7, only selected methods were chosen for the calculations of XB adducts 8 and 9. The methods that approximately described the geometric structure of the crystal structures 3 and 7 were used.

Table S33: Calculated bond lengths and the SCF, ZPE and GIBBS energy Δ*G* for diiodobenzene **4**. Calculations were made with *Gaussian16*.

method	basis set	SCF [hartree]	ZPE [hartree]	ΔG [hartree]	C–I [Å]
B3LYP gd3	def2-QZVP	–826.75856351	–826.67917100	–826.71499100	2.11071
B3LYP gd3	def2-QZVPD	–826.75868740	–826.67929000	–826.71510900	2.11071
B3LYP gd3	def2-QZVPP	–826.75856351	–826.67917100	–826.71499100	2.11071
B3LYP gd3	def2-SVP	–826.46862485	–826.38916300	–826.42499400	2.12735
B3LYP gd3	def2-SVPD	–826.48221958	–826.40267000	–826.43847100	2.12802
B3LYP gd3	def2-TZVP	–826.74073635	–826.66140600	–826.69723200	2.11315
B3LYP gd3	def2-TZVPD	–826.74156393	–826.66217200	–826.69799800	2.11313
B3LYP gd3	def2-TZVPP	–826.74254467	–826.66308400	–826.69890400	2.11316
B3LYP gd3	LanL2DZ	–253.77487227	–253.69484900	–253.73070100	2.14344
B3LYP gd3	LanL2DZDP	–253.84910755	–253.77003800	–253.80588700	2.10852
M062X	def2-QZVP	–826.30958656	–826.22941600	–826.26500800	2.08671
M062X	def2-QZVPD	–826.30972205	-826.22932000	–826.26510900	2.0866
M062X	def2-QZVPP	–826.30958656	–825.93577900	–826.26500800	2.08671
M062X	def2-SVP	–826.01608390	–825.94678800	–825.97147900	2.10222
M062X	def2-SVPD	–826.02688913	–826.20779800	–825.98247600	2.10282
M062X	def2-TZVP	–826.28803607	–826.20862300	–826.24348300	2.08869
M062X	def2-TZVPD	–826.28883432	–826.20934500	–826.24431700	2.08882
M062X	def2-TZVPP	–826.28967446	–826.22932000	–826.24502500	2.08862

Table S34: Calculated bond lengths and the SCF, ZPE and GIBBS energy Δ*G* for 1,4-diiodotetrafluorobenzene **6**. Calculations were made with *Gaussian16*.

method	basis set	SCF [hartree]	ZPE [hartree]	ΔG [hartree]	C–I [Å]
B3LYP gd3	def2-QZVP	–1,223.87501670	–1,223.82840000	–1,223.86876900	2.0882
B3LYP gd3	def2-QZVPD	–1,223.87545377	–1,223.82883800	–1,223.86920600	2.08821
B3LYP gd3	def2-QZVPP	–1,223.87501670	–1,223.82840000	–1,223.86876900	2.0882
B3LYP gd3	def2-SVP	–1,223.07812475	–1,223.03105200	–1,223.07145100	2.10465
B3LYP gd3	def2-SVPD	–1,223.12380214	–1,223.07673700	–1,223.11707400	2.10553
B3LYP gd3	def2-TZVP	–1,223.83816212	–1,223.79151700	–1,223.83189400	2.09036
B3LYP gd3	def2-TZVPD	–1,223.84158659	–1,223.79491300	–1,223.83528500	2.09036
B3LYP gd3	def2-TZVPP	–1,223.83816212	–1,223.79151700	–1,223.83189400	2.09036
B3LYP gd3	LanL2DZ	–650.71379818	–650.66777100	–650.70826500	2.10739
B3LYP gd3	LanL2DZDP	–650.88315807	–650.83668500	–650.87712600	2.08657
M062X	def2-QZVP	–1,223.31979106	–1,223.27177700	–1,223.31196400	2.06526
M062X	def2-QZVPD	–1,223.32023286	–1,223.27221400	–1,223.31240000	2.06527
M062X	def2-QZVPP	–1,223.31979106	–1,223.27177700	–1,223.31196400	2.06526
M062X	def2-SVP	–1,222.51806435	–1,222.46962400	–1,222.50981600	2.0804
M062X	def2-SVPD	–1,222.55869267	–1,222.51035600	–1,222.55055400	2.07946
M062X	def2-TZVP	–1,223.27389360	–1,223.22587500	–1,223.26608100	2.06687
M062X	def2-TZVPD	–1,223.27704107	–1,223.22900300	–1,223.26920800	2.0669
M062X	def2-TZVPP	–1,223.27389360	–1,223.22587500	–1,223.26608100	2.06687

Table S35: Calculated SCF, ZPE and GIBBS energy Δ*G* for quinuclidine **1**. Calculations were made with *Gaussian16*.

method	basis set	SCF [hartree]	ZPE [hartree]	ΔG [hartree]
B3LYP gd3	def2-QZVP	–329.47205265	–329.27751400	–329.30829200
B3LYP gd3	def2-QZVPD	–329.47217989	–329.27764400	–329.30842000
B3LYP gd3	def2-QZVPP	–329.47205265	–329.27751400	–329.30829200
B3LYP gd3	def2-SVP	–329.09019147	–328.89551800	–328.92593400
B3LYP gd3	def2-SVPD	–329.11224893	–328.91788200	–328.94927700
B3LYP gd3	def2-TZVP	–329.44648648	–329.25177900	–329.28256400
B3LYP gd3	def2-TZVPD	–329.44769585	–329.25306400	–329.28385400
B3LYP gd3	def2-TZVPP	–329.45143491	–329.25672200	–329.28747800
B3LYP gd3	LanL2DZ	–329.26863658	–329.07263400	–329.10344300
B3LYP gd3	LanL2DZDP	–329.37283474	–329.17855000	–329.20958200
B3LYP	def2-QZVP	–329.45448972	–329.26018000	–329.29081700
B3LYP	def2-QZVPD	–329.45461776	–329.26031100	–329.29094600
B3LYP	def2-QZVPP	–329.45448972	–329.26018000	–329.29081700
B3LYP	def2-SVP	–329.07265104	–328.87825000	–328.90861900
B3LYP	def2-SVPD	–329.09469514	–328.90055000	–328.93142400
B3LYP	def2-TZVP	–329.42893196	–329.23446600	–329.26509200
B3LYP	def2-TZVPD	–329.43013705	–329.23574200	–329.26636300
B3LYP	def2-TZVPP	–329.43388133	–329.23942100	–329.27005400
B3LYP	LanL2DZ	–329.25104666	–329.05528300	–329.08597000
B3LYP	LanL2DZDP	–329.35527136	–329.16120800	–329.19197100
M062X	def2-QZVP	–329.30703197	–329.11056500	–329.14107600
M062X	def2-QZVPD	–329.30716691	–329.11071800	–329.14123700
M062X	def2-QZVPP	–329.30702874	–329.11056400	–329.14106600
M062X	def2-SVP	–328.90820851	–328.71210900	–328.74309800
M062X	def2-SVPD	–328.92865146	–328.73250800	–328.76298000
M062X	def2-TZVP	–329.27448792	–329.07789600	–329.10842000
M062X	def2-TZVPD	–329.27564796	–329.07916500	–329.10970900
M062X	def2-TZVPP	–329.27962434	–329.08299800	–329.11350600

Table S36: Calculated SCF, ZPE and GIBBS energy Δ*G* for XB adduct **7**. Calculations were made with *Gaussian16*.

method	basis set	SCF [hartree]	ZPE [hartree]	ΔG [hartree]
B3LYP gd3	def2-QZVP	–1,355.44207017	–1,355.19701900	–1,355.24890300
B3LYP gd3	def2-QZVPD	–1,355.44272010	–1,355.19767200	–1,355.24954500
B3LYP gd3	def2-QZVPP	–1,355.44207017	–1,355.19701900	–1,355.24890300
B3LYP gd3	def2-SVP	–1,354.14837966	–1,353.90302700	–1,353.95457200
B3LYP gd3	def2-SVPD	–1,354.22454531	–1,353.97922100	–1,354.03189000
B3LYP gd3	def2-TZVP	–1,355.37540755	–1,355.13023500	–1,355.18220300
B3LYP gd3	def2-TZVPD	–1,355.38069662	–1,355.13553300	–1,355.18745400
B3LYP gd3	def2-TZVPP	–1,355.38033656	–1,355.13514700	–1,355.18706000
B3LYP gd3	LanL2DZ	–1,068.45896665	–1,068.21313200	–1,068.26482200
B3LYP gd3	LanL2DZDP	–1,068.74590734	–1,068.50109700	–1,068.55327800
B3LYP	def2-QZVP	–1,355.40721057	–1,355.16248800	–1,355.21446800
B3LYP	def2-QZVPD	–1,355.40785764	–1,355.16313800	–1,355.21511300
B3LYP	def2-QZVPP	–1,355.40721057	–1,355.16248800	–1,355.21446800
B3LYP	def2-SVP	–1,354.11344749	–1,353.86843100	–1,353.92020700
B3LYP	def2-SVPD	–1,354.18946503	–1,353.94443800	–1,353.99647100
B3LYP	def2-TZVP	–1,355.34059310	–1,355.09574500	–1,355.14780800
B3LYP	def2-TZVPD	–1,355.34585758	–1,355.10101900	–1,355.15303400
B3LYP	def2-TZVPP	–1,355.34551827	–1,355.10065600	–1,355.15269600
B3LYP	LanL2DZ	–1,068.42363346	–1,068.17811800	–1,068.22985800
B3LYP	LanL2DZDP	–1,068.71100172	–1,068.46651700	–1,068.51870100
M062X	def2-QZVP	–1,354.85853639	–1,354.61035800	–1,354.66215400
M062X	def2-QZVPD	–1,354.85918126	–1,354.61100600	–1,354.66278300
M062X	def2-QZVPP	–1,354.85853639	–1,354.61035800	–1,354.66215400
M062X	def2-SVP	–1,353.54062870	–1,353.29274900	–1,353.34487400
M062X	def2-SVPD	–1,353.60956005	–1,353.36143500	–1,353.41303400
M062X	def2-TZVP	–1,354.77439976	–1,354.52614900	–1,354.57820100
M062X	def2-TZVPD	–1,354.77909291	–1,354.53090600	–1,354.58297100
M062X	def2-TZVPP	–1,354.77943291	–1,354.53115200	–1,354.58323000

Table S37: Calculated SCF, ZPE and GIBBS energy Δ*G* for XB adduct **8**. Calculations were made with *Gaussian16*.

method	basis set	SCF [hartree]	ZPE [hartree]	ΔG [hartree]
B3LYP gd3	def2-QZVP	–1,485.72469029	–1,485.25441600	–1,485.32303600
B3LYP gd3	def2-SVP	–1,484.67828891	–1,484.20741300	–1,484.27253500
M062X	def2-QZVP	–1,484.94212725	–1,484.46753600	–1,484.53487900
M062X	def2-SVP	–1,483.85791014	–1,483.38360400	–1,483.44958300
M062X	def2-SVPD	–1,483.90746691	–1,483.43358300	–1,483.50062400

Table S38: Calculated SCF, ZPE and GIBBS energy Δ*G* for XB adduct **9**. Calculations were made with *Gaussian16*.

method	basis set	SCF [hartree]	ZPE [hartree]	ΔG [hartree]
B3LYP gd3	def2-SVPD	–1,881.38656514	–1,880.94829000	–1,881.02055200
M062X	def2-SVPD	–1,880.44965515	–1,880.00703600	–1,880.07515100

Table S39: Calculated bond lengths and the GIBBS energy Δ*G* (in kJ·mol⁻¹) for the XB-adduct **7**. The bond lengths from the solid structure are also given. Calculations were made with *Gaussian16*.

	**exp. data**	**2.1295**	**2.6781**	
**method**	**basis set**	**C–I [Å]**	**N---I [Å]**	**ΔG [kJ·mol⁻¹]**
B3LYP gd3	def2-QZVP	2.13538	2.76685	1.25
B3LYP gd3	def2-QZVPD	2.13554	2.76616	1.32
B3LYP gd3	def2-QZVPP	2.13538	2.76685	1.25
B3LYP gd3	def2-SVP	2.14968	2.76707	–7.39
B3LYP gd3	def2-SVPD	2.15592	2.73341	–7.14
B3LYP gd3	def2-TZVP	2.1362	2.77812	0.68
B3LYP gd3	def2-TZVPD	2.13678	2.77272	1.25
B3LYP gd3	def2-TZVPP	2.13624	2.77786	0.82
B3LYP gd3	LanL2DZ	2.17752	2.68722	–15.48
B3LYP gd3	LanL2DZDP	2.13264	2.76538	–2.76
B3LYP	def2-QZVP	2.12718	2.83381	19.14
B3LYP	def2-QZVPD	2.12733	2.83299	19.21
B3LYP	def2-QZVPP	2.12718	2.83381	19.14
B3LYP	def2-SVP	2.14254	2.82513	10.56
B3LYP	def2-SVPD	2.14878	2.7851	12.15
B3LYP	def2-TZVP	2.12799	2.84798	18.45
B3LYP	def2-TZVPD	2.12861	2.84106	19.04
B3LYP	def2-TZVPP	2.12806	2.84738	18.65
B3LYP	LanL2DZ	2.16983	2.7301	3.21
B3LYP	LanL2DZDP	2.12478	2.82991	14.92
M062X	def2-QZVP	2.10194	2.75947	6.44
M062X	def2-QZVPD	2.10203	2.75882	6.57
M062X	def2-QZVPP	2.10194	2.75947	6.42
M062X	def2-SVP	2.11551	2.76254	–2.18
M062X	def2-SVPD	2.11889	2.73032	–0.88
M062X	def2-TZVP	2.10258	2.77207	5.04
M062X	def2-TZVPD	2.10336	2.76414	5.53
M062X	def2-TZVPP	2.10245	2.77309	5.19

Table S40: Calculated bond lengths and the GIBBS energy Δ*G* (in kJ·mol⁻¹) for the XB-adduct **8**. The bond lengths from the solid structure are also given. Calculations were made with *Gaussian16*.

	**exp. data**	**2.1096**	**2.9568**	
**method**	**basis set**	**C–I [Å]**	**N---I [Å]**	**ΔG [kJ·mol⁻¹]**
B3LYP gd3	def2-QZVP	2.12716	**2.94533**	22.42
B3LYP gd3	def2-SVP	2.14465	**2.92062**	11.36
M062X	def2-QZVP	2.09822	**2.95210**	299.49
M062X	def2-SVP	2.11451	**2.92975**	2110.98
M062X	def2-SVPD	2.11419	**2.91927**	–83.89

Table S41: Calculated bond lengths and the GIBBS energy Δ*G* (in kJ·mol⁻¹) for the XB-adduct **9**. The bond lengths from the solid structure are also given. Calculations were made with *Gaussian16*.

	**exp. data**	**2.1142**<br>**2.1189**	**2.7173**<br>**2.7476**	
**method**	**basis set**	**C–I [Å]**	**N---I [Å]**	**ΔG [kJ·mol⁻¹]**
B3LYP gd3	def2-SVPD	2.14801	**2.7694**	–12.93
M062X	def2-SVPD	2.11241	**2.76979**	–100.82

In a second step calculations with orca were carried out. At first structural optimizations with DFT-calculations and RI-MP2 with the basis set def2-TZVPP were performed for the reactants and the XB-adduct. Single point calculations were then performed based on RI-MP2/def2-TZVPP structures. The following combinations were computed: RI-MP2/aug-cc-pVQZ (for iodine aug-cc-pwCVQZ-PP)

and CCSD(T)/def2-TZVPP. A weighted core valence basis set has been chosen for iodine in order to take correlation effects the 4d-shell electrons exactly into account. Based on these two calculations, the energy value *E* for CCSD(T)/aug-cc-pVQZ was approximately determined using the following equation (E1): [21]

$$E_{\text{CCSD(T)/aug-cc-pVQZ}} \approx E_{\text{CCSD(T)/def2-TZVPP}} + (E_{\text{RI-MP2/aug-cc-pVQZ}} - E_{\text{RI-MP2/def2-TZVPP}}) \quad \text{(E1)}$$

To determine the reaction GIBBS energy $G_{reaction}$, the molar enthalpy *H* and GIBBS energy *G* were first calculated by using the thermodynamic values *U* and Sfrom the BP86 calculations. The following two equations E2 and E3 were used:

$$H = U + R \cdot T \quad \text{(E2)}$$
$$G = H - T \cdot S \quad \text{(E3)}$$

Table S42: Calculated electronic energies *E* at 298.15 K and 1 bar pressure for pentafluoroiodobenzene **5**, quinuclidine **1** and the XB-adduct **7**. Calculations were made with *Orca*.

		***E* [hartree]**		
**method**	**basis set**	**Pentafluoroiodobenzene**	**Quinuclidine**	**XB-adduct 7**
BP86	def2-TZVPP	–1,025.968147	–329.4246509	–1,355.405258
B3LYP D3-BJ	def2-TZVPP	–1,025.528326	–329.2432751	–1,354.790376
PBE0	def2-TZVPP	–1,025.093141	–329.0357725	–1,354.141470
RI-MP2	def2-TZVPP	–1,023.928213	–328.5971214	–1,352.544739
SP-RI-MP2	aug-cc-pVQZ[a]	–1,023.995796	–328.7232117	–1,352.736151
SP-CCSD(T)	def2-TZVPP	–1,022.448462	–328.7095979	–1,351.176457
Approx. CCSD(T)	aug-cc-pVQZ[a]	–1,022.516045	–328.8356883	–1,351.367869

[a]aug-cc-pwCVQZ-PP was choosen for iodine.

Table S43: Calculated Internal Energies *U* as well as Entropy Sat 298.15 K and 1 bar pressure for pentafluoroiodobenzene **5**, quinuclidine **1** and the XB-adduct **7**. Calculations were made with *Orca*.

	**method**	**basis set**	***U* [hartree]**	***TS* [hartree]**
Pentafluoroiodobenzene	BP86	def2-TZVPP	–1,025.909964	0.04924518
Quinuclidine	BP86	def2-TZVPP	–329.2291343	0.0381737
XB adduct **7**	BP86	def2-TZVPP	–1,355.148055	0.06847437

Table S44: Calculated bond lengths and the enthalpy $\Delta H_{reaction}$ as well as the GIBBS energy $\Delta G_{reaction}$ for the XB-adduct **7**. The bond lengths from the solid structure are also given. Calculations were made with *Orca*.

	**exp. data**	**2.1295**	**2.6781**		
**method**	**basis set**	**C–I [Å]**	**N---I [Å]**	**$\Delta H_{reaction}$ [kJ·mol^{-1}]**	**$\Delta G_{reaction}$ [kJ·mol^{-1}]**
BP86	def2-TZVPP	2.1458	2.7556	–58.71141165	–8.972600642
B3LYP D3-BJ	def2-TZVPP	2.1336	2.7446	–75.2898635	–25.55105254
PBE0	def2-TZVPP	2.1063	2.7660	–58.96500784	–9.226196834
RI-MP2	def2-TZVPP	2.0944	2.6904	–76.94305458	–27.20424358
SP-RI-MP2	aug-cc-pVQZ[a]	-	-	–71.00557524	–21.26676423
SP-CCSD(T)	def2-TZVPP	-	-	–74.29636415	–24.55755315
Approx. CCSD(T)	aug-cc-pVQZ[a]	-	-	–68.35888481	**–18.6200738**

[a]aug-cc-pwCVQZ-PP was choosen for iodine.

6 NMR Investigations in Solution

First, ^{1}H, ^{13}C and ^{15}N HMBC spectra of the two reactants and the mixture were measured in cyclohexane-d12. The ^{1}H NMR chemical shifts are reported relative to TMS, which was added to the NMR tube (5 µL) as reference (δ = 0.00 ppm).

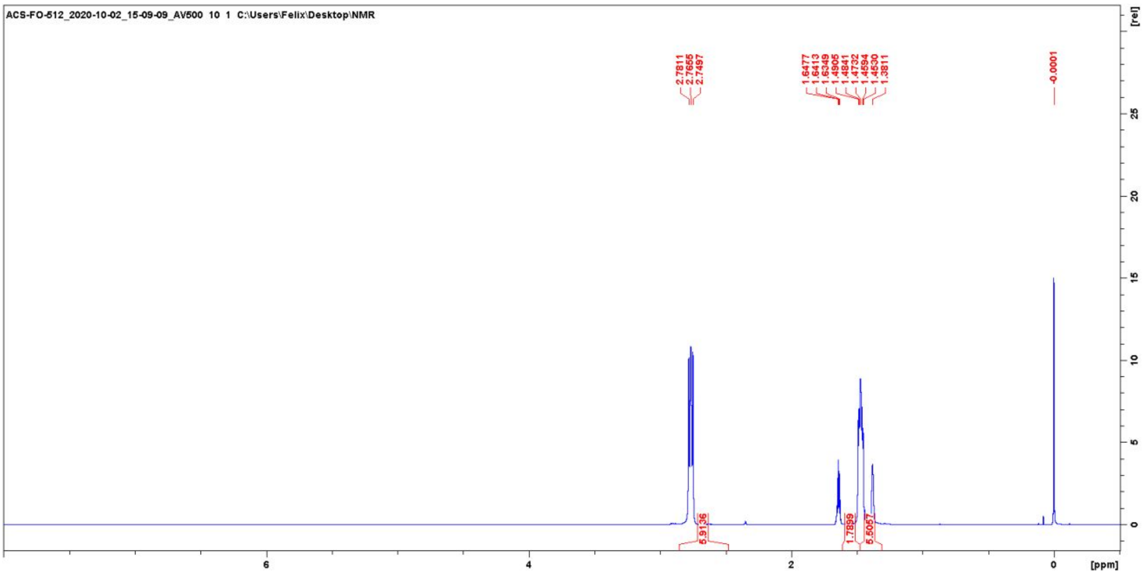

Figure S16: ^{1}H NMR spectra of quinuclidine in cyclohexane-d12. Chemical shifts are reported relative to TMS.

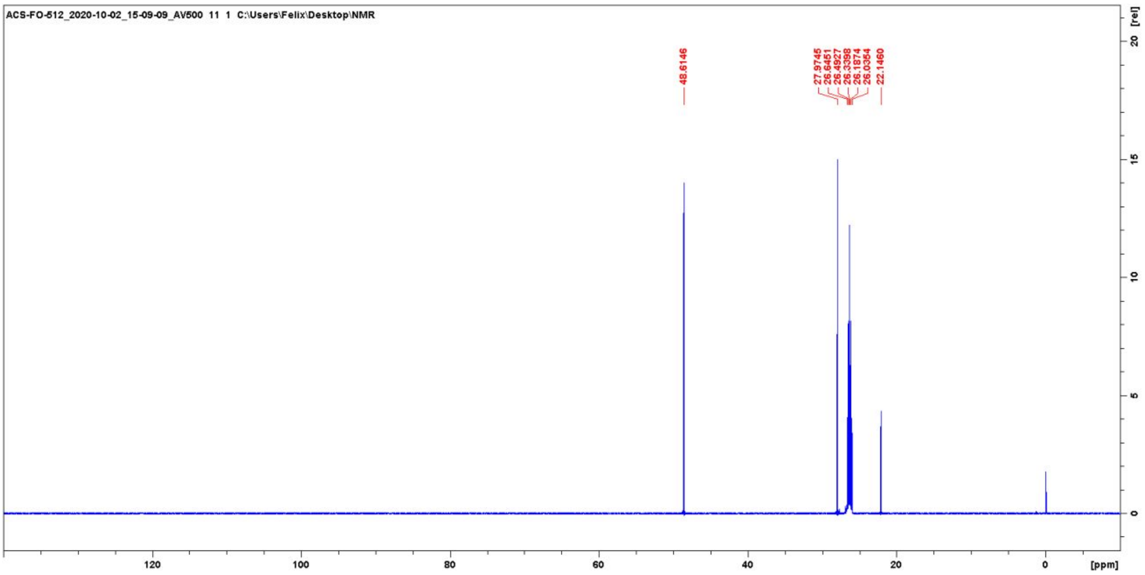

Figure S17: ^{13}C NMR spectra of quinuclidine in cyclohexane-d12. Chemical shifts are reported relative to TMS.

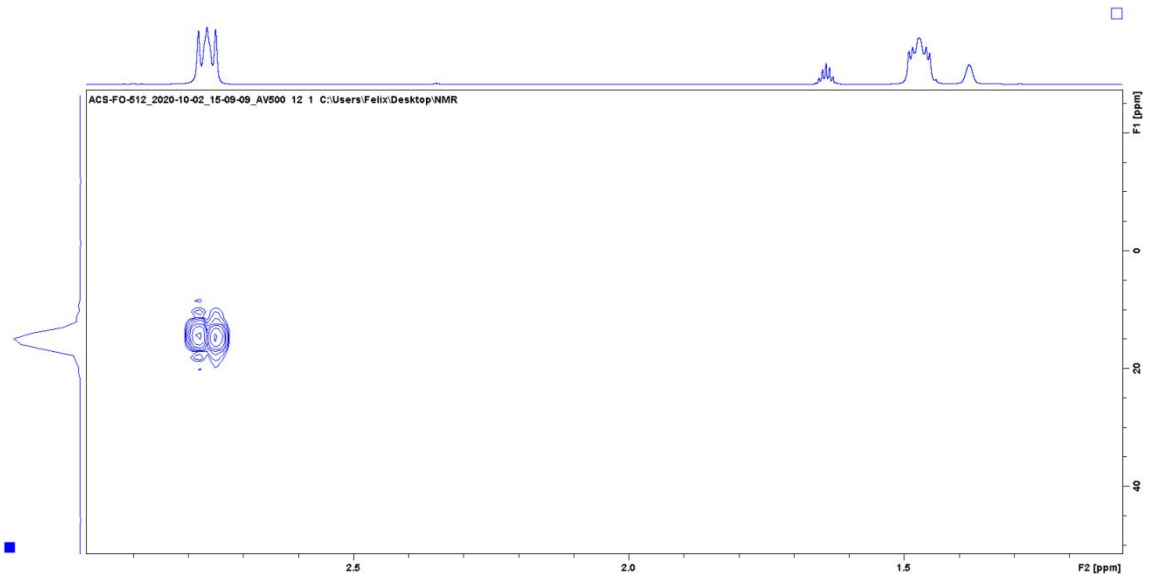

Figure S18: ^{15}N HMBC NMR spectra of quinuclidine in cyclohexane-d12.

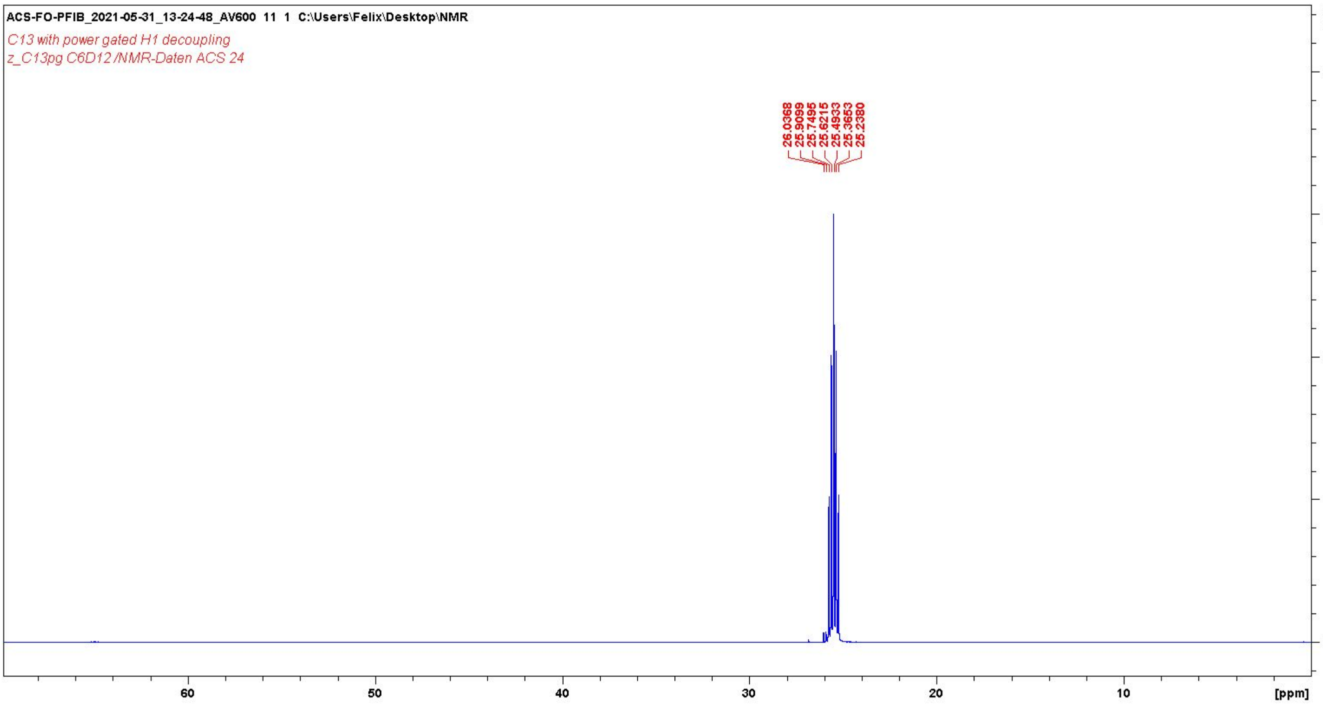

Figure S19: ^{13}C NMR spectra of pentafluoroiodobenzene in cyclohexane-d12. Chemical shifts are reported relative to TMS.

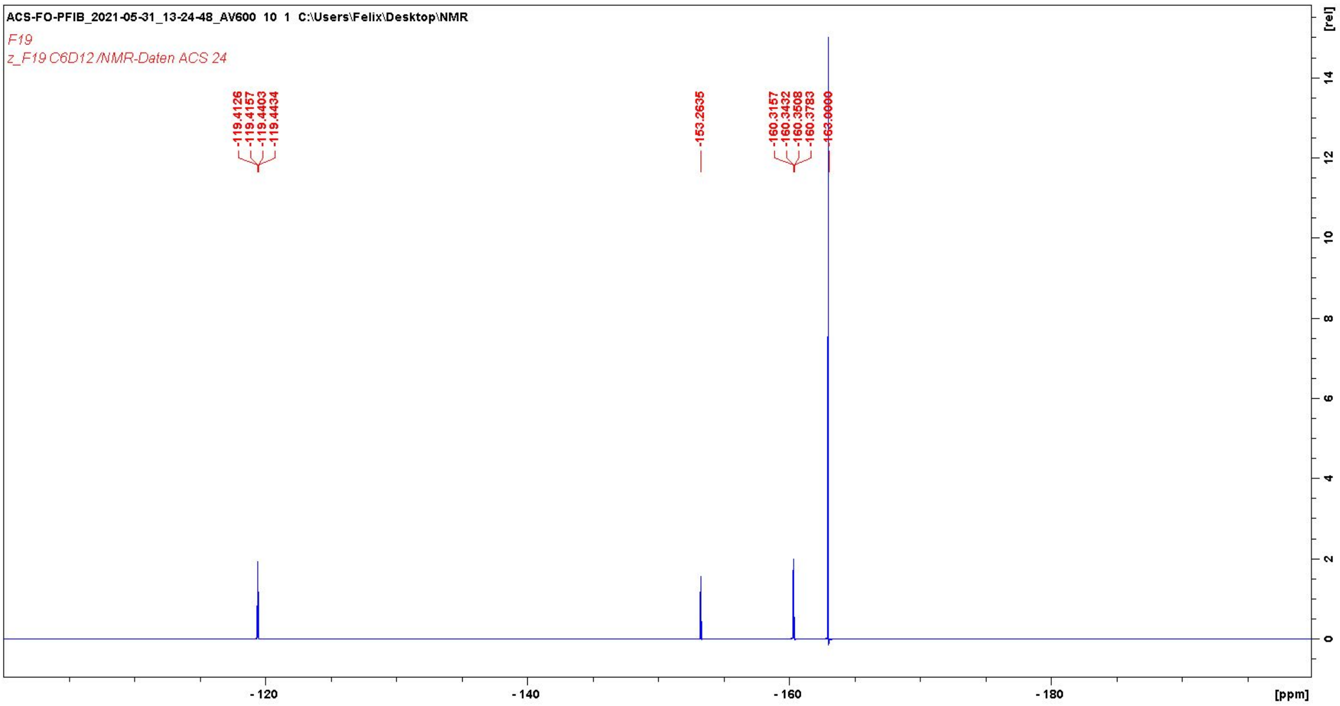

Figure S20: ^{19}F NMR spectra of pentafluoroiodobenzene in cyclohexane-d12. Chemical shifts are reported relative to hexafluorobenzene.

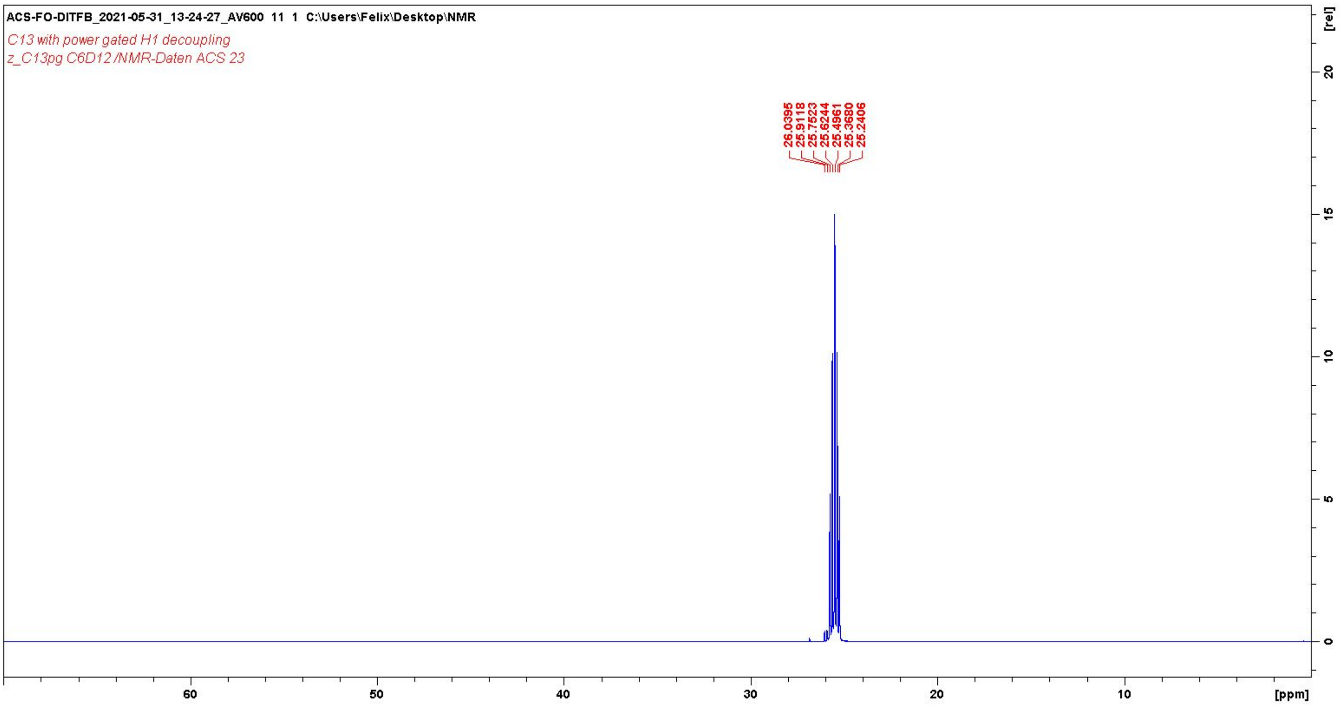

Figure S21: ^{13}C NMR spectra of 1,4-diiodotetrafluorobenzene in cyclohexane-d_{12}. Chemical shifts are reported relative to TMS.

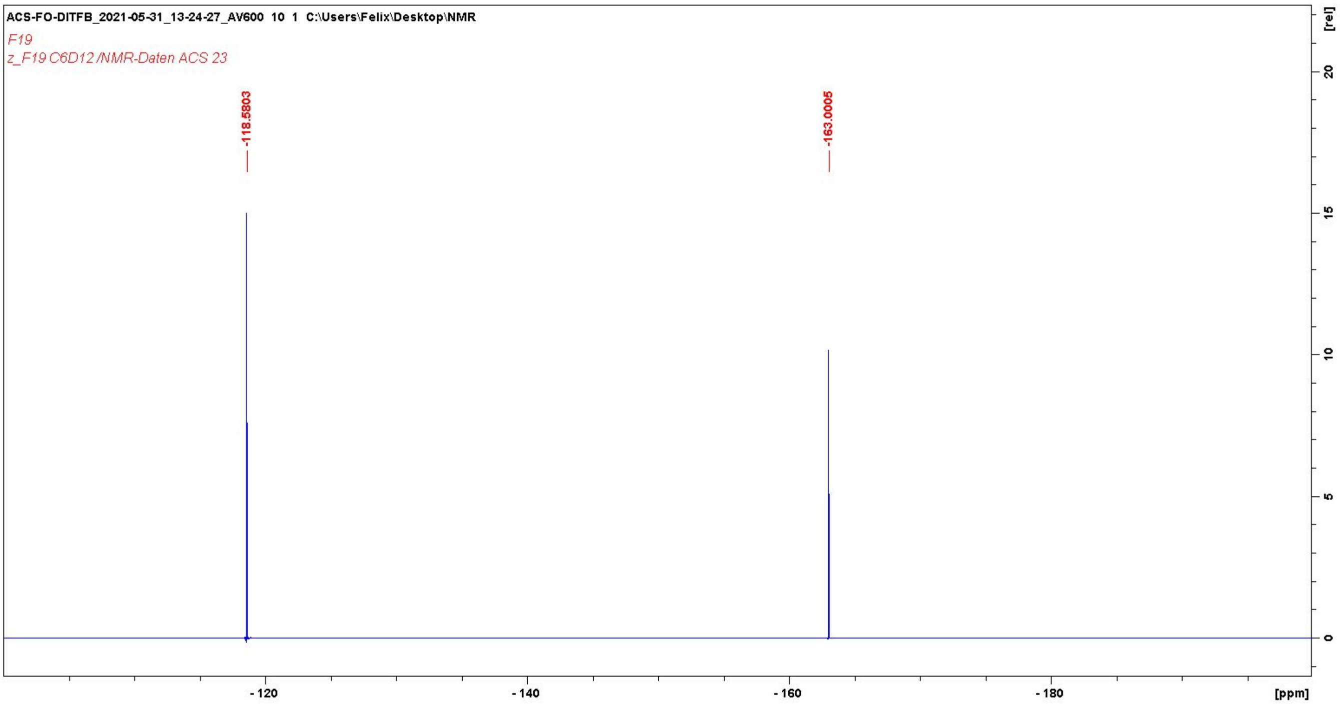

Figure S22: ^{19}F NMR spectra of 1,4-diiodotetrafluorobenzene in cyclohexane-d12. Chemical shifts are reported relative to hexafluorobenzene.

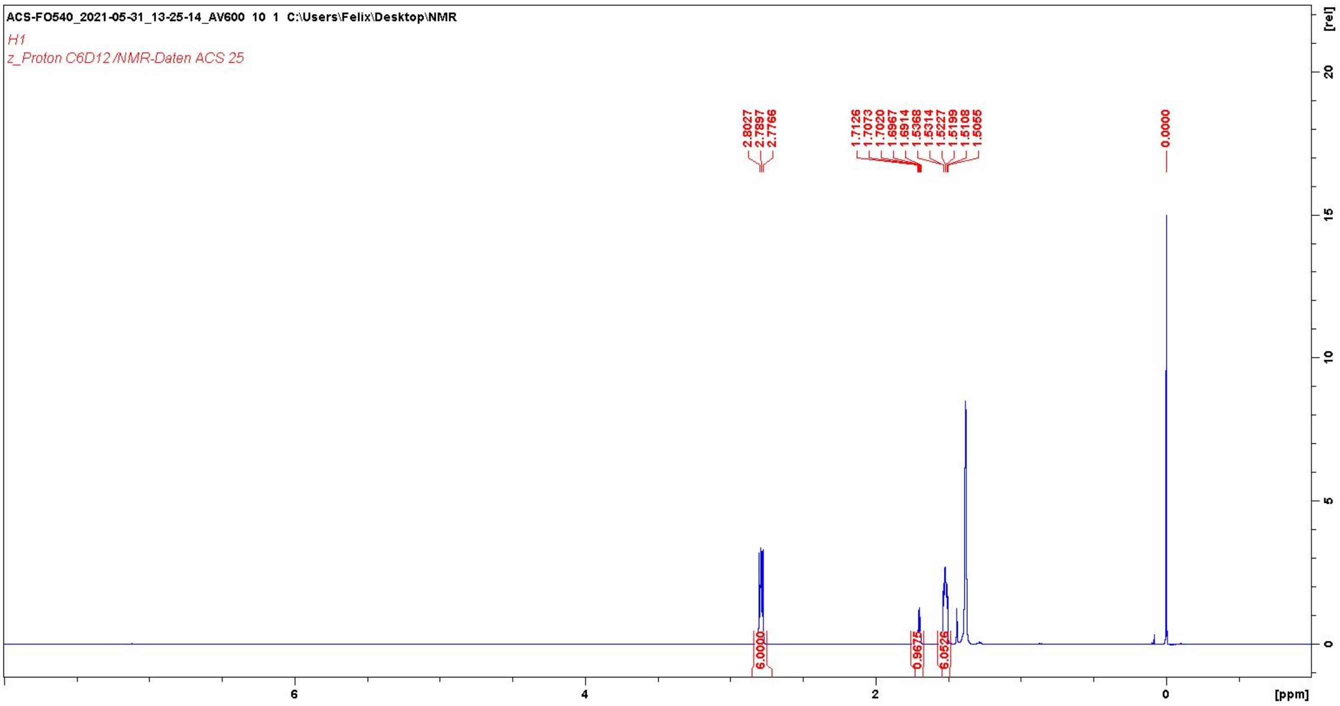

Figure S23: ^{1}H NMR spectra of quinuclidine and 1,4-diiodotetrafluorobenzene (ratio 1:5) in cyclohexane-d12. Chemical shifts are reported relative to TMS.

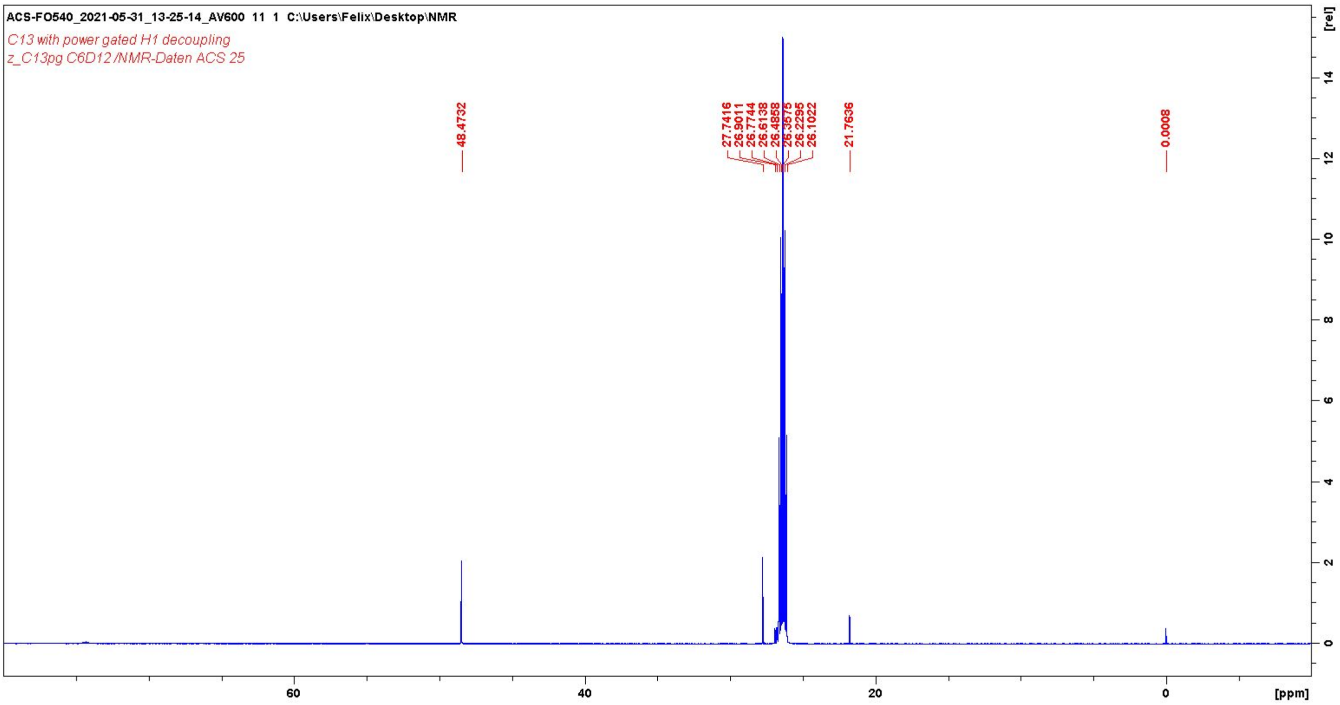

Figure S24: ^{13}C NMR spectra of quinuclidine and 1,4-diiodotetrafluorobenzene (ratio 1:5) in cyclohexane-d12. Chemical shifts are reported relative to TMS.

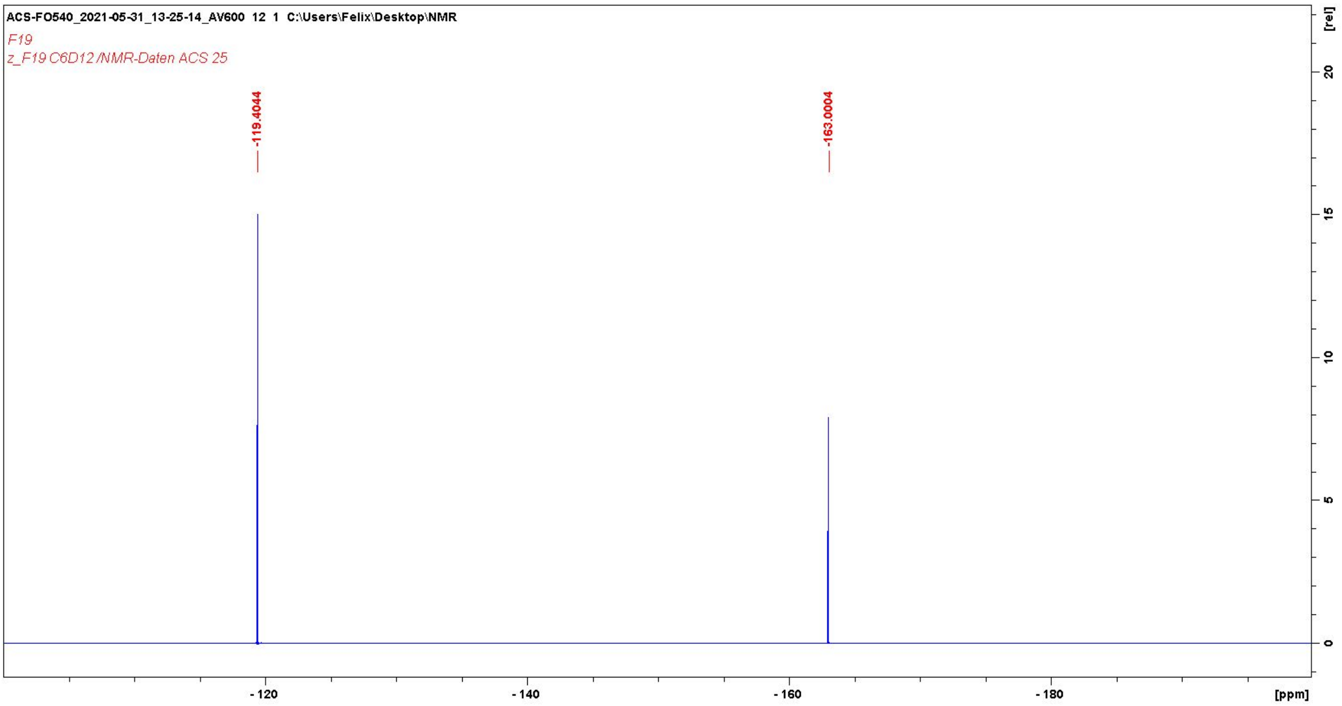

Figure S25: ^{19}F NMR spectra of quinuclidine and 1,4-diiodotetrafluorobenzene (ratio 1:5) in cyclohexane-d12. Chemical shifts are reported relative to hexafluorobenzene.

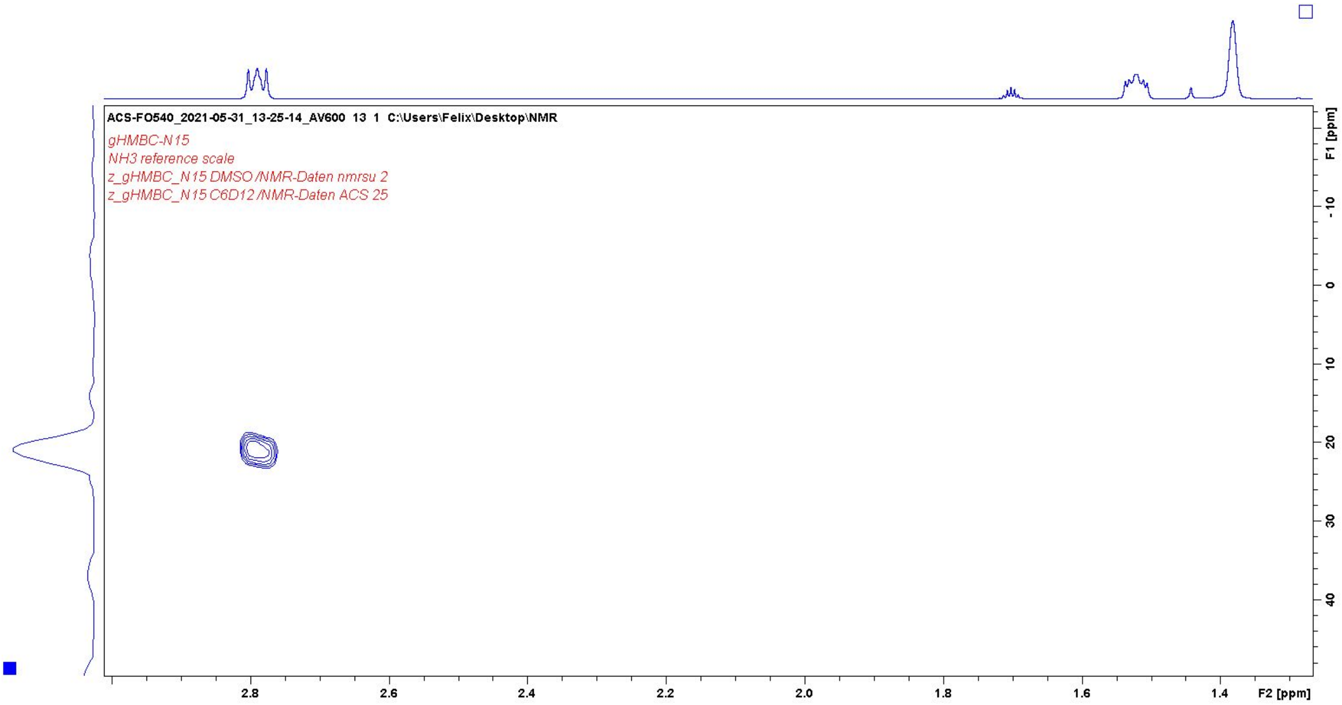

Figure S26: ^{15}N HMBC NMR spectra of quinuclidine and 1,4-diiodotetrafluorobenzene (ratio 1:5) in cyclohexane-d12. Chemical shifts are reported relative to TMS.

For the NMR titrations experiments a solution (~20mM) consisting of XB-donor or quinuclidine and C_6D_{12} as solvent was prepared. This mixture was then used to prepare a second solution as mixture of host and guest. This second solution has a ~20-fold concentration of the guest in relation to the host. The chemical which is kept constant is called 'host' during the titration experiments and the other component is called 'guest'. In the measurements performed, the NMR-active nucleus of the host was considered (here ^{1}H and ^{19}F, respectively). Due to the fast nature of the exchange between complex and free host, their signals cannot be separated and a complexation-induced shift (CIS) δ is observed. For the evaluation of the experimental data we orientated on Roger S. Macomber's work from 1992 [22]. The CIS is calculated according to the following equation E4.

$$\delta = \delta_h + \left(\frac{\delta_c - \delta_h}{2c_{0h}}\right) \cdot \left(B - \sqrt{B^2 - 4c_{0h}c_g}\right) \tag{E4}$$

In equation E4 the chemical shift of free host prior to the titration is given as δ_h. The saturated complex has the chemical shift δ_c. The concentrations of host c_{0h} and guest c_g are represented by the named variables.
The chemical shift of the saturated complex was calculated by a logarithmic fit of the experimental CIS against the concentration of guest using the RGP-function in Excel.

$$\delta = m \cdot \ln c_g + \delta_c \tag{E5}$$

B is defined as followed:

$$B = c_{0h} + c_g + \frac{1}{K} \tag{E6}$$

The value of interest is the stabilitiy constant *K* which can be determined as followed:

$$K = \frac{1}{\frac{-c_{0h}(\delta_h - \delta)}{\delta_c - \delta_h} - \frac{c_g(\delta_c - \delta_h)}{\delta_h - \delta} - c_{0h} - c_g} \tag{E7}$$

With equation E7 the stability constant *K* was calculated for every NMR-measurement separately. The arithmetic average over all datapoints led to the final value of *K* which was used for the calculation of ΔG_0.

$$\Delta G_0 = -RT \ln K \tag{E8}$$

The uncertainties of *K* and ΔG_0 were based on the standard deviation of the arithmetic average of *K* and the Gaussian error propagation for $\Delta\Delta G_0$.

$$\Delta\Delta G_0 = \frac{\delta \Delta G_0}{\delta K} \cdot \Delta K = \frac{RT}{K} \cdot \Delta K \tag{E9}$$

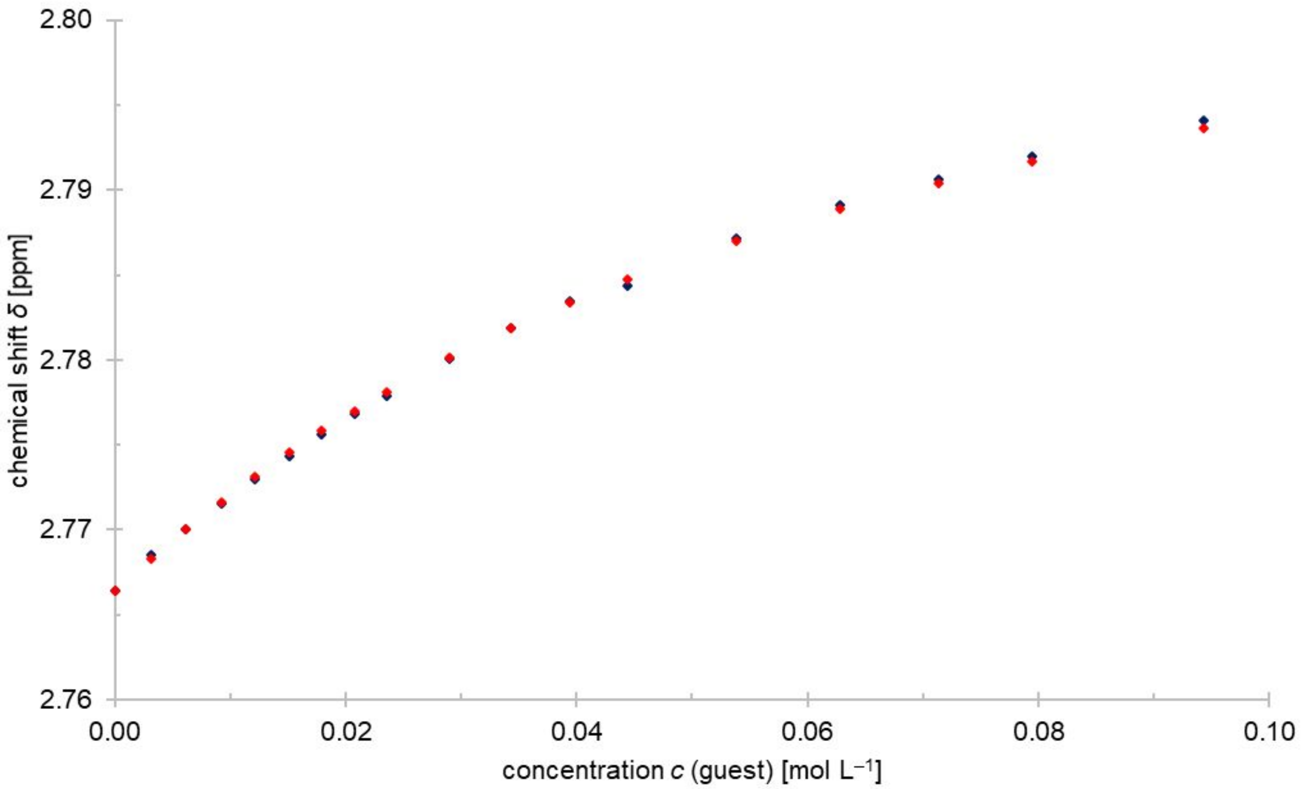

Graph S1: Titration curve of a quinuclidine (host) /pentafluoriodobenzene (guest) mixture in C_6D_{12} at 298.13 K, where the chemical shift *δ* is plotted against the guest concentration *c*. The quinuclidine concentraion was maintained constant. The experimental values were determined from the observation of the *ortho* proton of the quinuclidine. A logarithmic fit could be generated. (blue = experimental data; red = theoretical data).

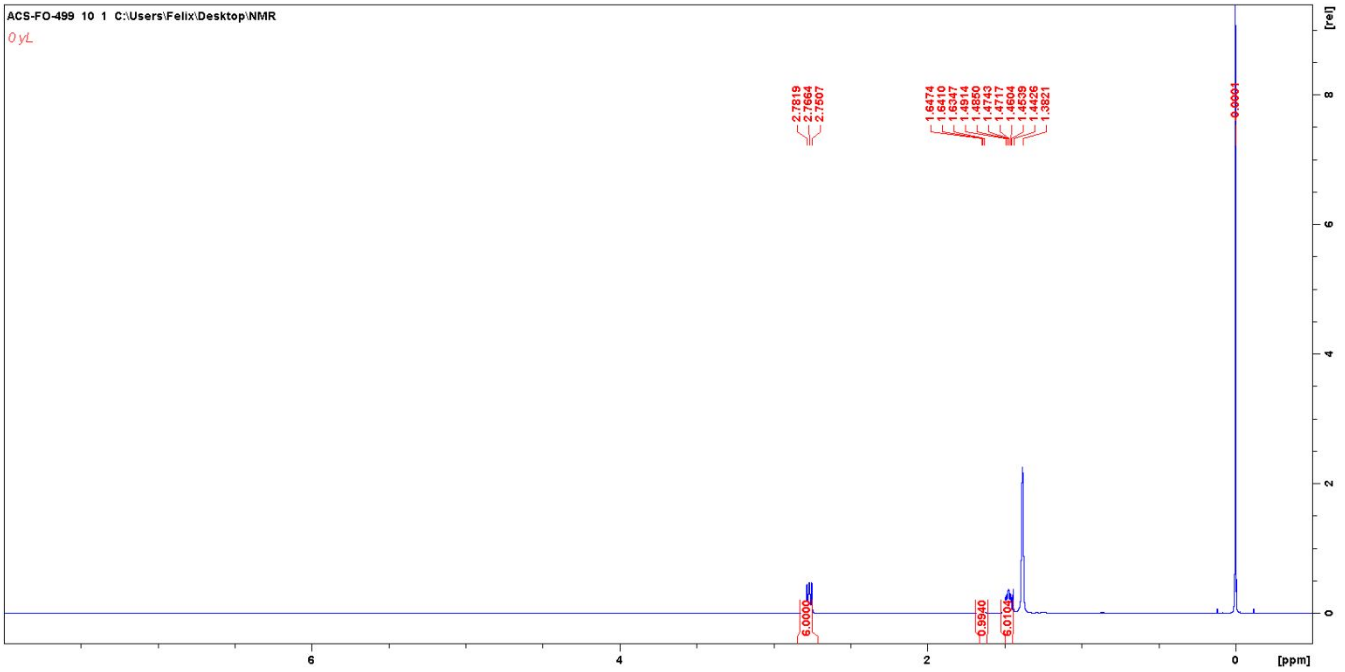

Figure S27: ¹H NMR spectra of quinuclidine in cyclohexane-d12. Chemical shifts are reported relative to TMS.

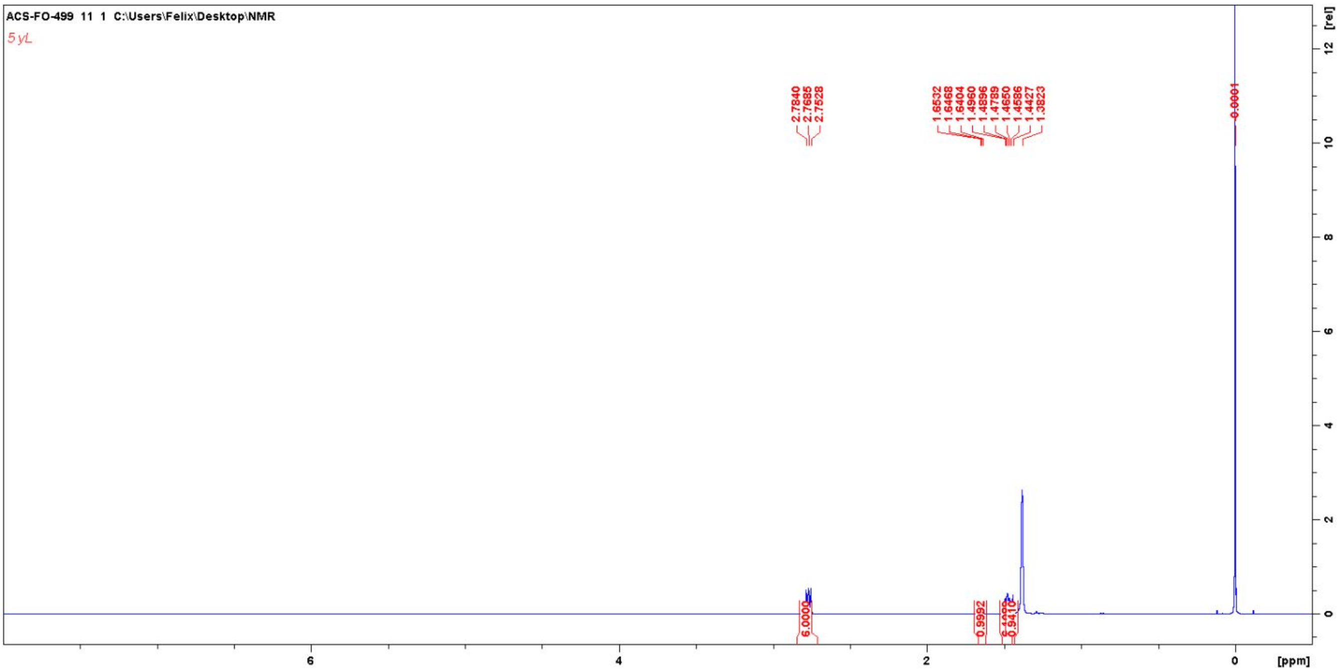

Figure S28: ^{1}H NMR spectra of quinuclidine (host) and pentafluoriodobenzene (guest, 5 µL) in cyclohexane-d12. Chemical shifts are reported relative to TMS.

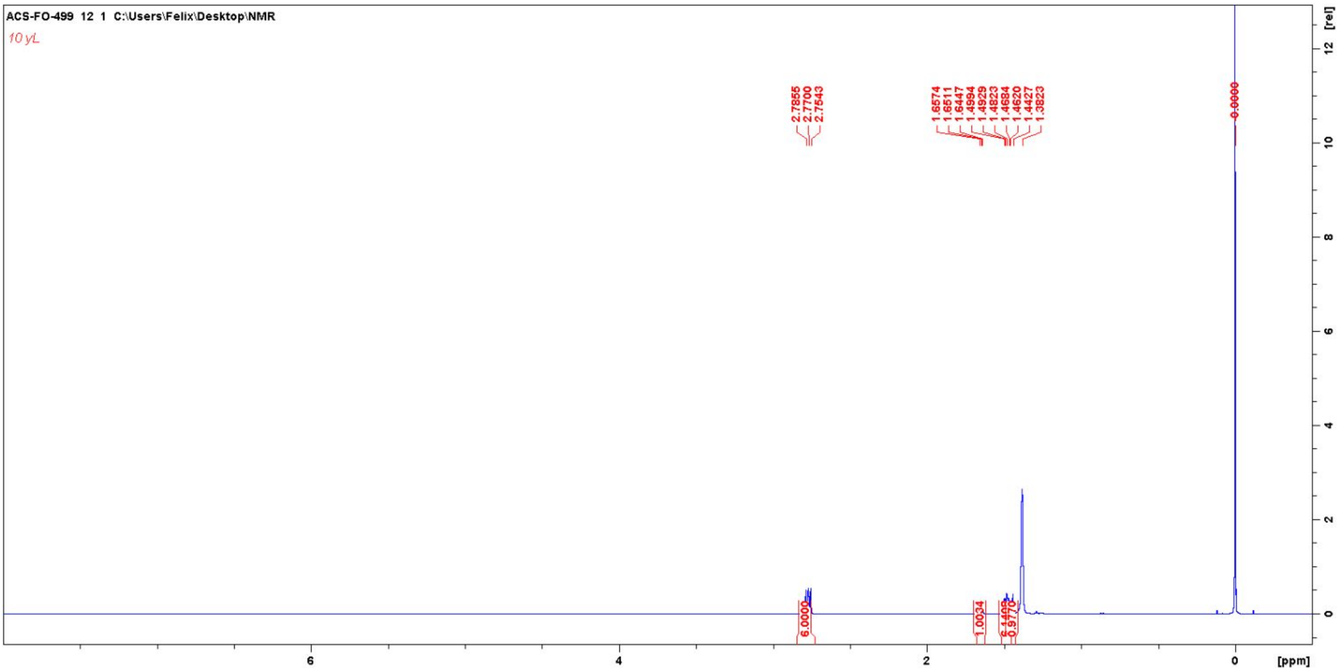

Figure S29: ^{1}H NMR spectra of quinuclidine (host) and pentafluoriodobenzene (guest, 10 µL) in cyclohexane-d12. Chemical shifts are reported relative to TMS.

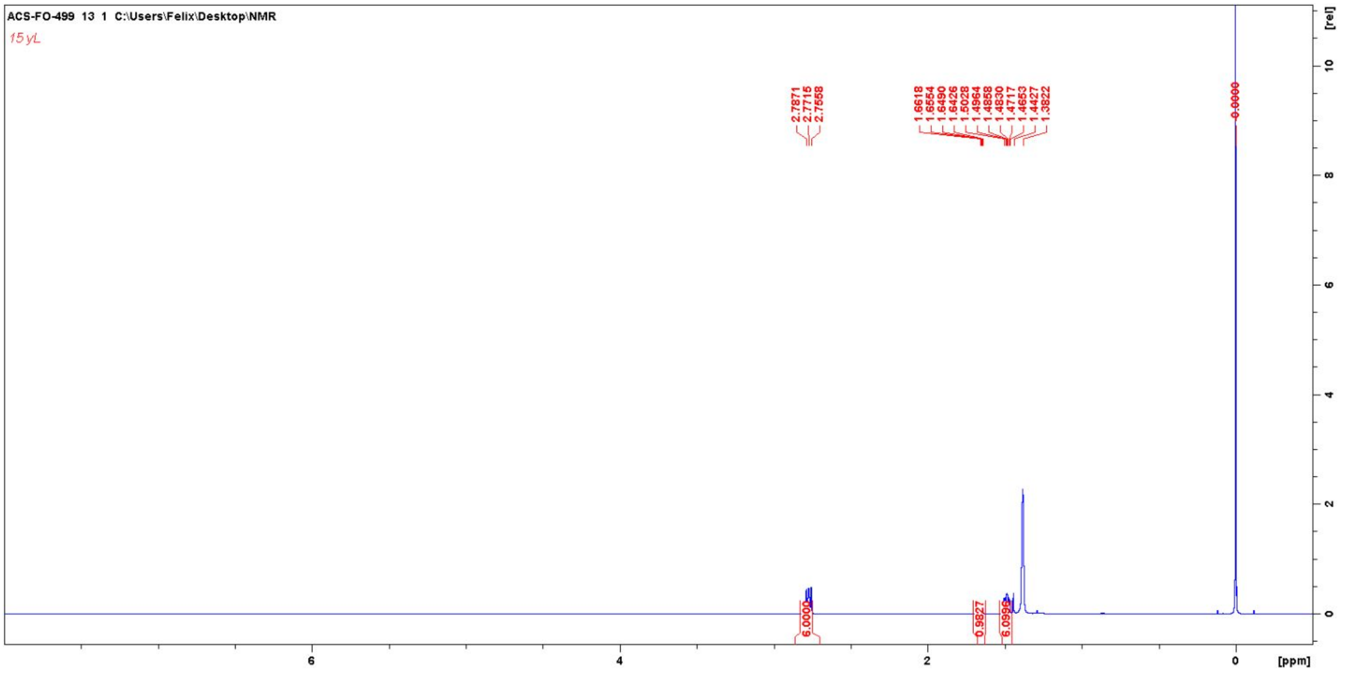

Figure S30: ^1H NMR spectra of quinuclidine (host) and pentafluoriodobenzene (guest, 15 µL) in cyclohexane-d12. Chemical shifts are reported relative to TMS.

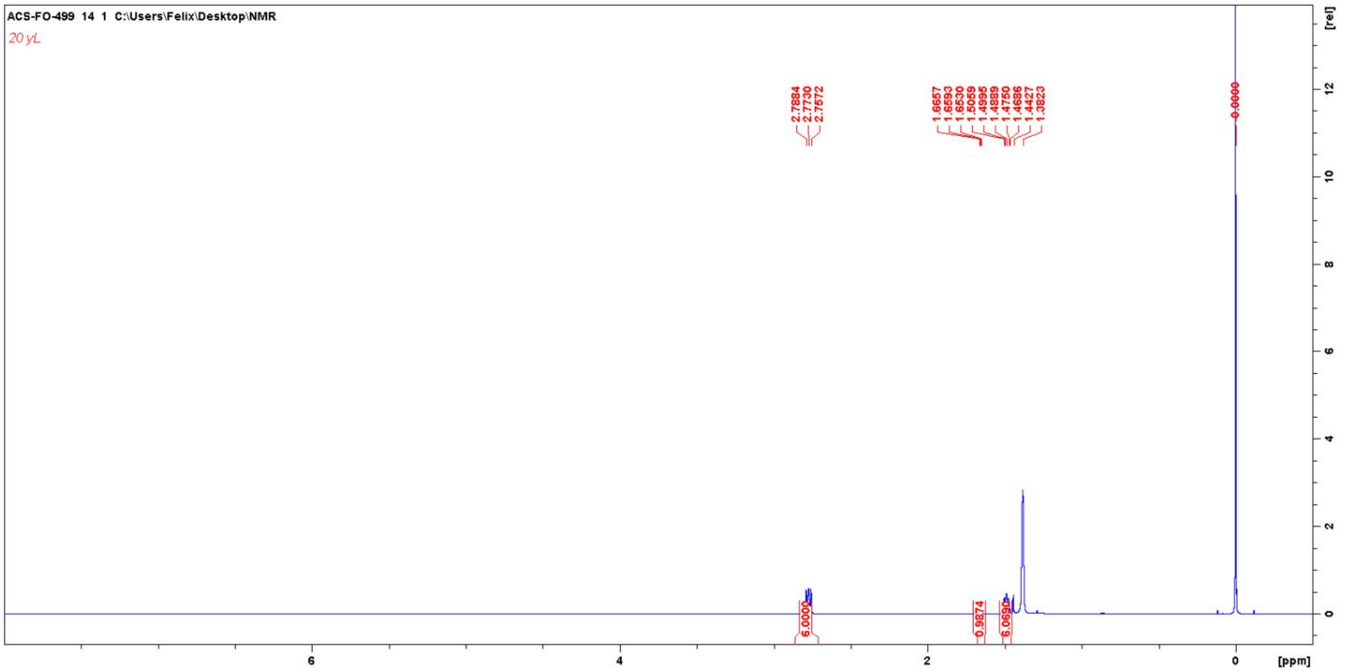

Figure S31: ^1H NMR spectra of quinuclidine (host) and pentafluoriodobenzene (guest, 20 µL) in cyclohexane-d12. Chemical shifts are reported relative to TMS.

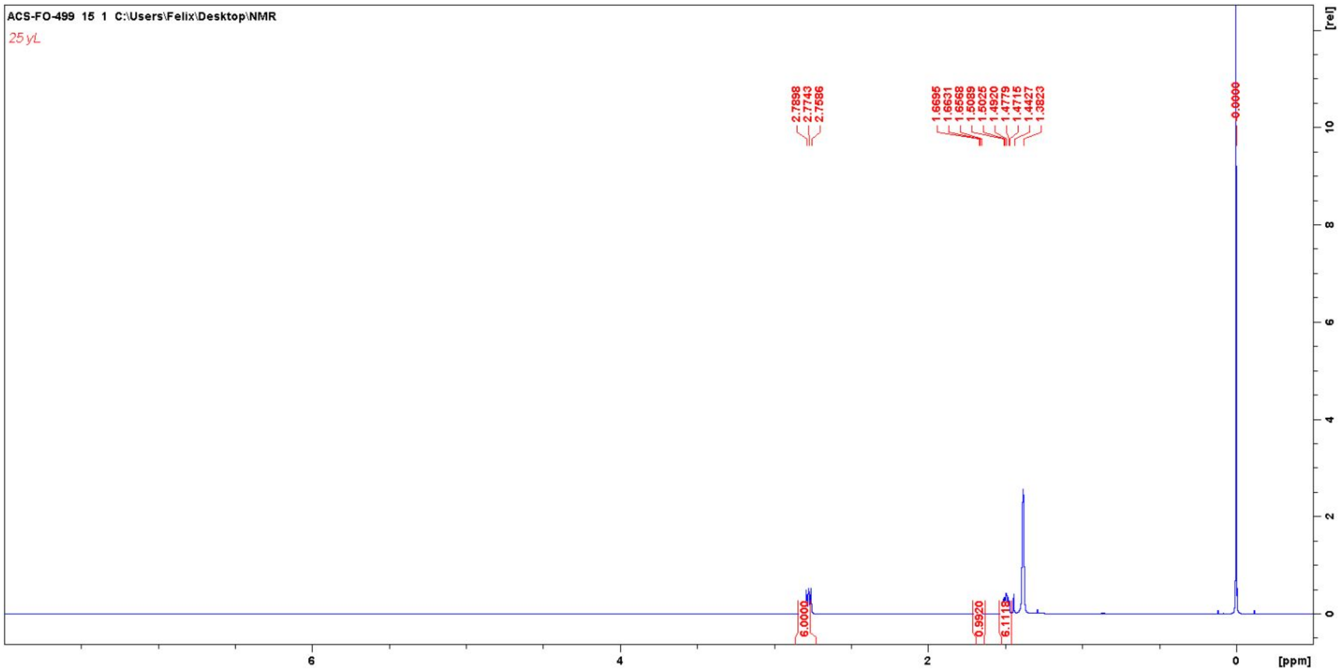

Figure S32: ^{1}H NMR spectra of quinuclidine (host) and pentafluoriodobenzene (guest, 25 µL) in cyclohexane-d12. Chemical shifts are reported relative to TMS.

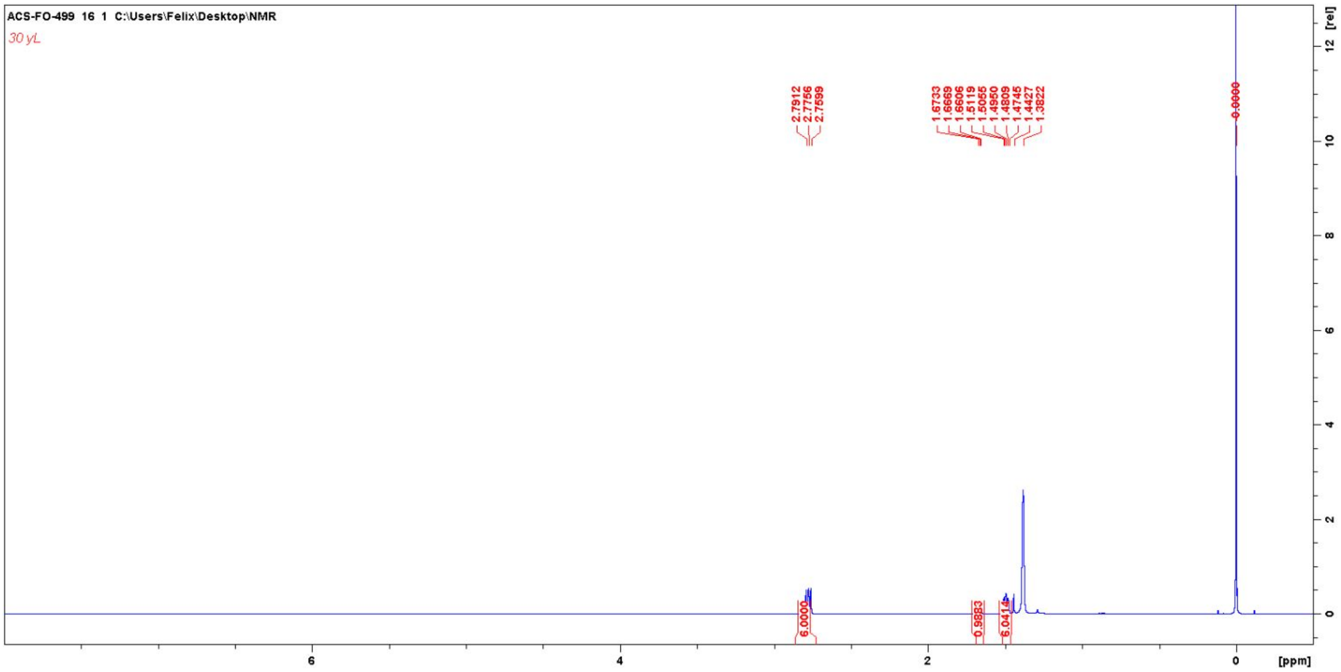

Figure S33: ^{1}H NMR spectra of quinuclidine (host) and pentafluoriodobenzene (guest, 30 µL) in cyclohexane-d12. Chemical shifts are reported relative to TMS.

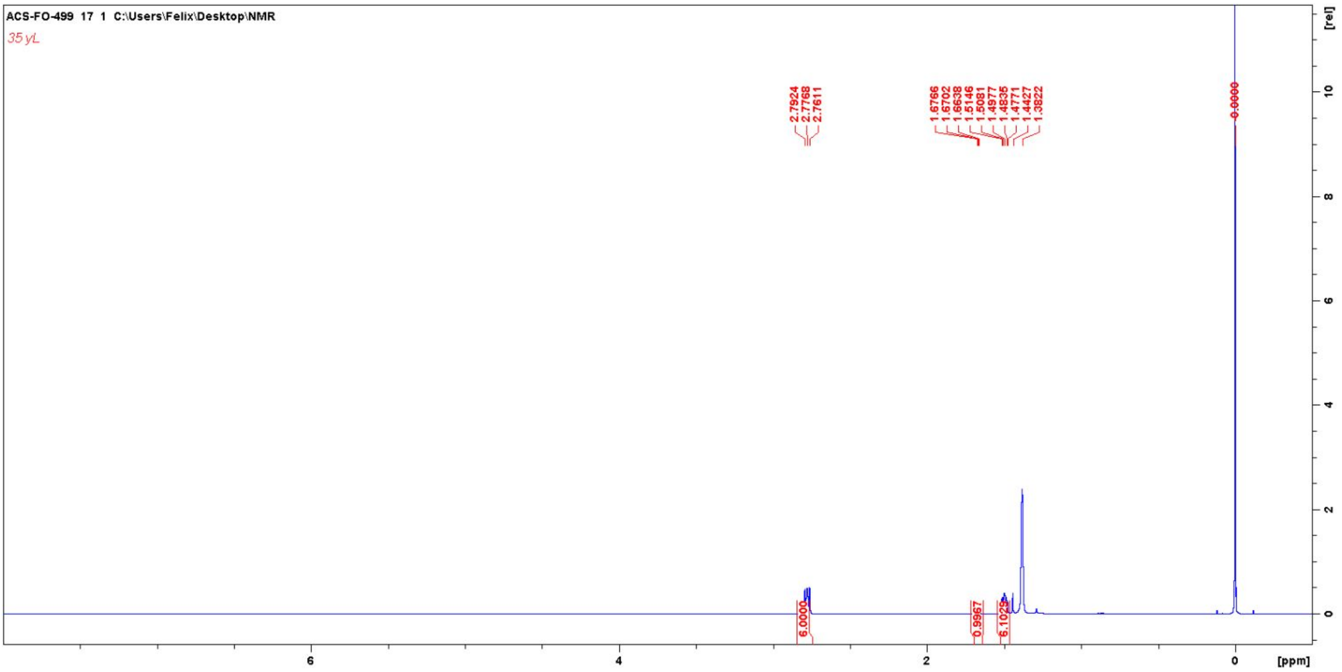

Figure S34: ^{1}H NMR spectra of quinuclidine (host) and pentafluoriodobenzene (guest, 35 µL) in cyclohexane-d12. Chemical shifts are reported relative to TMS.

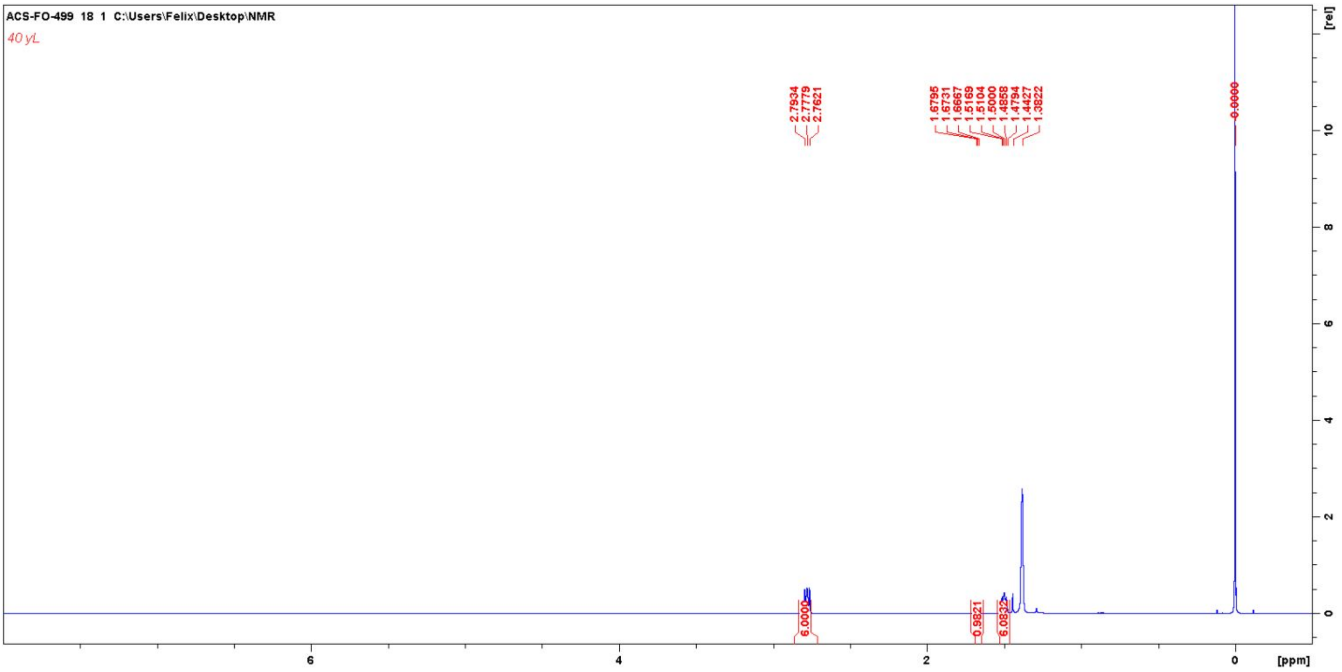

Figure S35: ^{1}H NMR spectra of quinuclidine (host) and pentafluoriodobenzene (guest, 40 µL) in cyclohexane-d12. Chemical shifts are reported relative to TMS.

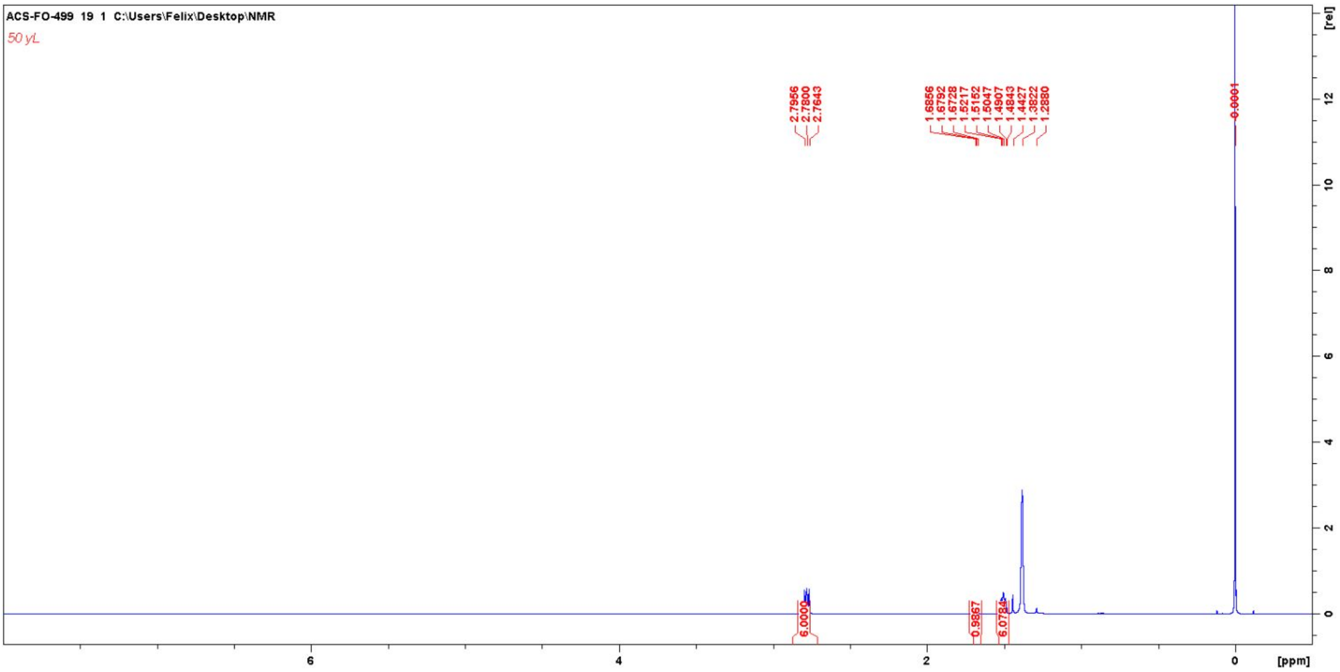

Figure S36: ^{1}H NMR spectra of quinuclidine (host) and pentafluoriodobenzene (guest, 50 µL) in cyclohexane-d12. Chemical shifts are reported relative to TMS.

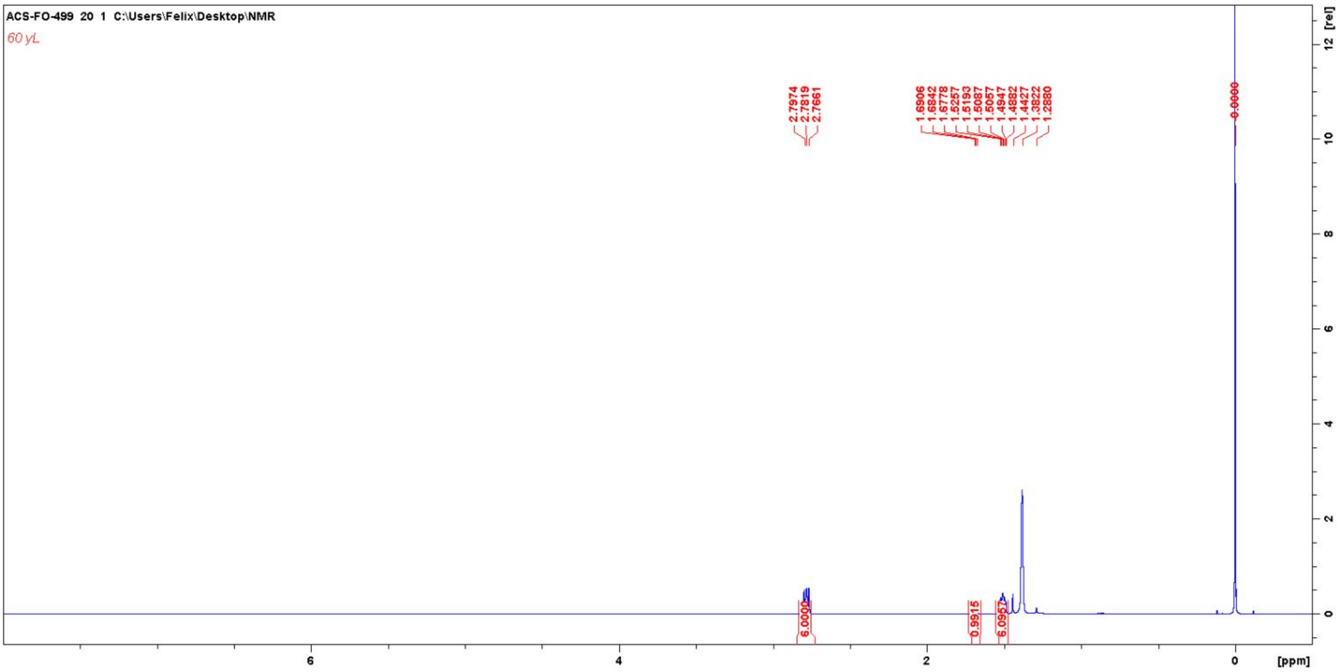

Figure S37: ^{1}H NMR spectra of quinuclidine (host) and pentafluoriodobenzene (guest, 60 µL) in cyclohexane-d12. Chemical shifts are reported relative to TMS.

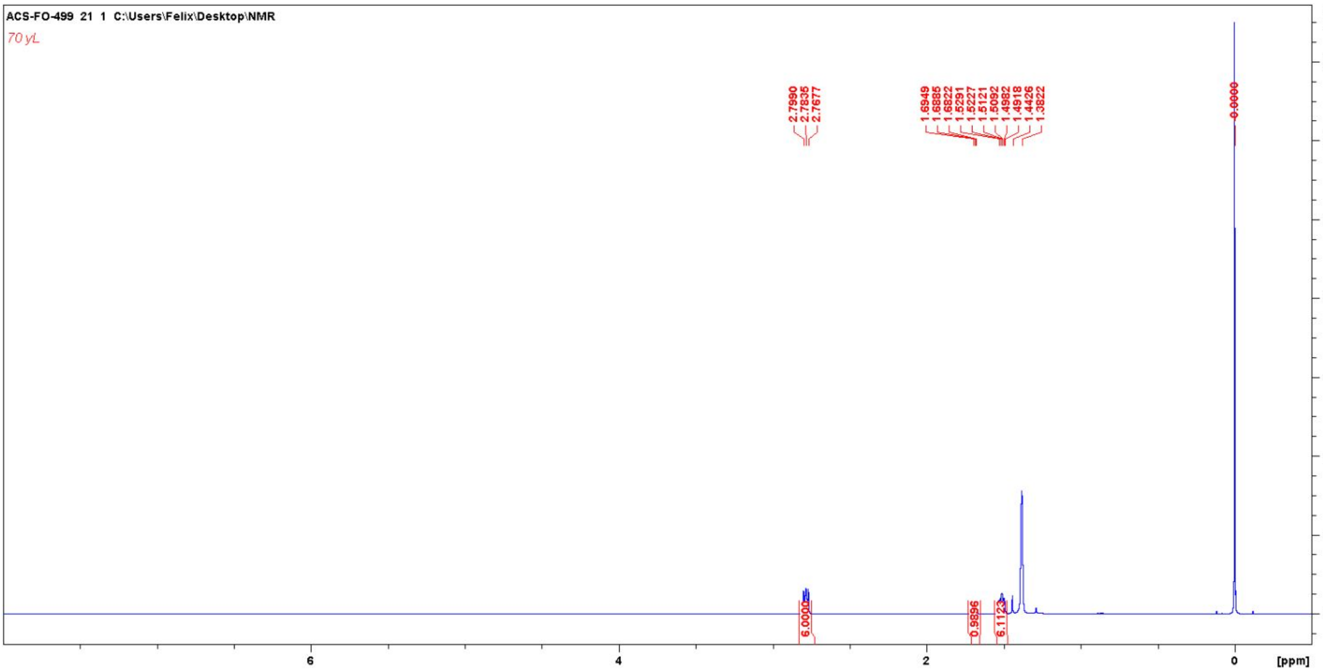

Figure S38: ^{1}H NMR spectra of quinuclidine (host) and pentafluoriodobenzene (guest, 70 µL) in cyclohexane-d12. Chemical shifts are reported relative to TMS.

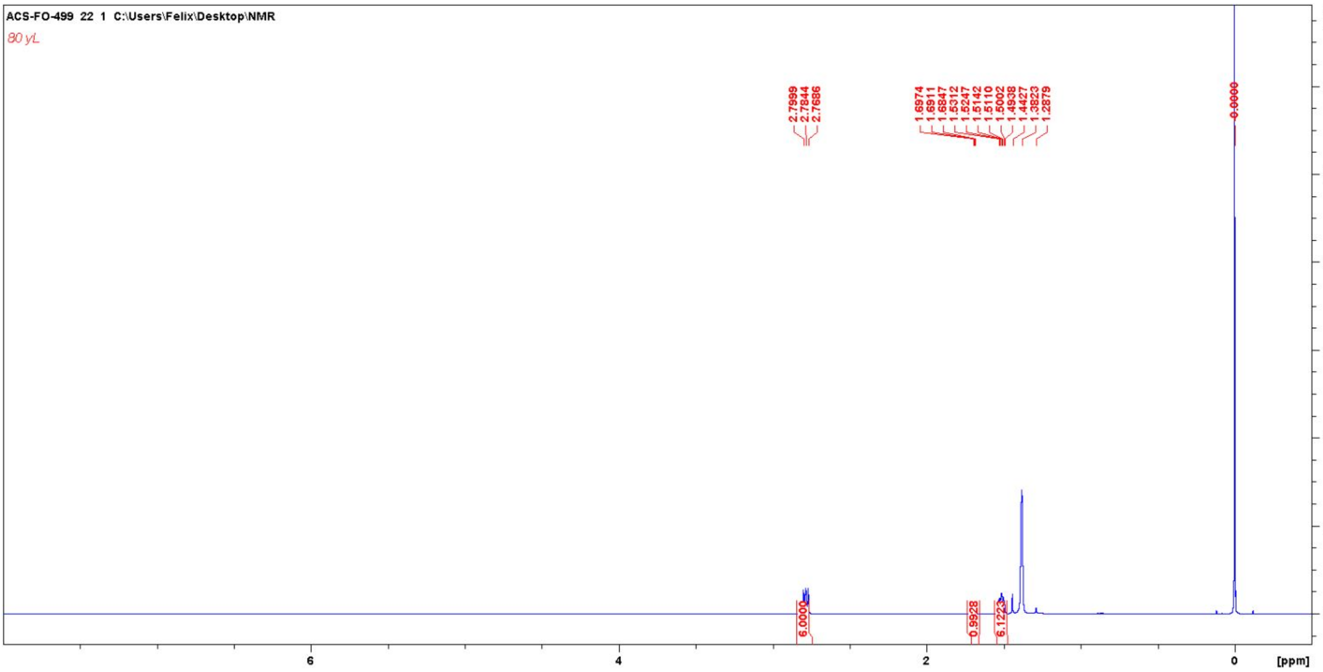

Figure S39: ^{1}H NMR spectra of quinuclidine (host) and pentafluoriodobenzene (guest, 80 µL) in cyclohexane-d12. Chemical shifts are reported relative to TMS.

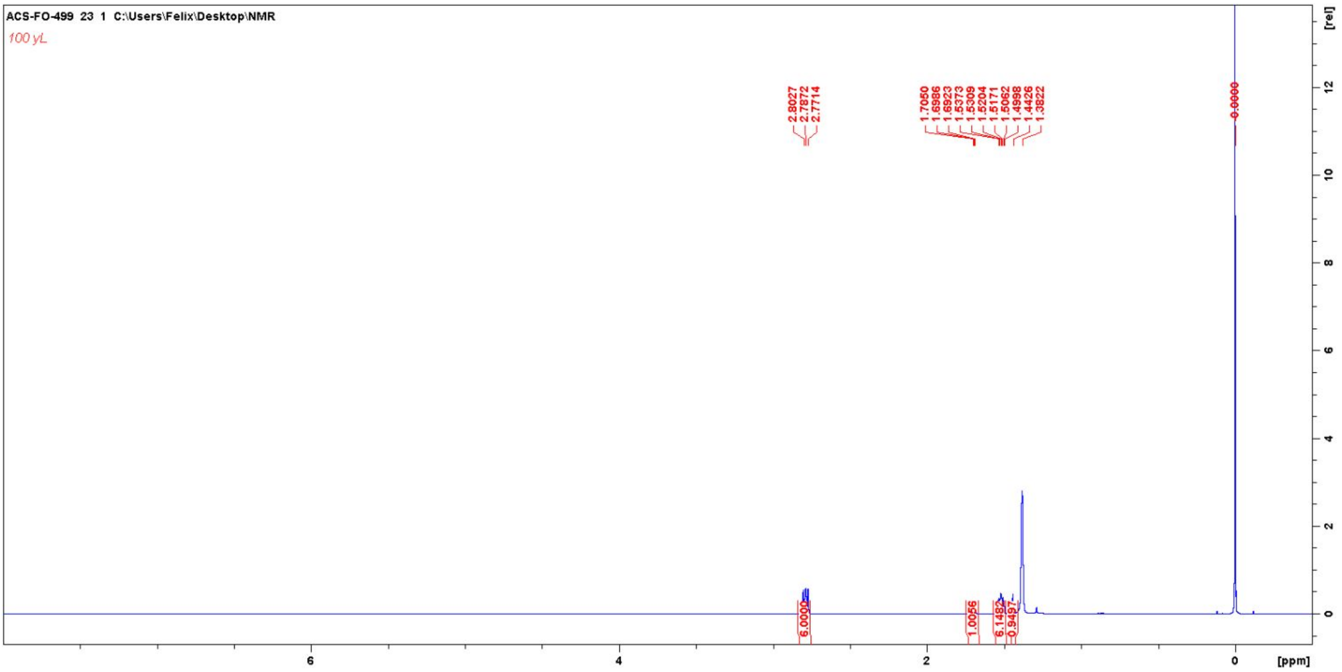

Figure S40: ^{1}H NMR spectra of quinuclidine (host) and pentafluoriodobenzene (guest, 100 µL) in cyclohexane-d12. Chemical shifts are reported relative to TMS.

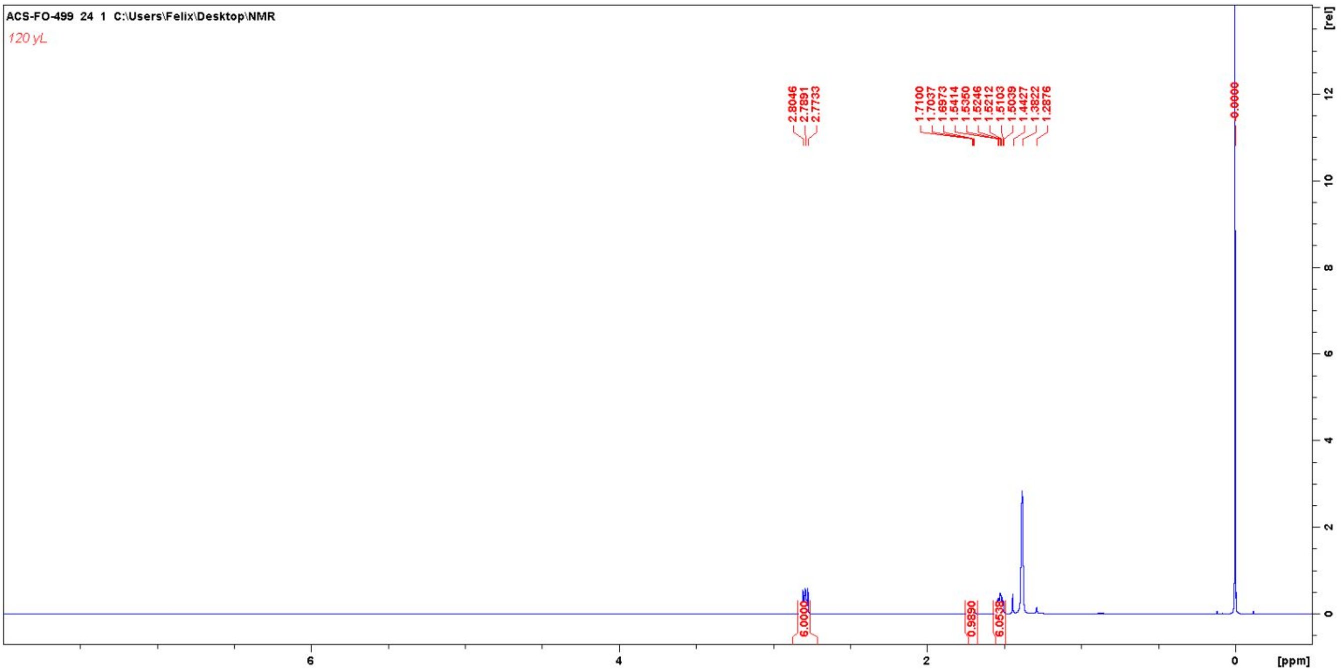

Figure S41: ^{1}H NMR spectra of quinuclidine (host) and pentafluoriodobenzene (guest, 120 µL) in cyclohexane-d12. Chemical shifts are reported relative to TMS.

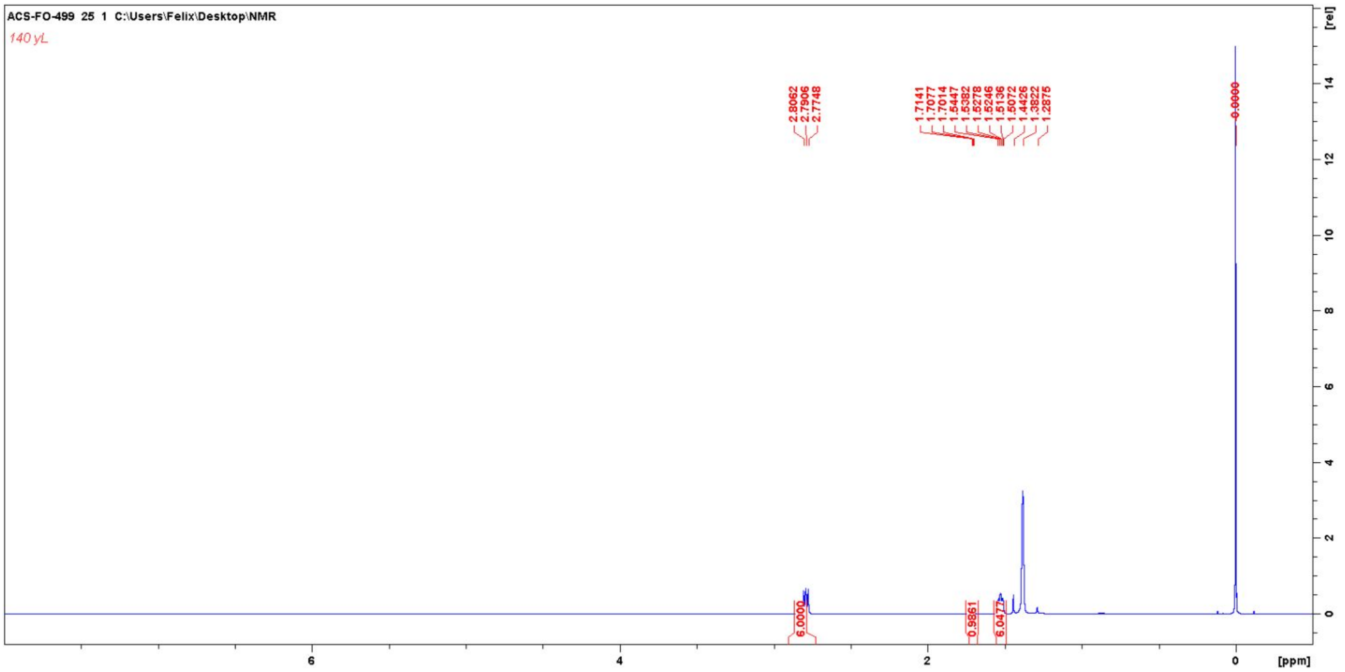

Figure S42: ^{1}H NMR spectra of quinuclidine (host) and pentafluoriodobenzene (guest, 140 µL) in cyclohexane-d12. Chemical shifts are reported relative to TMS.

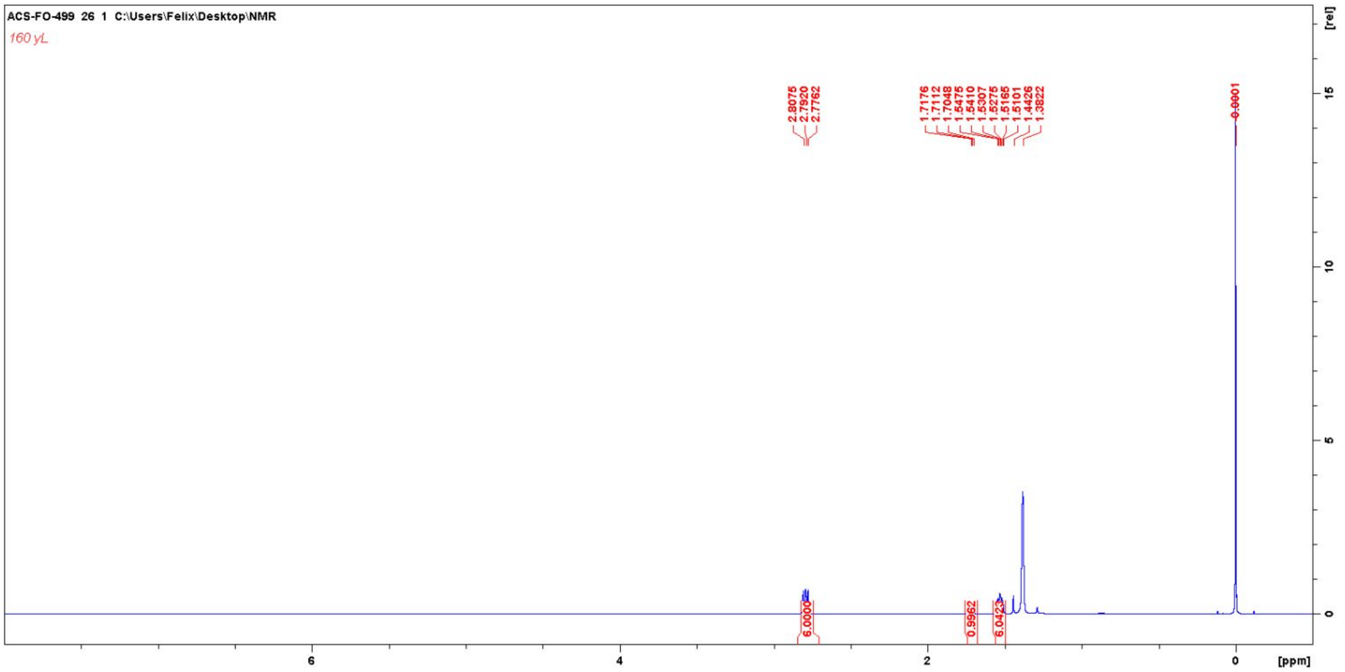

Figure S43: ^{1}H NMR spectra of quinuclidine (host) and pentafluoriodobenzene (guest, 160 µL) in cyclohexane-d12. Chemical shifts are reported relative to TMS.

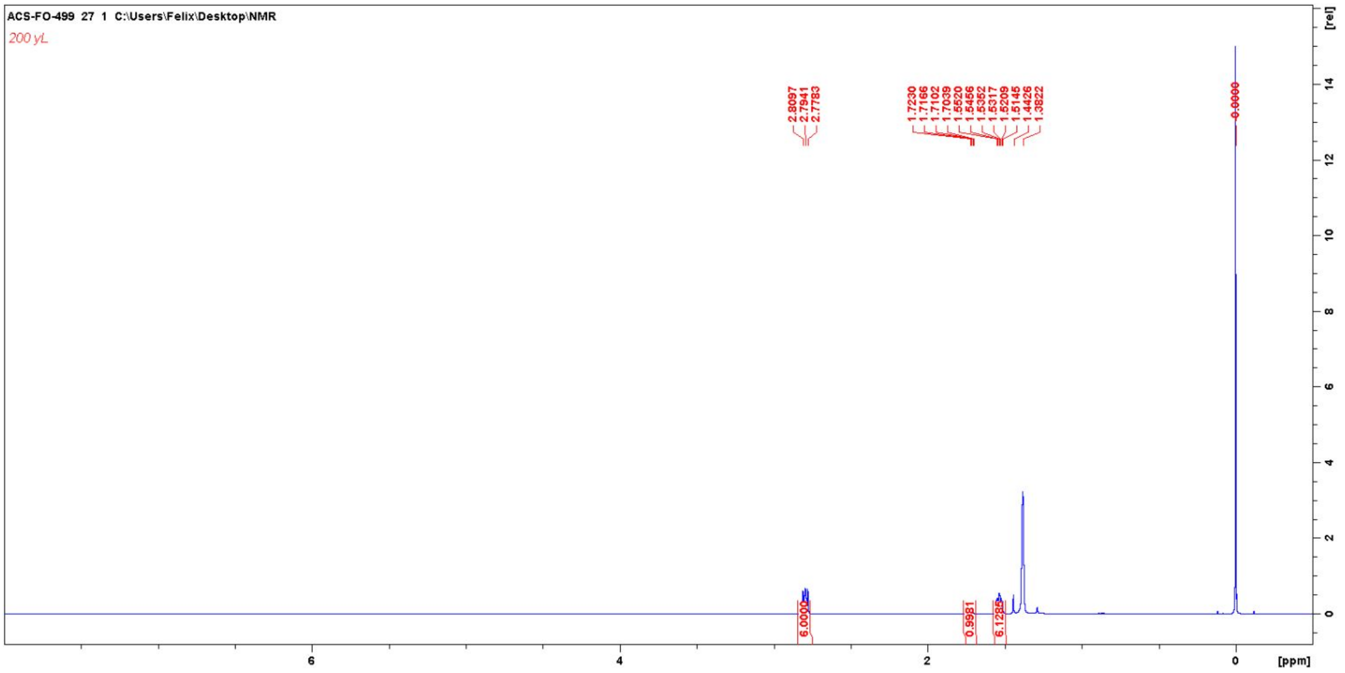

Figure S44: ^{1}H NMR spectra of quinuclidine (host) and pentafluoriodobenzene (guest, 200 µL) in cyclohexane-d12. Chemical shifts are reported relative to TMS.

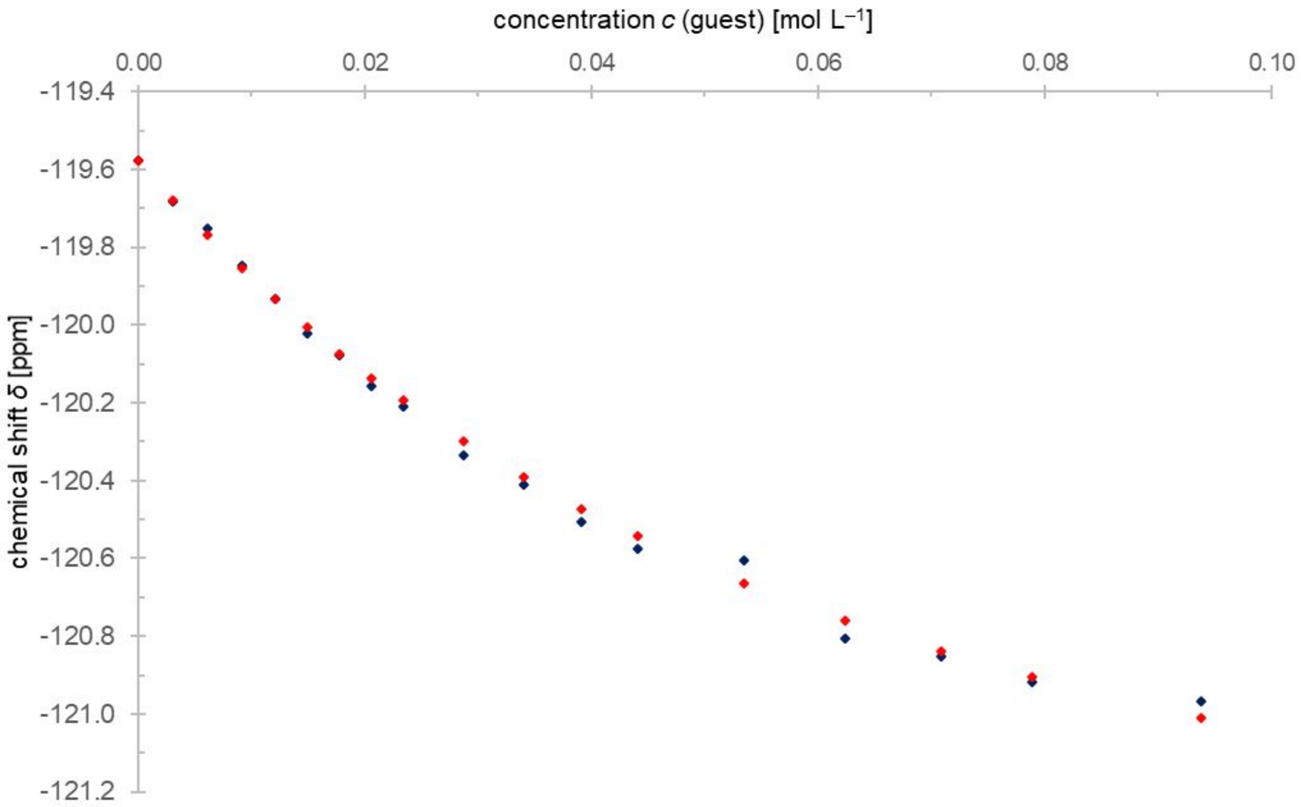

Graph S2: Titration curve of pentafluoriodobenzene (host) quinuclidine (guest) mixture in C_6D_{12} at 298.13 K, where the chemical shift *δ* is plotted against the guest concentration *c*. The pentafluoriodobenzene concentraion was maintained constant. The experimental values were determined from the observation of the *ortho* fluorine atoms of the pentafluoriodobenzene. A logarithmic fit could be generated. (blue = experimental data; red = theoretical data).

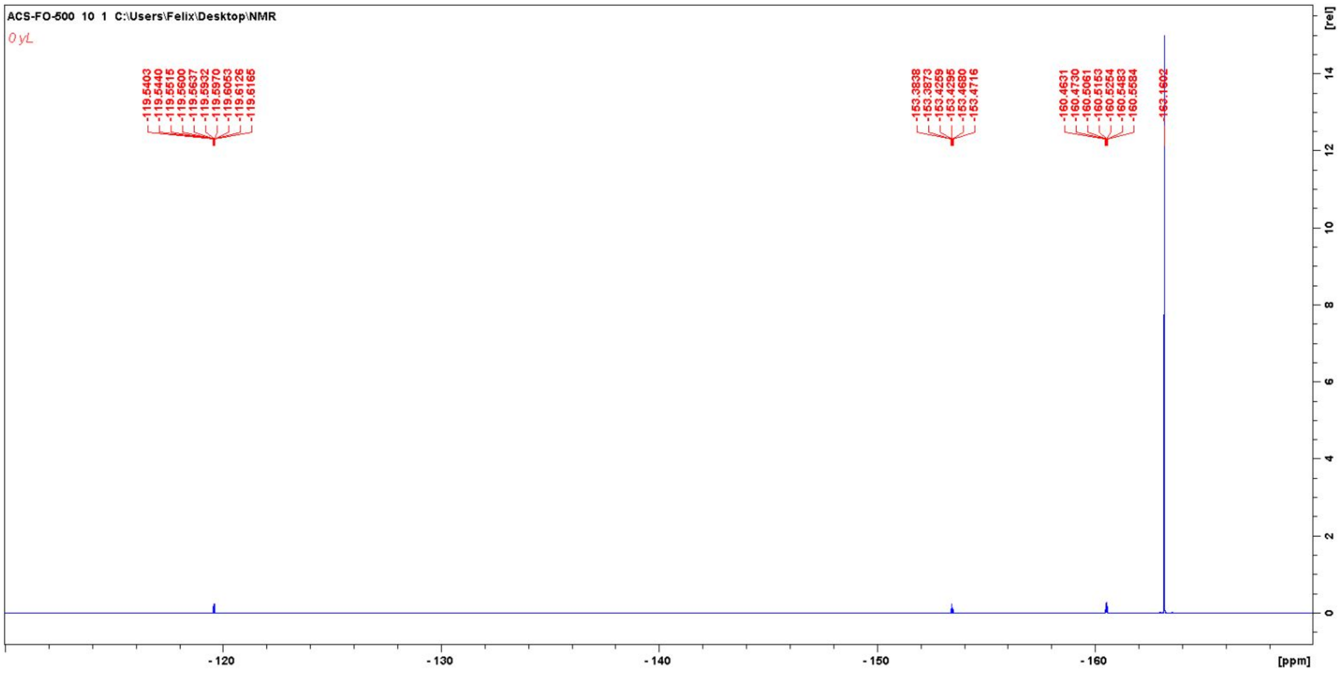

Figure S45: ^{19}F NMR spectra of pentafluoriodobenzene in cyclohexane-d12. Chemical shifts are reported relative to hexafluorobenzene.

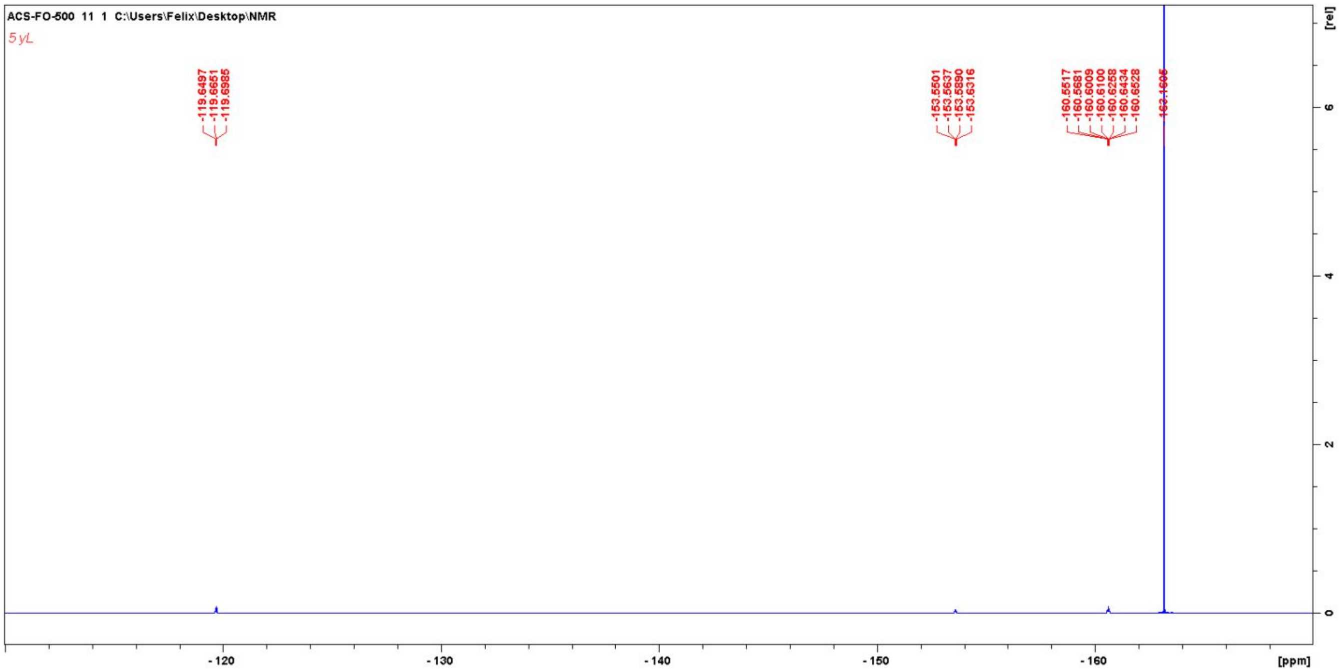

Figure S46: ^{19}F NMR spectra of pentafluoriodobenzene (host) and quinuclidine (guest, 5 µL) in cyclohexane-d12. Chemical shifts are reported relative to hexafluorobenzene.

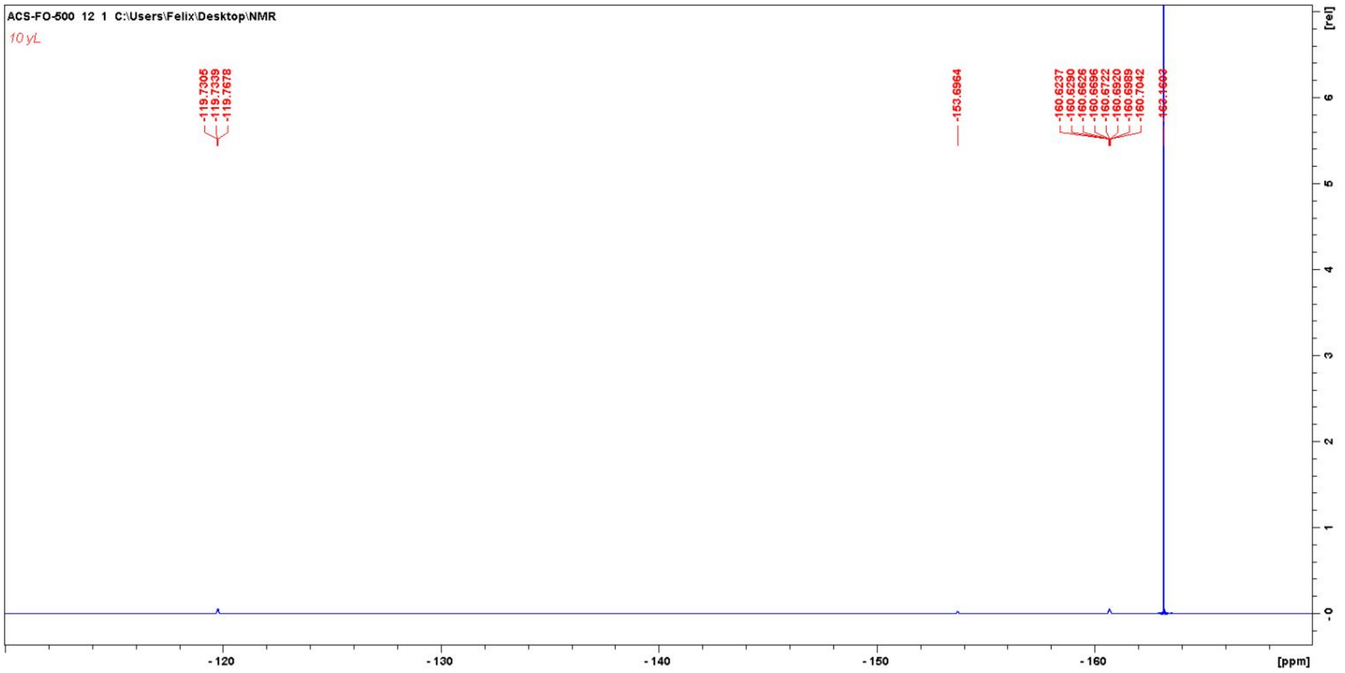

Figure S47: ^{19}F NMR spectra of pentafluoriodobenzene (host) and quinuclidine (guest, 10 µL) in cyclohexane-d12. Chemical shifts are reported relative to hexafluorobenzene.

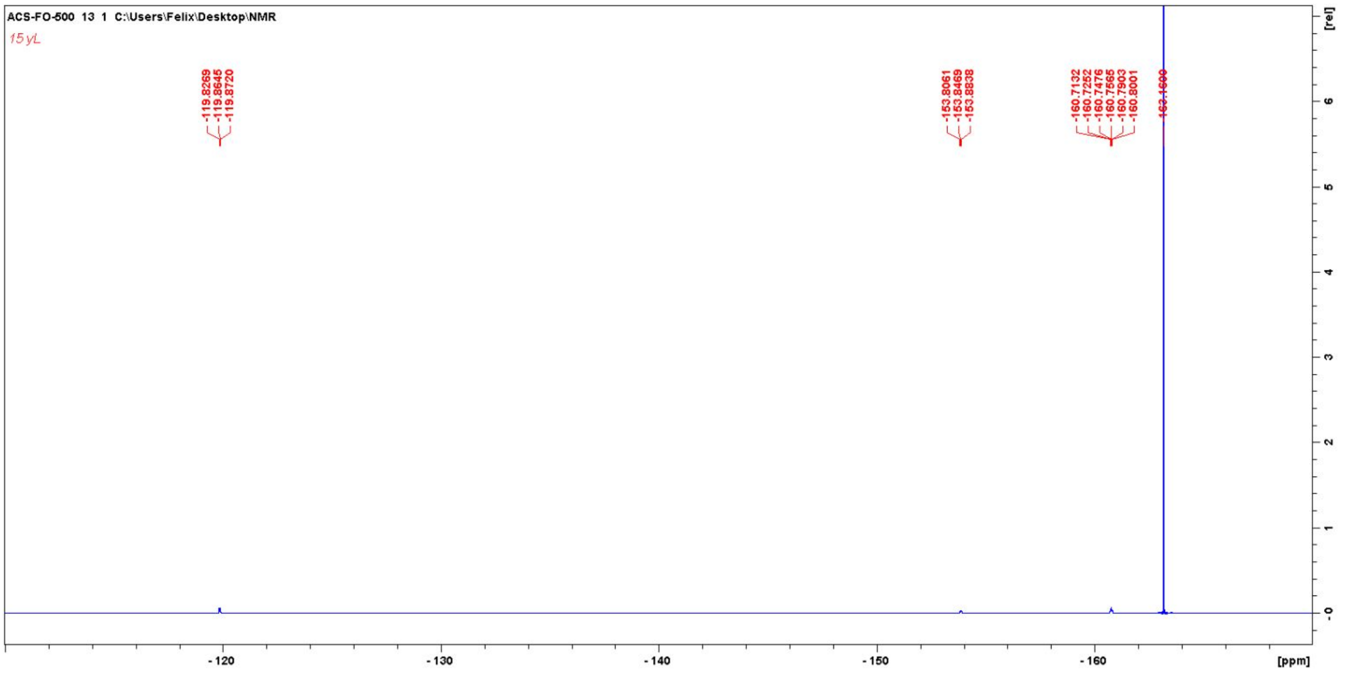

Figure S48: ^{19}F NMR spectra of pentafluoriodobenzene (host) and quinuclidine (guest, 15 µL) in cyclohexane-d12. Chemical shifts are reported relative to hexafluorobenzene.

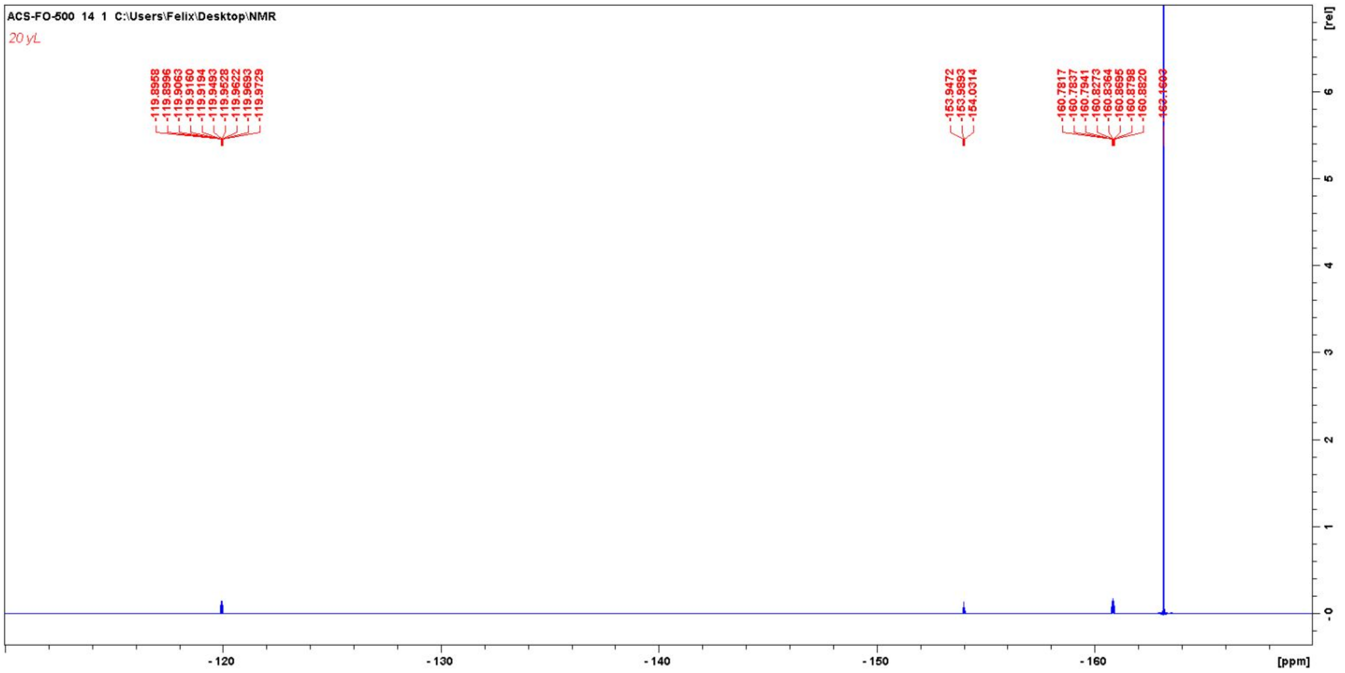

Figure S49: ^{19}F NMR spectra of pentafluoriodobenzene (host) and quinuclidine (guest, 20 µL) in cyclohexane-d12. Chemical shifts are reported relative to hexafluorobenzene.

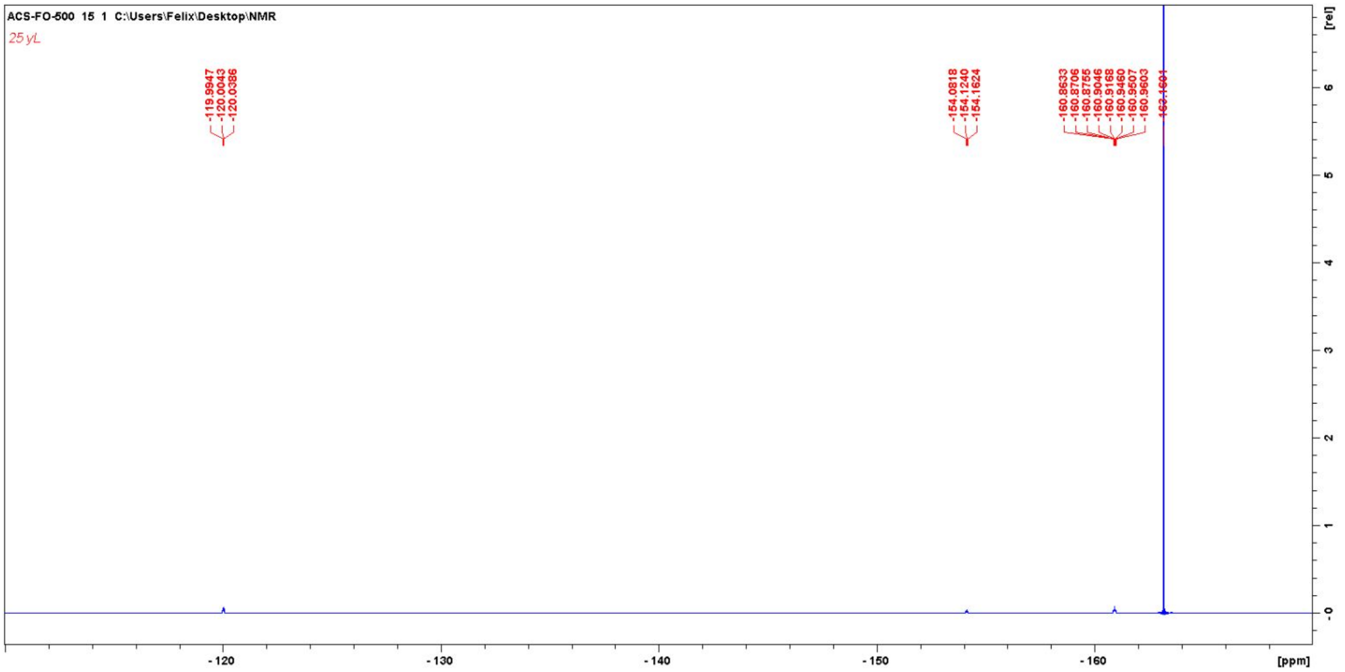

Figure S50: ^{19}F NMR spectra of pentafluoriodobenzene (host) and quinuclidine (guest, 25 µL) in cyclohexane-d12. Chemical shifts are reported relative to hexafluorobenzene.

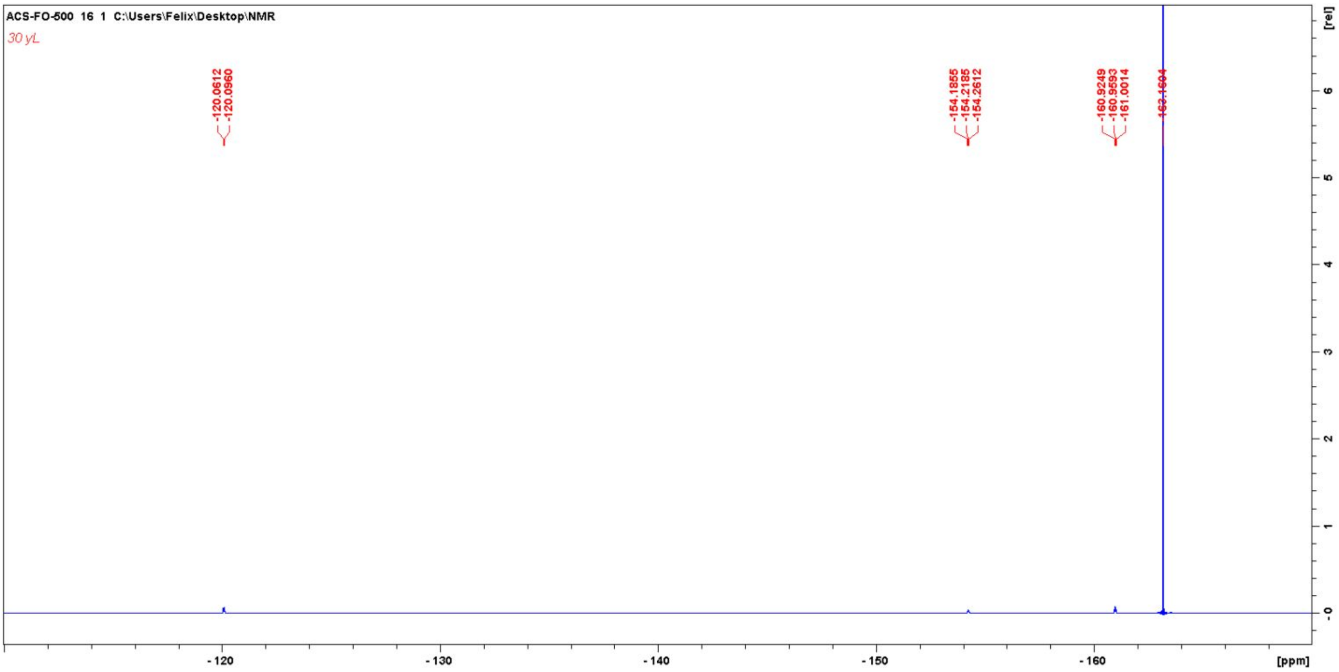

Figure S51: ^{19}F NMR spectra of pentafluoriodobenzene (host) and quinuclidine (guest, 30 µL) in cyclohexane-d12. Chemical shifts are reported relative to hexafluorobenzene.

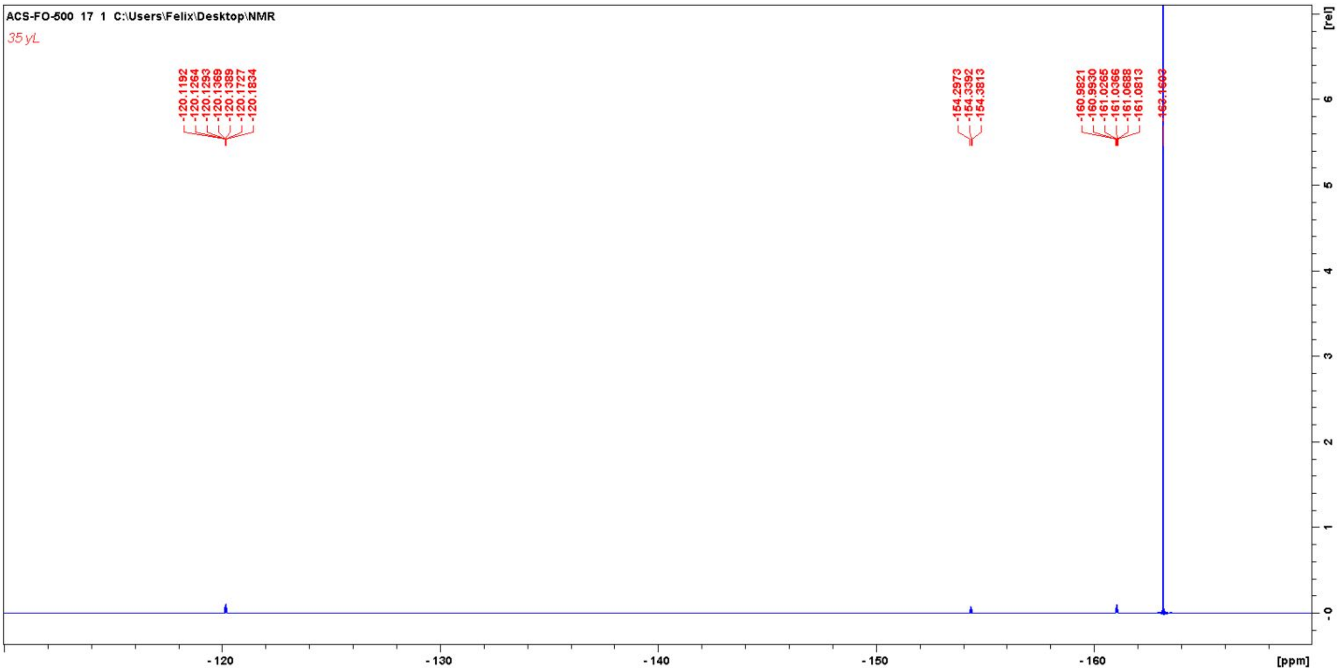

Figure S52: ^{19}F NMR spectra of pentafluoriodobenzene (host) and quinuclidine (guest, 35 µL) in cyclohexane-d12. Chemical shifts are reported relative to hexafluorobenzene.

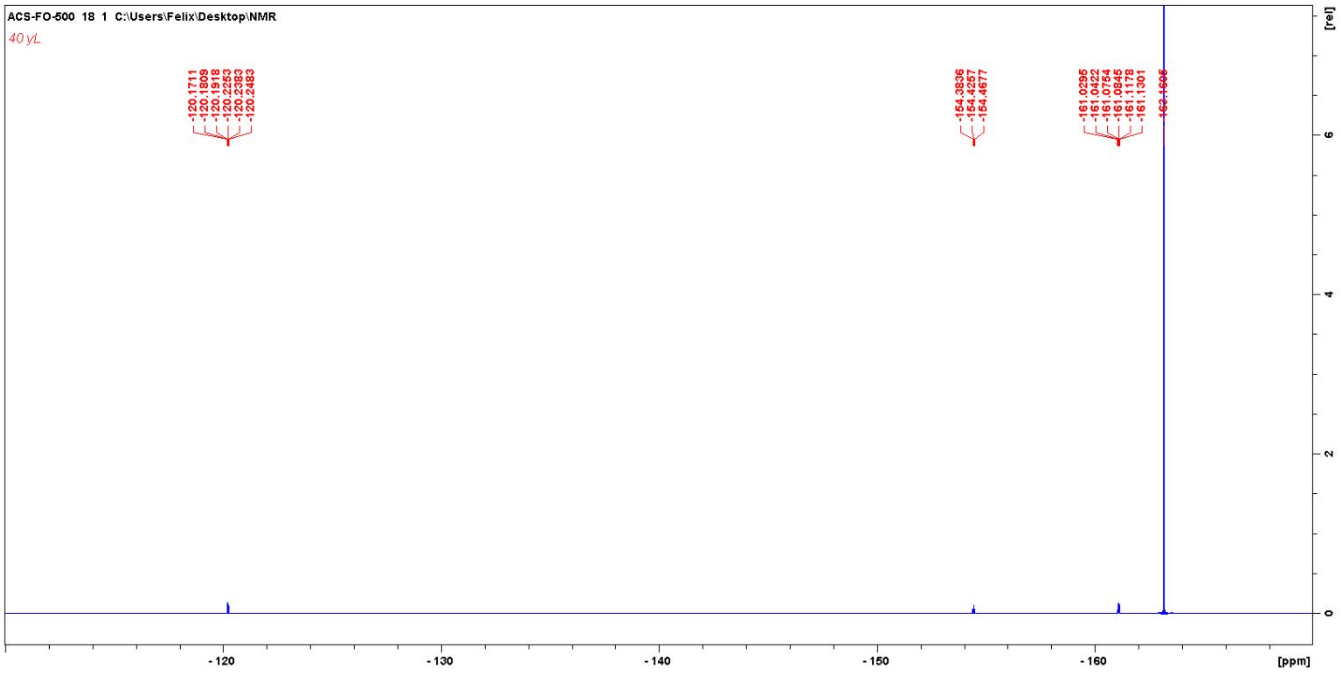

Figure S53: ^{19}F NMR spectra of pentafluoriodobenzene (host) and quinuclidine (guest, 40 µL) in cyclohexane-d12. Chemical shifts are reported relative to hexafluorobenzene.

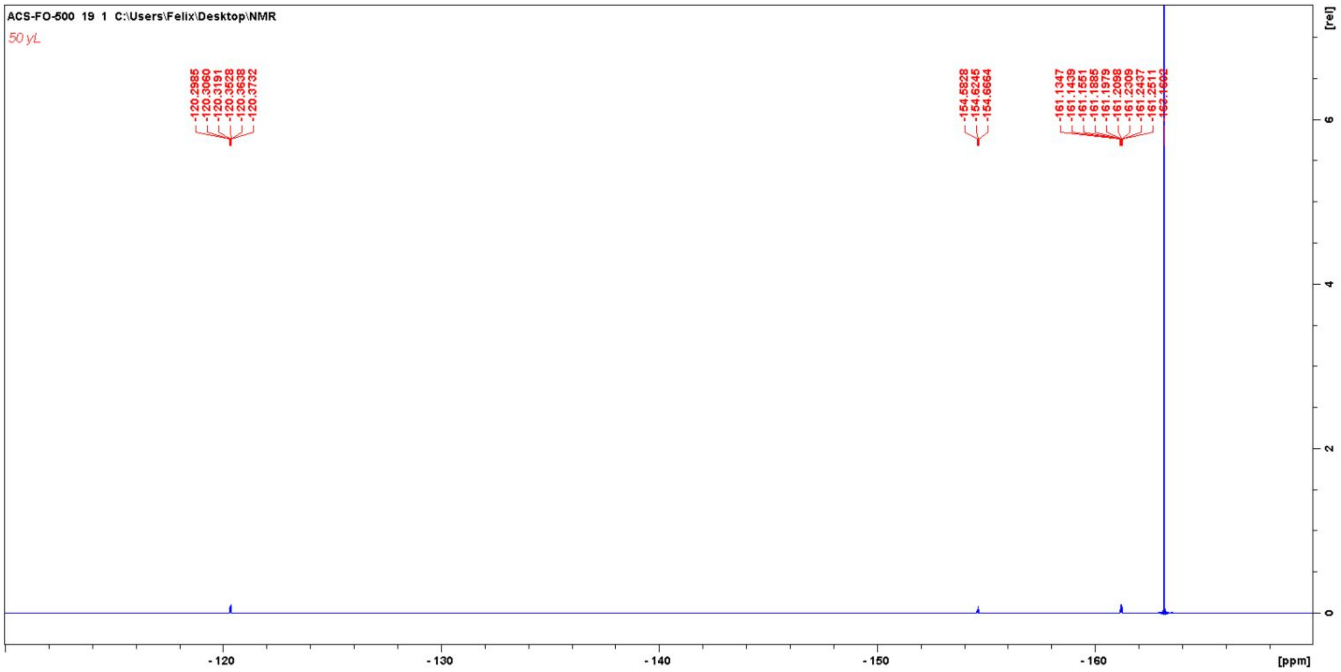

Figure S54: ^{19}F NMR spectra of pentafluoriodobenzene (host) and quinuclidine (guest, 50 µL) in cyclohexane-d12. Chemical shifts are reported relative to hexafluorobenzene.

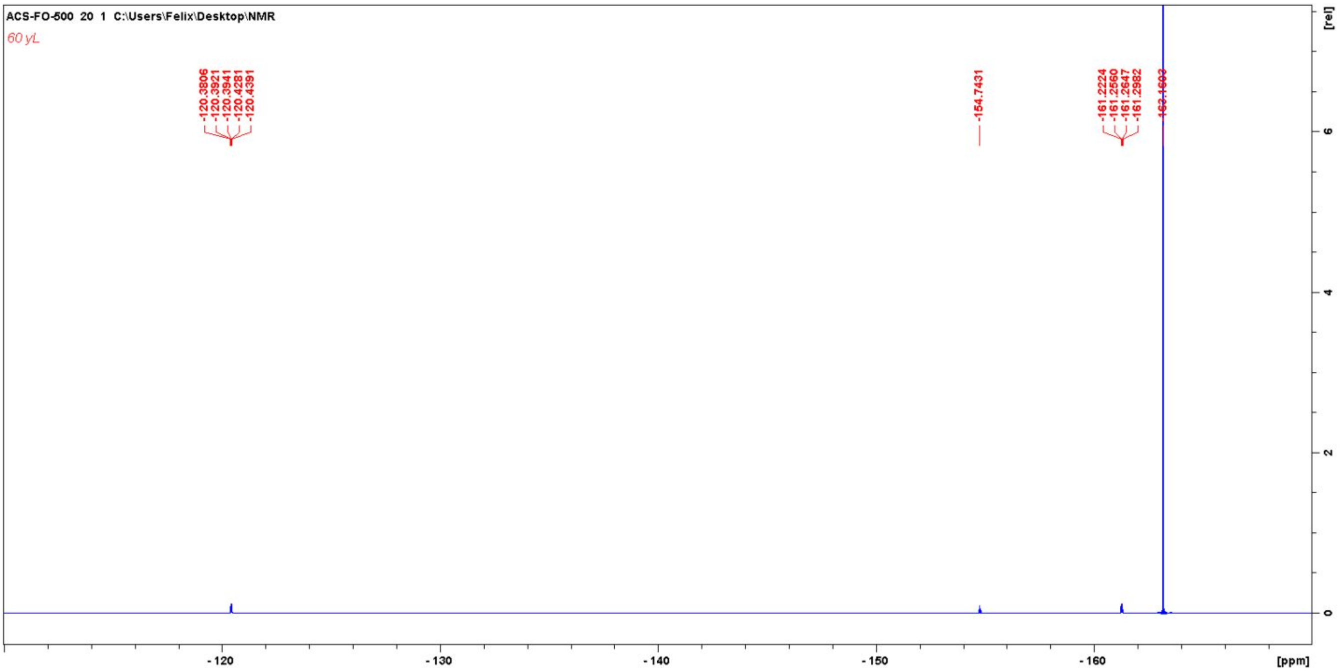

Figure S55: ^{19}F NMR spectra of pentafluoriodobenzene (host) and quinuclidine (guest, 60 µL) in cyclohexane-d12. Chemical shifts are reported relative to hexafluorobenzene.

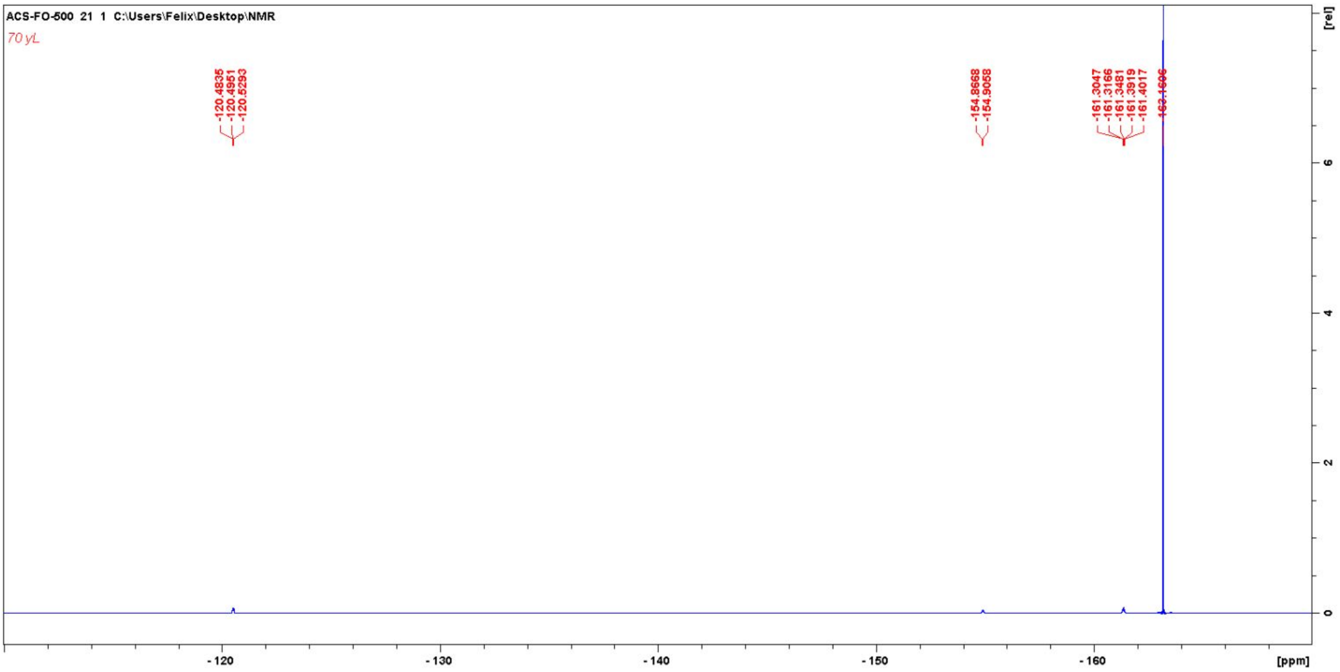

Figure S56: ^{19}F NMR spectra of pentafluoriodobenzene (host) and quinuclidine (guest, 70 µL) in cyclohexane-d12. Chemical shifts are reported relative to hexafluorobenzene.

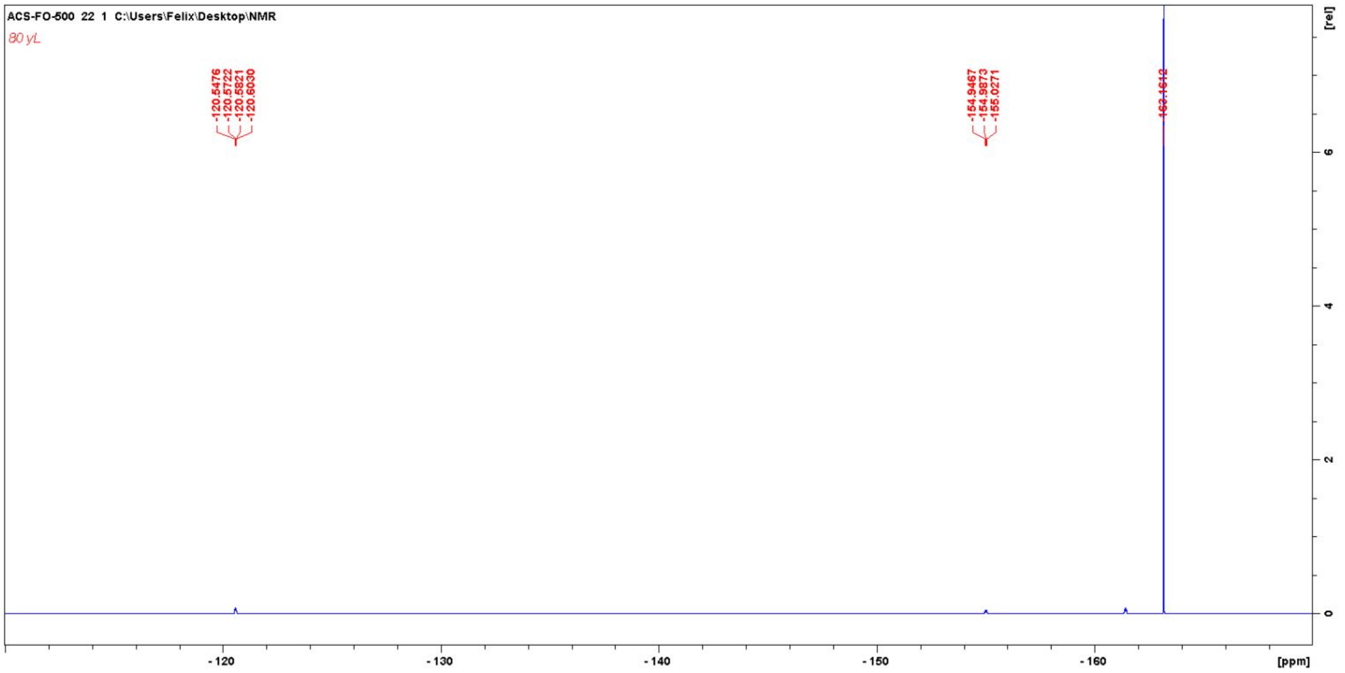

Figure S57: ^{19}F NMR spectra of pentafluoriodobenzene (host) and quinuclidine (guest, 80 µL) in cyclohexane-d12. Chemical shifts are reported relative to hexafluorobenzene.

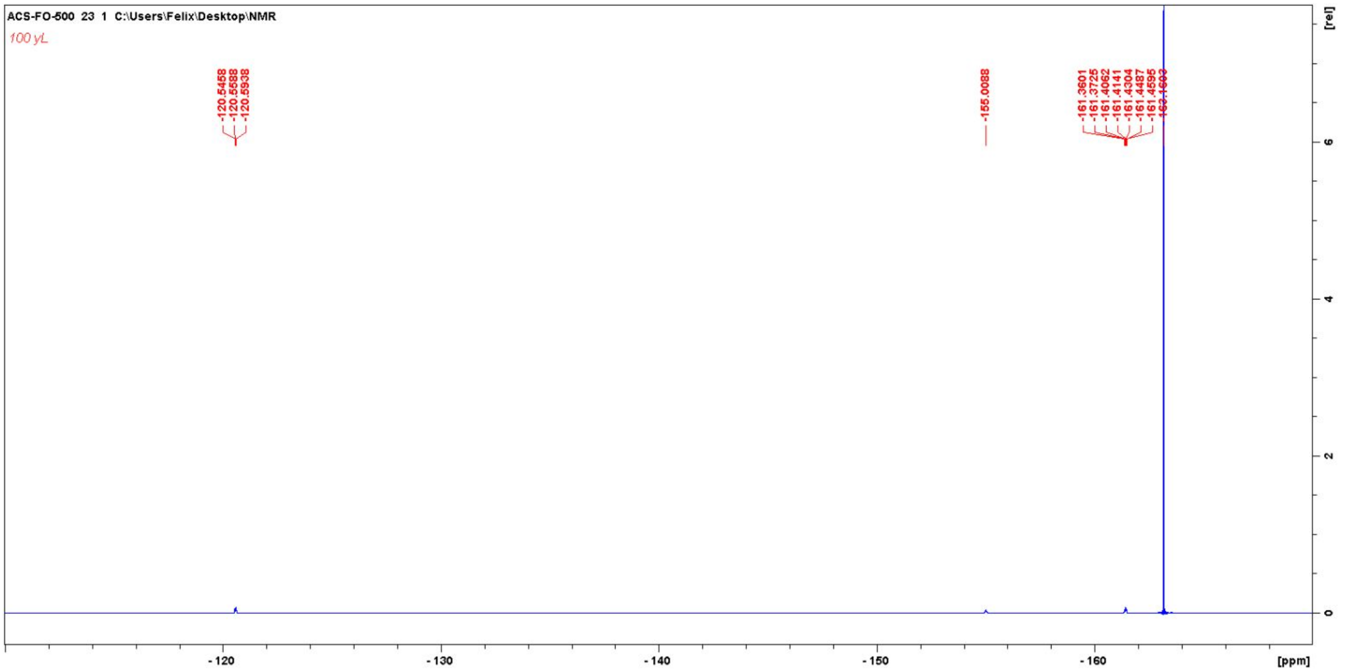

Figure S58: ^{19}F NMR spectra of pentafluoriodobenzene (host) and quinuclidine (guest, 100 µL) in cyclohexane-d12. Chemical shifts are reported relative to hexafluorobenzene.

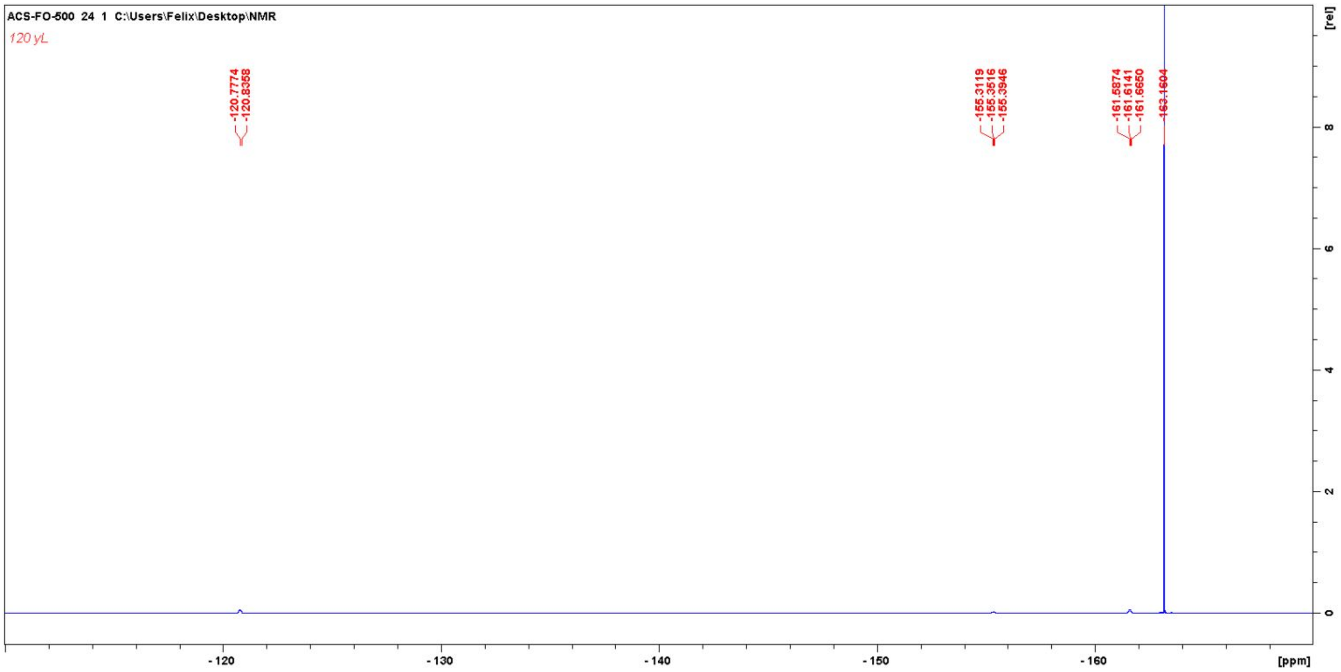

Figure S59: ^{19}F NMR spectra of pentafluoriodobenzene (host) and quinuclidine (guest, 120 µL) in cyclohexane-d12. Chemical shifts are reported relative to hexafluorobenzene.

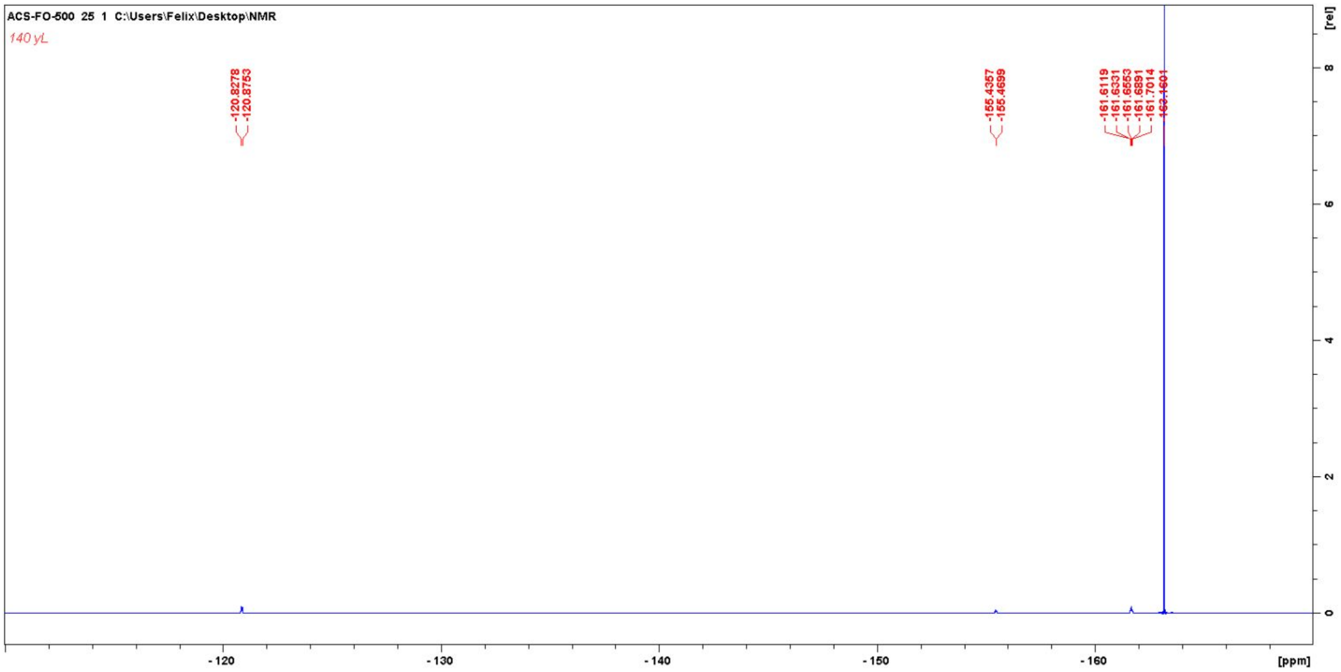

Figure S60: ^{19}F NMR spectra of pentafluoriodobenzene (host) and quinuclidine (guest, 140 µL) in cyclohexane-d12. Chemical shifts are reported relative to hexafluorobenzene.

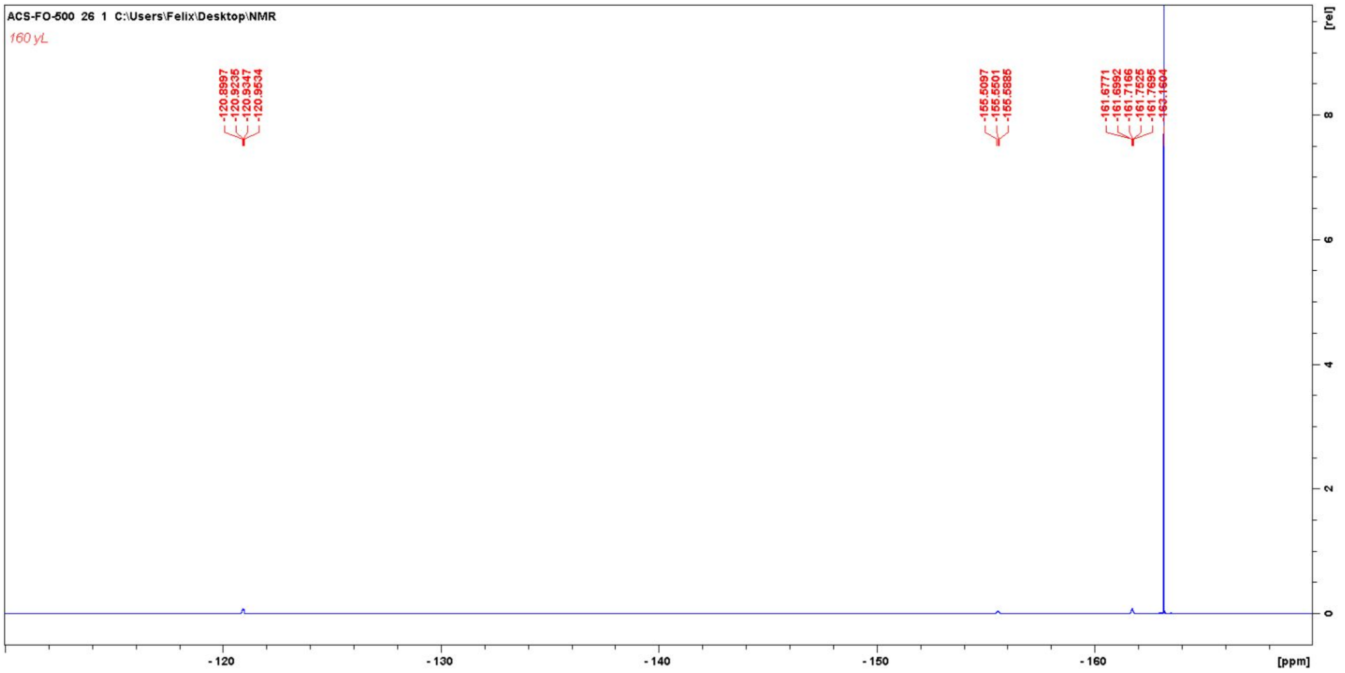

Figure S61: ^{19}F NMR spectra of pentafluoriodobenzene (host) and quinuclidine (guest, 160 µL) in cyclohexane-d12. Chemical shifts are reported relative to hexafluorobenzene.

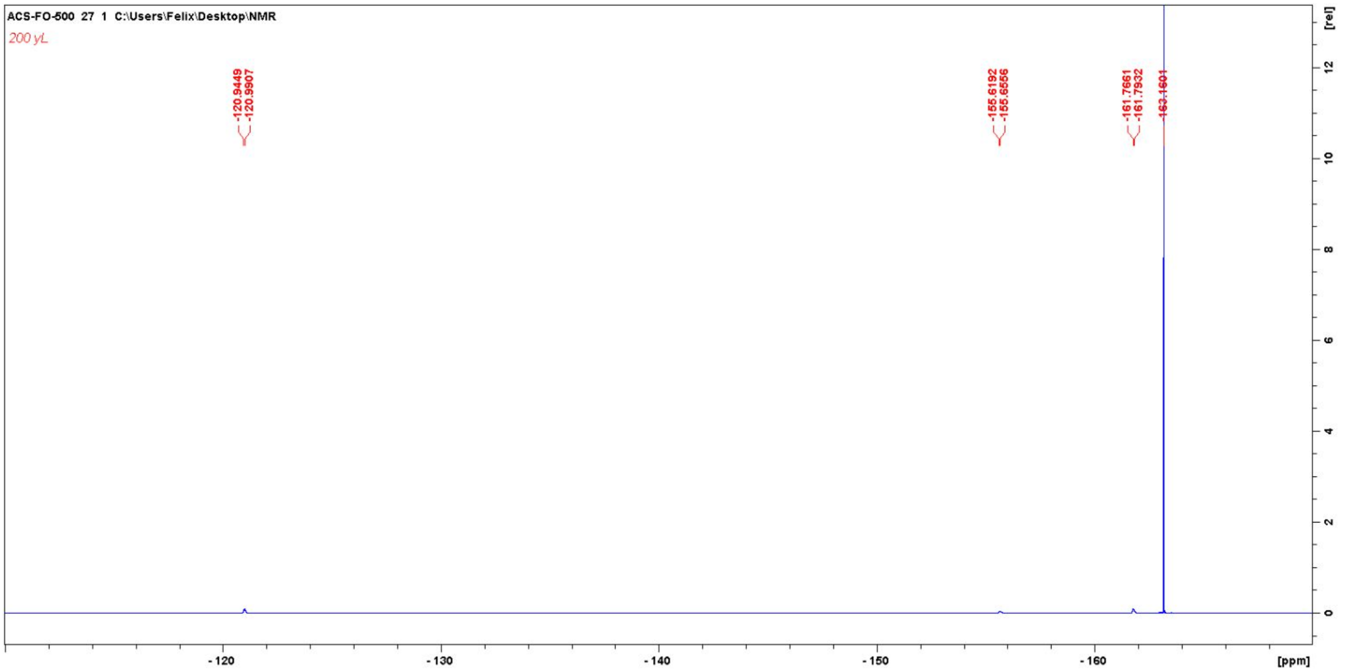

Figure S62: ^{19}F NMR spectra of pentafluoriodobenzene (host) and quinuclidine (guest, 200 µL) in cyclohexane-d12. Chemical shifts are reported relative to hexafluorobenzene.

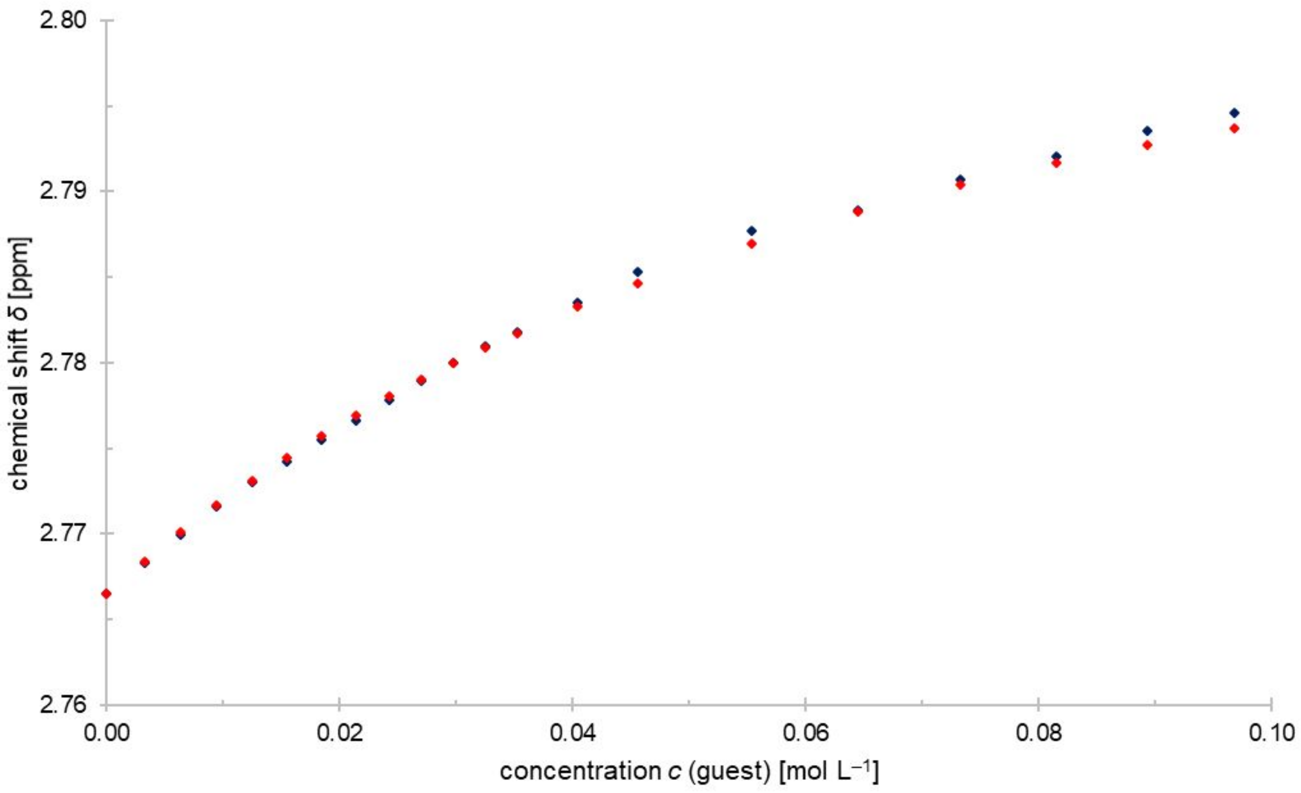

Graph S3: Titration curve of quinuclidine (host) 1,4-diiodotetrafluorobenzene (guest) mixture in C_6D_{12} at 298.13 K, where the chemical shift *δ* is plotted against the guest concentration *c*. The quinuclidine concentraion was maintained constant. The experimental values were determined from the observation of the *ortho* hydrogen atoms of the quinuclidine. A logarithmic fit could be generated. (blue = experimental data; red = theoretical data).

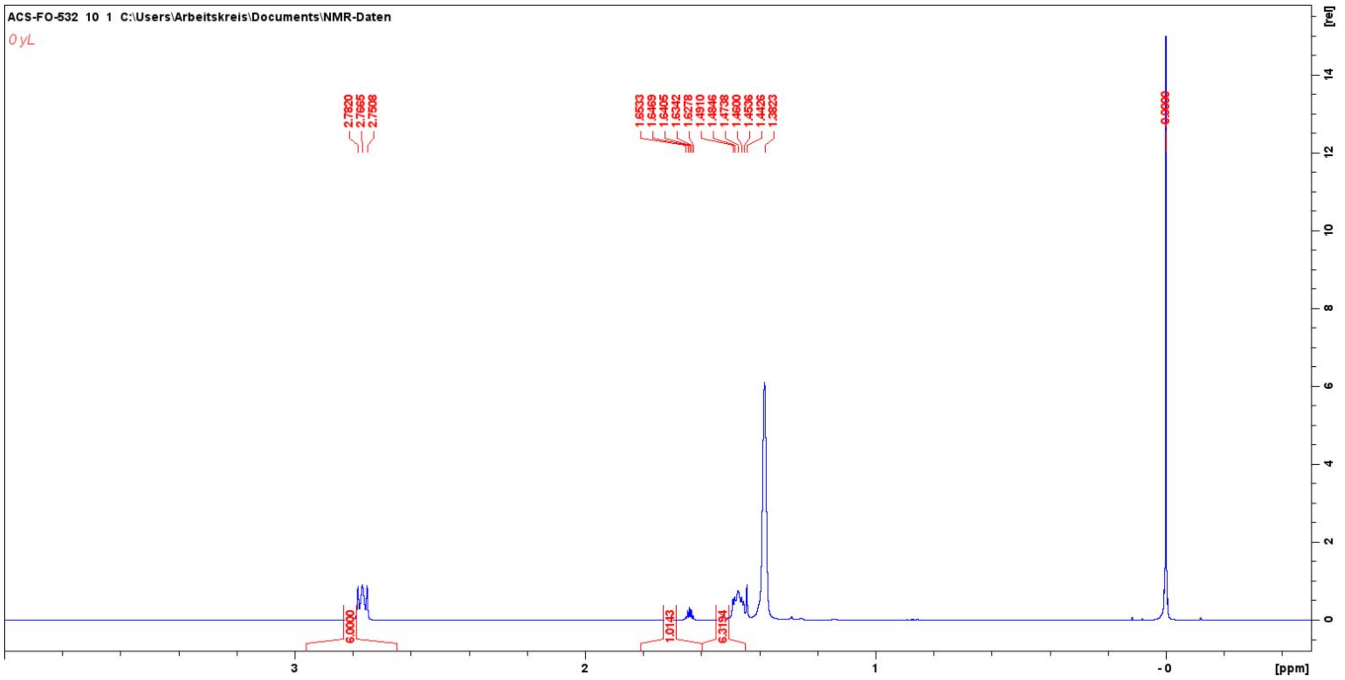

Figure S63: ^{1}H NMR spectra of quinuclidine in cyclohexane-d12. Chemical shifts are reported relative to TMS.

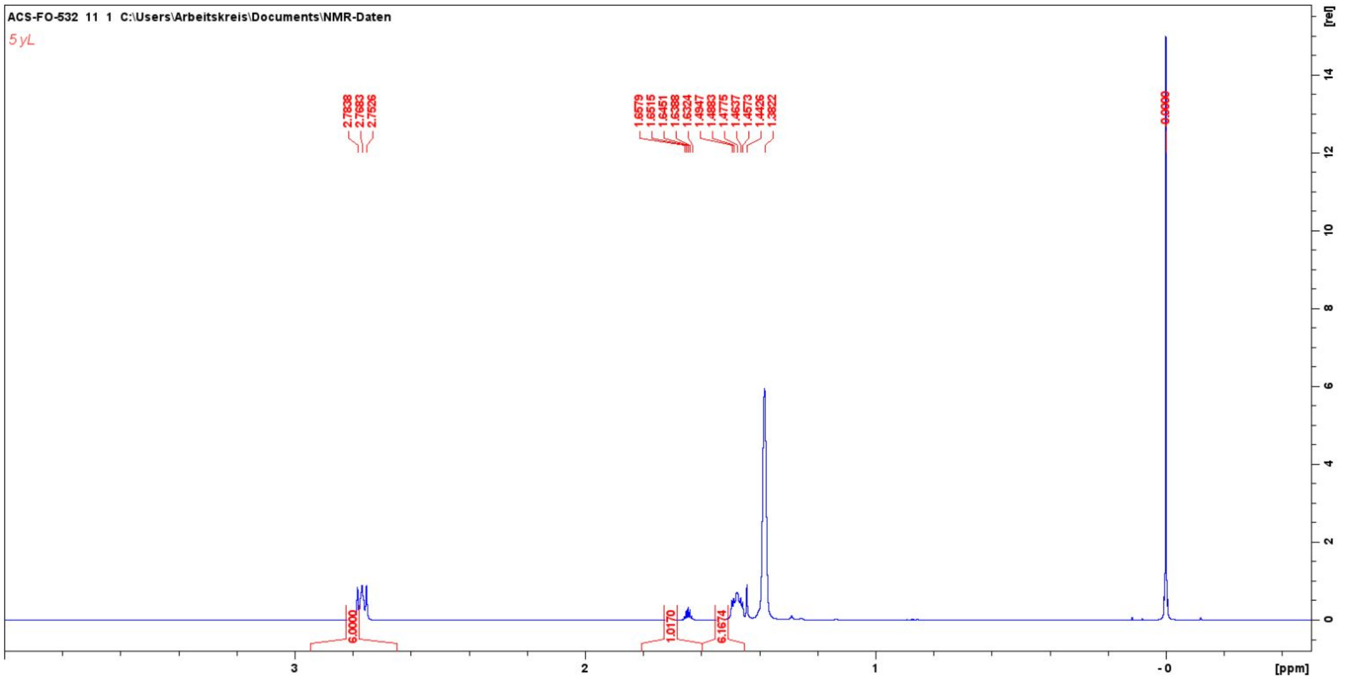

Figure S64: ^{1}H NMR spectra of quinuclidine (host) and 1,4-diiodotetrafluorenzene (guest, 5 µL) in cyclohexane-d12. Chemical shifts are reported relative to TMS.

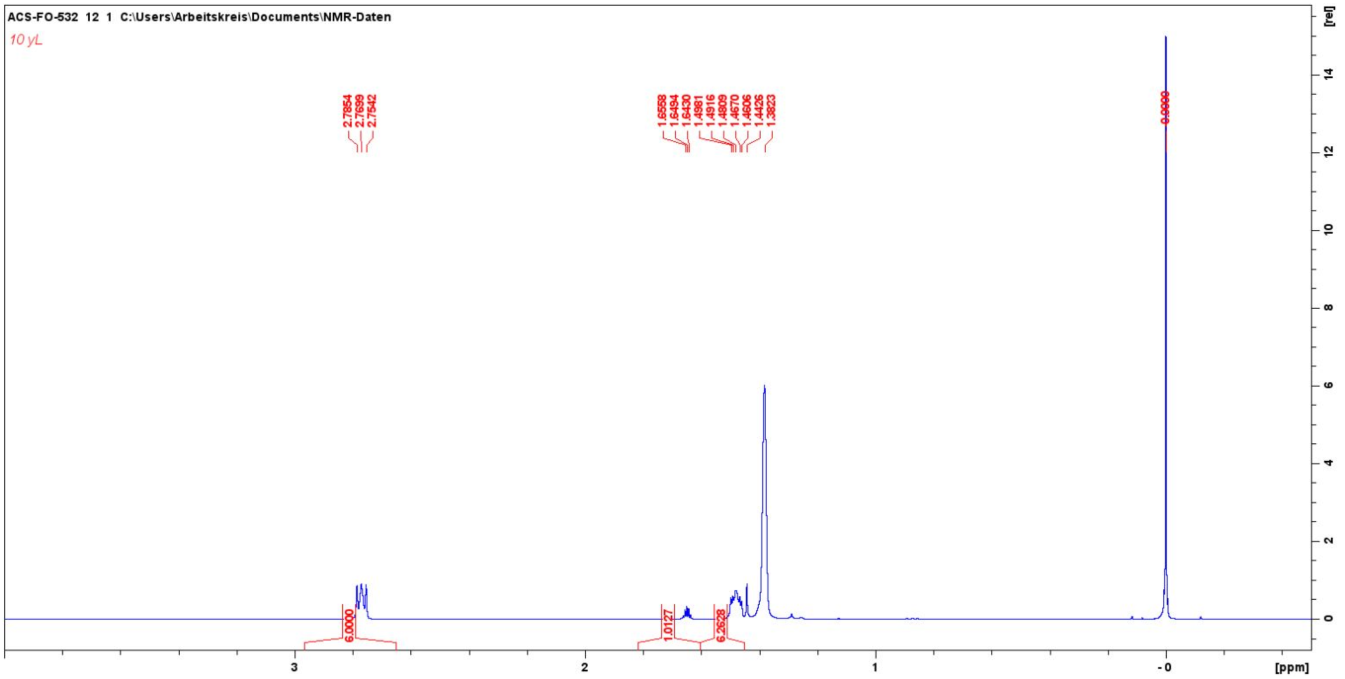

Figure S65: ^{1}H NMR spectra of quinuclidine (host) and 1,4-diiodotetrafluorenzene (guest, 10 µL) in cyclohexane-d12. Chemical shifts are reported relative to TMS.

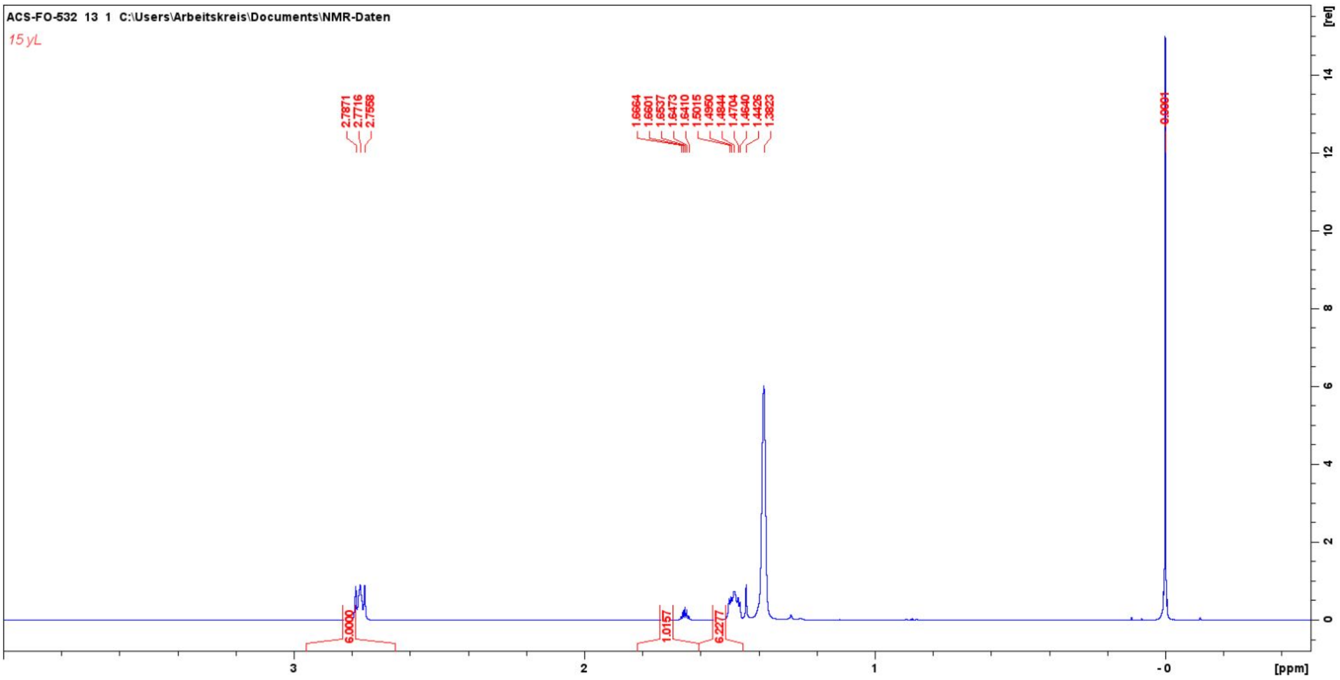

Figure S66: ^{1}H NMR spectra of quinuclidine (host) and 1,4-diiodotetrafluorenzene (guest, 15 µL) in cyclohexane-d12. Chemical shifts are reported relative to TMS.

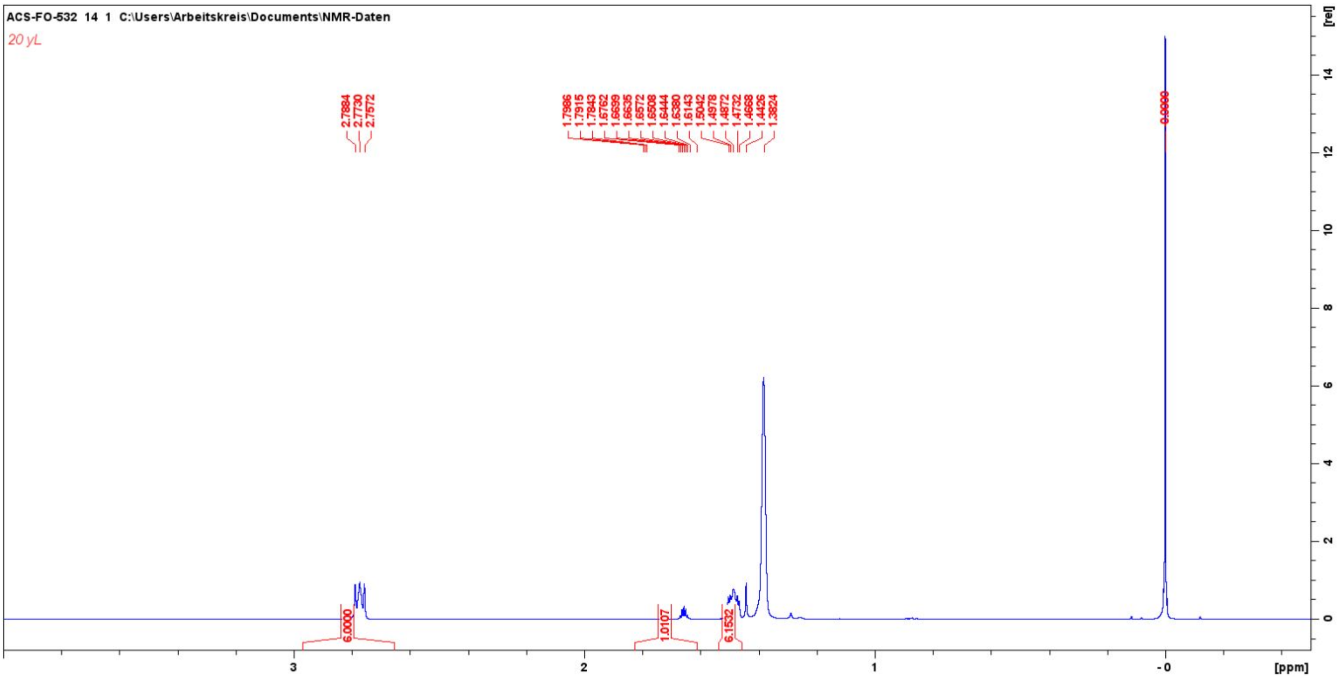

Figure S67: ^{1}H NMR spectra of quinuclidine (host) and 1,4-diiodotetrafluorenzene (guest, 20 µL) in cyclohexane-d12. Chemical shifts are reported relative to TMS.

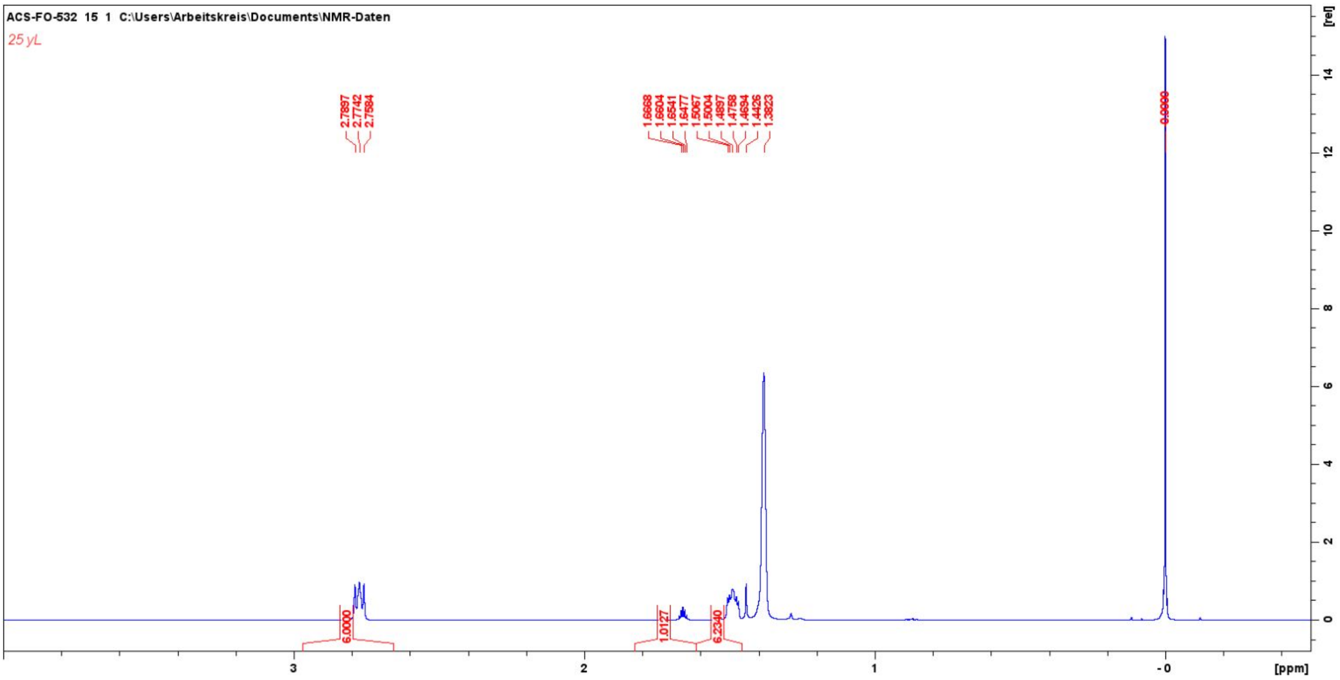

Figure S68: ^{1}H NMR spectra of quinuclidine (host) and 1,4-diiodotetrafluorenzene (guest, 25 µL) in cyclohexane-d12. Chemical shifts are reported relative to TMS.

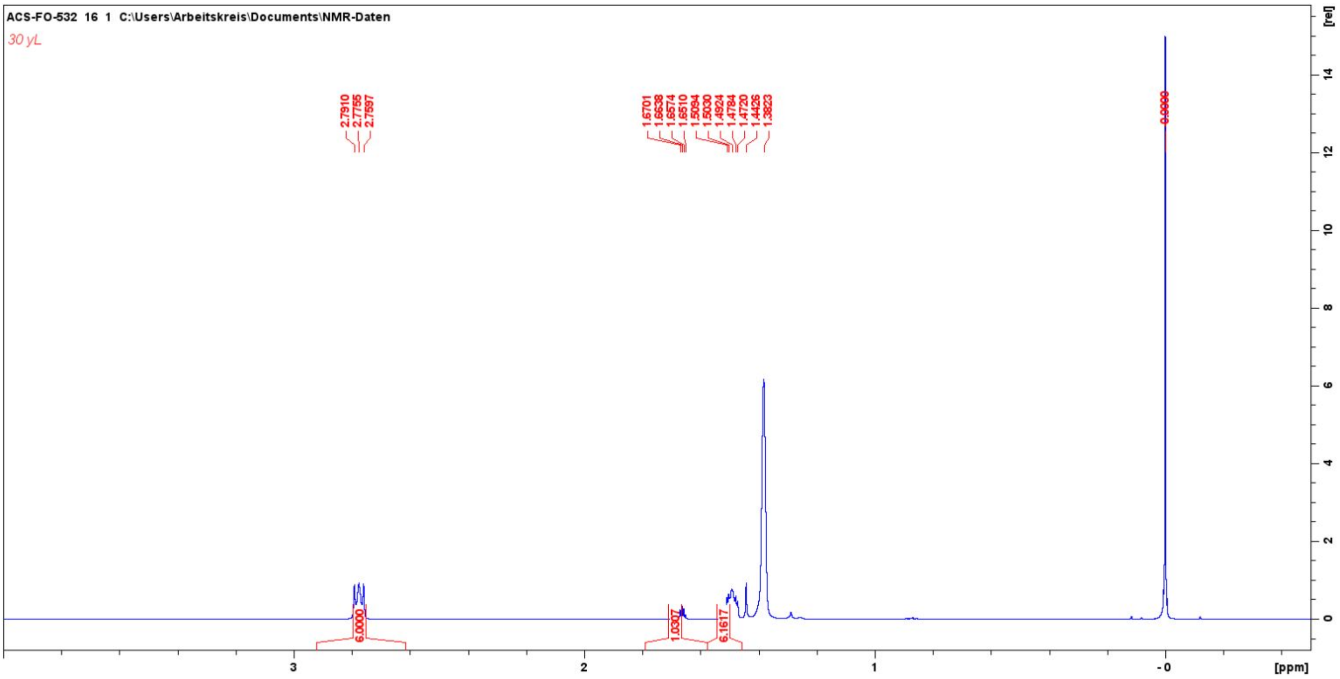

Figure S69: ^{1}H NMR spectra of quinuclidine (host) and 1,4-diiodotetrafluorenzene (guest, 30 µL) in cyclohexane-d12. Chemical shifts are reported relative to TMS.

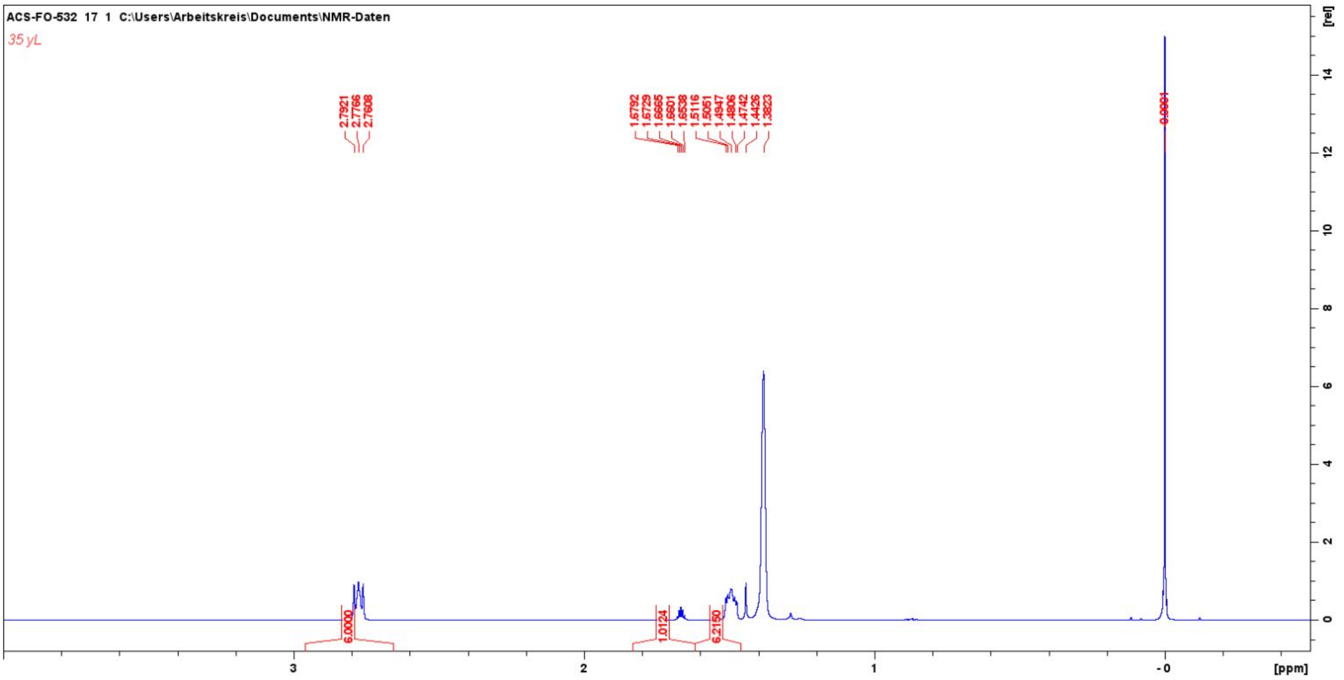

Figure S70: ^{1}H NMR spectra of quinuclidine (host) and 1,4-diiodotetrafluorenzene (guest, 35 μL) in cyclohexane-d12. Chemical shifts are reported relative to TMS.

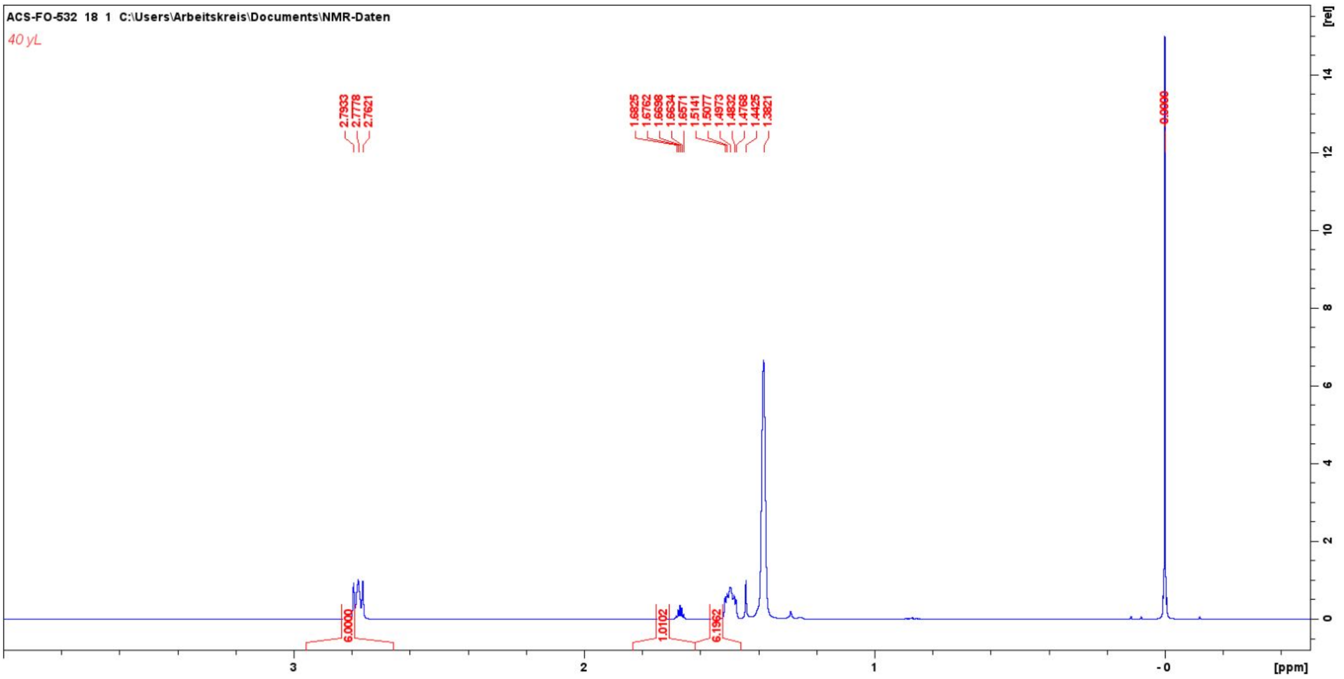

Figure S71: ^{1}H NMR spectra of quinuclidine (host) and 1,4-diiodotetrafluorenzene (guest, 40 μL) in cyclohexane-d12. Chemical shifts are reported relative to TMS.

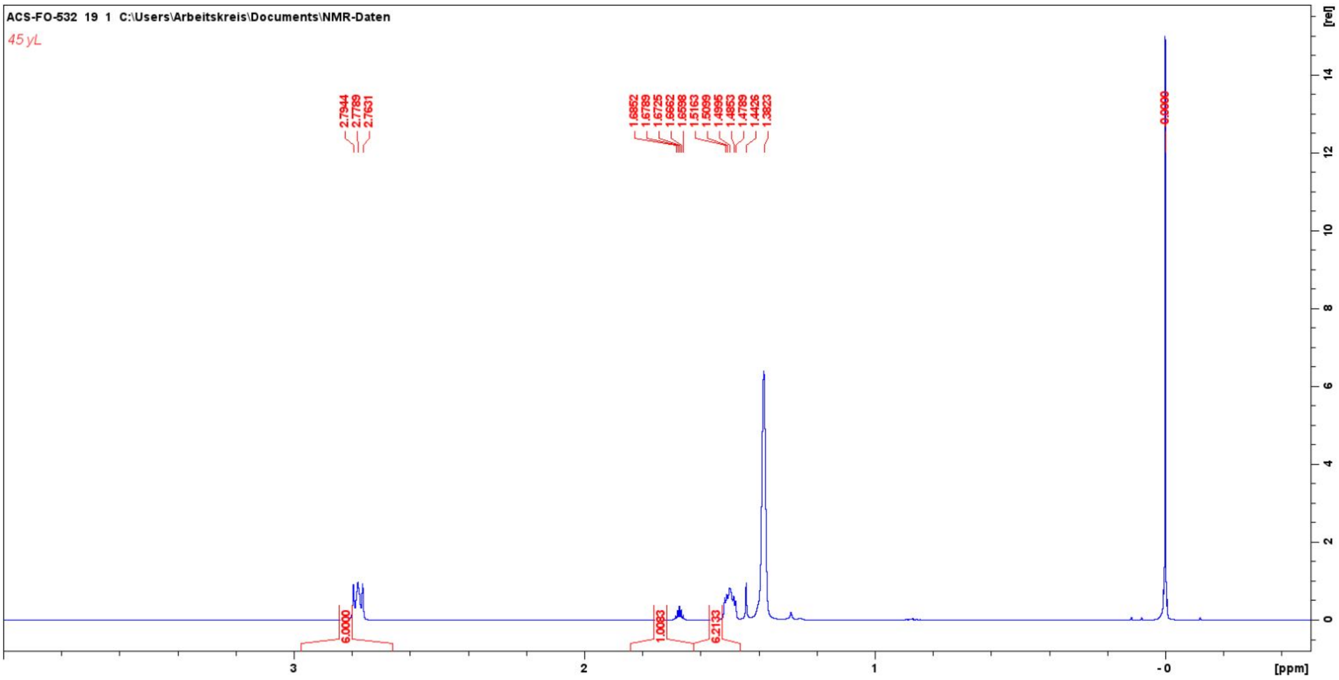

Figure S72: ^{1}H NMR spectra of quinuclidine (host) and 1,4-diiodotetrafluorenzene (guest, 45 µL) in cyclohexane-d12. Chemical shifts are reported relative to TMS.

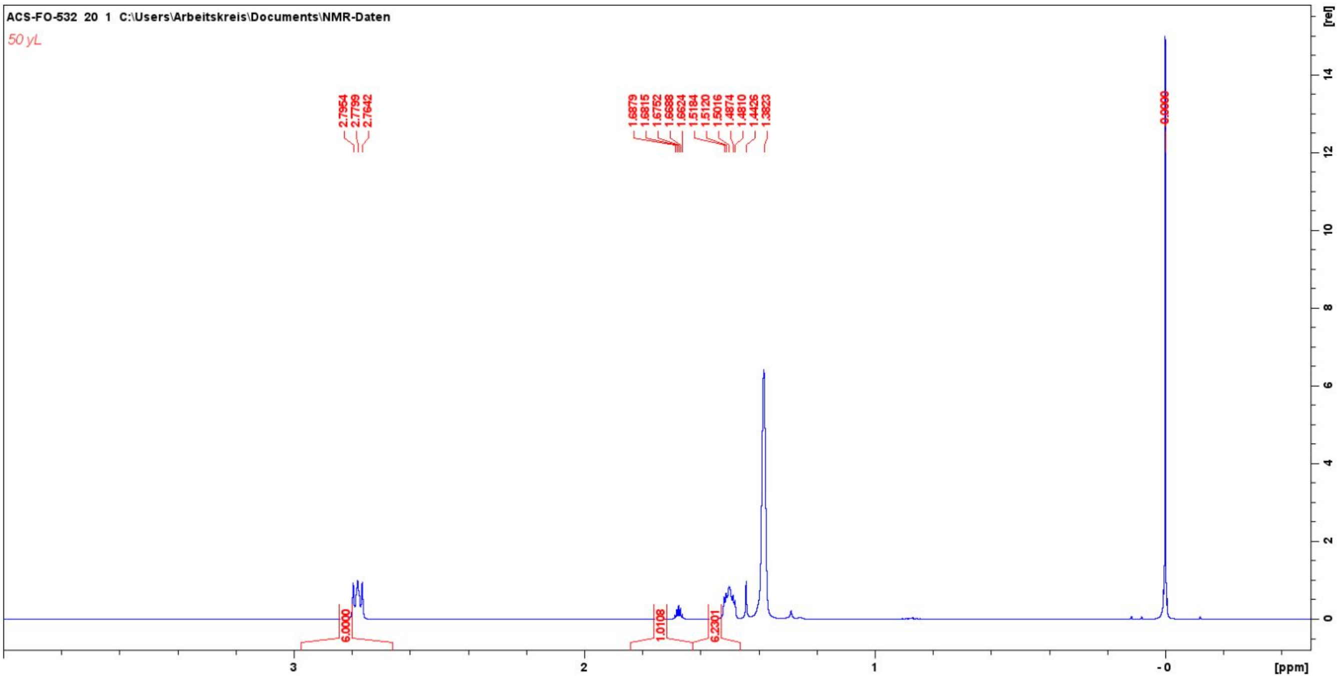

Figure S73: ^{1}H NMR spectra of quinuclidine (host) and 1,4-diiodotetrafluorenzene (guest, 50 µL) in cyclohexane-d12. Chemical shifts are reported relative to TMS.

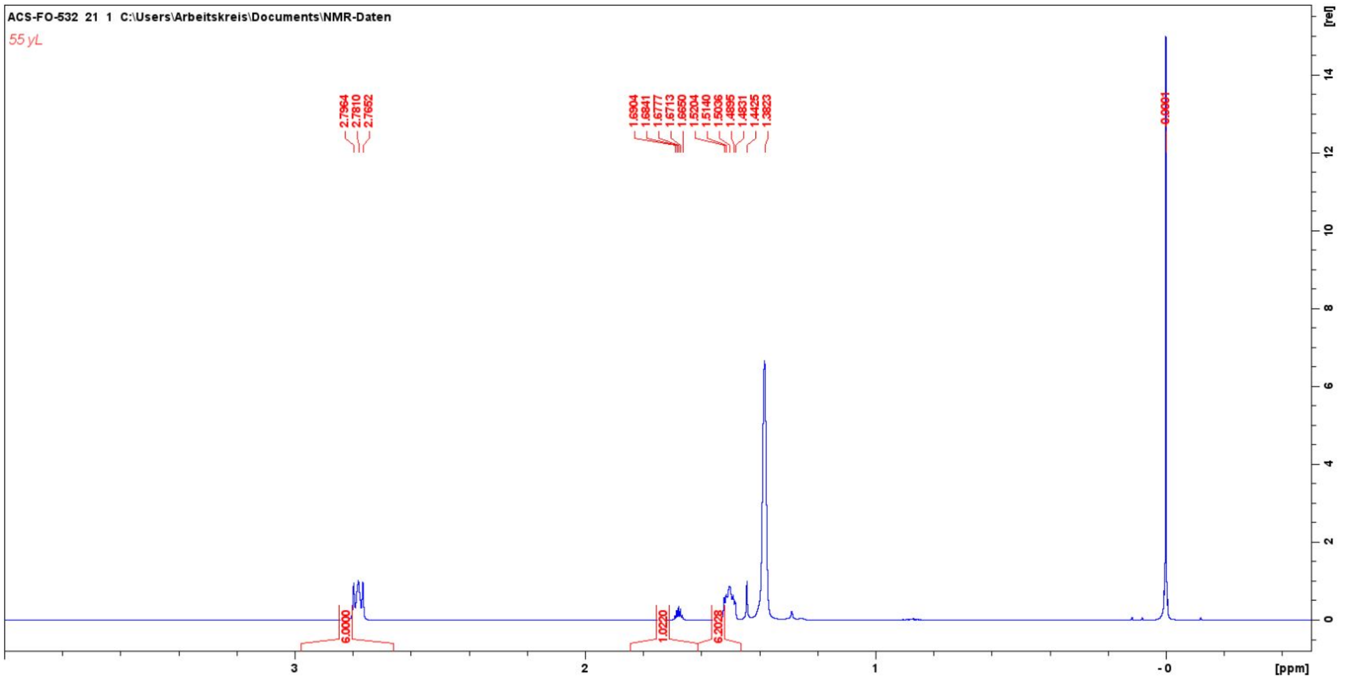

Figure S74: ^{1}H NMR spectra of quinuclidine (host) and 1,4-diiodotetrafluorenzene (guest, 55 µL) in cyclohexane-d12. Chemical shifts are reported relative to TMS.

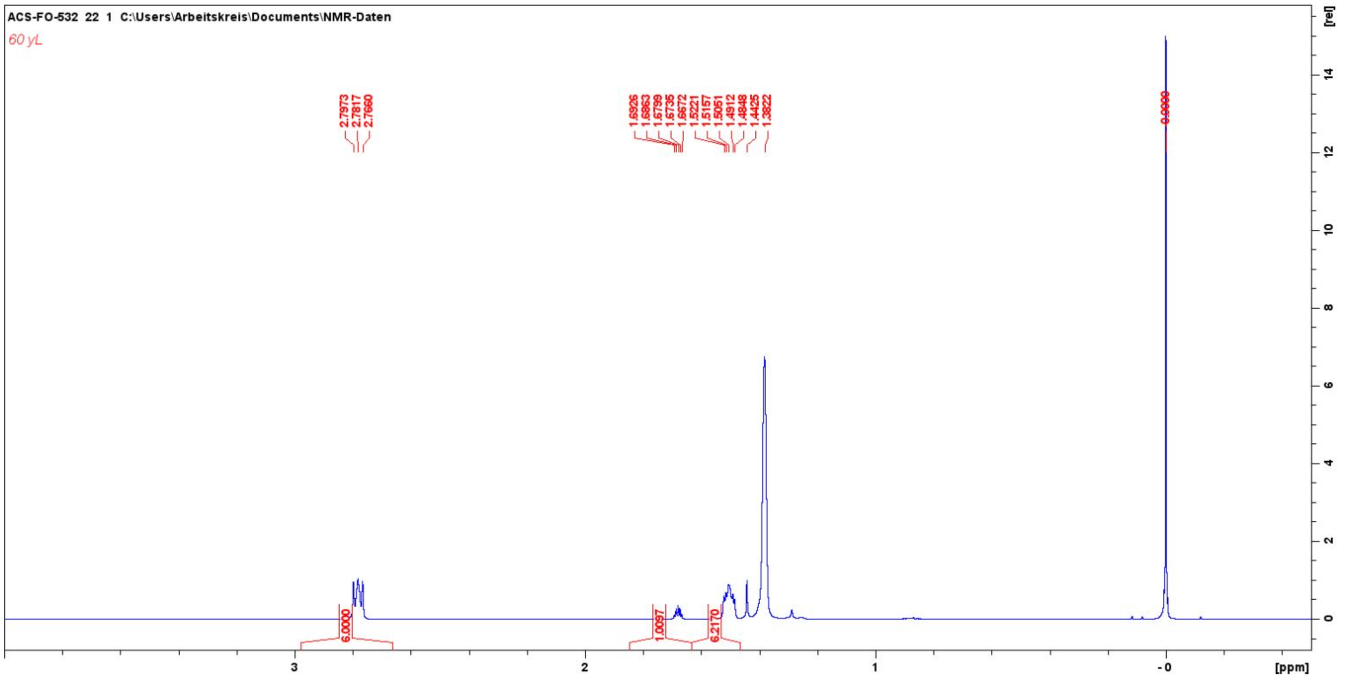

Figure S75: ^{1}H NMR spectra of quinuclidine (host) and 1,4-diiodotetrafluorenzene (guest, 60 µL) in cyclohexane-d12. Chemical shifts are reported relative to TMS.

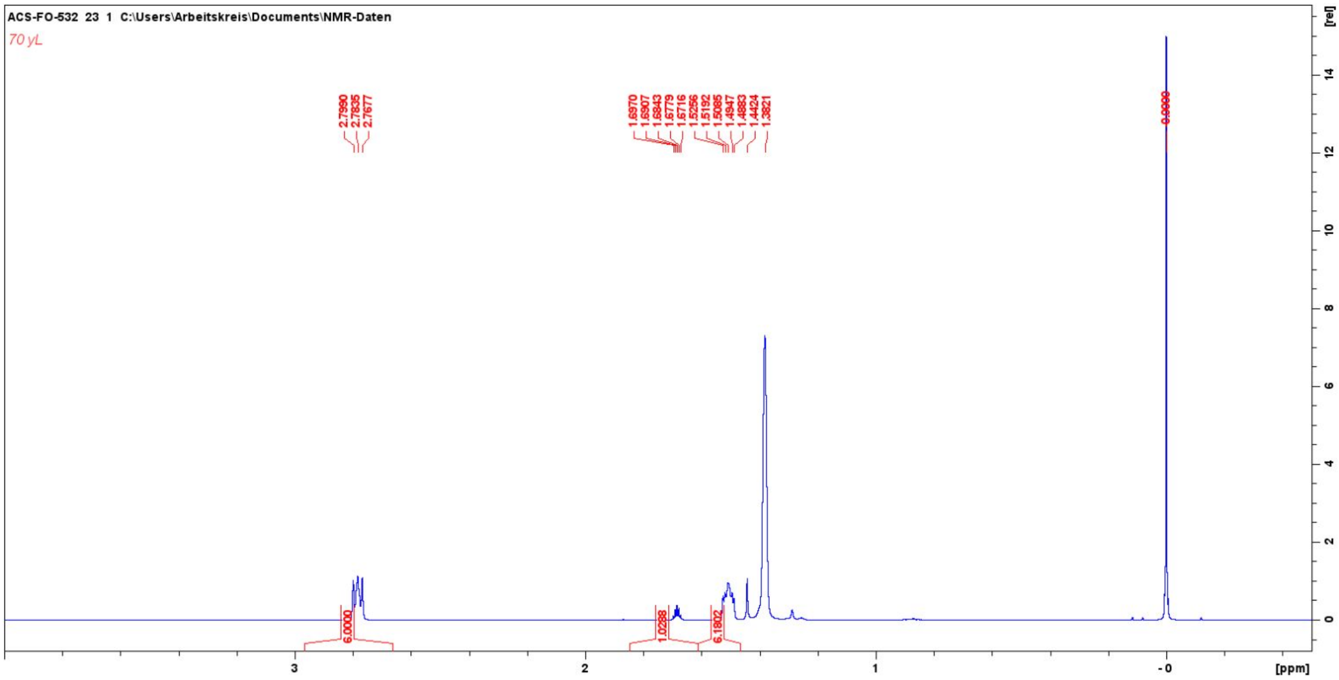

Figure S76: ^{1}H NMR spectra of quinuclidine (host) and 1,4-diiodotetrafluorenzene (guest, 70 μL) in cyclohexane-d12. Chemical shifts are reported relative to TMS.

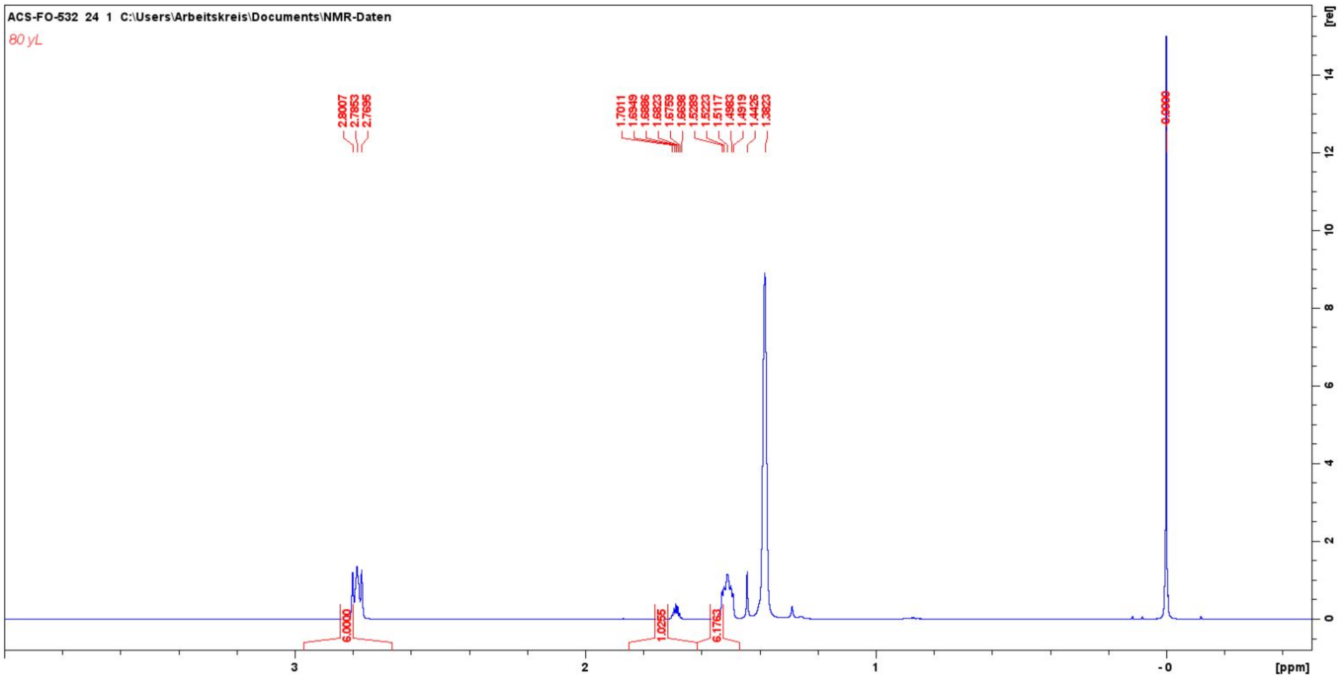

Figure S77: ^{1}H NMR spectra of quinuclidine (host) and 1,4-diiodotetrafluorenzene (guest, 80 μL) in cyclohexane-d12. Chemical shifts are reported relative to TMS.

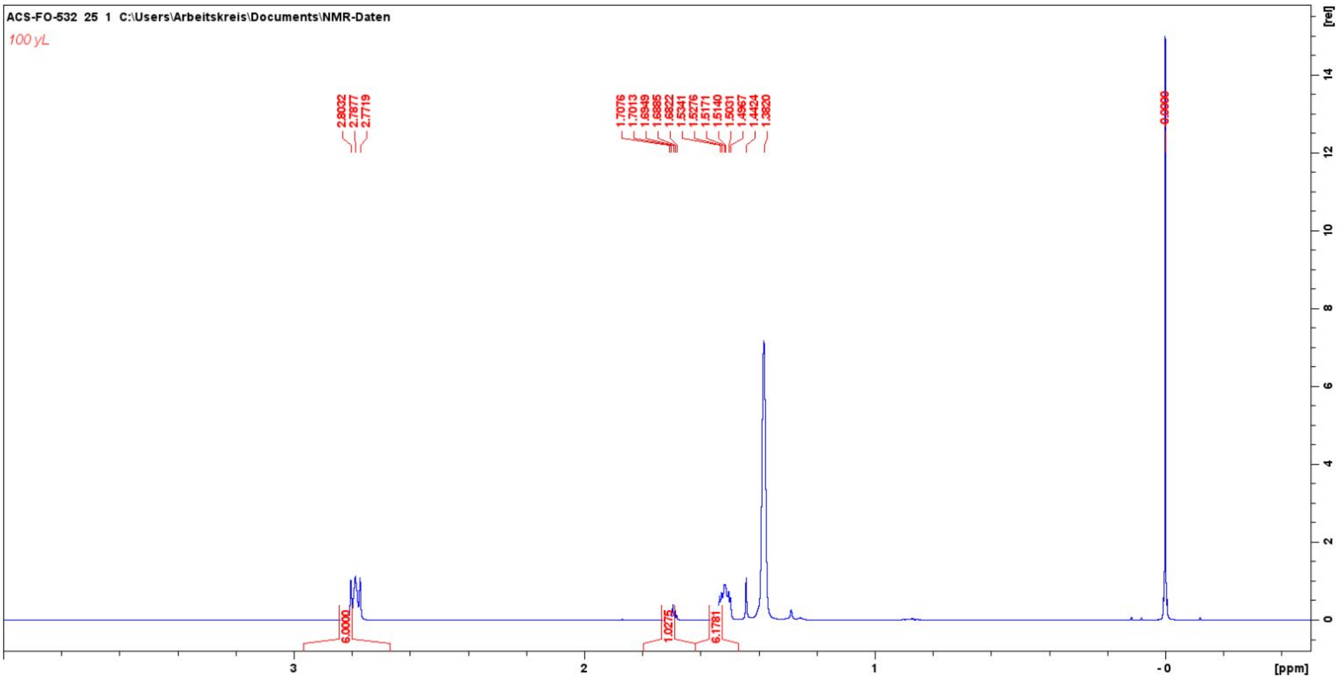

Figure S78: ^{1}H NMR spectra of quinuclidine (host) and 1,4-diiodotetrafluorenzene (guest, 100 µL) in cyclohexane-d12. Chemical shifts are reported relative to TMS.

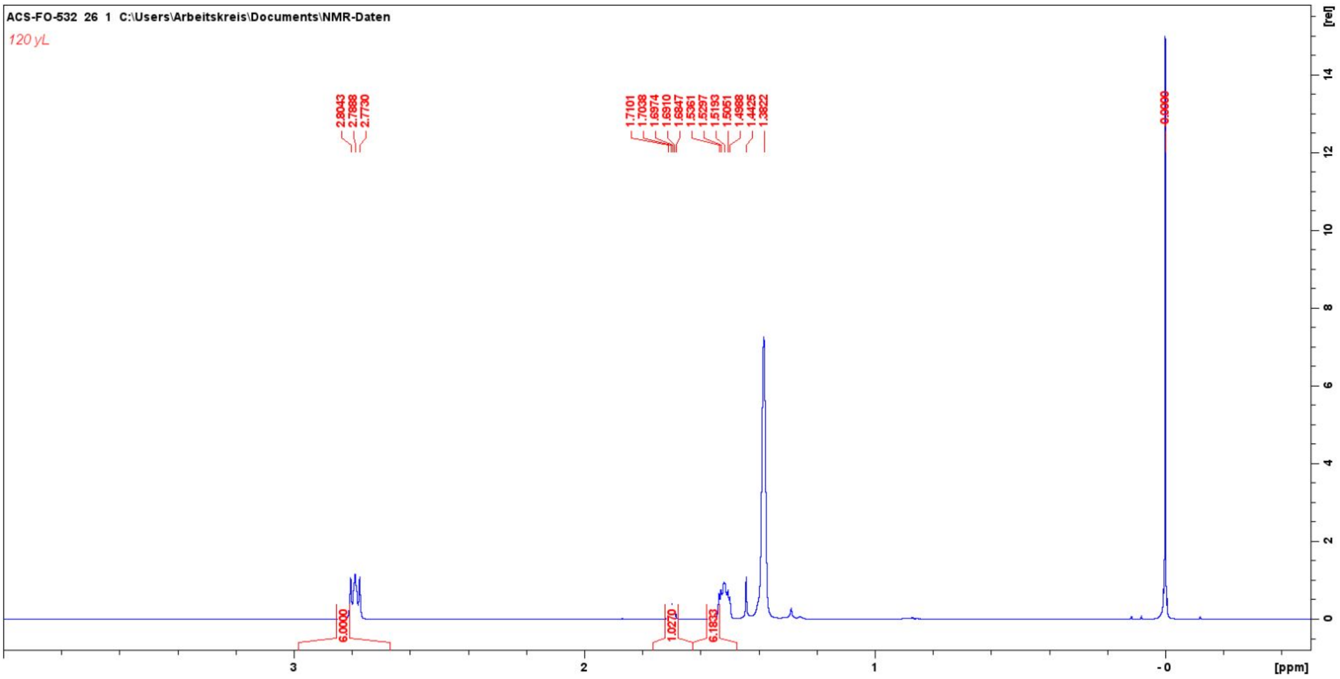

Figure S79: ^{1}H NMR spectra of quinuclidine (host) and 1,4-diiodotetrafluorenzene (guest, 120 µL) in cyclohexane-d12. Chemical shifts are reported relative to TMS.

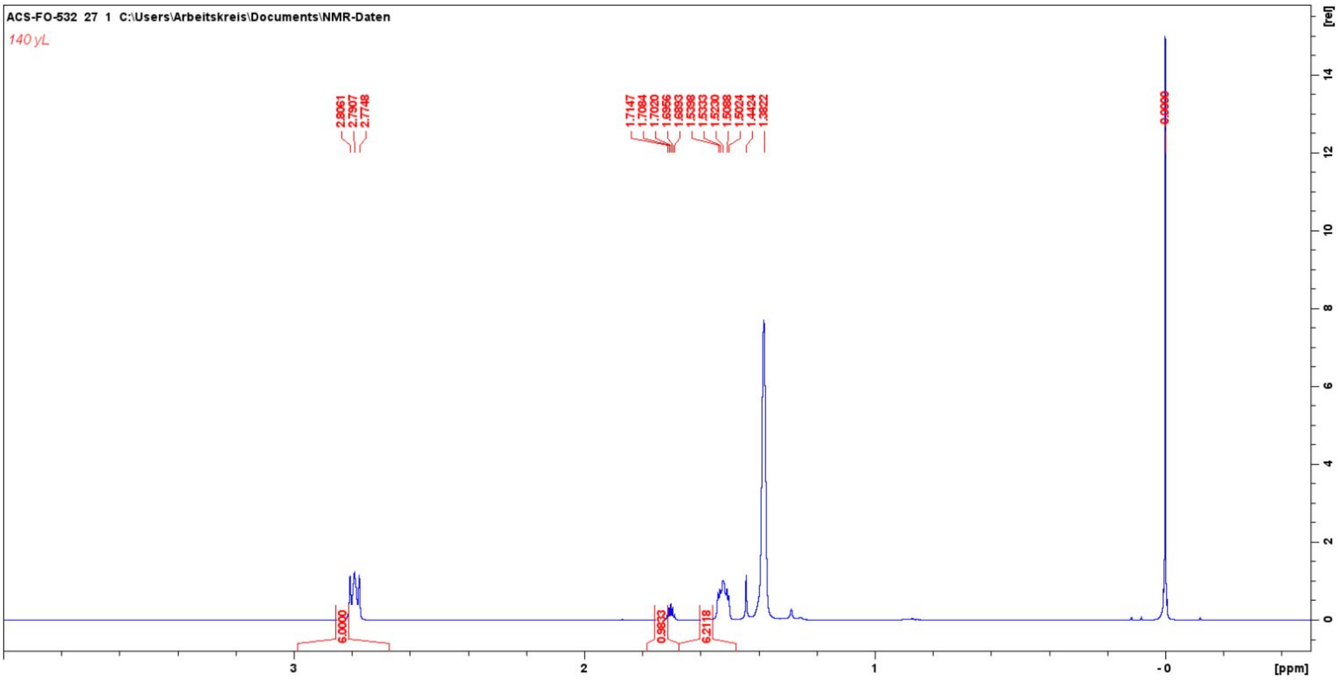

Figure S80: ^{1}H NMR spectra of quinuclidine (host) and 1,4-diiodotetrafluorenzene (guest, 140 µL) in cyclohexane-d12. Chemical shifts are reported relative to TMS.

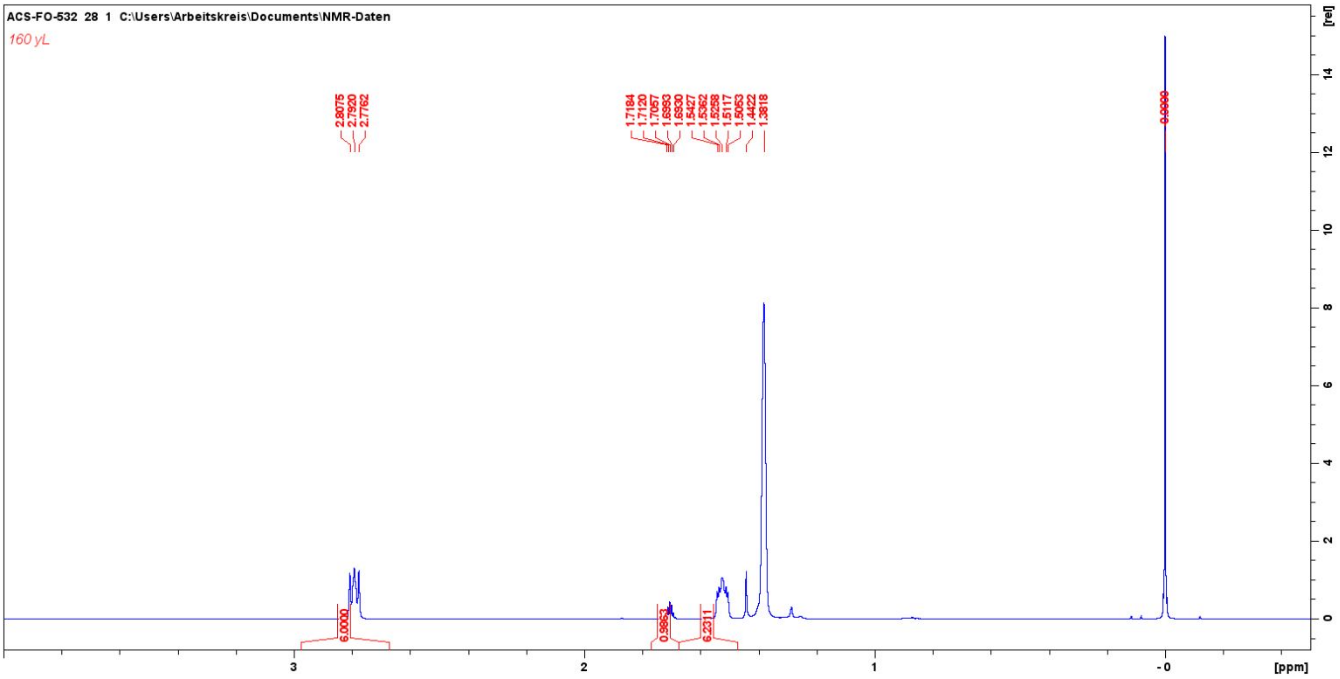

Figure S81: ^{1}H NMR spectra of quinuclidine (host) and 1,4-diiodotetrafluorenzene (guest, 160 µL) in cyclohexane-d12. Chemical shifts are reported relative to TMS.

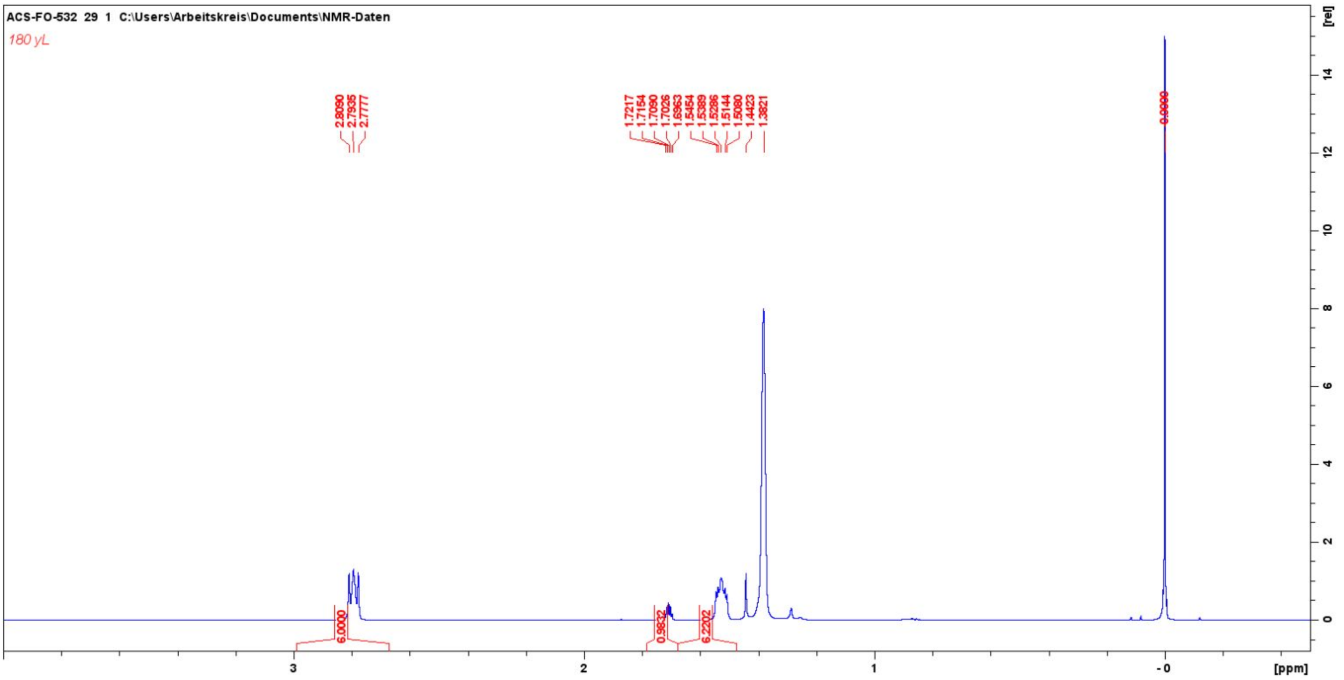

Figure S82: ^{1}H NMR spectra of quinuclidine (host) and 1,4-diiodotetrafluorenzene (guest, 180 µL) in cyclohexane-d12. Chemical shifts are reported relative to TMS.

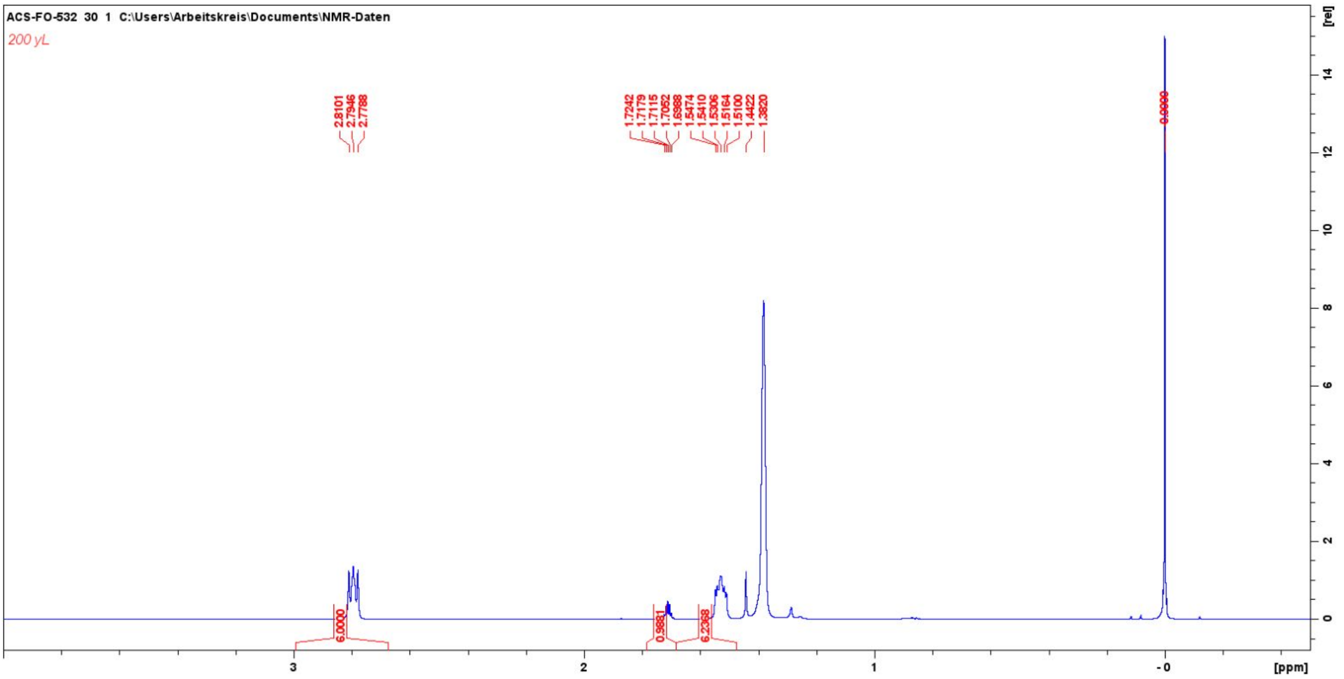

Figure S83: ^{1}H NMR spectra of quinuclidine (host) and 1,4-diiodotetrafluorenzene (guest, 200 µL) in cyclohexane-d12. Chemical shifts are reported relative to TMS.

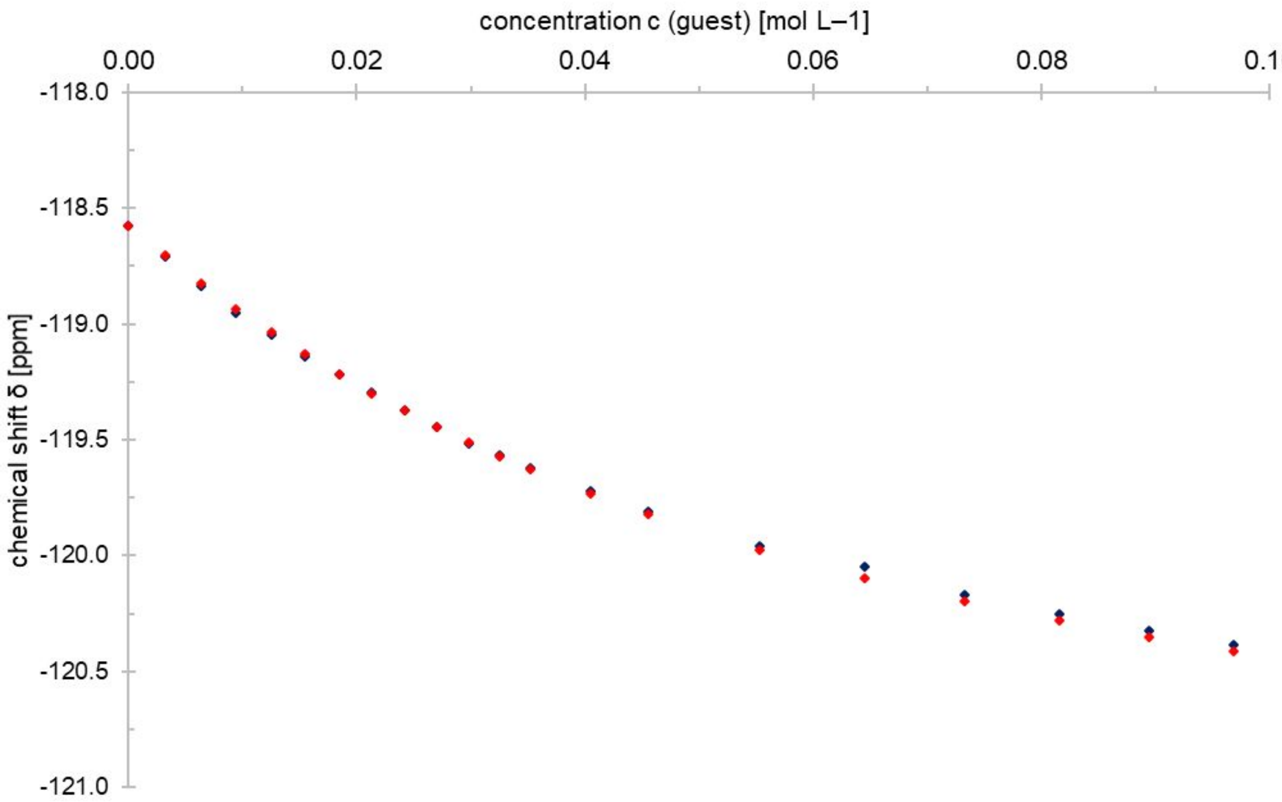

Graph S4: Titration curve of 1,4-diiodotetrafluorobenzene (host) quinuclidine (guest) mixture in C_6D_{12} at 298.13 K, where the chemical shift *δ* is plotted against the guest concentration *c*. The 1,4-diiodotetrafluorobenzene concentraion was maintained constant. The experimental values were determined from the observation of the fluorine atoms of the 1,4-diiodotetrafluorobenzene. A logarithmic fit could be generated. (blue = experimental data; red = theoretical data).

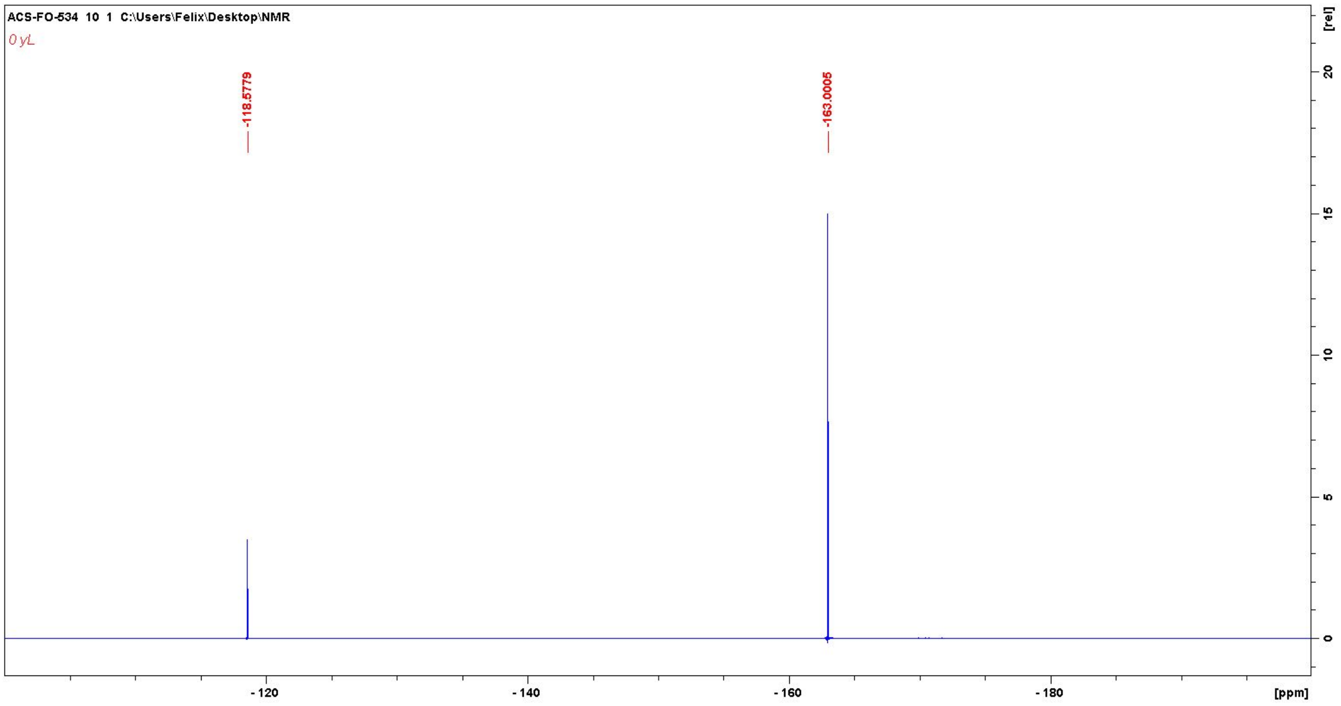

Figure S84: ^{19}F NMR spectra of 1,4-diiodtetrafluorobenzene (host) in cyclohexane-d12. Chemical shifts are reported relative to hexafluorobenzene.

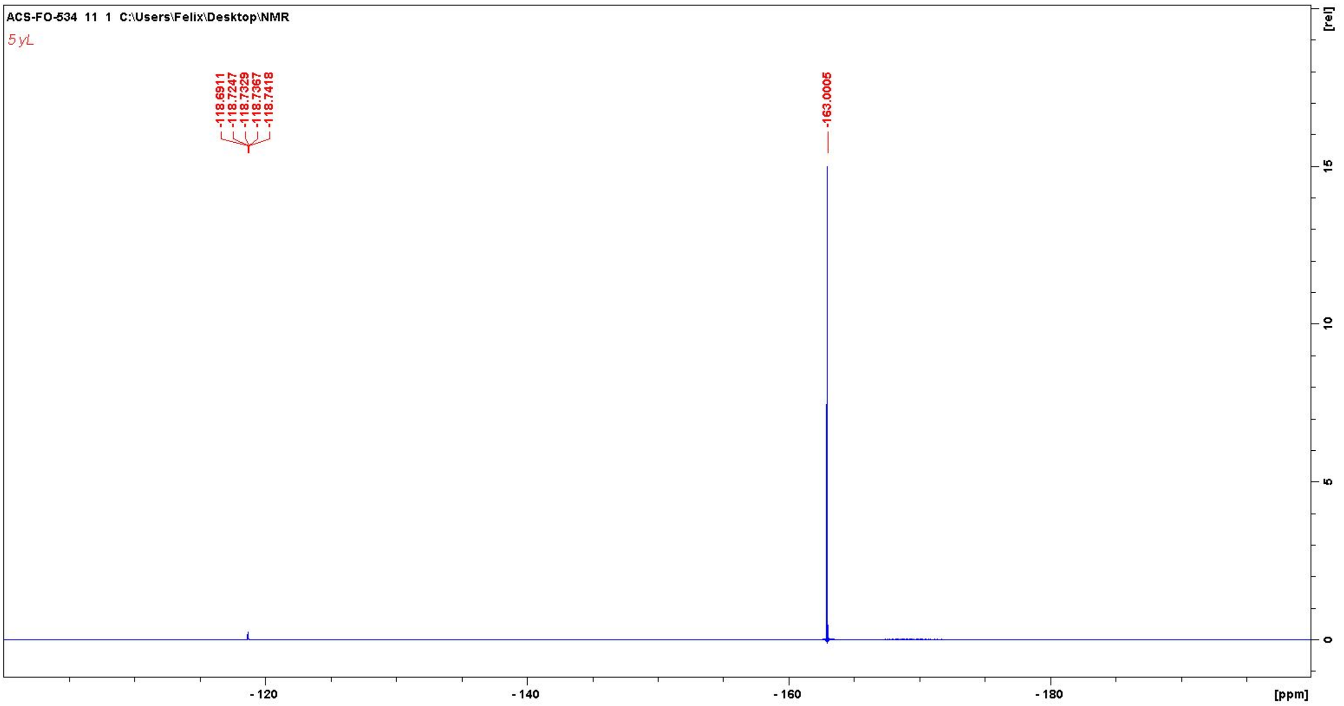

Figure S85: ^{19}F NMR spectra of 1,4-diiodtetrafluorobenzene (host) and quinuclidine (guest, 5 µL) in cyclohexane-d12. Chemical shifts are reported relative to hexafluorobenzene.

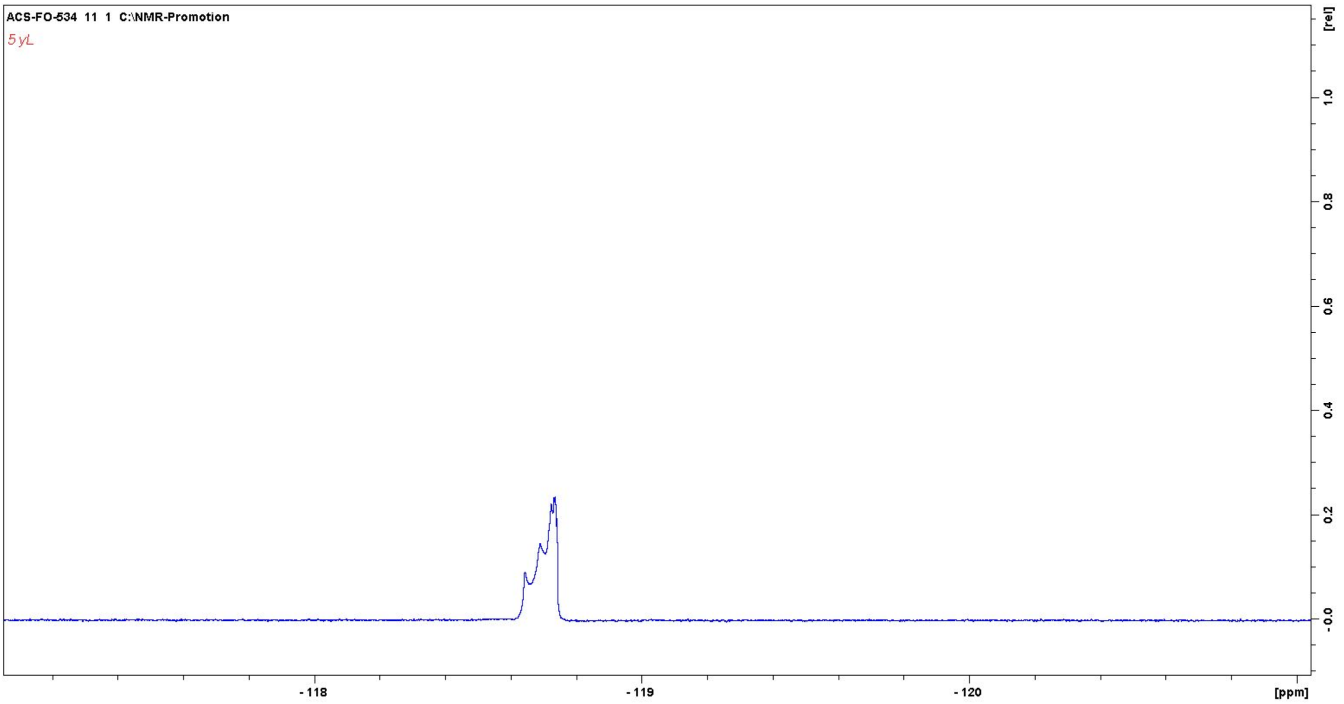

Figure S86: Zoom in on the indefinable multiplet after the addition of quinuclidine in the ^{19}F NMR spectra of 1,4-diiodtetrafluorobenzene (host) and quinuclidine (guest, 5 µL) in cyclohexane-d12. Chemical shifts are reported relative to hexafluorobenzene.

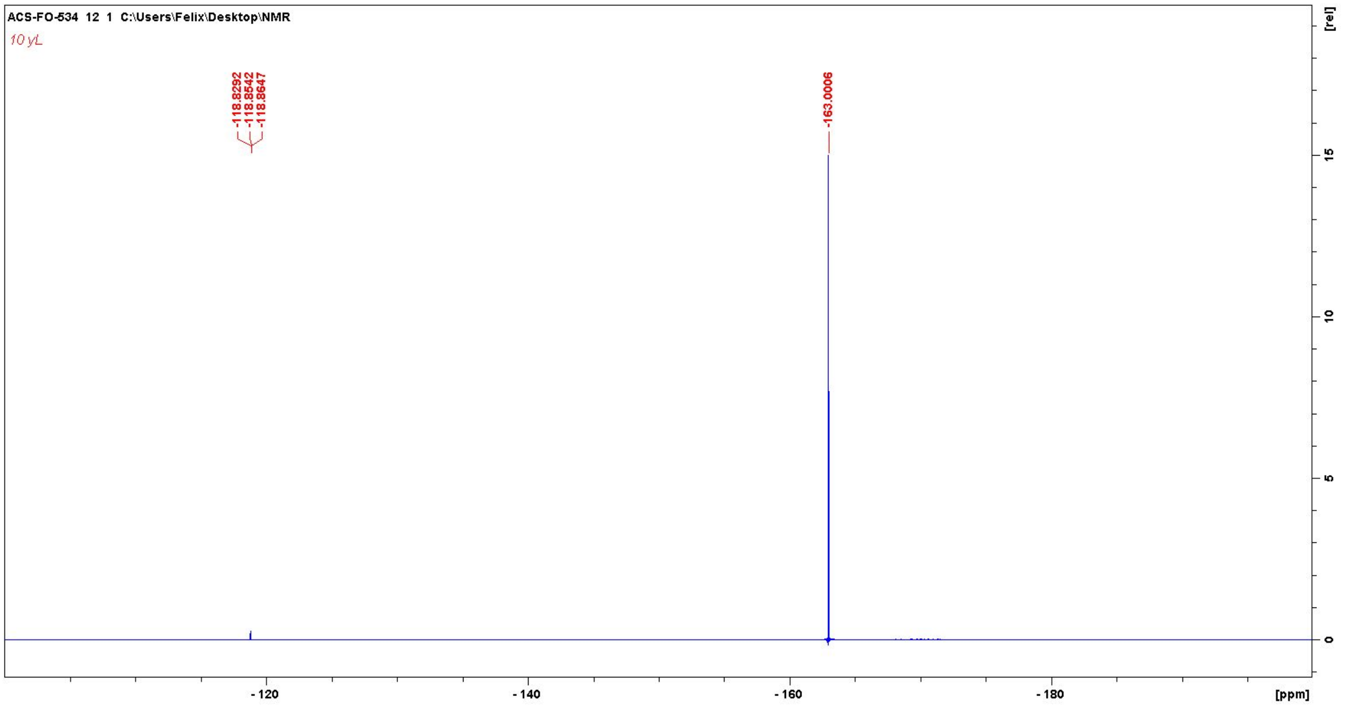

Figure S87: ^{19}F NMR spectra of 1,4-diiodtetrafluorobenzene (host) and quinuclidine (guest, 10 µL) in cyclohexane-d12. Chemical shifts are reported relative to hexafluorobenzene.

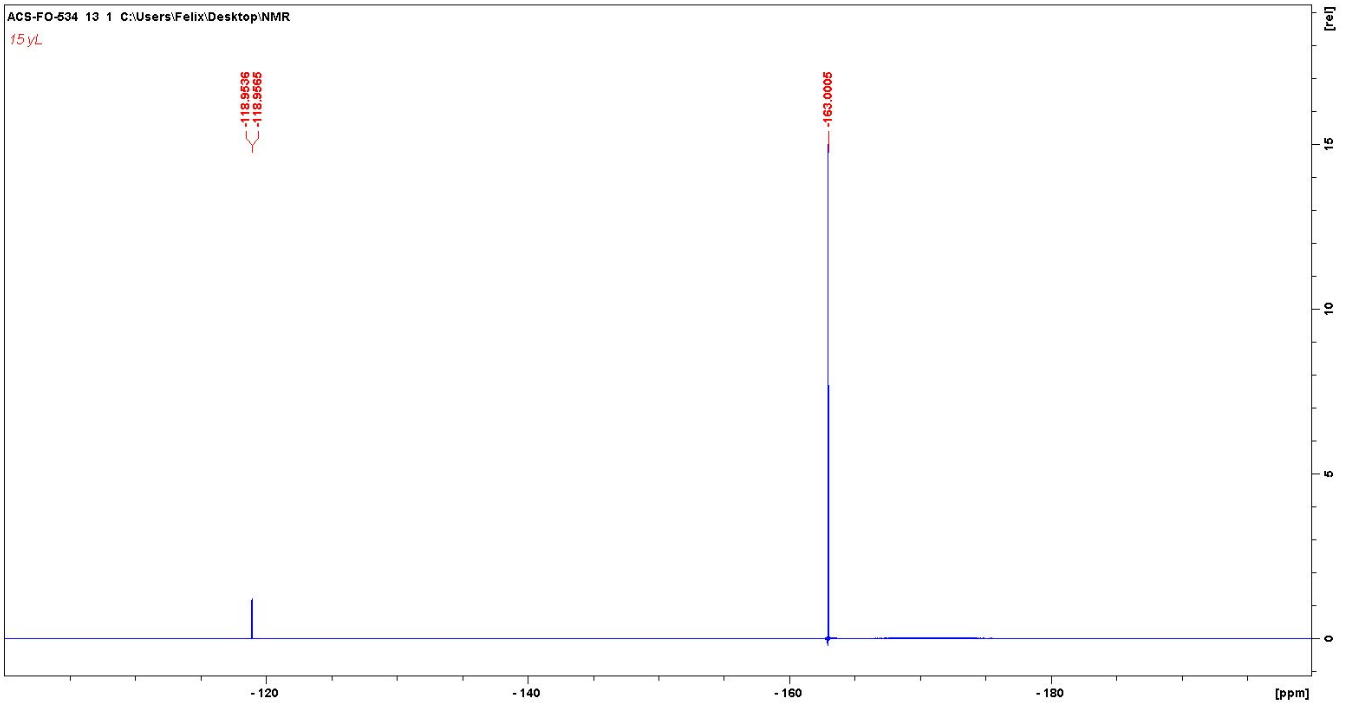

Figure S88: ^{19}F NMR spectra of 1,4-diiodtetrafluorobenzene (host) and quinuclidine (guest, 15 µL) in cyclohexane-d12. Chemical shifts are reported relative to hexafluorobenzene.

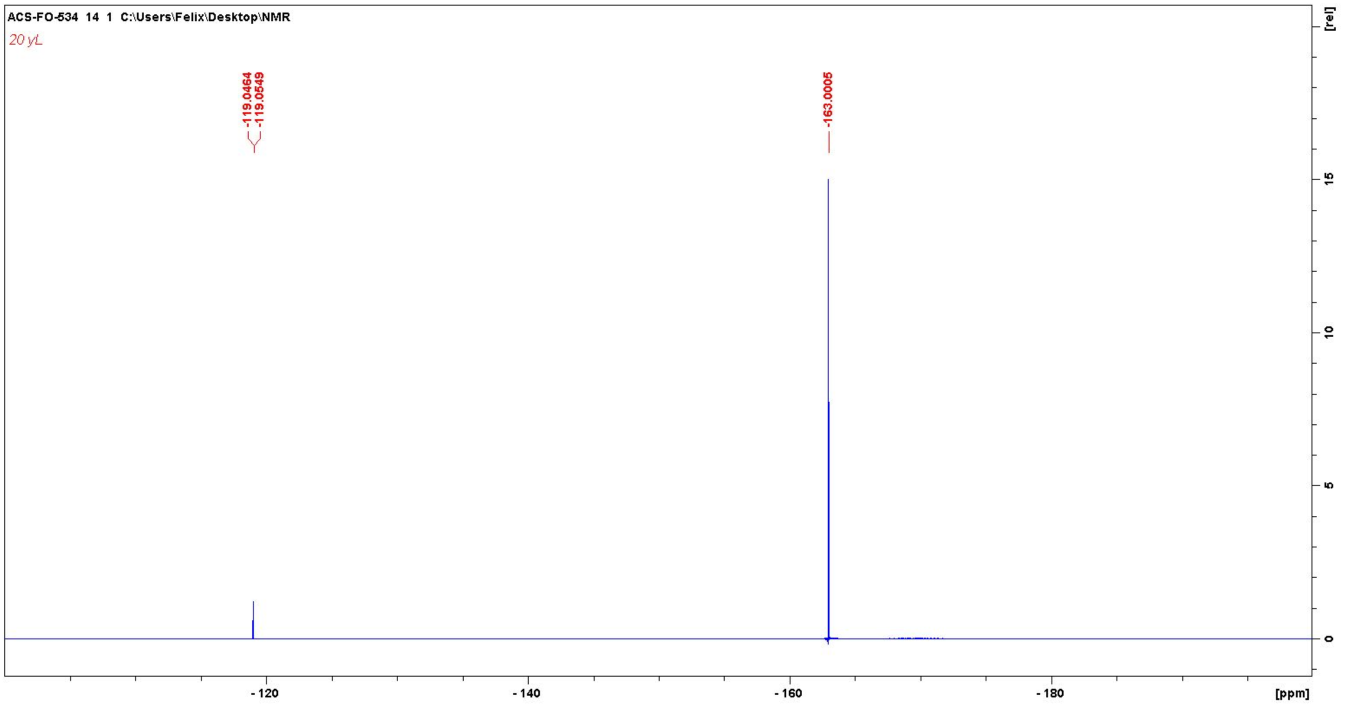

Figure S89: ^{19}F NMR spectra of 1,4-diiodtetrafluorobenzene (host) and quinuclidine (guest, 20 µL) in cyclohexane-d12. Chemical shifts are reported relative to hexafluorobenzene.

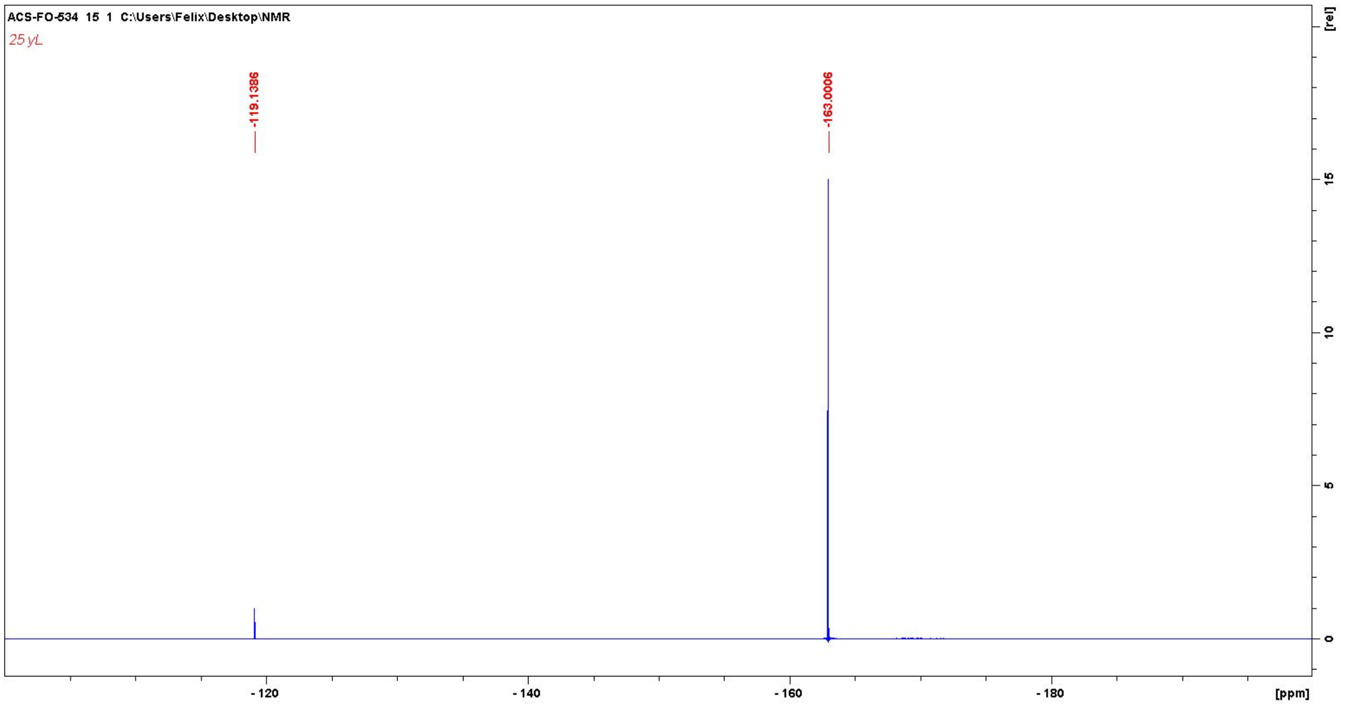

Figure S90: ^{19}F NMR spectra of 1,4-diiodtetrafluorobenzene (host) and quinuclidine (guest, 25 µL) in cyclohexane-d12. Chemical shifts are reported relative to hexafluorobenzene.

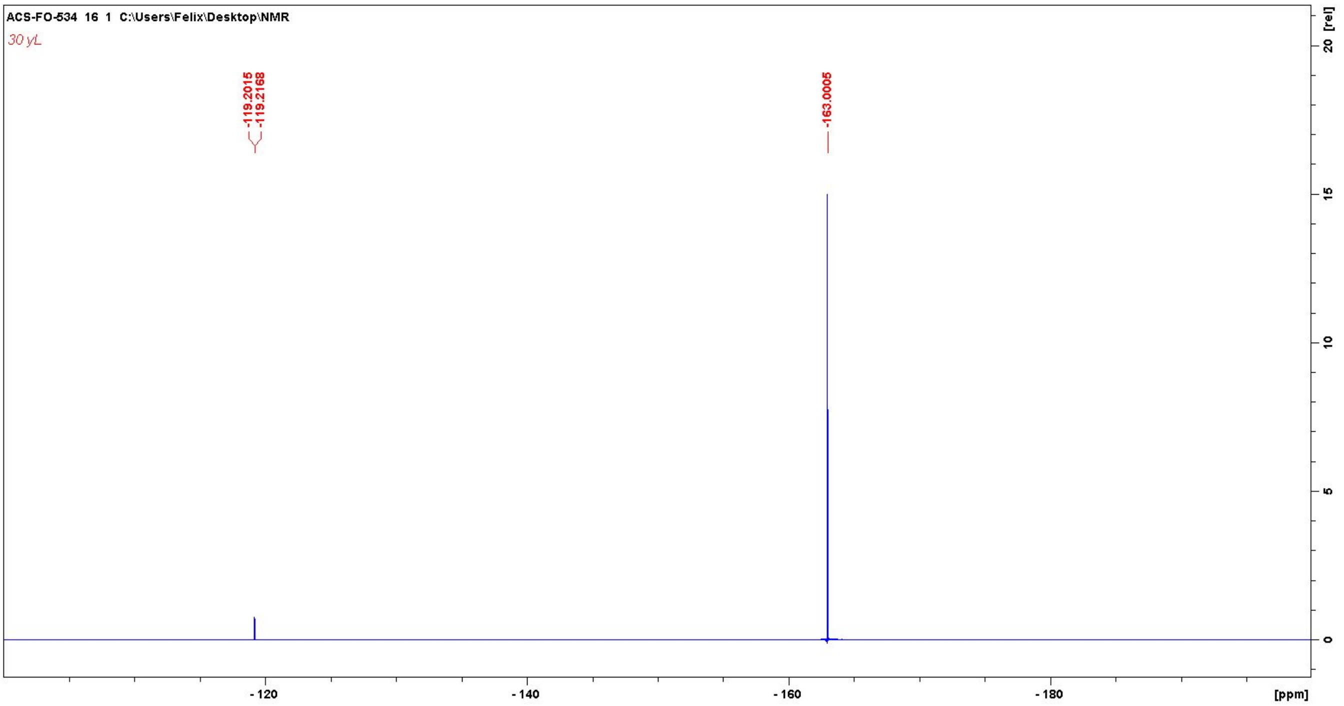

Figure S91: ^{19}F NMR spectra of 1,4-diiodtetrafluorobenzene (host) and quinuclidine (guest, 30 µL) in cyclohexane-d12. Chemical shifts are reported relative to hexafluorobenzene.

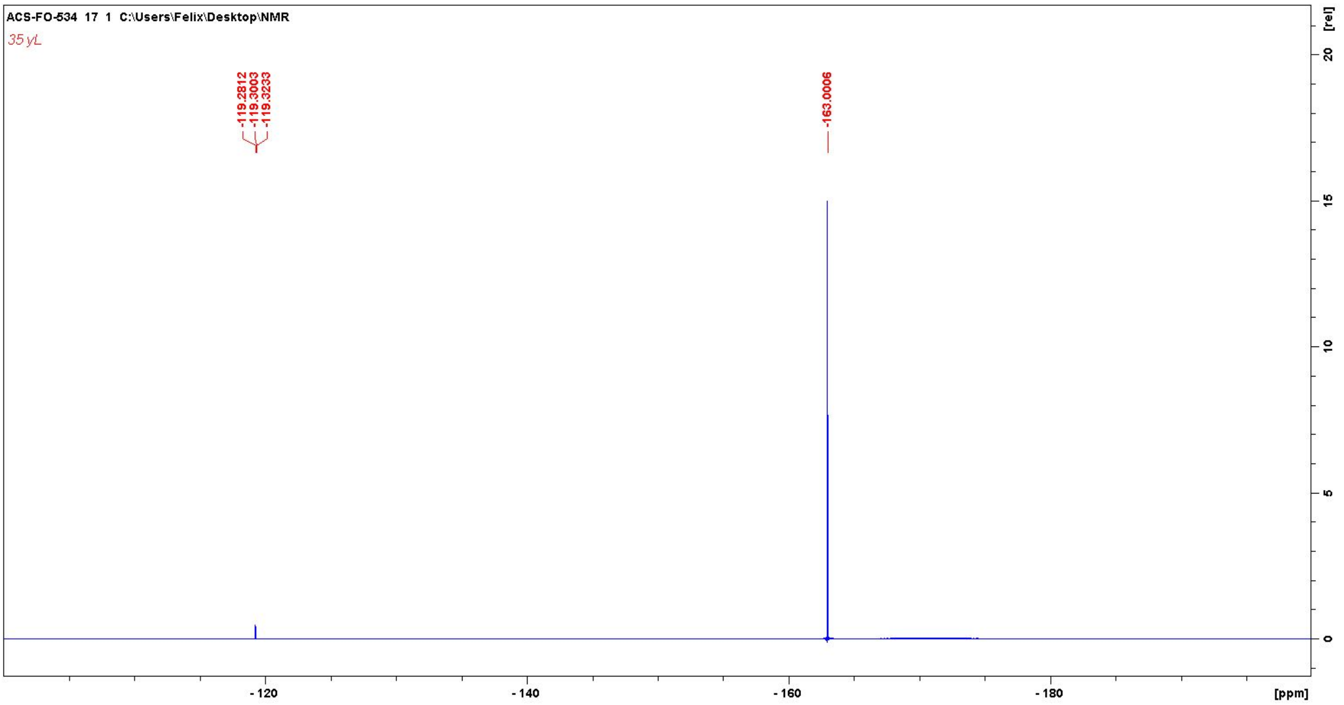

Figure S92: ^{19}F NMR spectra of 1,4-diiodtetrafluorobenzene (host) and quinuclidine (guest, 35 µL) in cyclohexane-d12. Chemical shifts are reported relative to hexafluorobenzene.

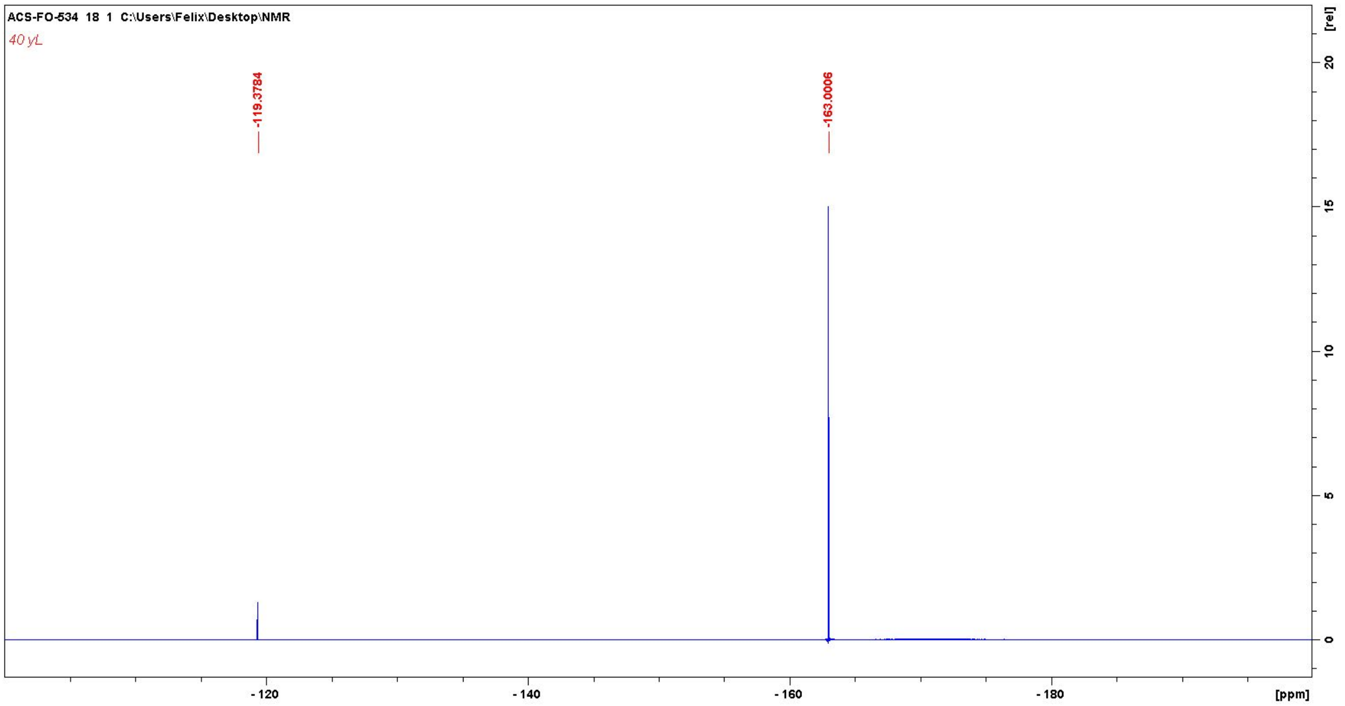

Figure S93: ^{19}F NMR spectra of 1,4-diiodtetrafluorobenzene (host) and quinuclidine (guest, 40 µL) in cyclohexane-d12. Chemical shifts are reported relative to hexafluorobenzene.

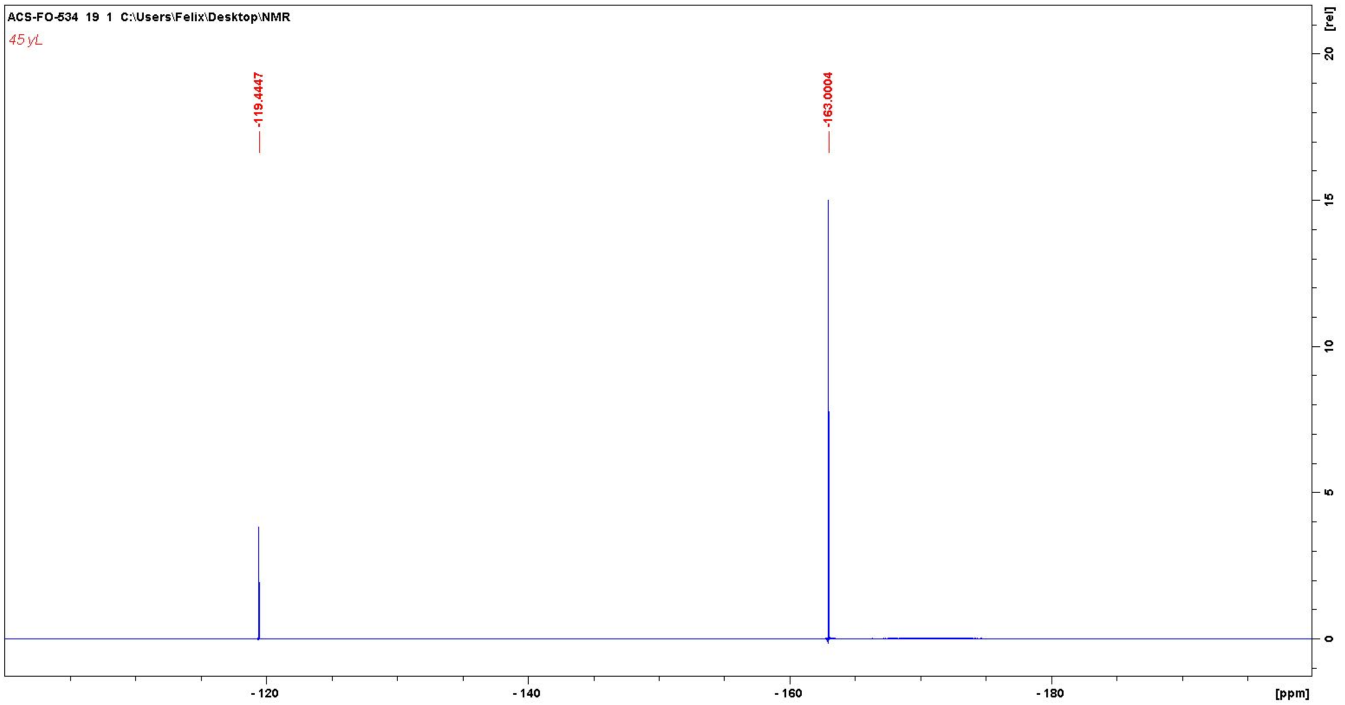

Figure S94: ^{19}F NMR spectra of 1,4-diiodtetrafluorobenzene (host) and quinuclidine (guest, 45 µL) in cyclohexane-d12. Chemical shifts are reported relative to hexafluorobenzene.

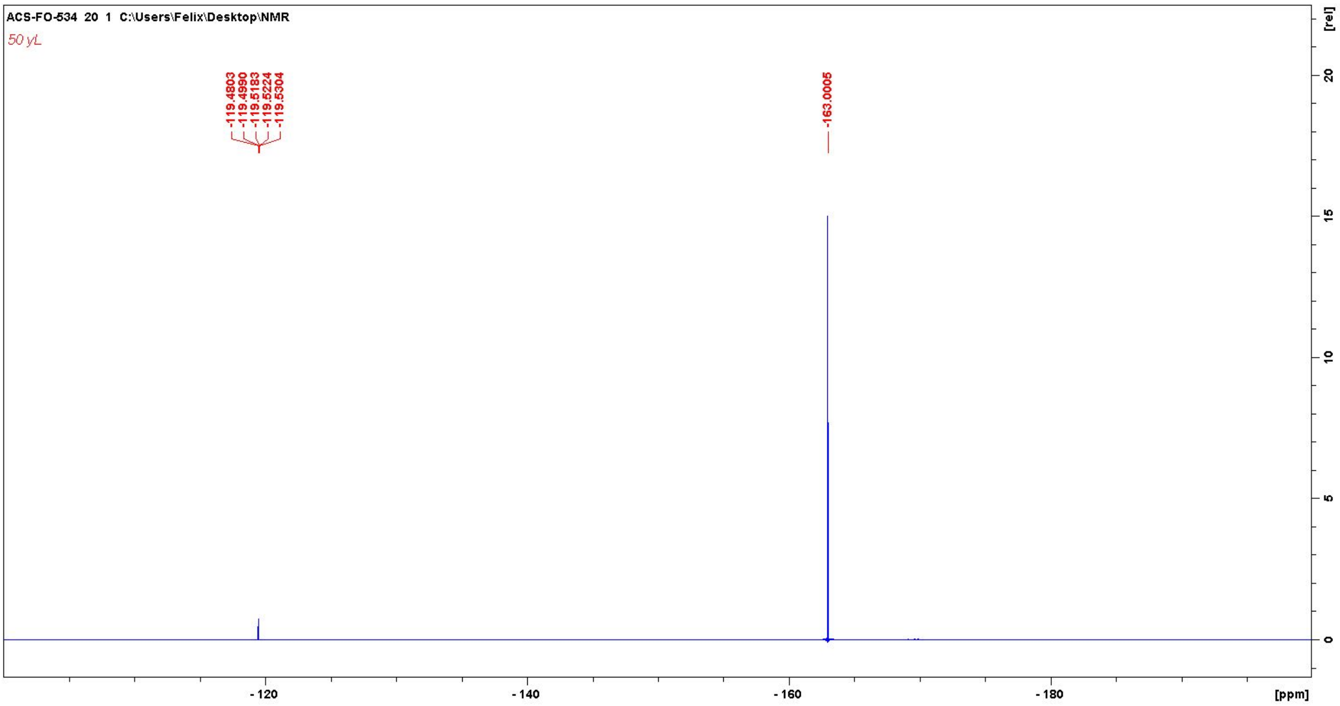

Figure S95: ^{19}F NMR spectra of 1,4-diiodtetrafluorobenzene (host) and quinuclidine (guest, 50 µL) in cyclohexane-d12. Chemical shifts are reported relative to hexafluorobenzene.

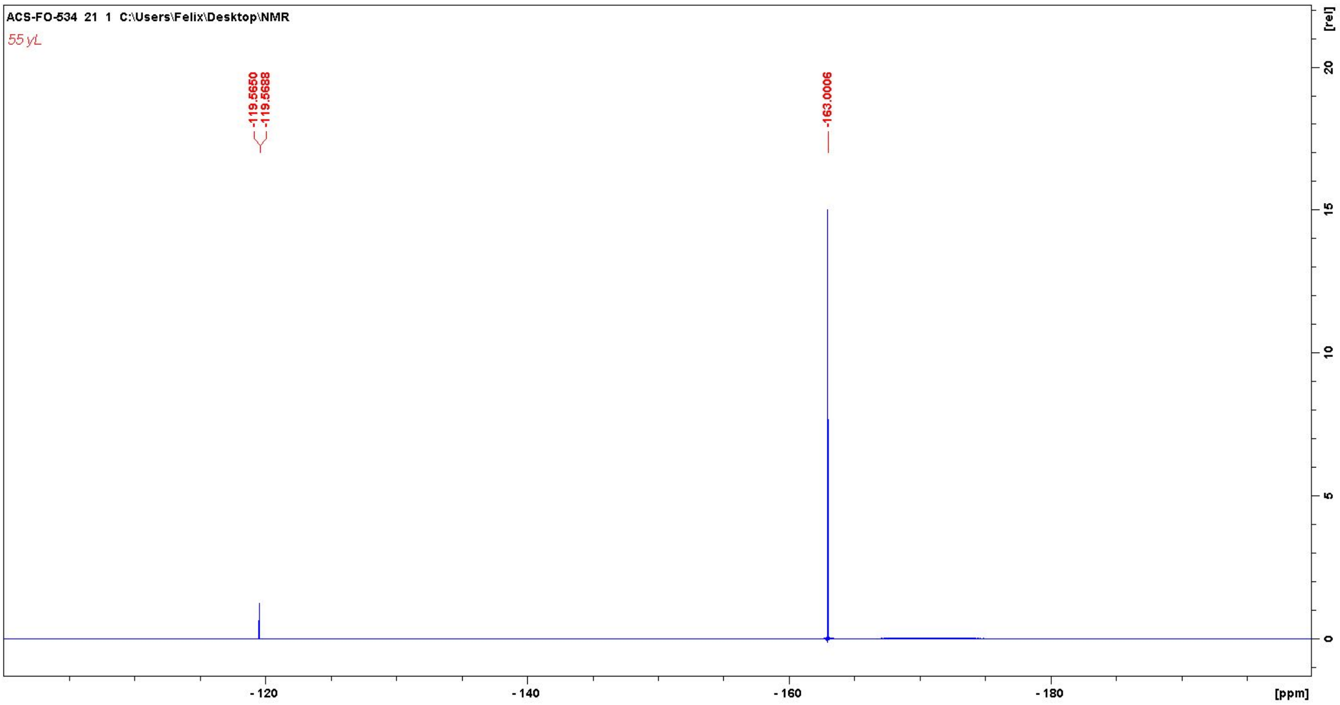

Figure S96: ^{19}F NMR spectra of 1,4-diiodtetrafluorobenzene (host) and quinuclidine (guest, 55 µL) in cyclohexane-d12. Chemical shifts are reported relative to hexafluorobenzene.

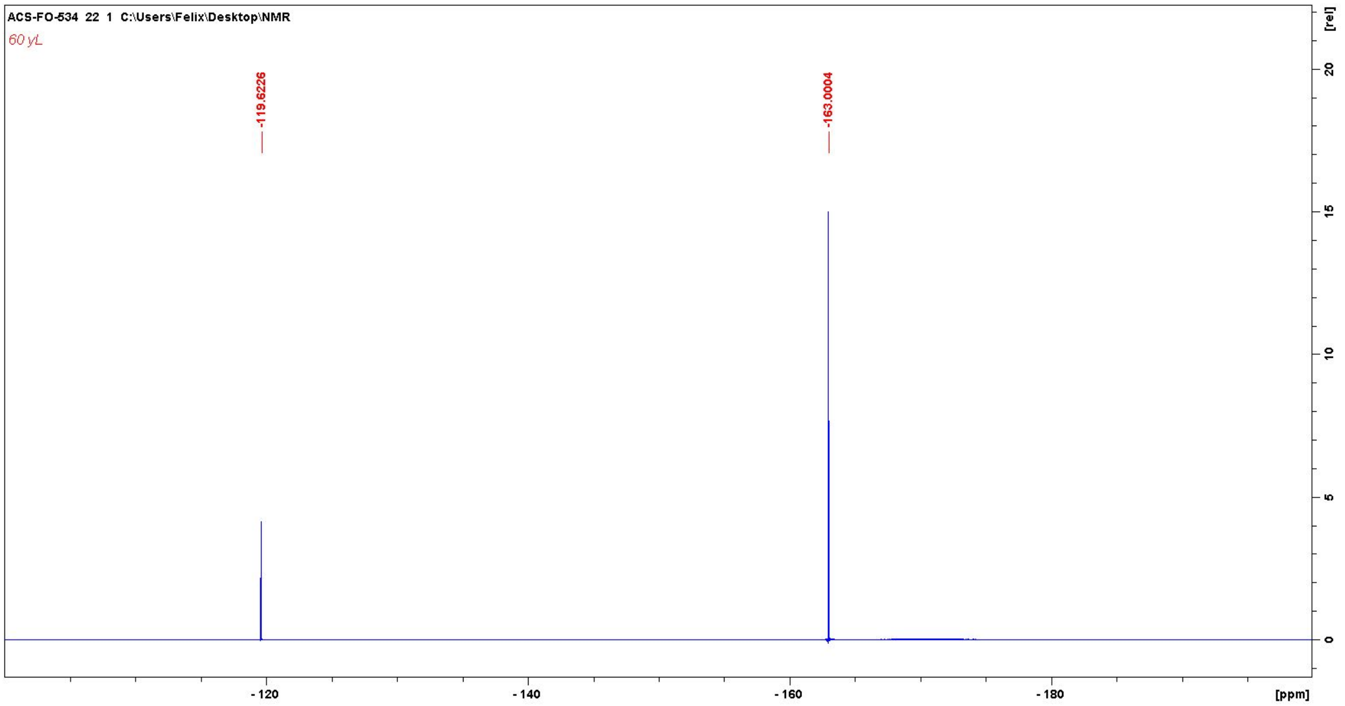

Figure S97: ^{19}F NMR spectra of 1,4-diiodtetrafluorobenzene (host) and quinuclidine (guest, 60 µL) in cyclohexane-d12. Chemical shifts are reported relative to hexafluorobenzene.

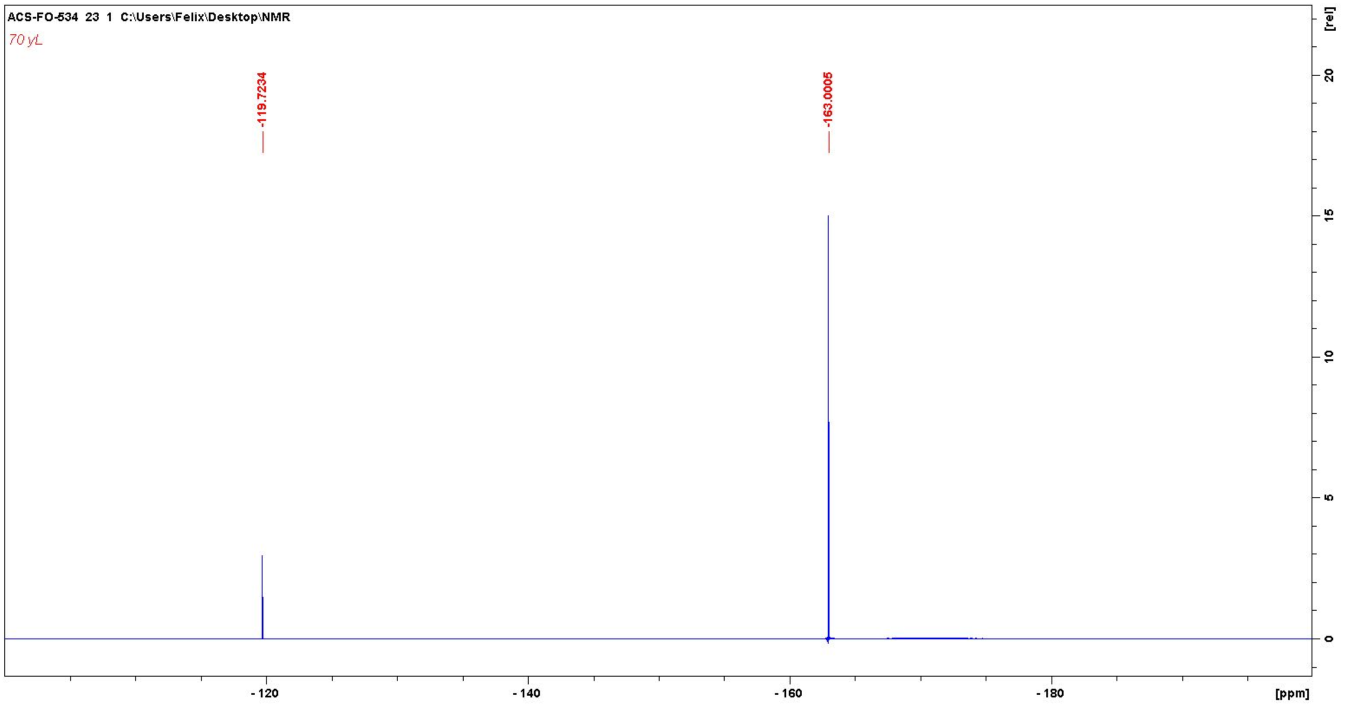

Figure S98: ^{19}F NMR spectra of 1,4-diiodtetrafluorobenzene (host) and quinuclidine (guest, 70 µL) in cyclohexane-d12. Chemical shifts are reported relative to hexafluorobenzene.

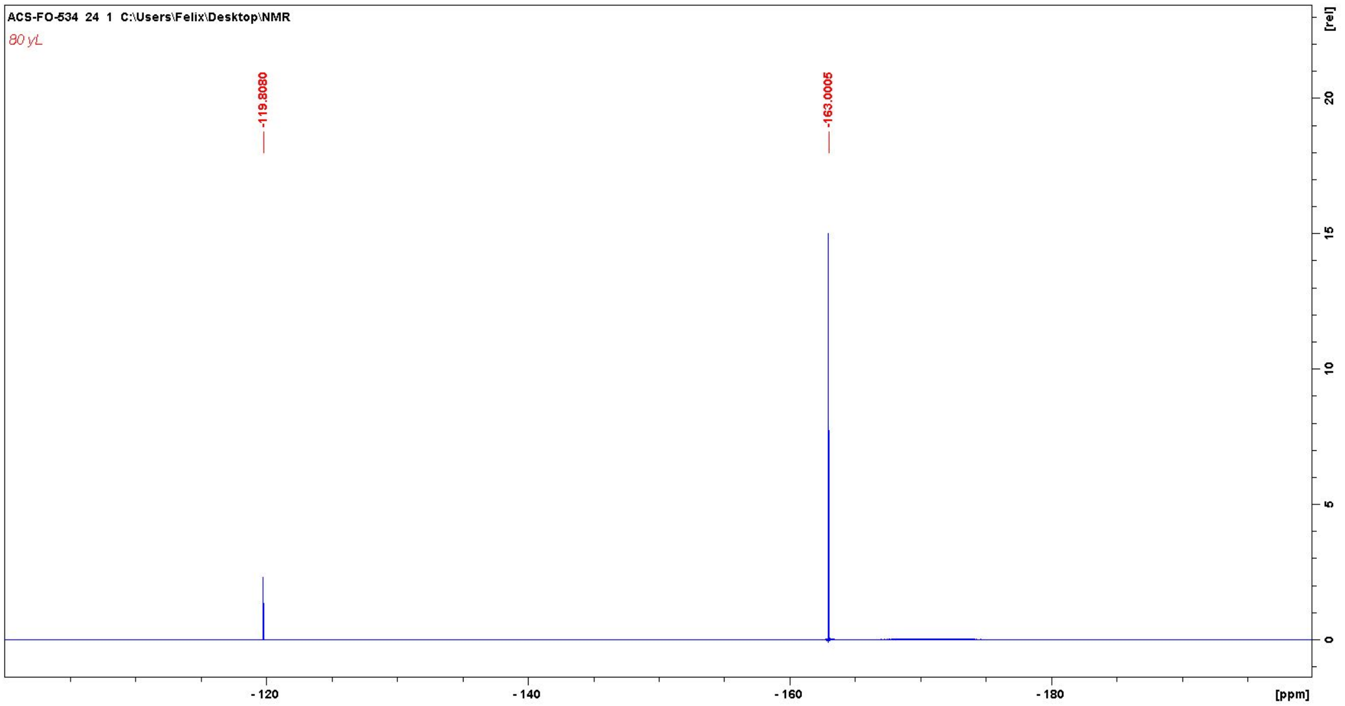

Figure S99: ^{19}F NMR spectra of 1,4-diiodtetrafluorobenzene (host) and quinuclidine (guest, 80 µL) in cyclohexane-d12. Chemical shifts are reported relative to hexafluorobenzene.

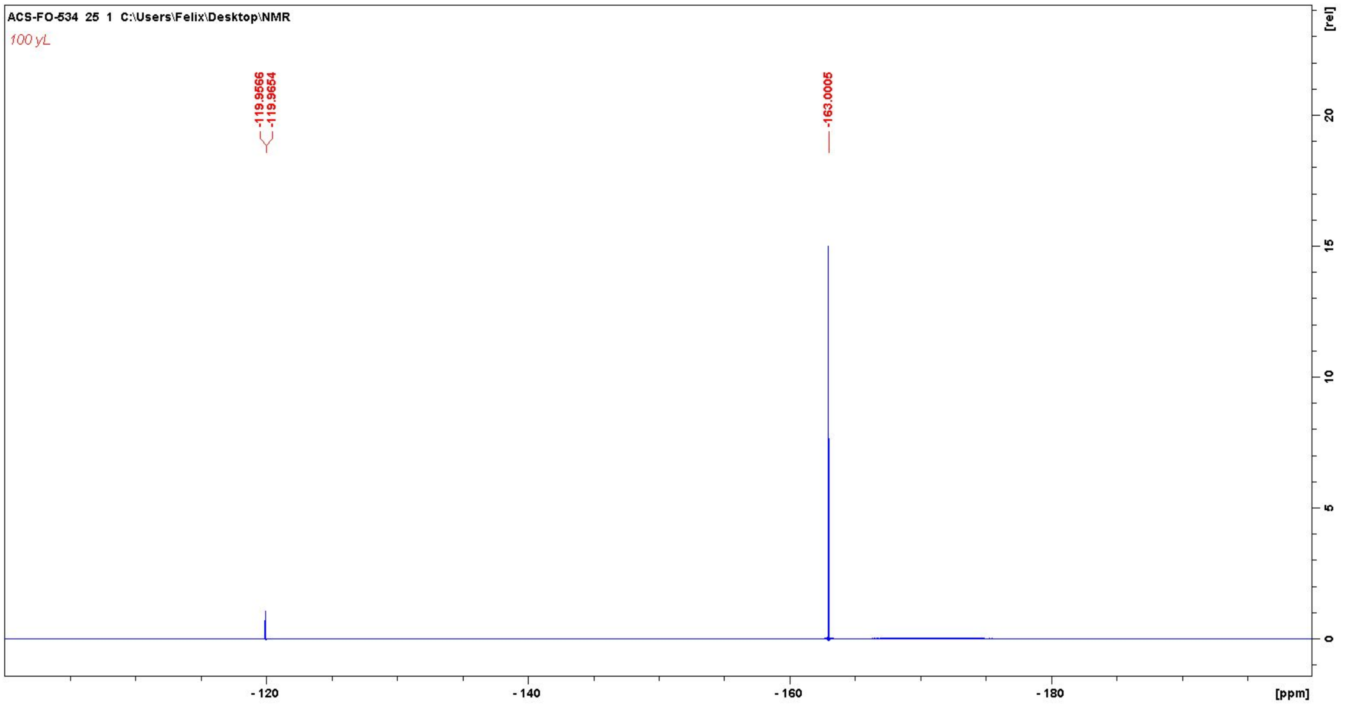

Figure S100: ^{19}F NMR spectra of 1,4-diiodtetrafluorobenzene (host) and quinuclidine (guest, 100 µL) in cyclohexane-d12. Chemical shifts are reported relative to hexafluorobenzene.

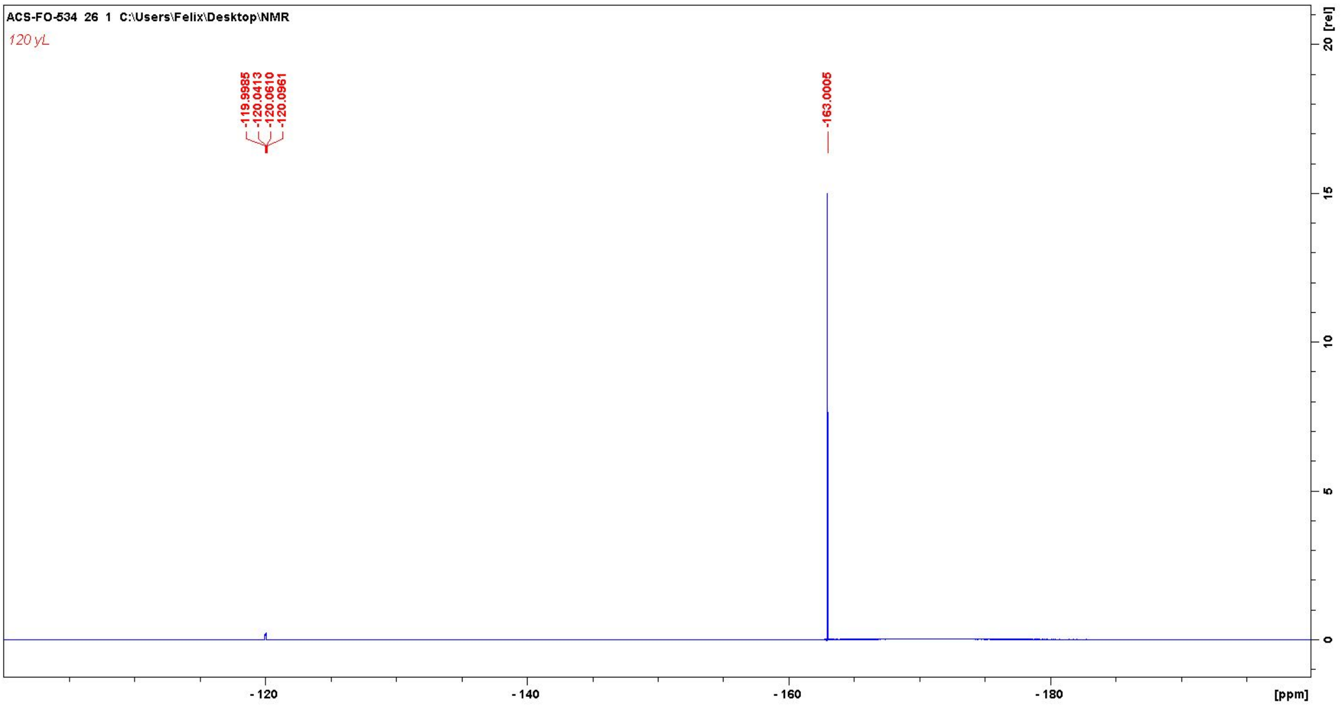

Figure S101: ^{19}F NMR spectra of 1,4-diiodtetrafluorobenzene (host) and quinuclidine (guest, 120 µL) in cyclohexane-d12. Chemical shifts are reported relative to hexafluorobenzene.

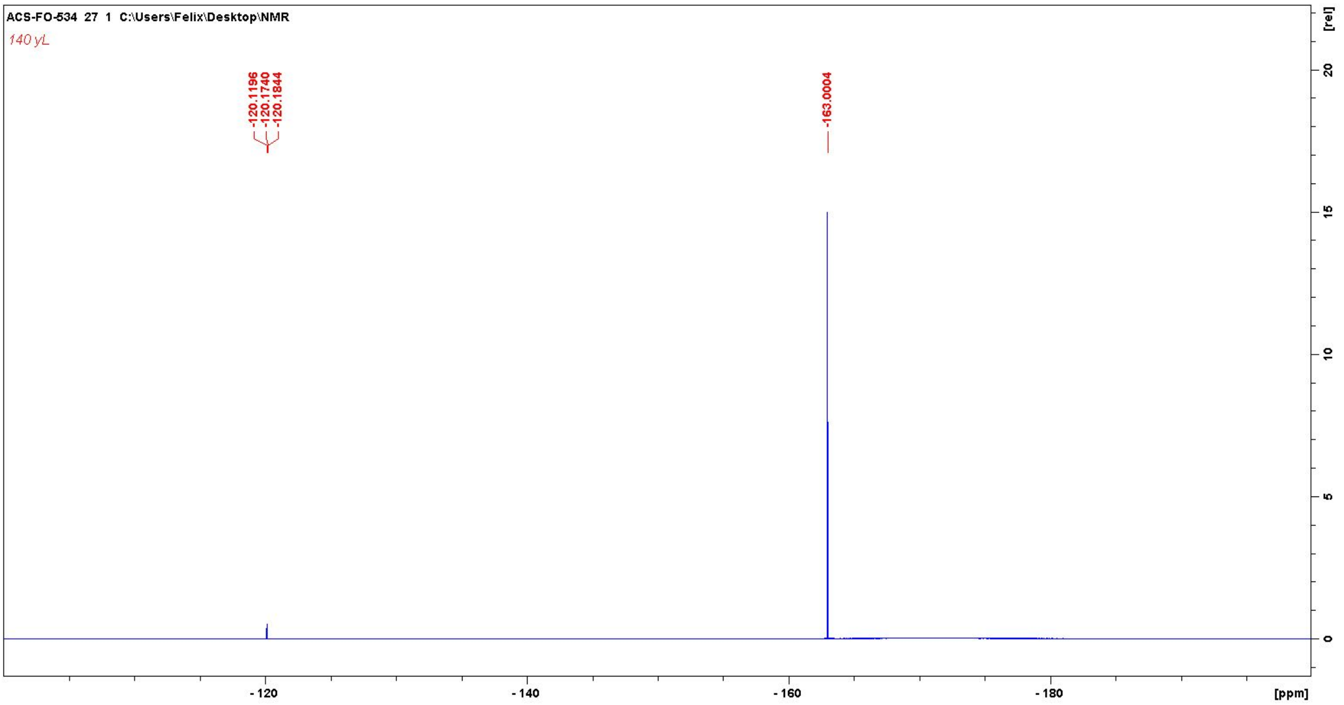

Figure S102: ^{19}F NMR spectra of 1,4-diiodtetrafluorobenzene (host) and quinuclidine (guest, 140 µL) in cyclohexane-d12. Chemical shifts are reported relative to hexafluorobenzene.

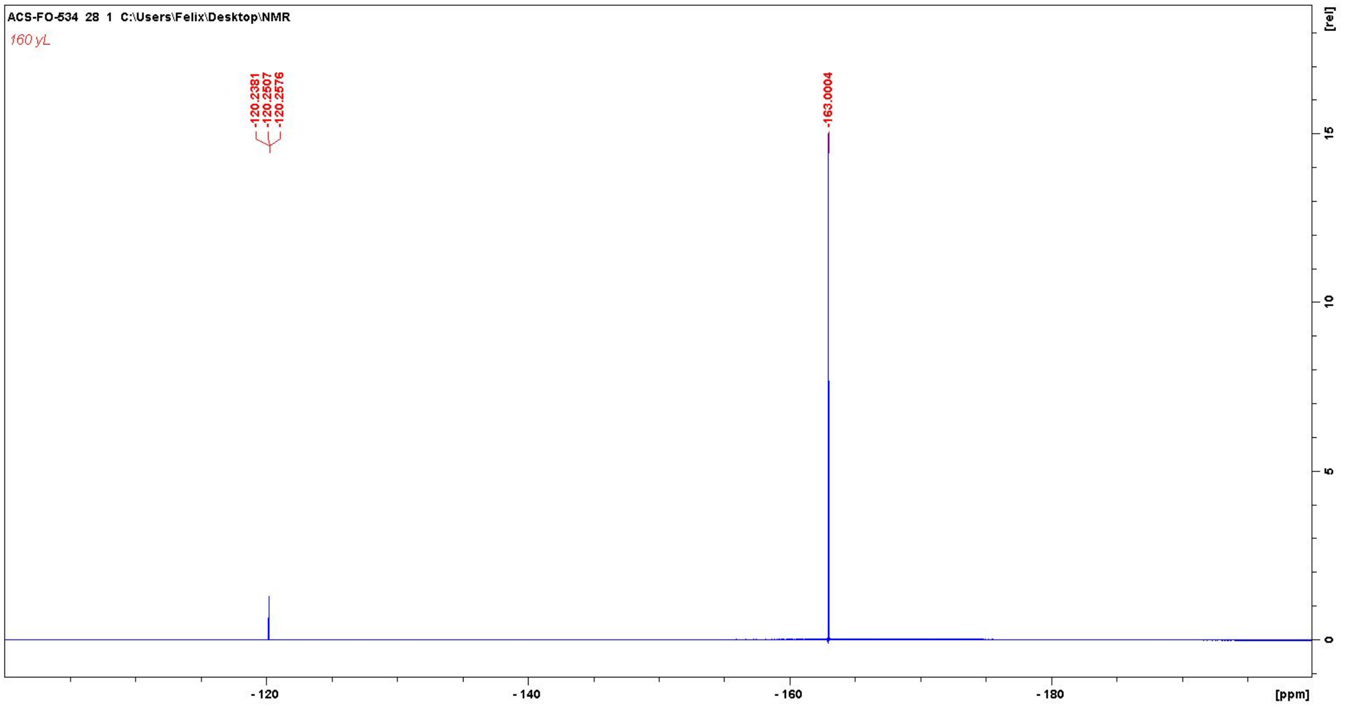

Figure S103: ^{19}F NMR spectra of 1,4-diiodtetrafluorobenzene (host) and quinuclidine (guest, 160 µL) in cyclohexane-d12. Chemical shifts are reported relative to hexafluorobenzene.

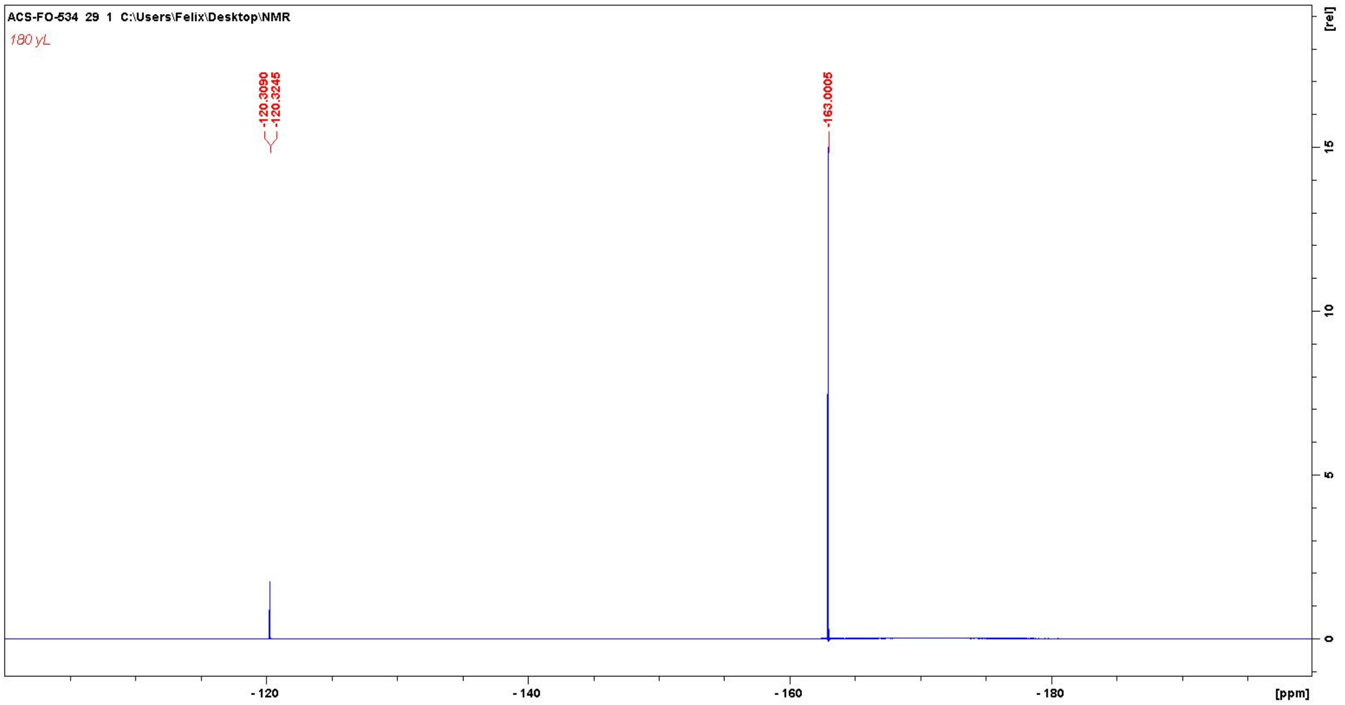

Figure S104: ^{19}F NMR spectra of 1,4-diiodtetrafluorobenzene (host) and quinuclidine (guest, 180 µL) in cyclohexane-d12. Chemical shifts are reported relative to hexafluorobenzene.

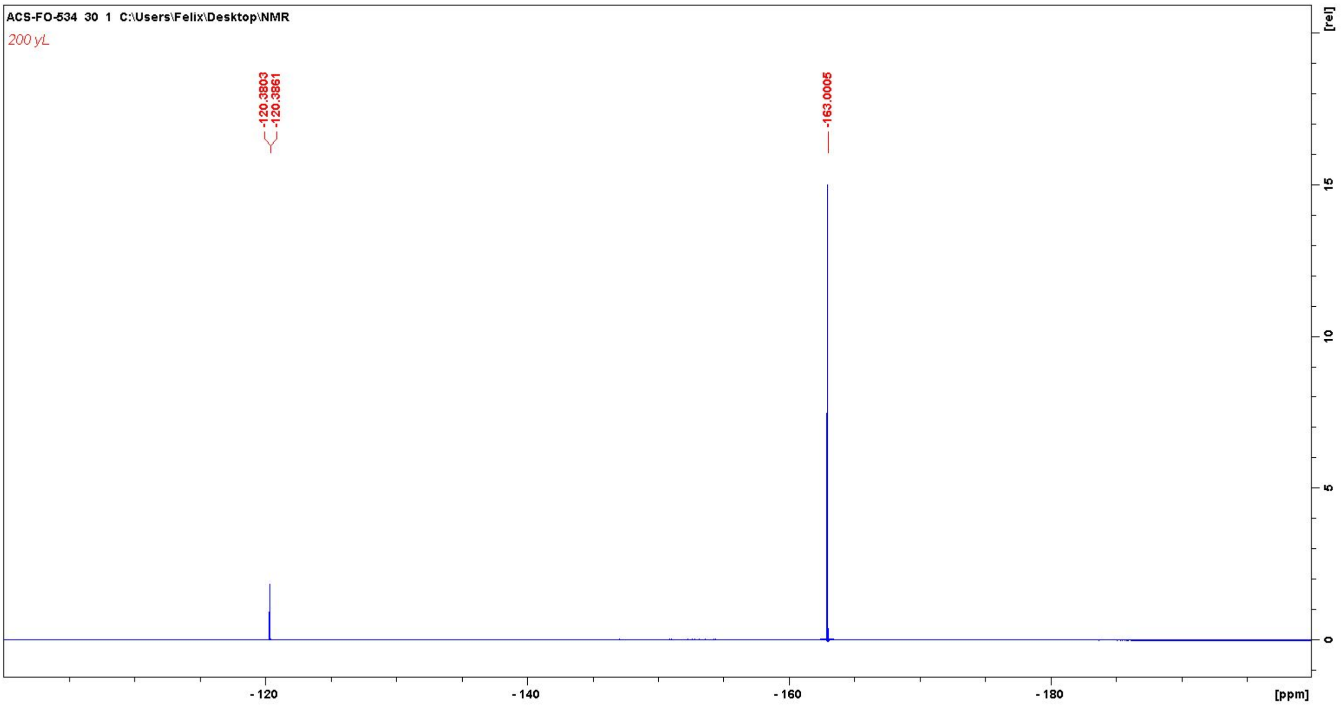

Figure S105: ^{19}F NMR spectra of 1,4-diiodtetrafluorobenzene (host) and quinuclidine (guest, 200 µL) in cyclohexane-d12. Chemical shifts are reported relative to hexafluorobenzene.

7 Raman spectroscopic data

The geometry optimization was done based on the crystal structures of the pure compounds as well as for the halogen bond adducts. All spectra calculations were done at DFT level using Gaussian 09 Rev. E.01 [23] carried out on the geometry optimized structures with b3lyp/6-311++G(2d,p) and the pseudo potential for iodine Lan2DZ. Raman spectra were simulated by assigning a uniform Lorentzian band shape of 2 cm^{-1} half-width at half-height to the computed dipole and rotational strength. The frequency scaling factor (x0.99) was determined empirically.

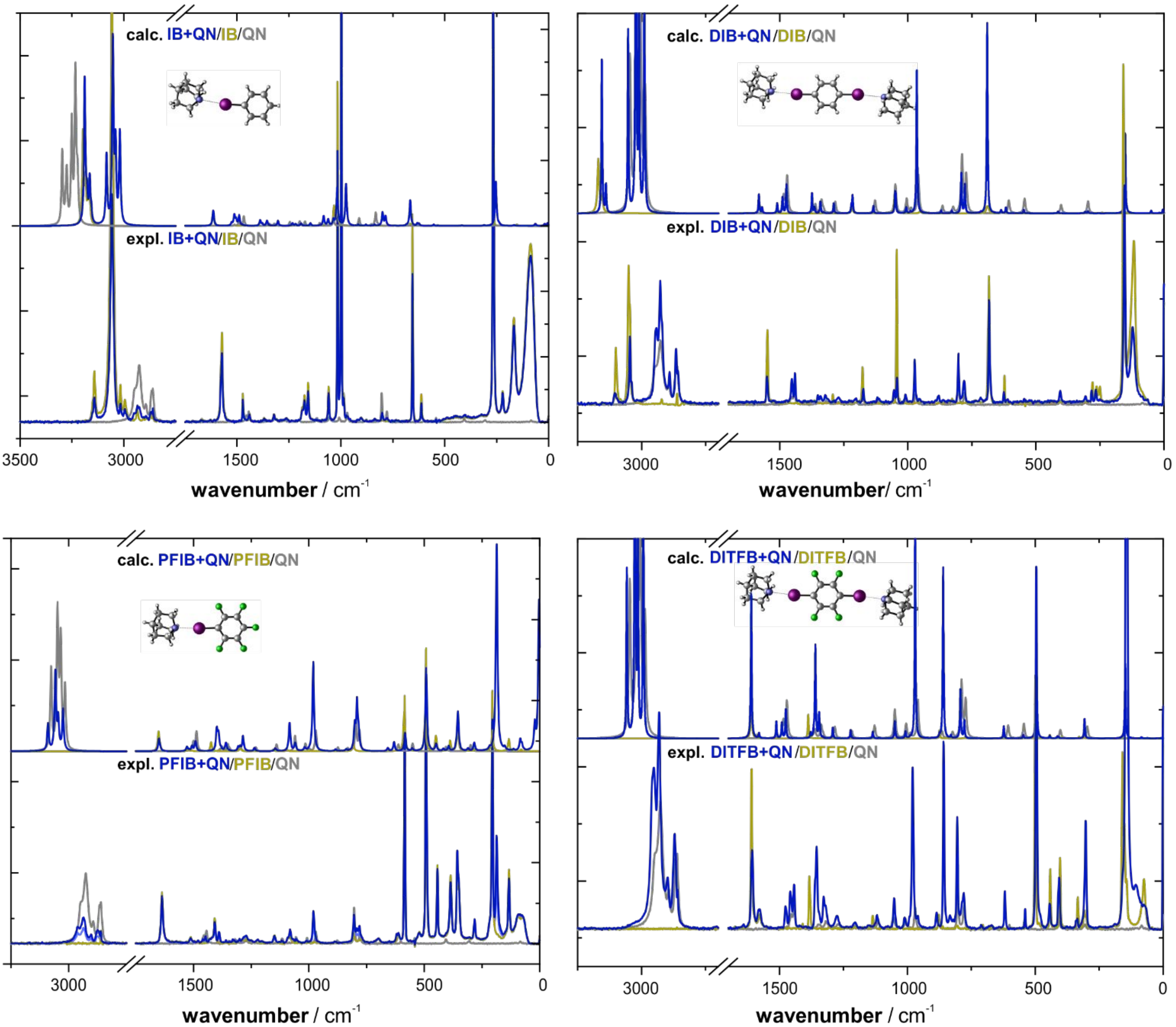

Figure S106: Comparison of experimental and calculated Raman spectra of the XB adducts and the respective starting materials.

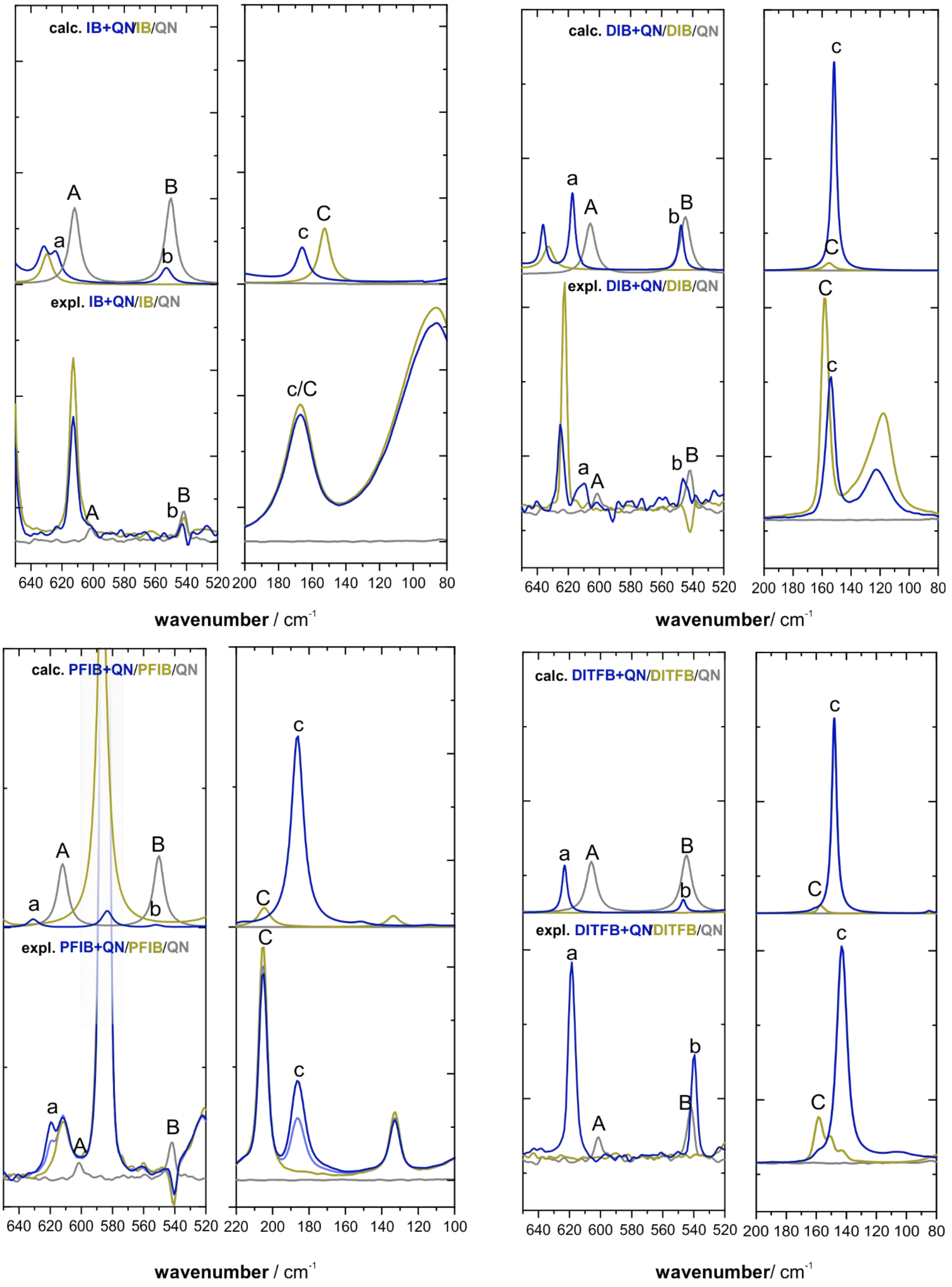

Figure S107: Enlarged view of band regions with the largest predicted shift in the Raman spectrum for the XB acceptor and the XB donor, respectively.

Table S45: Comparison of experimental vibrational shifts of the XB adducts **3**, **7**, **8**, **9** relative to the respective pure compounds. Shift A and B are belonging to the XB acceptor quinuclidine and shift C to the XB donors, respectively. Predicted values are noted in paratheses.

XB adduct	shift A [cm^{-1}]	shift B [cm^{-1}]	shift C [cm^{-1}]
**3**	X (–19)	–1 (–9)	0 (–14)
**7**	–18 (–25)	X (–8)	19 (19)
**8**	–9 (–11)	–5 (–3)	5 (4)
**9**	–17 (–17)	2 (–2)	15 (9)

Table S46: Optimized geometry for Raman spectrum simulation of quinuclidine **1**.

atom	x	y	z
C	1.21923200	0.70392400	–0.71867800
C	0.00000000	0.00000000	–1.28831300
C	0.85636200	1.06489100	0.76064200
H	2.03981500	0.02156500	–0.79569200
H	1.47066100	1.61253500	–1.22475000
H	1.76796000	1.18687500	1.30746000
H	0.28505500	1.96783700	0.81717200
C	0.00000000	–1.40784800	–0.71867800
H	–1.00123200	–1.77731400	–0.79569200
H	0.66116600	–2.07989700	–1.22475000
C	0.49404200	–1.27407700	0.76064200
H	0.14388400	–2.12453600	1.30746000
H	1.56166900	–1.23078300	0.81717200
H	0.00000000	0.00000000	–2.35831300
C	–1.35040400	0.20918600	0.76064200
H	–1.84672400	–0.73705400	0.81717200
H	–1.91184400	0.93766100	1.30746000
C	–1.21923200	0.70392400	–0.71867800
H	–2.13182700	0.46736200	–1.22475000
H	–1.03858300	1.75574900	–0.79569200
N	0.00000000	0.00000000	1.28861100

Table S47: Optimized geometry for Raman spectrum simulation of Iodobenzene **2**.

atom	x	y	z
C	–2.64396600	1.20209000	0.00000100
C	–3.34321100	0.00000200	–0.00000200
C	–2.64398400	–1.20208000	0.00000300
C	–1.25184700	–1.20880800	–0.00000100
C	–0.56607700	–0.00001100	–0.00000500
C	–1.25184400	1.20880700	0.00000100
I	1.55503200	0.00000000	0.00000000
H	–0.71394100	–2.14726500	–0.00000100
H	–3.17850200	–2.14459300	0.00000500
H	–4.42629200	0.00001600	–0.00000100
H	–3.17849300	2.14459800	0.00000300
H	–0.71391400	2.14725000	0.00000200

Table S48: Optimized geometry for Raman spectrum simulation of XB adduct **3**.

atom	x	y	z
C	–4.28081700	–0.00026300	1.44233400
C	–4.80754500	–0.00001100	–0.00368900
C	–2.72289400	–0.00024500	1.39036800
H	–4.65262500	0.87881700	1.97561400
H	–4.65261500	–0.87953600	1.97530300
H	–2.30756200	0.87755100	1.88972500
H	–2.30755200	–0.87821500	1.88941100
C	–4.27609500	1.25137000	–0.72480100
H	–4.64451100	1.27430900	–1.75395100
H	–4.64773700	2.15330700	–0.23104000
C	–2.71839400	1.20325500	–0.69381800
H	–2.29968400	1.19569900	–1.70228900
H	–2.30337700	2.07391900	–0.18175300
H	–5.89974100	–0.00001800	–0.00606600
C	–2.71837800	–1.20300200	–0.69424700
H	–2.29966900	–1.19508000	–1.70271600
H	–2.30334800	–2.07384300	–0.18249400
C	–4.27607800	–1.25112800	–0.72524500
H	–4.64449600	–1.27370800	–1.75440200
H	–4.64770700	–2.15324600	–0.23180300
N	–2.23304800	0.00000600	0.00198200
I	0.75651300	0.00000600	0.00209200
C	2.88683800	0.00000000	0.00030400
C	3.58308900	–1.20535700	–0.00029000
C	3.58309600	1.20535300	–0.00029100
C	4.97523500	–1.20174200	–0.00212800
H	3.04668900	–2.14569600	0.00015900
C	4.97524200	1.20172900	–0.00212800
H	3.04670300	2.14569600	0.00015800
C	5.67562100	–0.00000900	–0.00283900
H	5.51045300	–2.14424600	–0.00275400
H	5.51046700	2.14422900	–0.00275400
H	6.75893200	–0.00001200	–0.00428500

Table S49: Optimized geometry for Raman spectrum simulation of 1,4-diiodobenzene **4**.

atom	x	y	z
C	–0.69594100	1.20497800	–0.00000100
C	0.69594100	1.20497900	0.00000000
C	1.38681700	0.00001700	–0.00000200
C	0.69593200	–1.20495900	–0.00000100
C	–0.69593200	–1.20496000	–0.00000100
C	–1.38681700	0.00001700	–0.00000300
H	–1.22657300	2.14756800	–0.00000100
H	1.22657300	2.14756800	0.00000200
H	1.22658200	–2.14753900	0.00000100
H	–1.22658200	–2.14753900	0.00000000
I	3.50373000	–0.00000500	0.00000000
I	–3.50373000	–0.00000500	0.00000000

Table S50: Optimized geometry for Raman spectrum simulation of XB adduct **8**.

atom	x	y	z
I	–1.61848200	2.35140900	2.06915400
C	–0.64125100	0.93152200	0.81935200
C	0.66794700	1.14880500	0.40316000
H	1.20040000	2.04014900	0.71008100
C	1.30728000	0.22006600	–0.41357700
H	2.32660700	0.40418100	–0.72866800
I	1.61848200	–2.35140900	–2.06915400
C	0.64125100	–0.93152200	–0.81935200
C	–0.66794700	–1.14880500	–0.40316000
H	–1.20040000	–2.04014900	–0.71008100
C	–1.30728000	–0.22006600	0.41357700
H	–2.32660700	–0.40418100	0.72866800
N	–2.98153900	4.34104700	3.83221700
C	–4.66076100	6.19566100	3.92512700
H	–4.47216700	7.20942500	3.56163700
H	–5.74286800	6.04157800	3.89826100
C	–1.96842800	5.22366100	4.43413300
H	–1.41800800	5.70138500	3.62093100
H	–1.25827000	4.59444800	4.97465500
C	–3.92805200	5.14293200	3.03929300
H	–4.63535800	4.45559500	2.57057600
H	–3.36595100	5.62108100	2.23444400
C	–4.14440000	6.04948800	5.36754200
H	–4.63784800	6.77423200	6.01882800
C	–4.43138100	4.61807200	5.85438000
H	–5.50933300	4.43539600	5.86282600
H	–4.07724400	4.49465600	6.88140100
C	–3.70766400	3.62574500	4.89463800
H	–2.98609200	3.00643800	5.43142300
H	–4.41640700	2.94763400	4.41472500
C	–2.62289600	6.28011300	5.37501600
H	–2.23577900	6.18747400	6.39334000
H	–2.39715800	7.29522000	5.03733100
N	2.98153900	–4.34104700	–3.83221700
C	4.66076100	–6.19566100	–3.92512700
H	4.47216700	–7.20942500	–3.56163700
H	5.74286800	–6.04157800	–3.89826100
C	1.96842800	–5.22366100	–4.43413300
H	1.41800800	–5.70138500	–3.62093100
H	1.25827000	–4.59444800	–4.97465500
C	3.92805200	–5.14293200	–3.03929300
H	4.63535800	–4.45559500	–2.57057600
H	3.36595100	–5.62108100	–2.23444400
C	4.14440000	–6.04948800	–5.36754200
H	4.63784800	–6.77423200	–6.01882800
C	4.43138100	–4.61807200	–5.85438000
H	5.50933300	–4.43539600	–5.86282600
H	4.07724400	–4.49465600	–6.88140100
C	3.70766400	–3.62574500	–4.89463800
H	2.98609200	–3.00643800	–5.43142300
H	4.41640700	–2.94763400	–4.41472500
C	2.62289600	–6.28011300	–5.37501600
H	2.23577900	–6.18747400	–6.39334000
H	2.39715800	–7.29522000	–5.03733100

Table S51: Optimized geometry for Raman spectrum simulation of pentafluoroiodobenzene **5**.

atom	x	y	z
C	1.89167600	–1.19965600	0.00000400
C	2.58880700	0.00000200	0.00000600
C	1.89168300	1.19965500	–0.00000200
C	0.50372300	1.19219200	0.00000500
C	–0.21126900	0.00000300	0.00000500
C	0.50372400	–1.19219500	–0.00000300
F	–0.12464700	2.37052500	–0.00000500
F	2.55807000	2.35522500	–0.00000200
F	3.92020400	–0.00000800	–0.00000100
F	2.55807600	–2.35522000	–0.00000200
F	–0.12465700	–2.37052100	–0.00000400
I	–2.30365100	0.00000000	0.00000000

Table S52: Optimized geometry for Raman spectrum simulation of XB adduct **7**.

atom	x	y	z
C	5.15157900	–0.00073800	1.44464200
C	5.68090200	0.00000400	0.00013700
C	3.59536300	–0.00069600	1.39300700
H	5.52121100	–0.88002000	1.97828100
H	5.52122600	0.87798600	1.97918800
H	3.17798100	–0.87899200	1.88889500
H	3.17800100	0.87712000	1.88975800
C	5.15178000	–1.25073200	–0.72273600
H	5.52185100	–1.27338300	–1.75087900
H	5.52130800	–2.15295000	–0.22879900
C	3.59559300	–1.20616100	–0.69749600
H	3.17852900	–1.19618000	–1.70618800
H	3.17766100	–2.07545700	–0.18651400
H	6.77281000	0.00000800	0.00028100
C	3.59558500	1.20685200	–0.69629300
H	3.17854500	1.19785900	–1.70500400
H	3.17762300	2.07563500	–0.18446300
C	5.15177100	1.25147800	–0.72145400
H	5.52186400	1.27520100	–1.74956500
H	5.52127200	2.15319200	–0.22657500
N	3.10730000	–0.00000400	–0.00023600
I	0.30126400	–0.00000100	–0.00013800
C	–1.83152300	0.00000000	0.00050900
C	–2.55385800	1.18606600	0.00049700
C	–2.55385800	–1.18606500	0.00049600
C	–3.94168700	1.19880400	–0.00002900
C	–3.94168800	–1.19880300	–0.00002900
C	–4.63953800	0.00000000	–0.00022800
F	–1.92701600	–2.37124200	0.00080000
F	–4.61109300	–2.35619900	–0.00022600
F	–5.97384100	0.00000000	–0.00065800
F	–4.61109300	2.35619900	–0.00022600
F	–1.92701600	2.37124300	0.00080000

Table S53: Optimized geometry for Raman spectrum simulation of 1,4-diiodotetrafluorobenzene **6**.

atom	x	y	z
C	–0.69385300	1.18887700	–0.00001100
C	0.69385300	1.18887700	–0.00001800
C	1.41176700	0.00000400	–0.00001100
C	0.69385000	–1.18887600	–0.00001200
C	–0.69385000	–1.18887600	–0.00001800
C	–1.41176700	0.00000300	–0.00001100
I	3.50267400	–0.00000100	0.00000500
I	–3.50267400	–0.00000100	0.00000500
F	1.32197300	–2.36862600	–0.00000100
F	–1.32197300	–2.36862600	0.00000100
F	1.32196800	2.36863100	0.00000200
F	–1.32196800	2.36863100	–0.00000200

Table S54: Optimized geometry for Raman spectrum simulation of XB adduct **9**.

atom	x	y	z
I	3.54522600	0.00576400	–0.01431400
F	1.32154800	–2.36312800	–0.02075200
F	–1.32324400	–2.36225500	–0.01990700
C	1.41946800	0.00726400	–0.02007000
C	0.69326700	–1.17530600	–0.02060400
C	–0.69416200	–1.17484300	–0.02017100
I	–3.54535600	0.00814200	–0.01279800
F	–1.32185600	2.37850700	–0.01877000
F	1.32333600	2.37763400	–0.01976200
C	–1.41957800	0.00820800	–0.01924600
C	–0.69340500	1.19078800	–0.01960600
C	0.69408600	1.19033100	–0.02010000
N	6.38738900	–0.00334300	0.00824800
C	8.96076800	–0.01658000	0.03873800
H	10.05270900	–0.02221600	0.05169700
C	6.89288700	0.62117200	–1.22882800
H	6.47963700	1.63009400	–1.28329000
H	6.48476400	0.06163400	–2.07267900
C	8.42301100	–1.45637800	0.11084200
H	8.78135700	–1.94445200	1.02089100
H	8.79863700	–2.03863500	–0.73457800
C	6.86685300	–1.39598900	0.09234000
H	6.45474600	–1.94002100	–0.75960100
H	6.43807200	–1.83975000	0.99281200
C	8.45010600	0.63947900	–1.25589800
H	8.82924100	0.09625100	–2.12536400
H	8.82463900	1.66377700	–1.32908100
C	6.86513800	0.75687900	1.17865400
H	6.43306700	0.29779700	2.06987700
H	6.45530600	1.76615300	1.10760800
C	8.42113400	0.77534800	1.24246000
H	8.79815200	1.80098100	1.21501100
H	8.77651100	0.32777800	2.17425300
N	–6.38722300	–0.00115300	0.00831600
C	–8.96061800	–0.02043900	0.03736700
H	–10.05254900	–0.02868300	0.04966800
C	–6.86302500	0.41454900	1.34127000
H	–6.43210200	–0.26984800	2.07450500
H	–6.45088200	1.40446800	1.54592300
C	–8.45060000	0.95929000	–1.03376300
H	–8.83003400	0.66922500	–2.01698100
H	–8.82543600	1.96551600	–0.82923300
C	–6.89339500	0.93493800	–1.01319000
H	–6.48033300	1.92099000	–0.79247800
H	–6.48527700	0.62511600	–1.97712400
C	–8.41902500	0.41827500	1.40906200
H	–8.79387600	1.41440400	1.65812400
H	–8.77498000	–0.26227600	2.18686500
C	–6.86794900	–1.36429900	–0.28671200
H	–6.45893300	–1.65709900	–1.25553600
H	–6.43717700	–2.03575600	0.45839500
C	–8.42405300	–1.42697400	–0.28103400
H	–8.78066600	–2.14123200	0.46561800
H	–8.80233200	–1.76086800	–1.25070300

8 References

(1) Dolomanov, O. V.; Bourhis, L. J.; Gildea, R. J.; Howard, J. A. K.; Puschmann, H. OLEX2: A Complete Structure Solution, Refinement and Analysis Program. *J. Appl. Crystallogr.* **2009**, *42* (2), 339–341. https://doi.org/10.1107/S0021889808042726.

(2) Sheldrick, G. M. SHELXT - Integrated Space-Group and Crystal-Structure Determination. *Acta Crystallogr. Sect. A Found. Crystallogr.* **2015**, *71* (1), 3–8. https://doi.org/10.1107/S2053273314026370.

(3) Sheldrick, G. M. A Short History of SHELX. *Acta Crystallogr. Sect. A Found. Crystallogr.* **2008**, *64* (1), 112–122. https://doi.org/10.1107/S0108767307043930.

(4) Kottke, T.; Stalke, D. Crystal Handling at Low Temperatures. *J. Appl. Crystallogr.* **1993**, *26*, 615–619. https://doi.org/10.1107/S0021889893002018.

(5) Groom, C. R.; Bruno, I. J.; Lightfoot, M. P.; Ward, S. C. *Acta Crystallogr., Sect. B: Struct. Sci.* **2016**, *72*, 171–179. https://doi.org/10.1107/S2052520616003954

(6) Bader, R. F. W. *Atoms in Molecules - a Quantum Theory*; Clarendon Press: Oxford, **1990**. ISBN:9780198551683.

(7) Hübschle, C. B.; Dittrich, B. MoleCoolQT – a molecule viewer for charge-density research. *J. Appl. Cryst.* **2011**, *44*, 238–240. https://doi.org/10.1107/S0021889810042482.

(8) Abramov, Yu. A. *Acta Cryst.* **1997**, *A53*, 264–272. https://doi.org/10.1107/S010876739601495X

(9) Espinosa, E., Molins, E., Lecomte, C. *Chem. Phys. Lett.* **1998**, *285*, 170–173. https://doi.org/10.1016/S0009-2614(98)00036-0

(10) Espinosa, E., Lecomte, C., Molins, E. *Chem. Phys. Lett.* **1999**, *300*, 745–748. https://doi.org/10.1016/S0009-2614(98)01399-2

(11) Turner, M J.; McKinnon, J. J.; Wolff, S. K.; Grimwood, D. J.; Spackman, P. R.; Jayatilaka, D.; Spackman, M. A. *CrystalExplorer17* **2017**, The University of Western Australia.

(12) Spackman, P. R.; Turner, M. J.; McKinnon, J. J.; Wolff, S. K.; Grimwood, D. J.; Jayatilaka, D.; Spackman, M. A. Christal Explorer: a program for Hirshfeld surface analysi, visualization and quantitative analysis of molecular crystals. *J. Appl. Cryst.* **2021**, *54*, 1006–1011. https://doi.org/10.1107/S1600576721002910

(13) Turner, M. J.; Grabowsky, S.; Jayatilaka, D.; Spackman, M. A. Accurate and Efficient Model Energies for Exploring Intermolecular Interactions in Molecular Crystals. *J. Phys. Chem. Lett.* **2014**, *5*, 4249–4255; https://doi.org/10.1021/jz502271c.

(14) Frisch, M. J.; Trucks, G. W.; Schlegel H. B.; Scuseria, G. E.; Robb, M. A.; Cheeseman, J. R.; Scalmani, G.; Barone, V.; Petersson, G. A.; Nakatsuji, H.; Li, X.; Caricato, M.; Marenich, A. V.; Bloino, J.; Janesko,

B. G.; Gomperts, R.; Mennucci, B.; Hratchian, H. P.; Ortiz, J. V.; Izmaylov, A. F.; Sonnenberg, J. L.; Williams-Young, D.; Ding, F.; Lipparini, F.; Egidi, F.; Goings, J.; Peng, B.; Petrone, A.; Henderson, T.; Ranasinghe, D.; Zakrzewski, V. G.; Gao, J.; Rega, N.; Zheng, G.; Liang, W.; Hada, M.; Ehara, M.; Toyota, K.; Fukuda, R.; Hasegawa, J.; Ishida, M.; Nakajima, T.; Honda, Y.; Kitao, O.; Nakai, H.; Vreven, T.; Throssell, K.; Montgomery, Jr., J. A.; Peralta, J. E.; Ogliaro, F.; Bearpark, M. J.; Heyd, J. J.; Brothers, E. N.; Kudin, K. N.; Staroverov, V. N.; Keith, T. A.; Kobayashi, R.; Normand, J.; Raghavachari, K.; Rendell, A. P.; Burant, J. C.; Iyengar, S. S.; Tomasi, J.; Cossi, M.; Millam, J. M.; Klene, M.; Adamo, C.; Cammi, R.; Ochterski, J. W.; Martin, R. L.; Morokuma, K.; Farkas, O.; Foresman, J. B.; Fox, D. J. Gaussian 16, Revision B.01. Gaussian Inc.: Wallingford CT **2016**.

(15) Neese, F. The ORCA Program System. *WIREs Comput. Mol. Sci.* **2012**, *2* (1), 73–78. https://doi.org/10.1002/wcms.81.

(16) Neese, F. Software Update: The ORCA Program System, Version 4.0. *WIREs Comput. Mol. Sci.* **2018**, *8* (1), 4–9. https://doi.org/10.1002/wcms.1327.

(17) Becke, A. D. Density-Functional Thermochemistry. III. The Role of Exact Exchange. *J. Chem. Phys.* **1993**, *98* (7), 5648–5652. https://doi.org/10.1063/1.464913

(18) Lee, C.; Yang, W.; Parr, R. G. Development of the Colle-Salvetti Correlation-Energy Formula into a Functional of the Electron Density. *Phys. Rev.* **1988**, *B37*, 785–789. https://doi.org/10.1103/PhysRevB.37.785

(19) Pritchard, B. P.; Altarawy, D.; Didier, B.; Gibson, T. D.; Windus, T. L. New Basis Set Exchange: An Open, Up-to-Date Resource for the Molecular Sciences Community. *J. Chem. Inf. Model.* **2019**, *59* (11), 4814–4820. https://doi.org/10.1021/acs.jcim.9b00725.

(20) Grimme, S.; Antony, J.; Ehrlich, S.; Krieg, H. A Consistent and Accurate Ab Initio Parametrization of Density Functional Dispersion Correction (DFT-D) for the 94 Elements H-Pu. *J. Chem. Phys.* **2010**, *132* 154104-1–19. https://doi.org/10.1063/1.3382344

(21) Burgenmeister, B.; Sonnenberg, K.; Riedel, S.; Krossing, I. From Square-Planar [ICl4]− to Novel Chloroiodates(III)? A Systematic Experimental and Theoretical Investigation of Their Ionic Liquids. *Chem. Eur. J.* **2017**, *23* (47), 11312–11322. https://doi.org/10.1002/chem.201701555.

(22) Macomber, R. S. An Introduction to NMR Titration for Studying Rapid Reversible Complexation. *J. Chem. Educ.* **1992**, *69* (5), 375–378. https://doi.org/10.1021/ed069p375.

(23) Frisch, M. J.; Trucks, G. W.; Schlegel, H. B.; Scuseria, G. E.; Robb, M. A.; Cheeseman, J. R.; Scalmani, G.; Barone, V.; Mennucci, B.; Petersson, G. A.; Nakatsuji, H.; Caricato, M.; Li, X.; Hratchian, H. P.; Izmaylov, A. F.; Bloino, J.; Zheng, G.; Sonnenberg, J. L.; Hada, M.; Ehara, M.; Toyota, K.; Fukuda, R.; Hasegawa, J.; Ishida, M.; Nakajima, T.; Honda, Y.; Kitao, O.; Nakai, H.; Vreven, T.; Montgomery, Jr., J. A.; Peralta, J. E.; Ogliaro, F.; Bearpark, M.; Heyd, J. J.; Brothers, E.; Kudin, K. N.; Staroverov, V. N.; Keith, T.; Kobayashi, R.; Normand, J.; Raghavachari, K.; Rendell, A.; Burant, J. C.; Iyengar, S. S.; Tomasi, J.; Cossi, M.; Rega, N.; Millam, J. M.; Klene, M.; Knox, J. E.; Cross, J. B.; Bakken, V.; Adamo, C.; Jaramillo, J.; Gomperts, R.; Stratmann, R. E.; Yazyev, O.; Austin, A. J.; Cammi, R.; Pomelli, C.; Ochterski, J. W.; Martin, R. L.; Morokuma, K.; Zakrzewski, V. G.; Voth, G. A.; Salvador, P.; Dannenberg, J. J.; Dapprich, S.; Daniels, A. D.; Farkas, O.; Foresman, J. B.; Ortiz, J. V.; Cioslowski, J.; Fox, D. J. Gaussian 09, Revision E.01, Gaussian Inc., Wallingford CT, **2013**.
